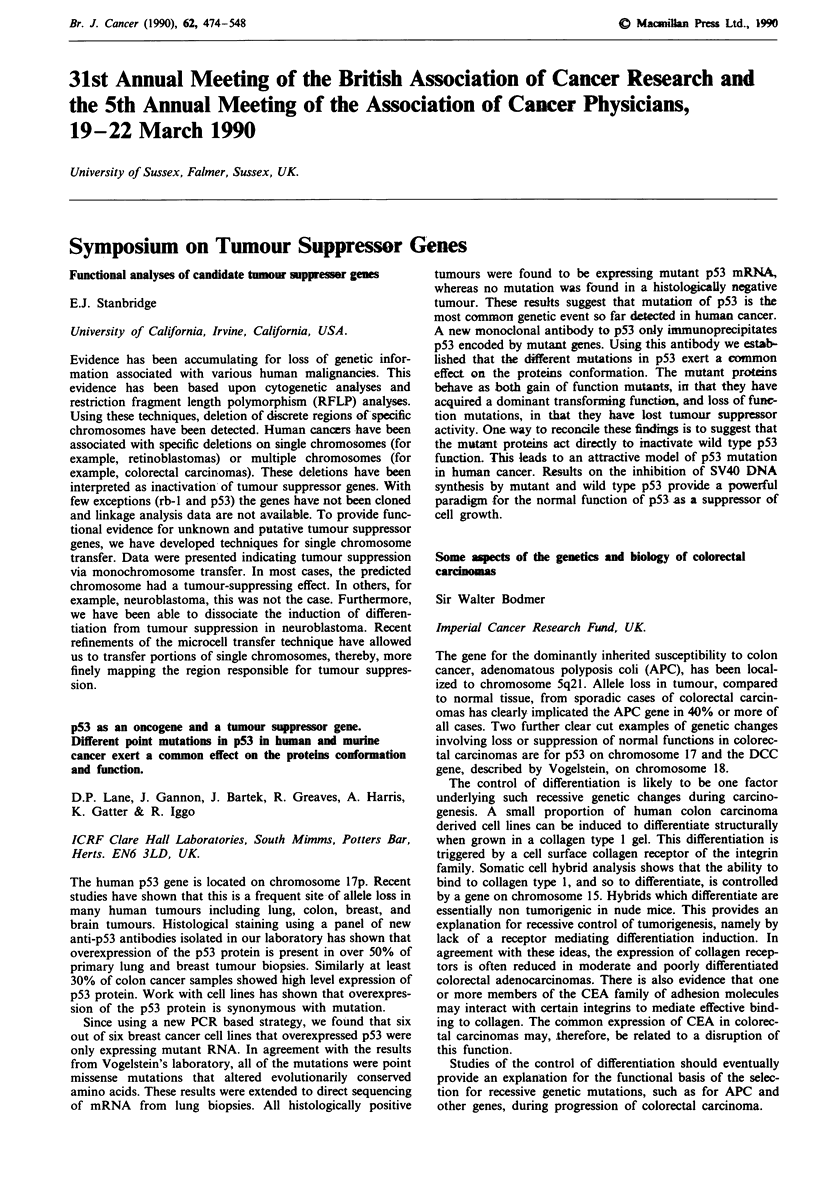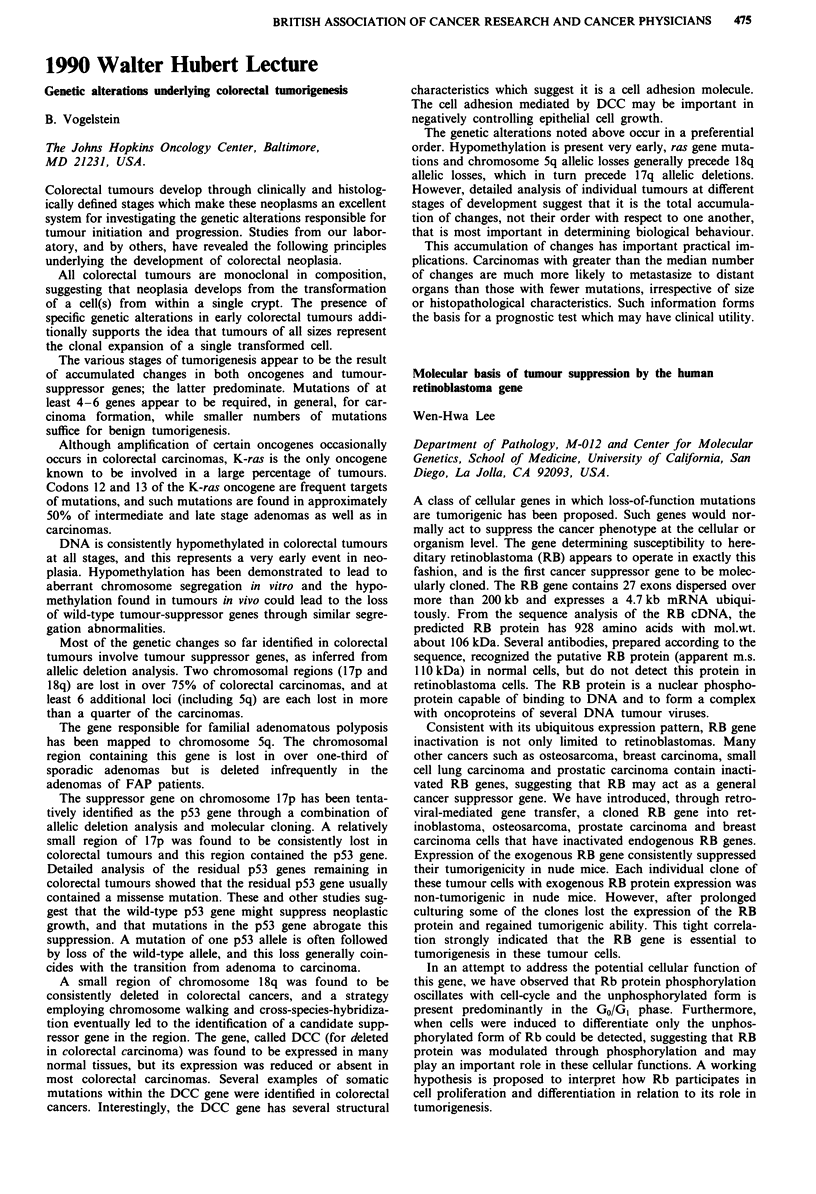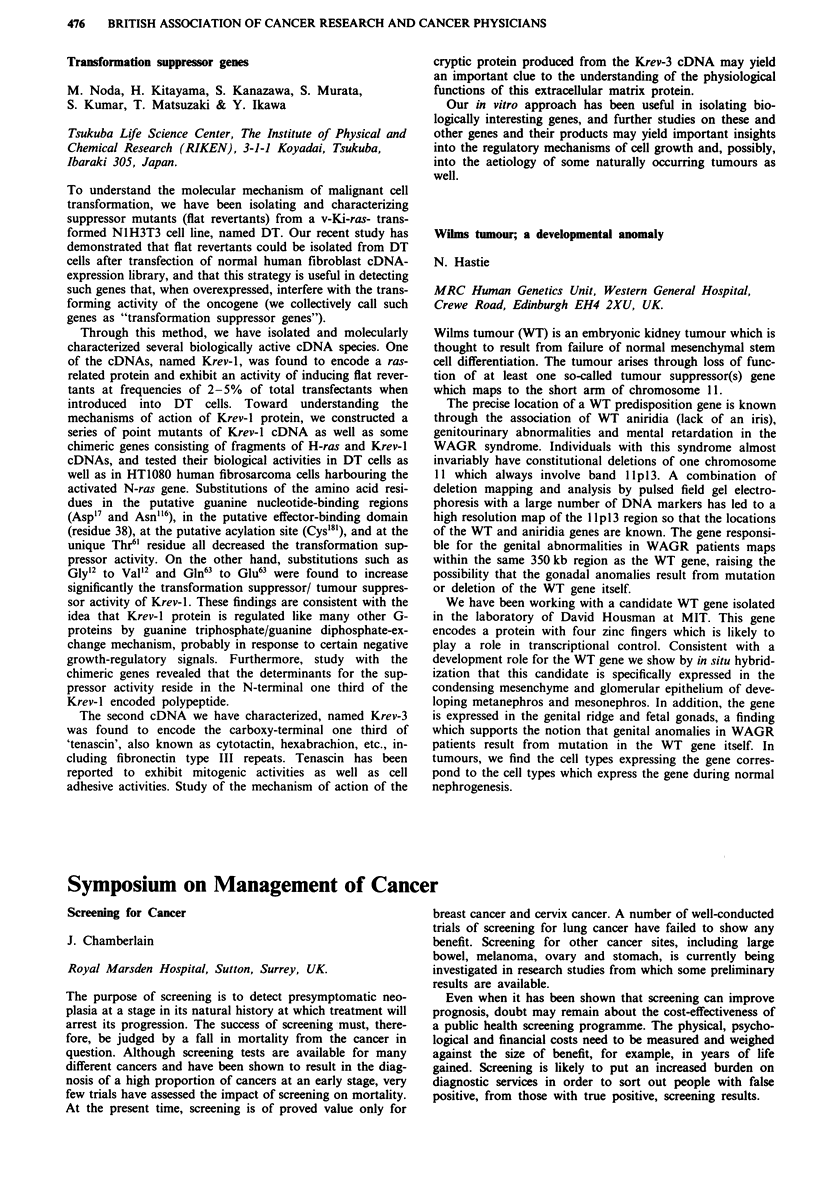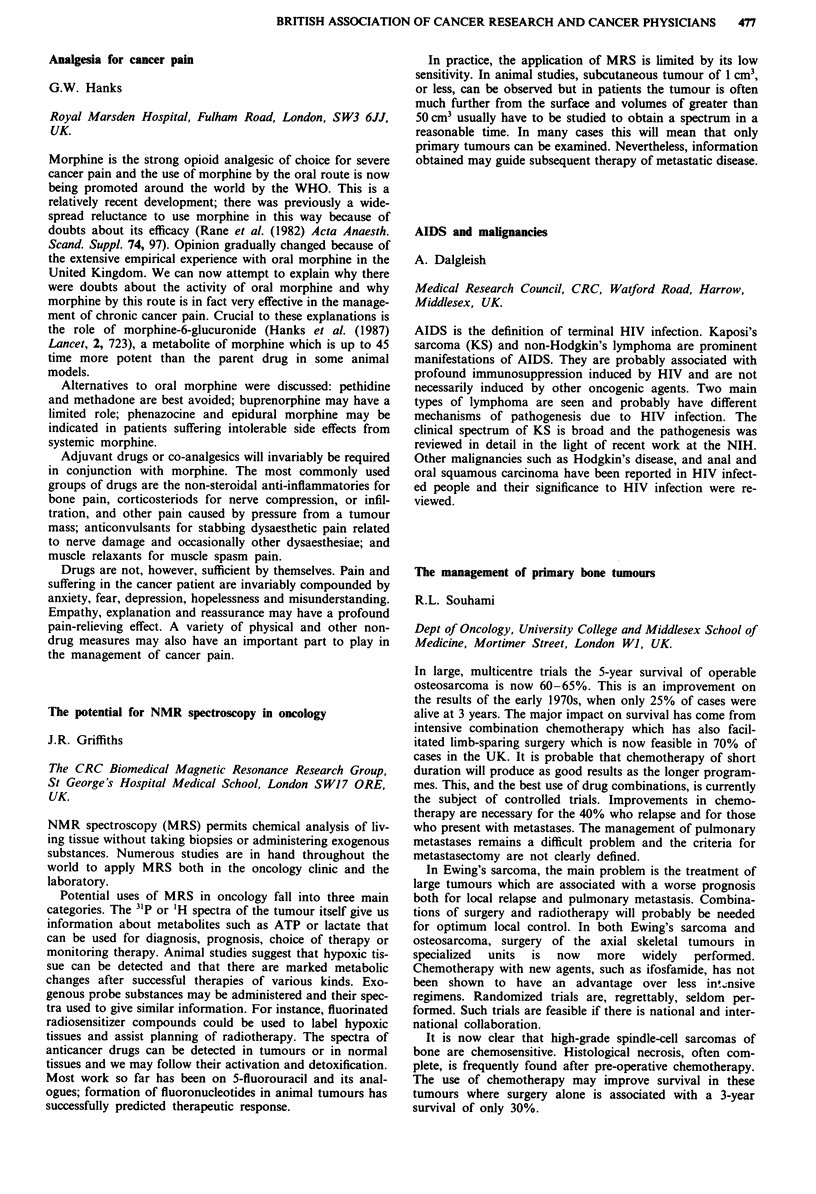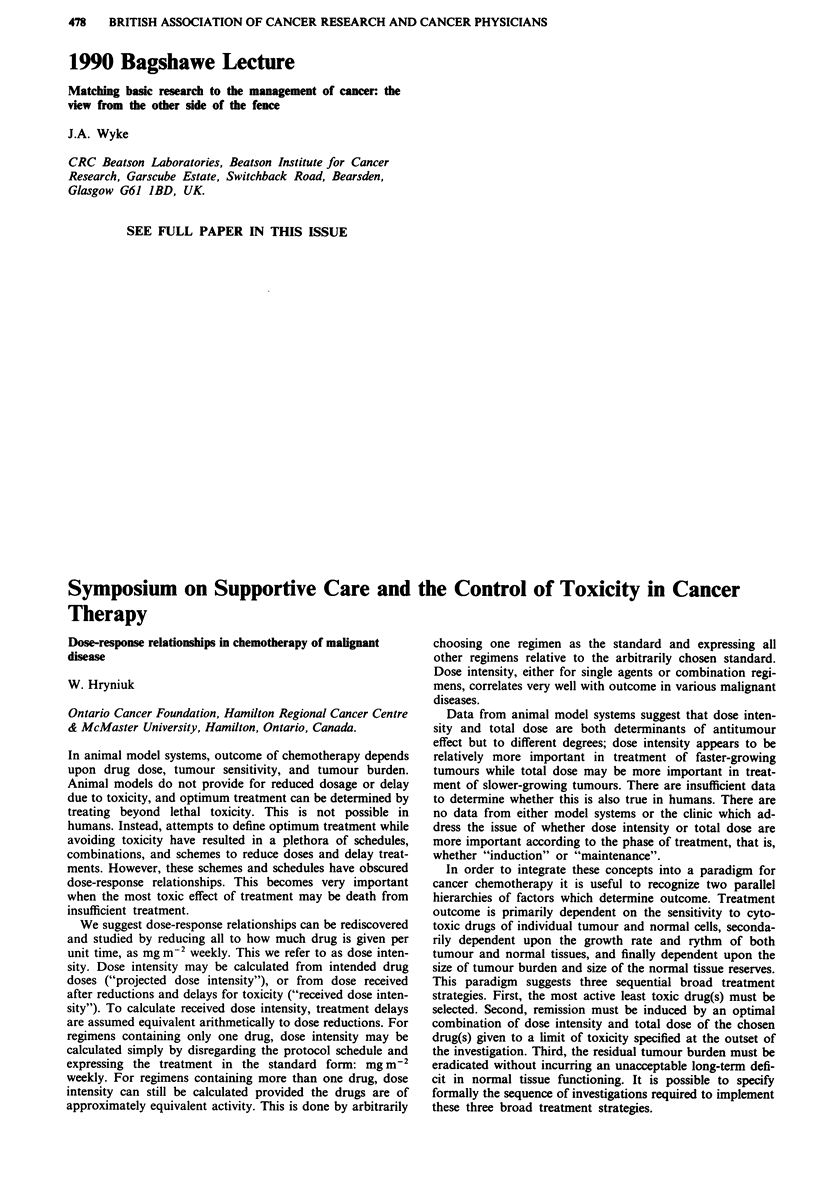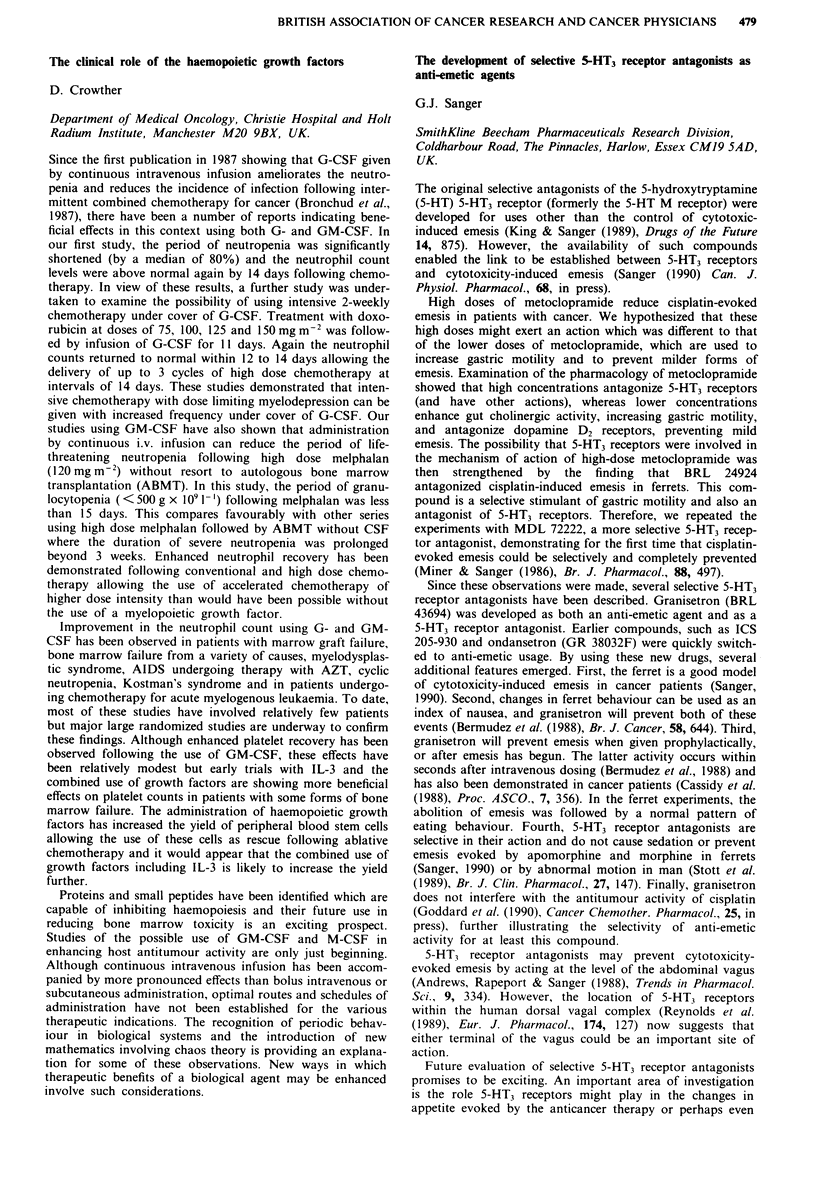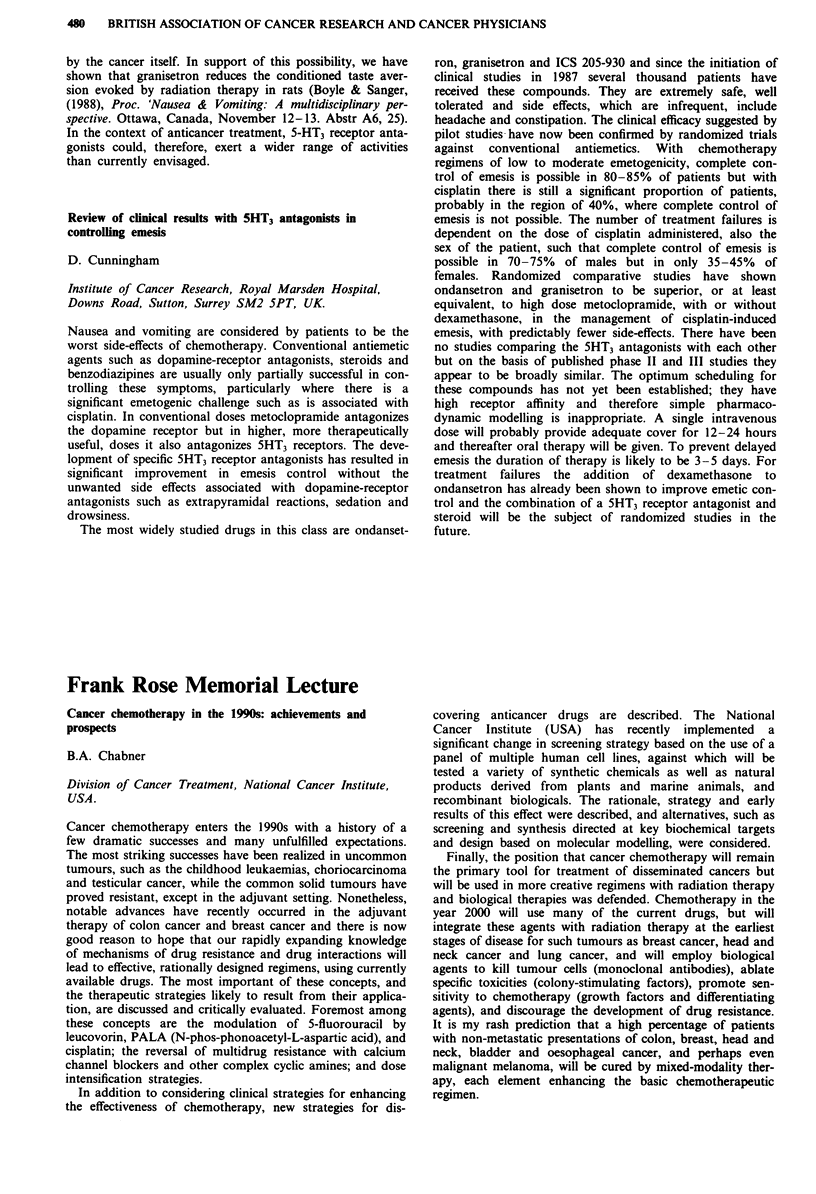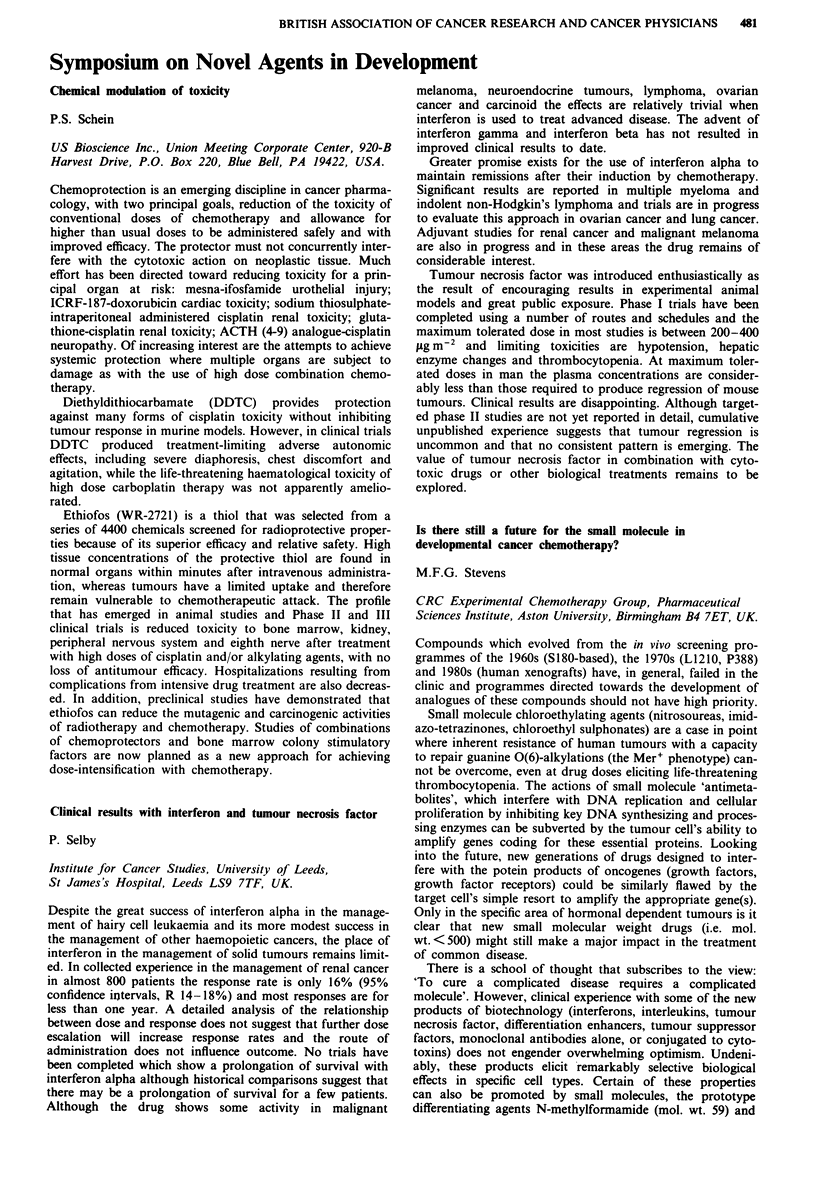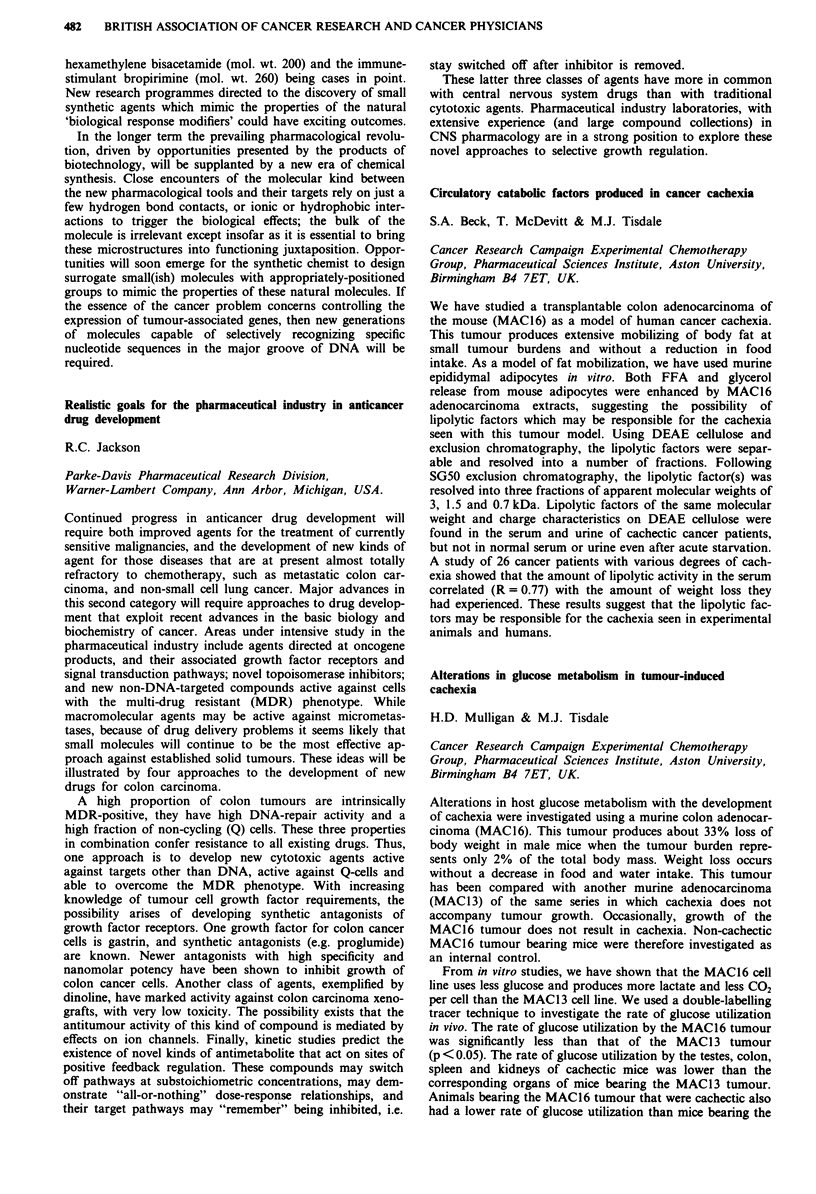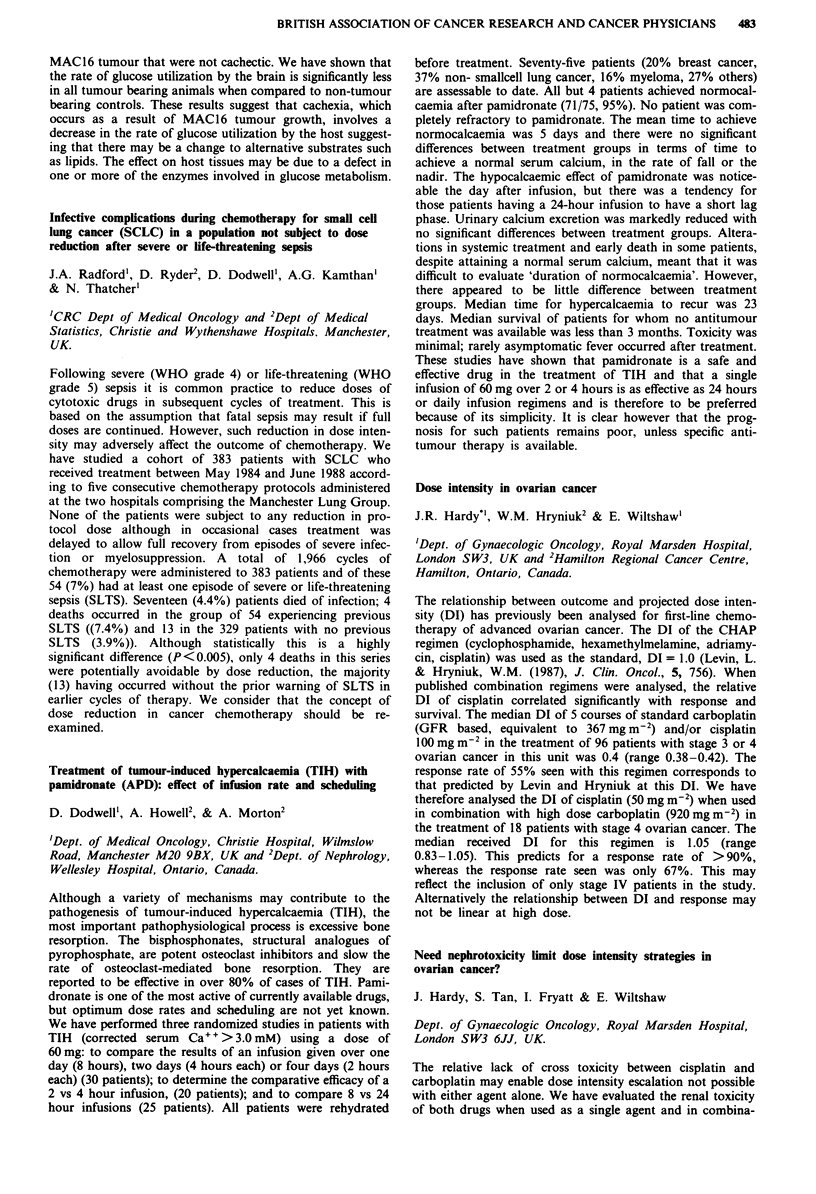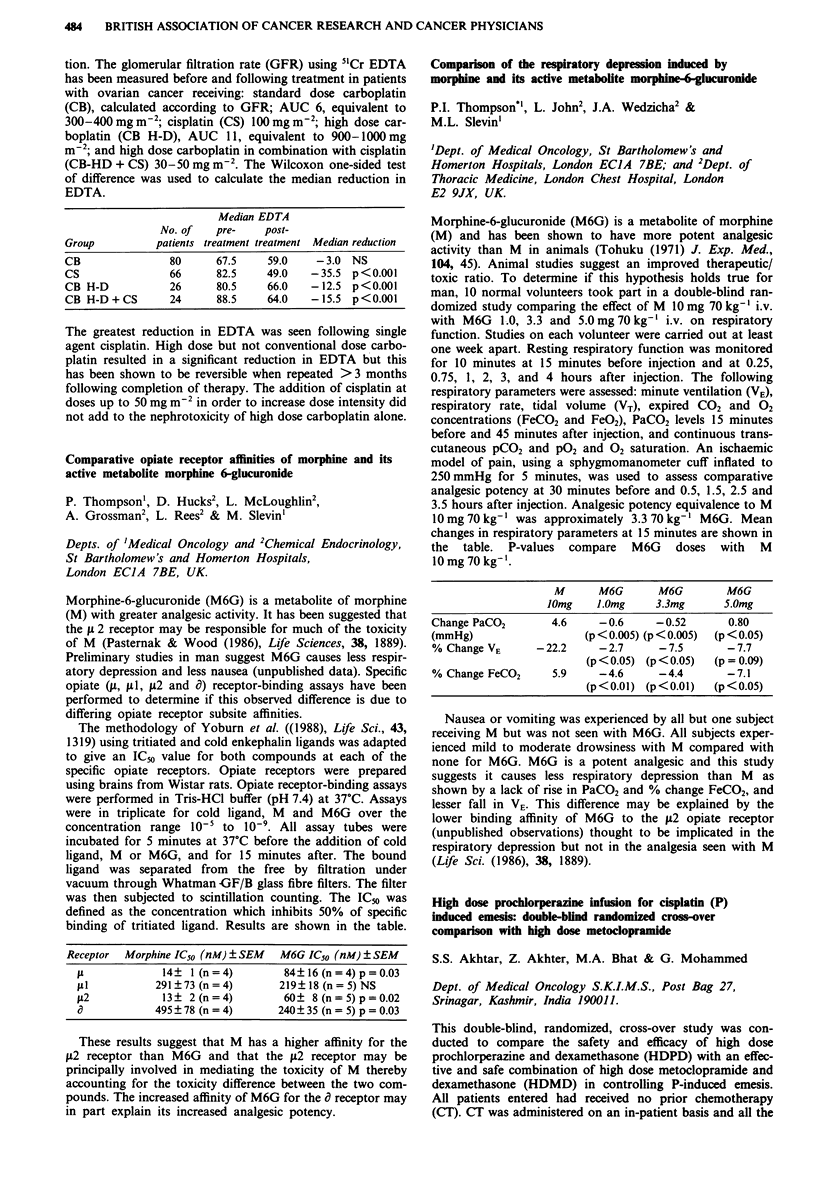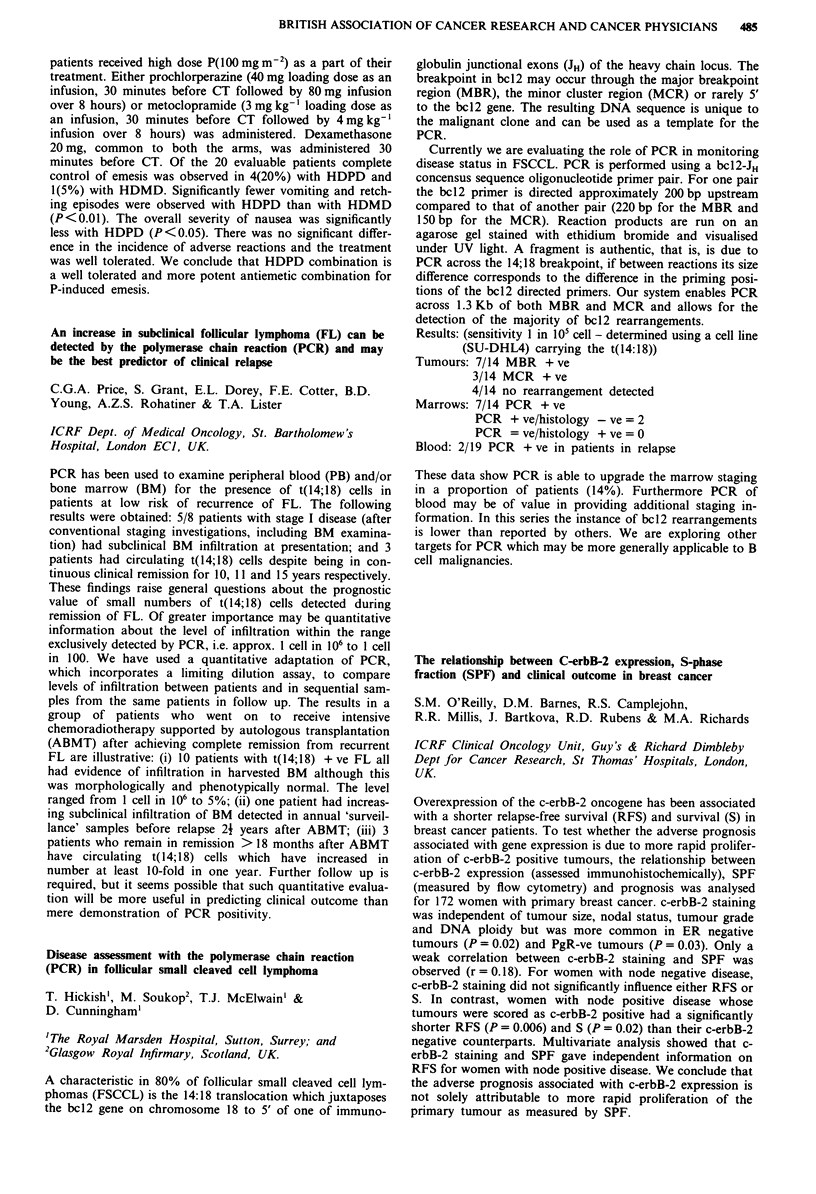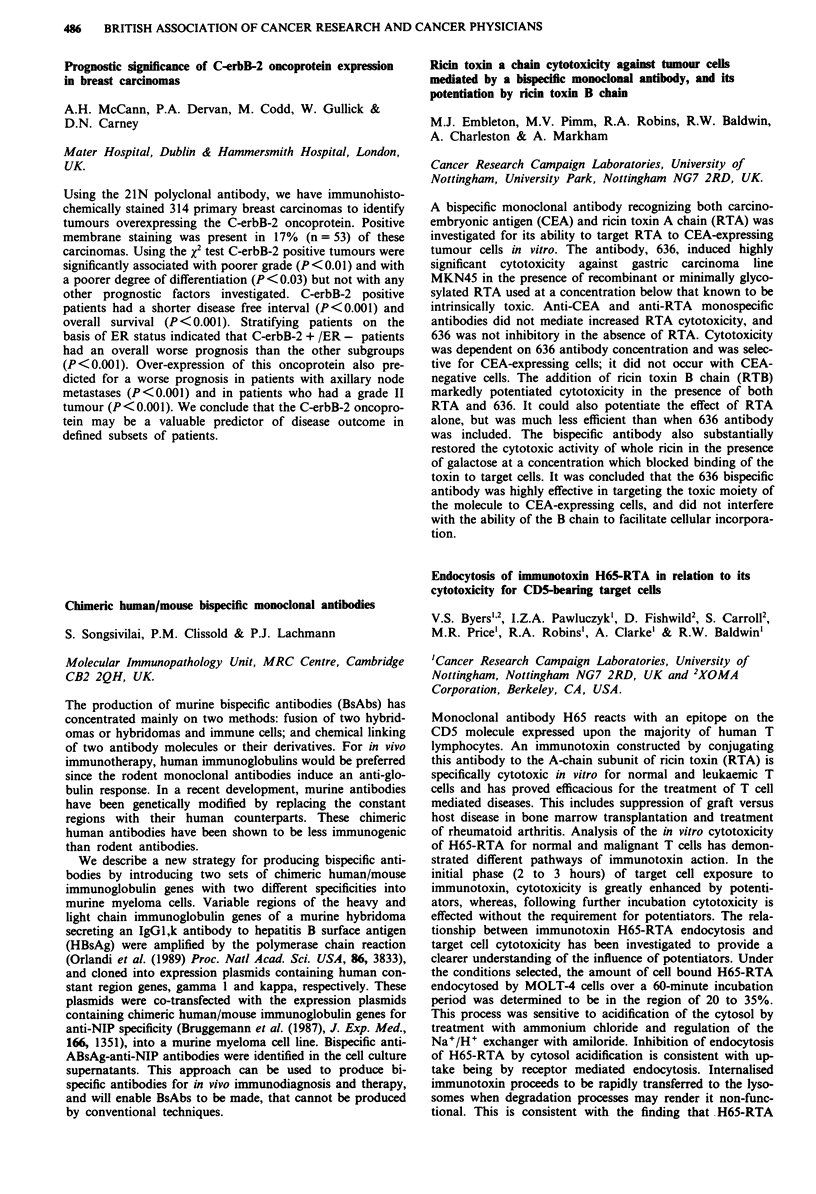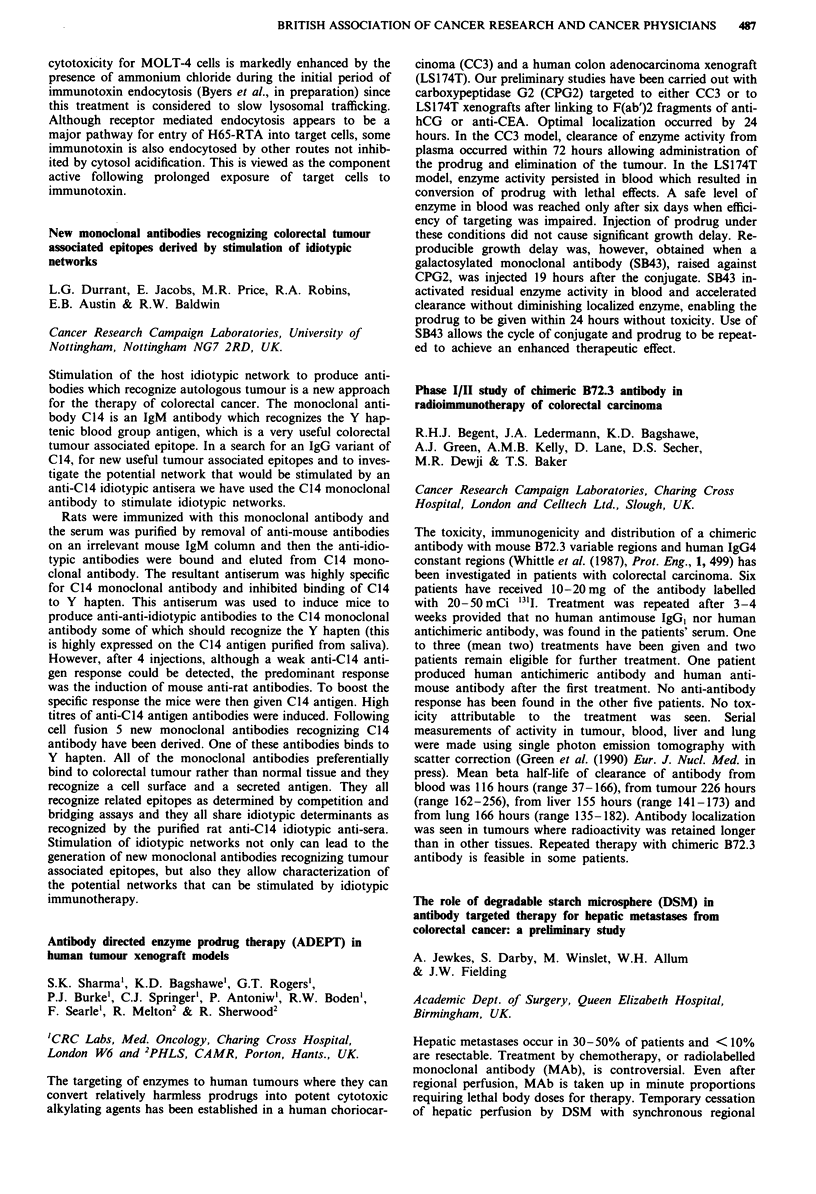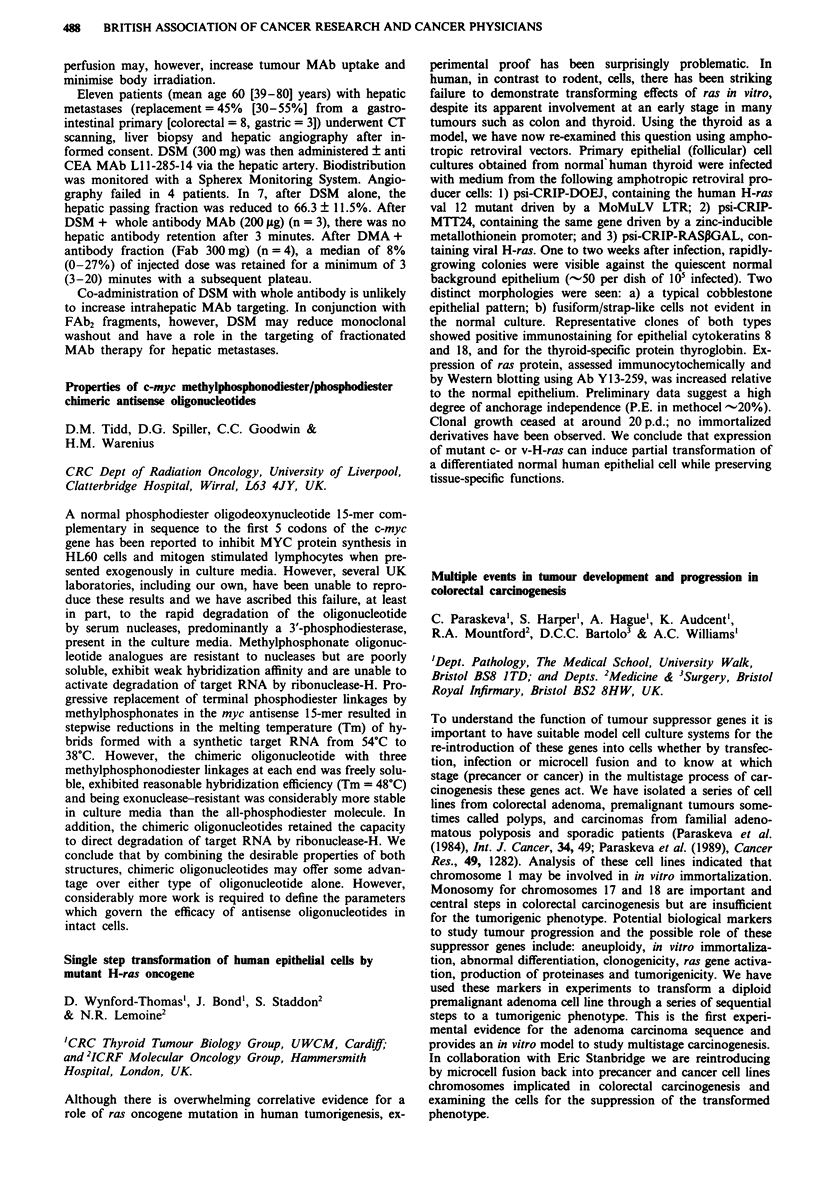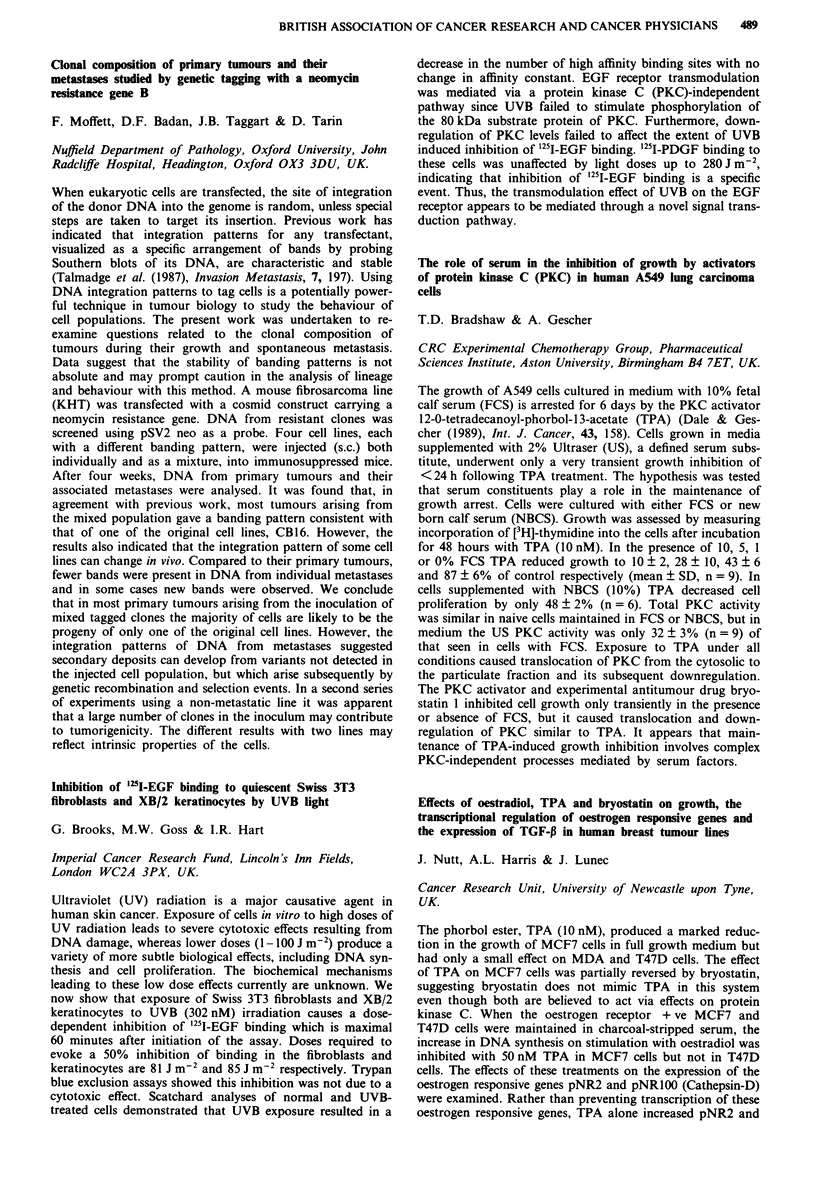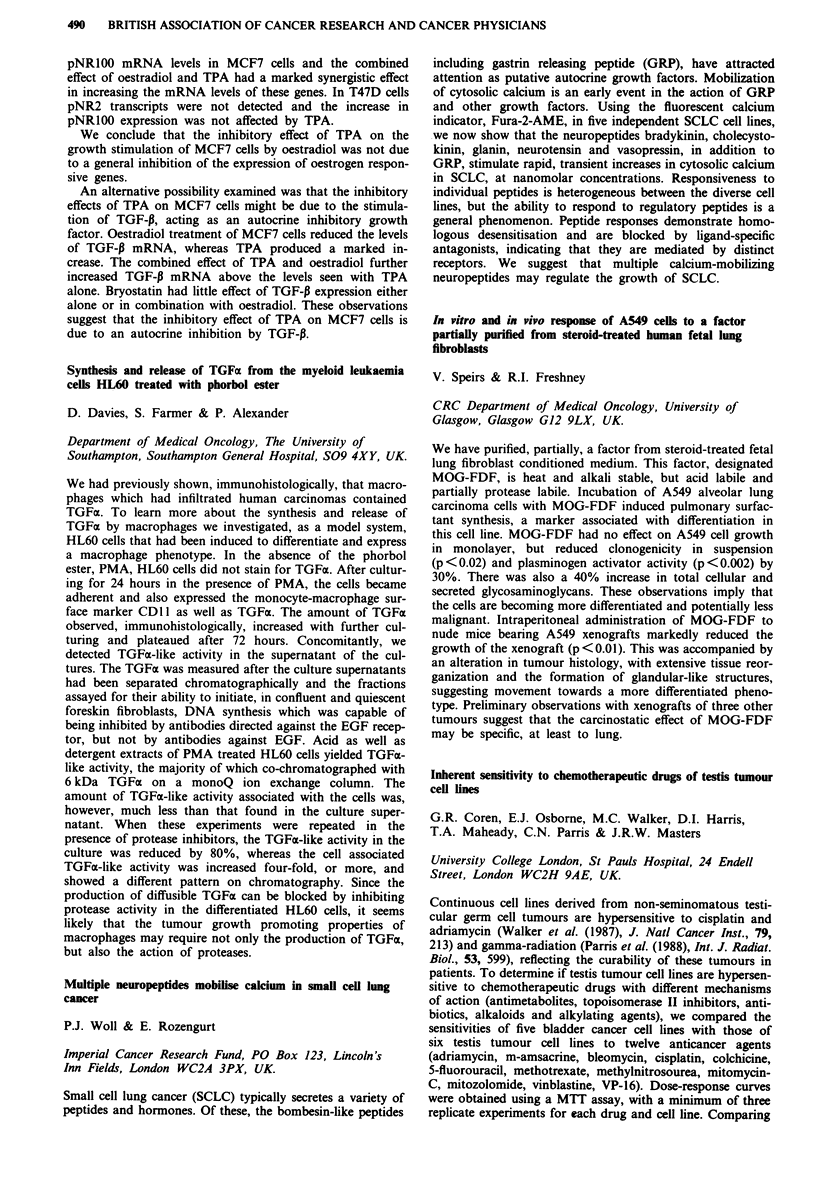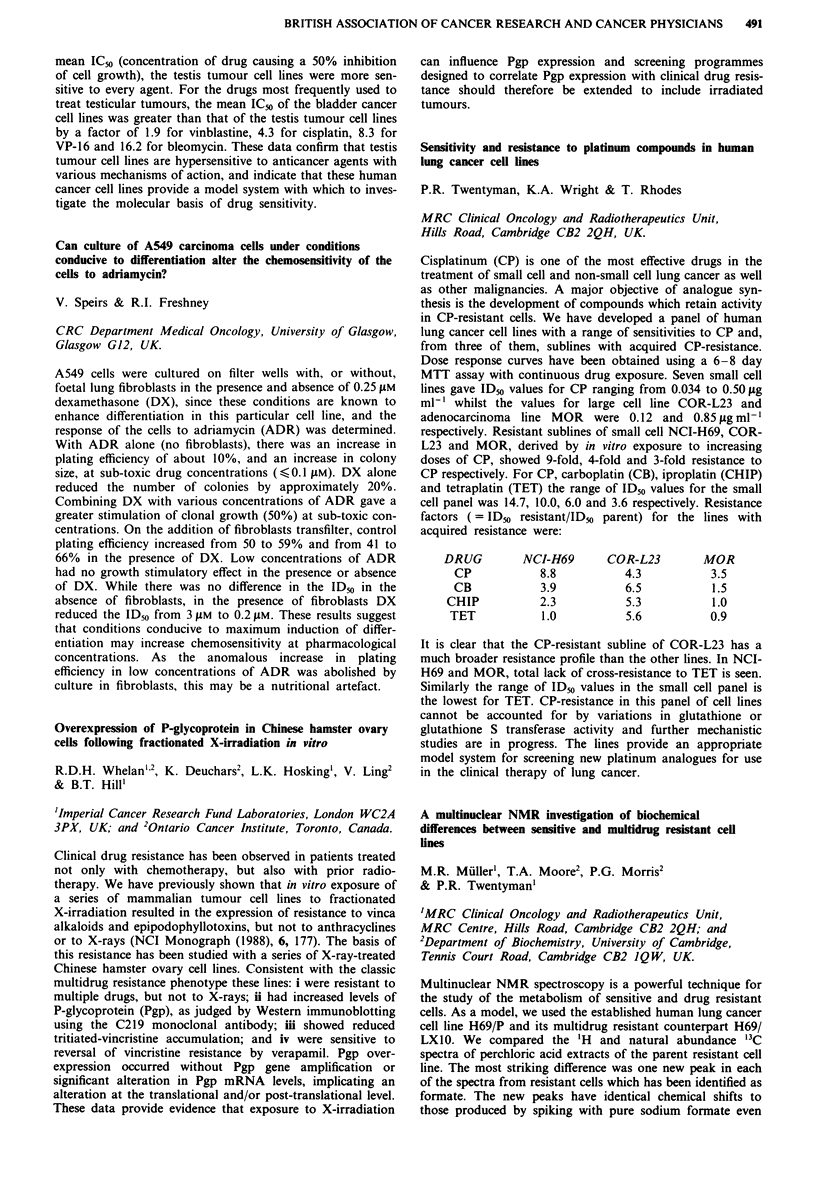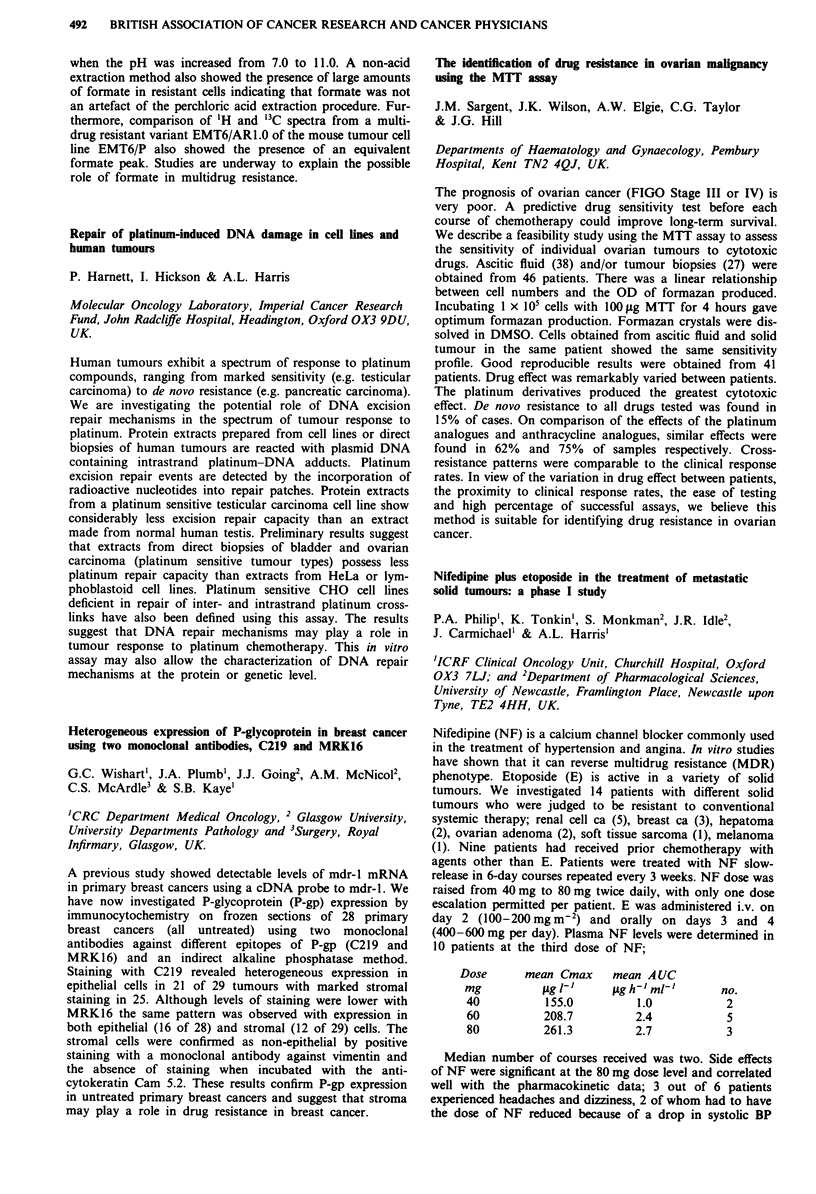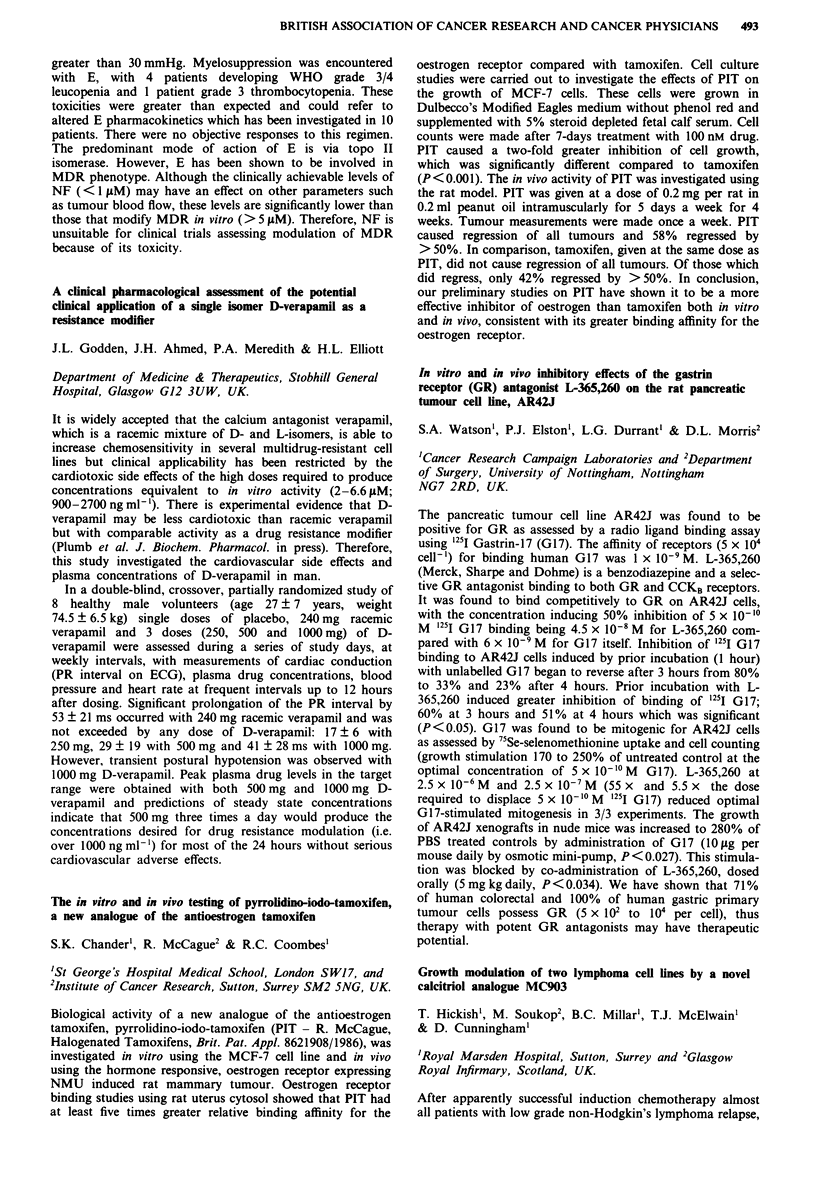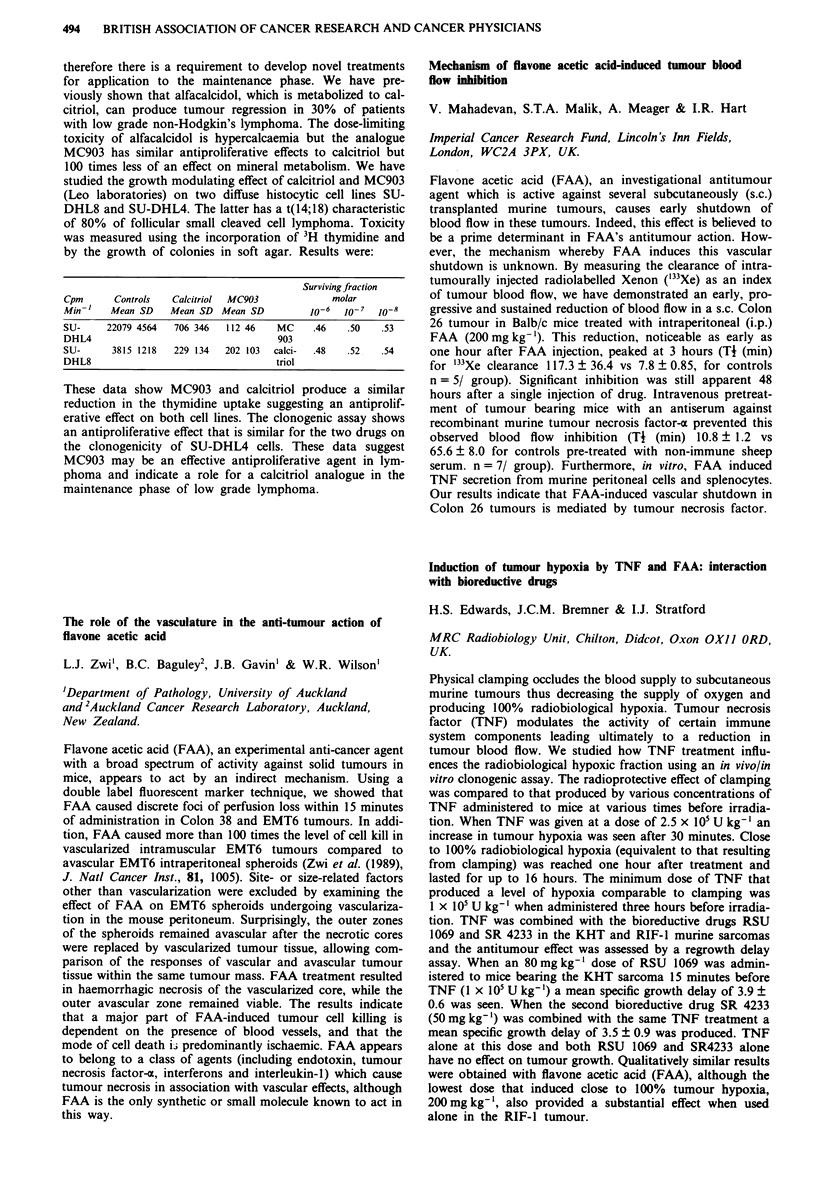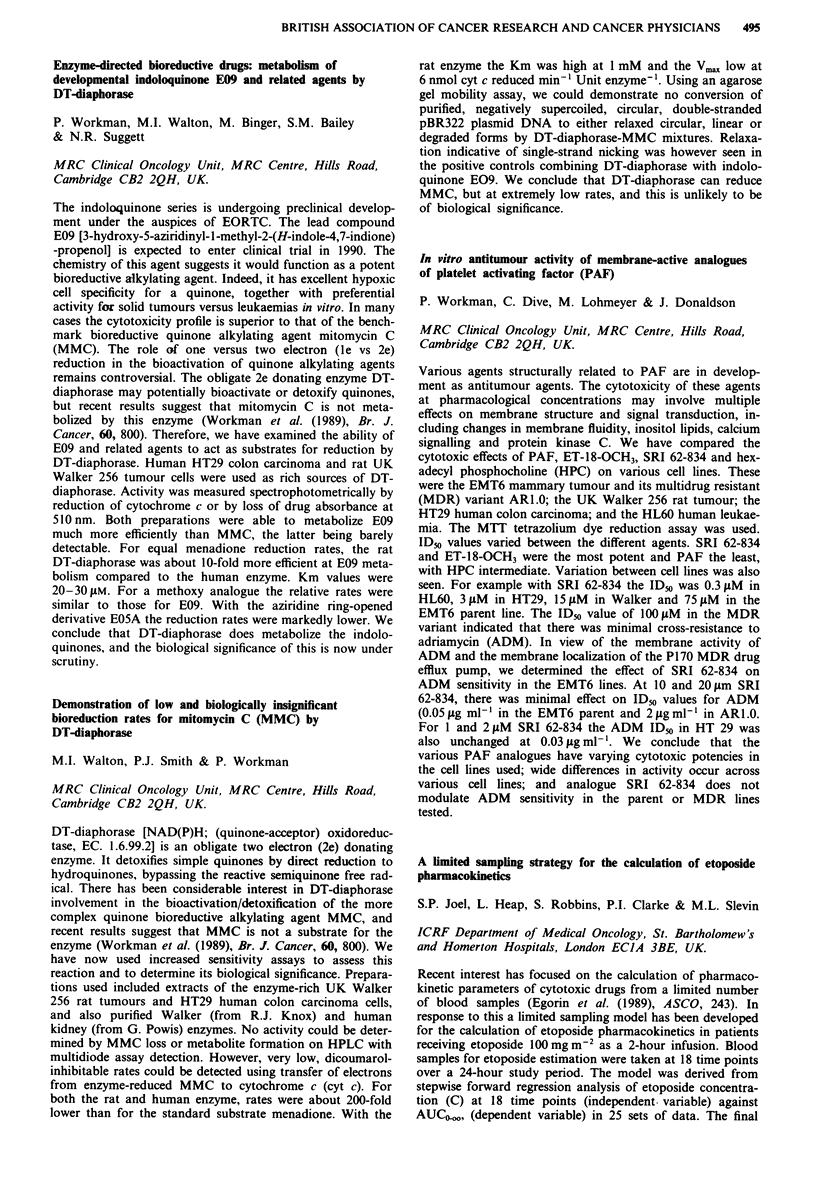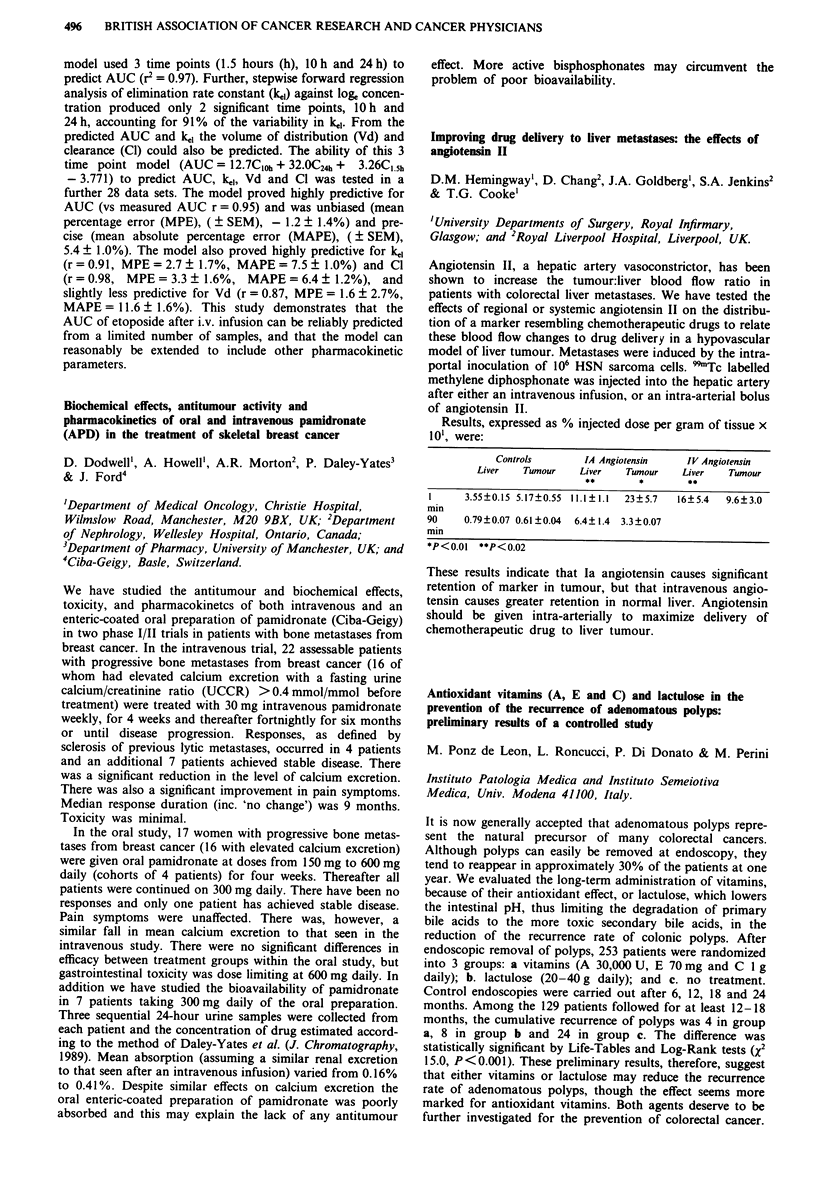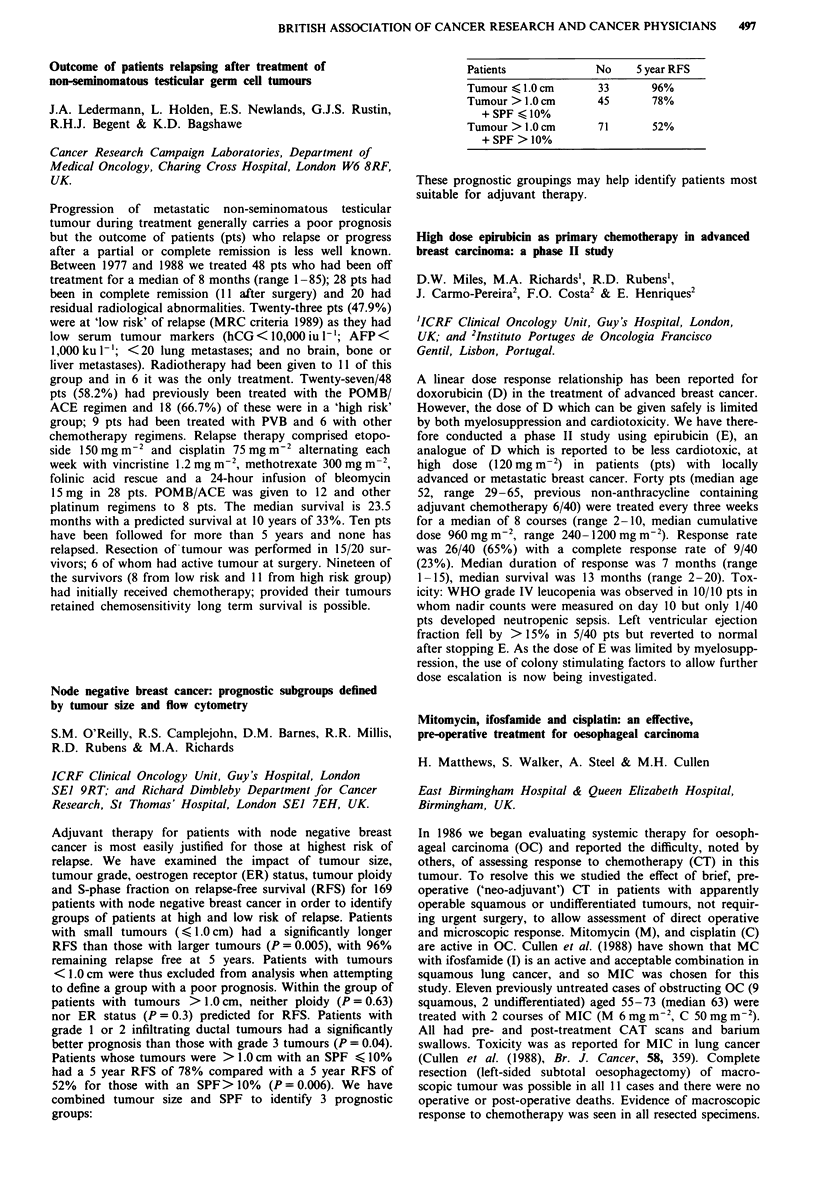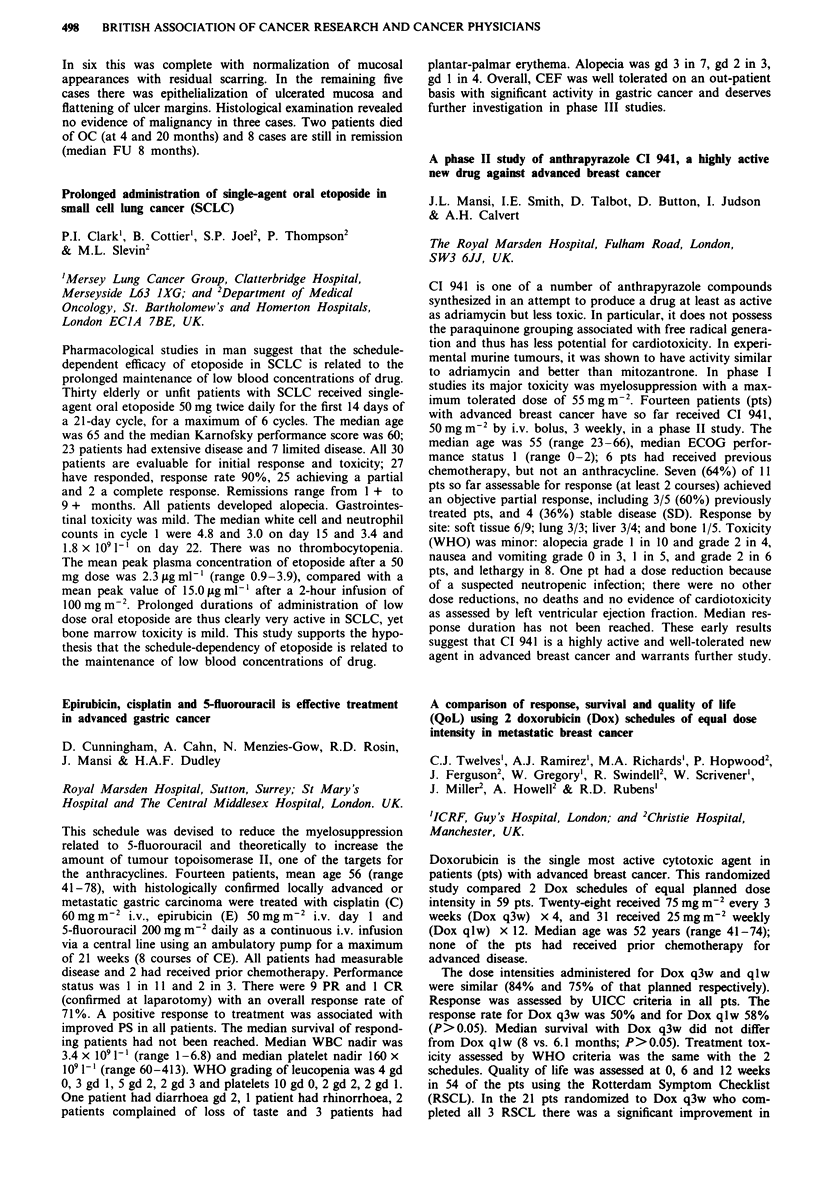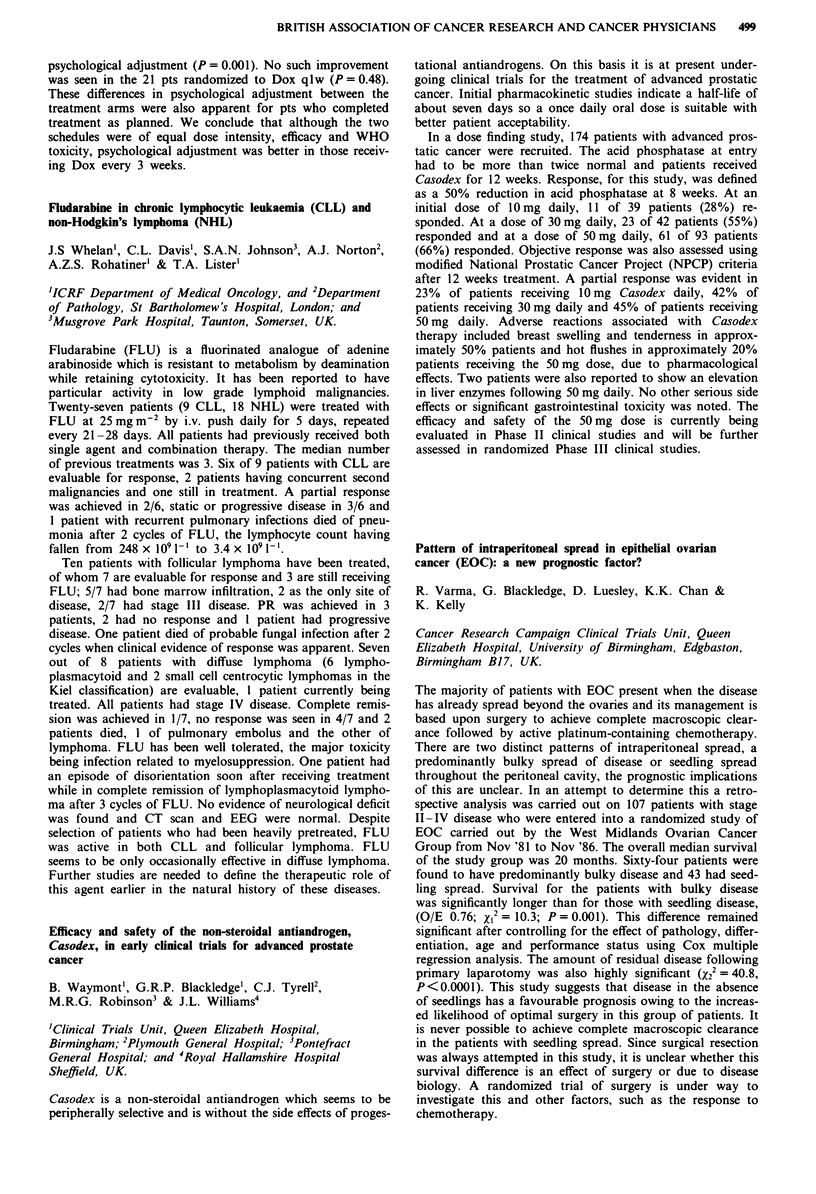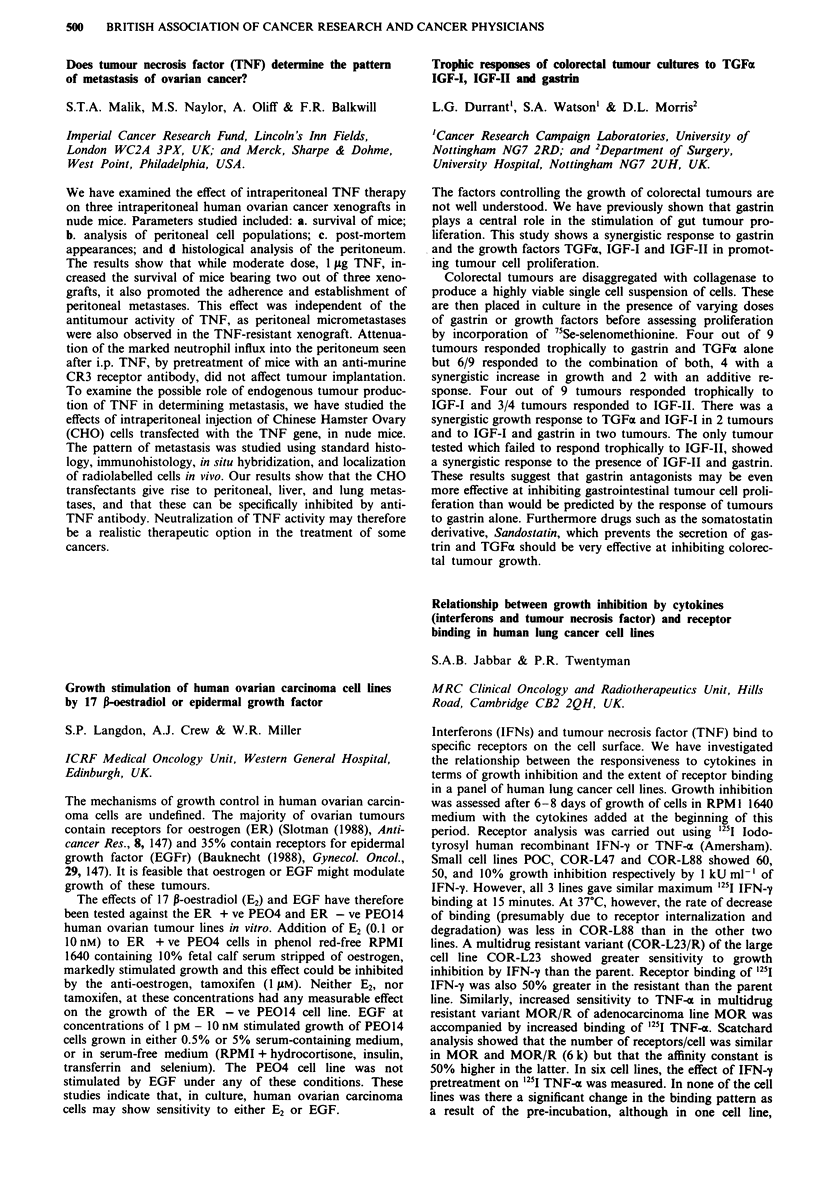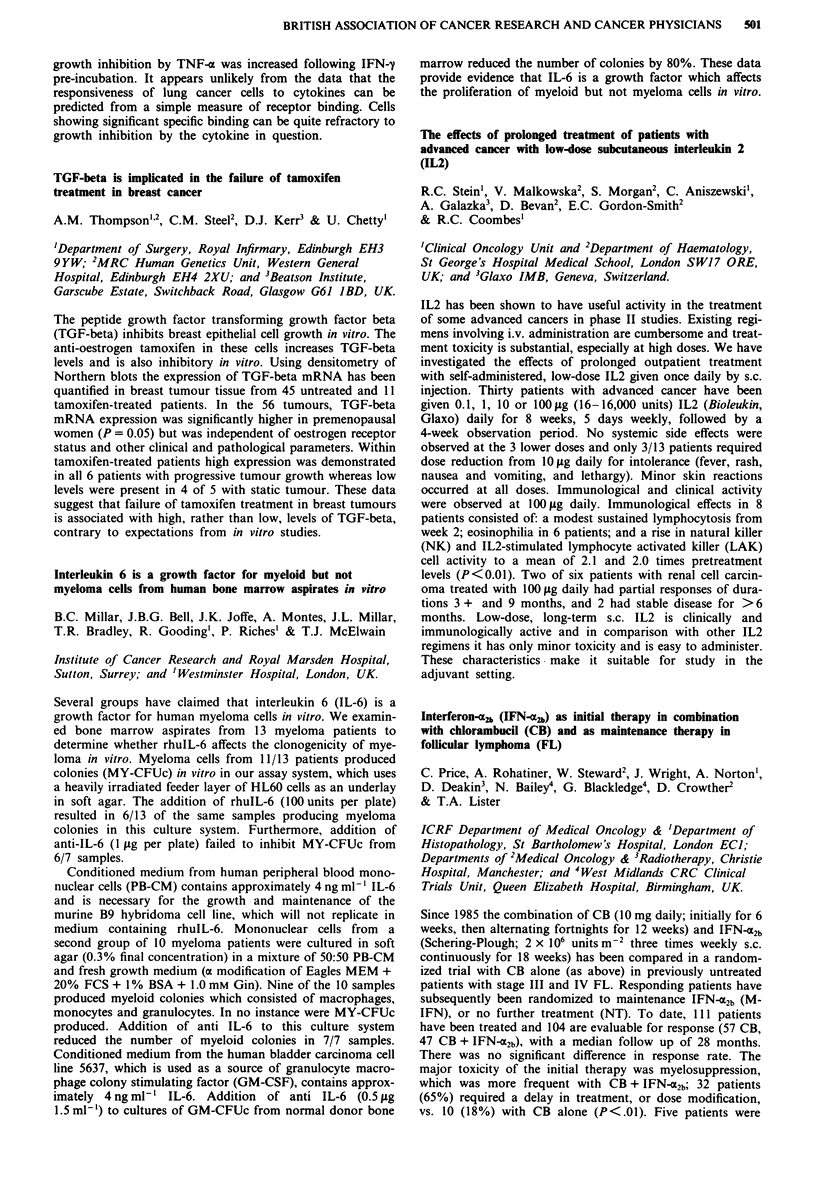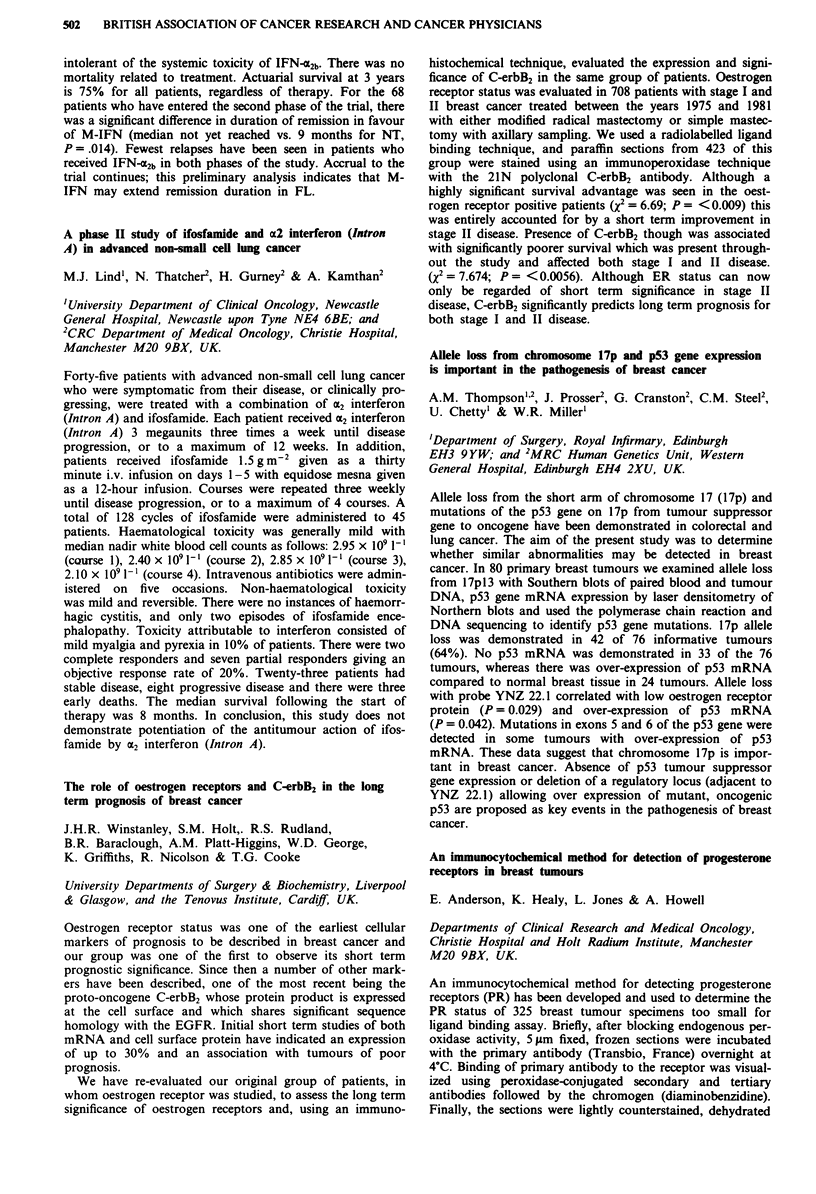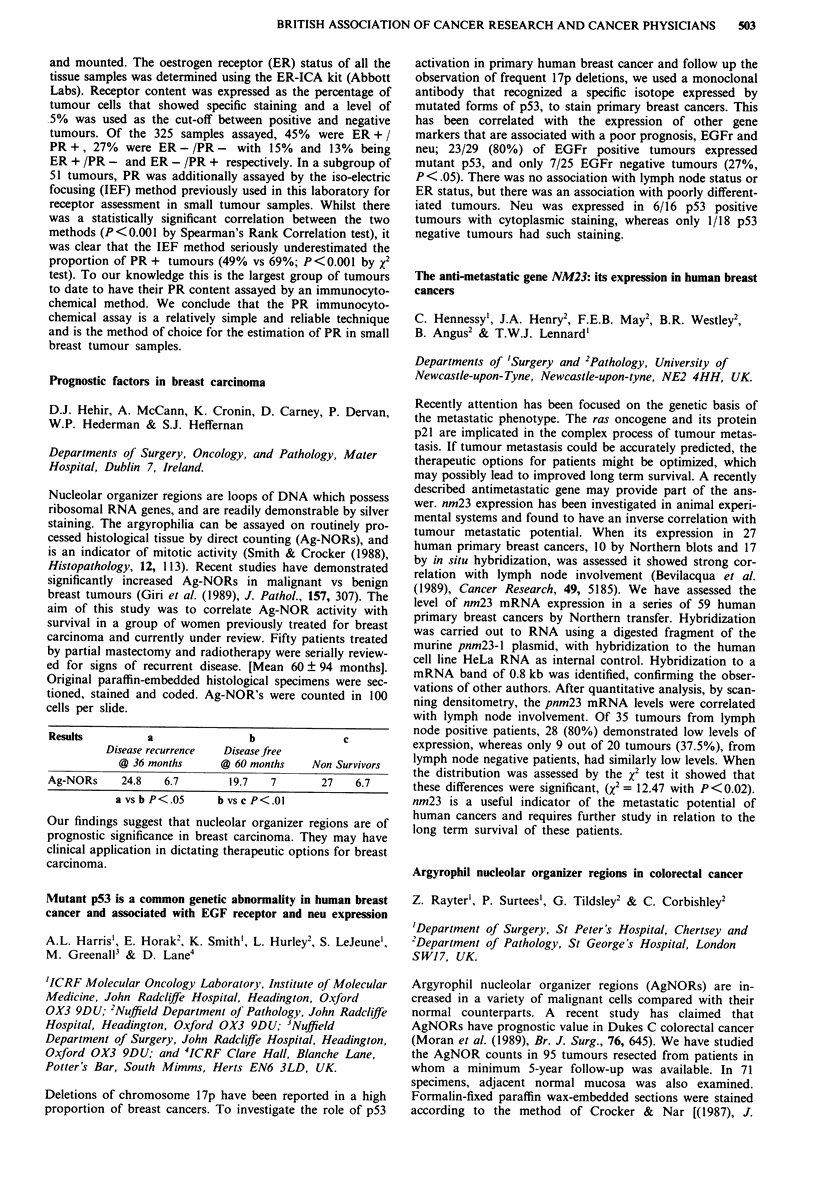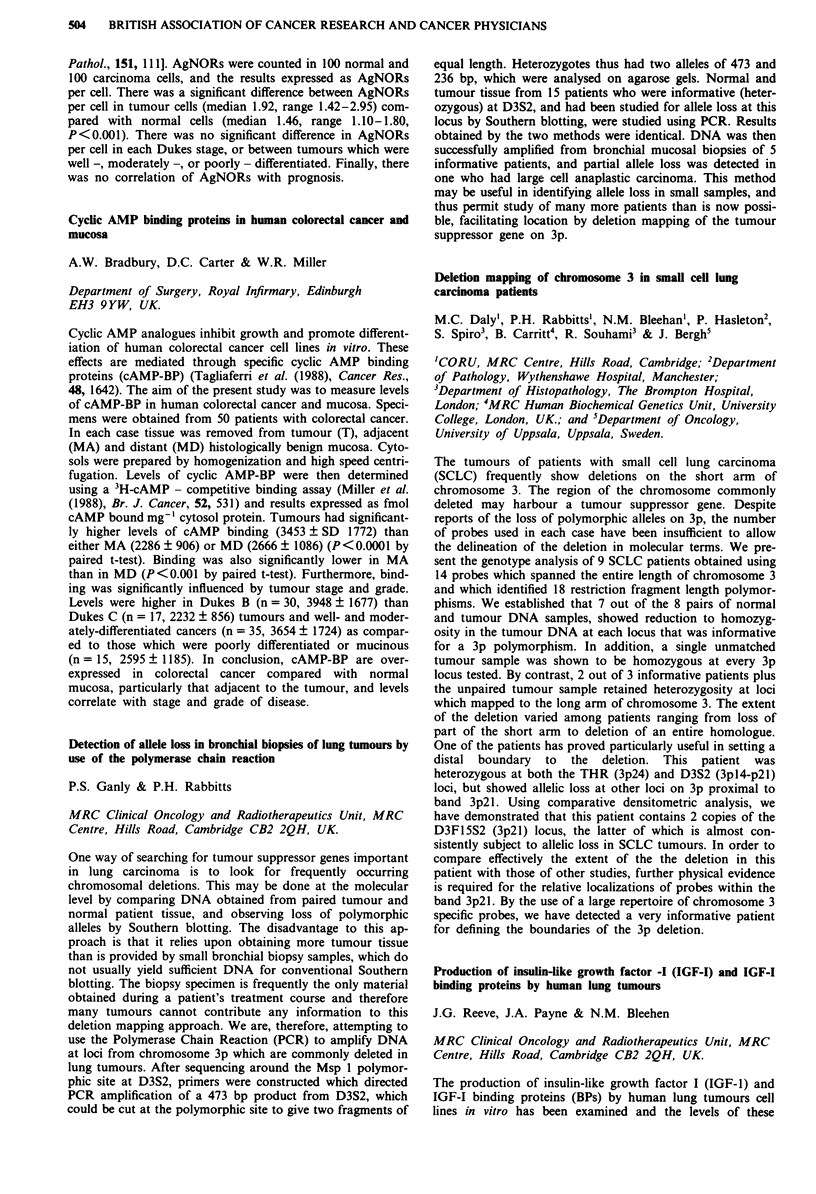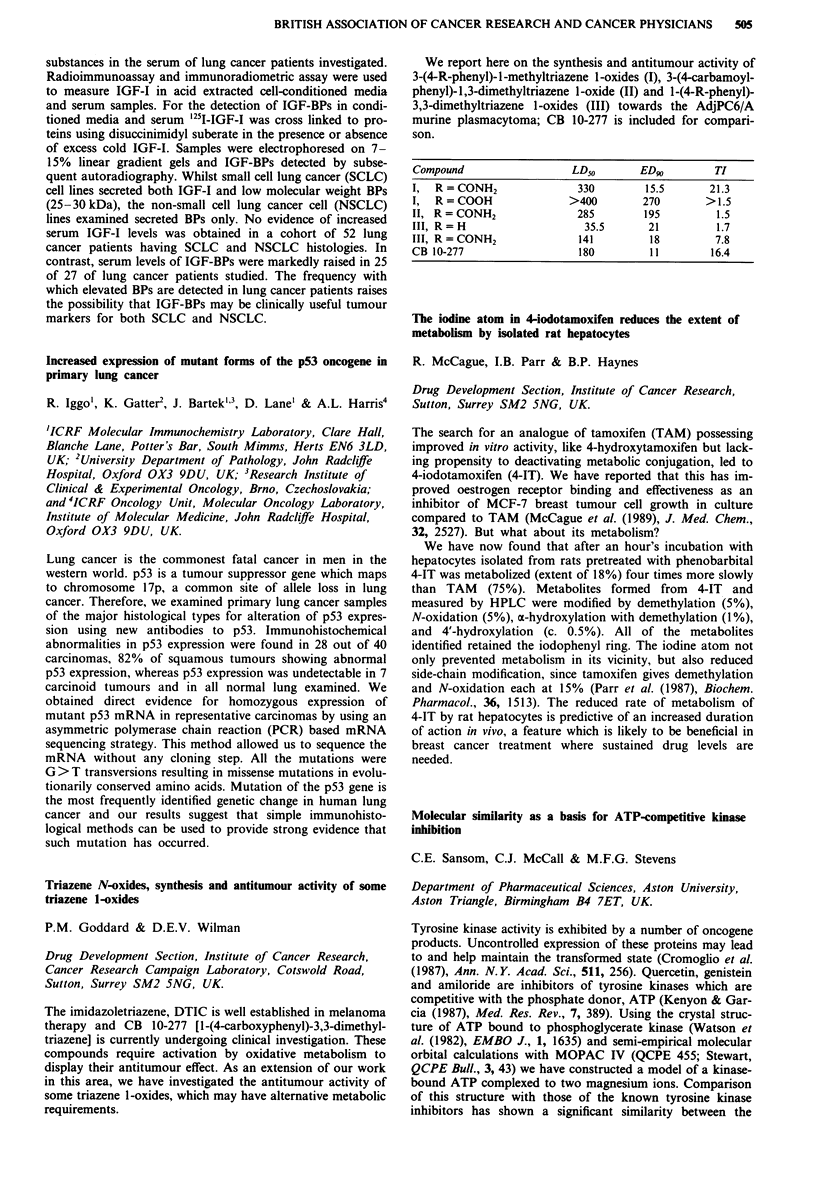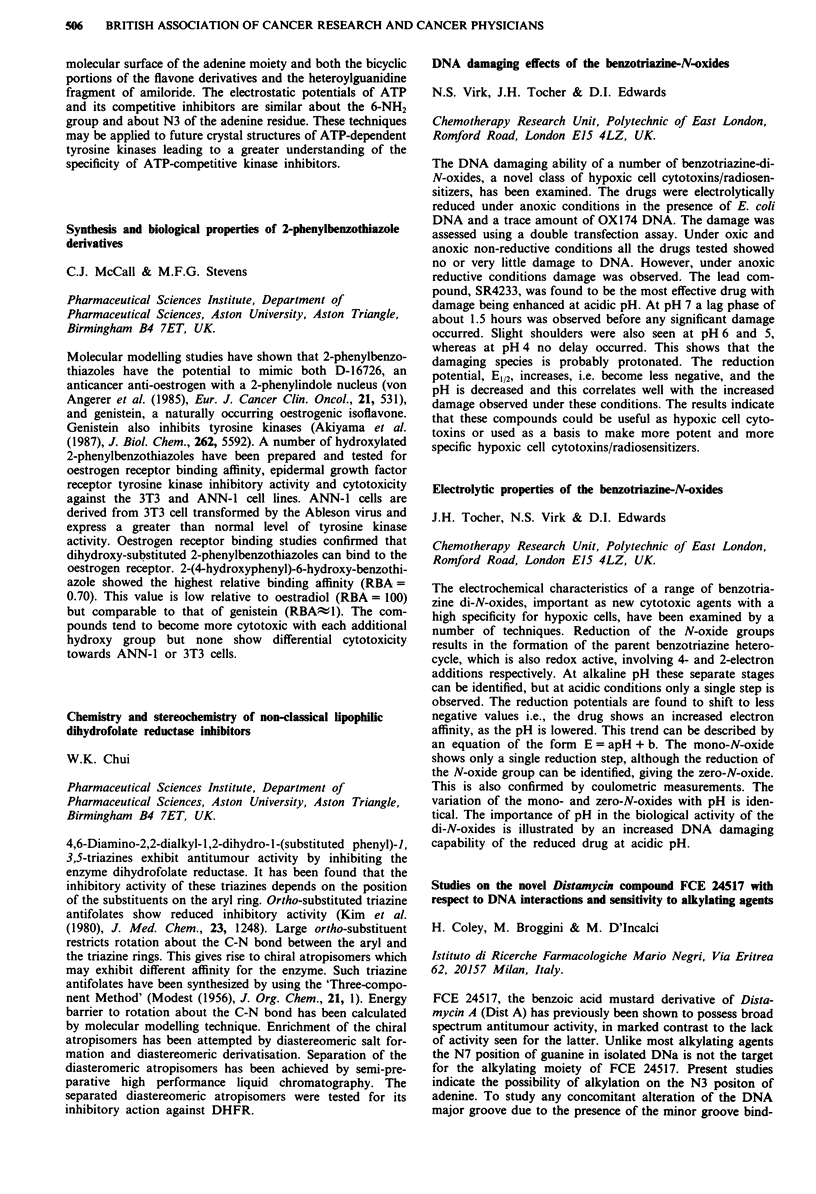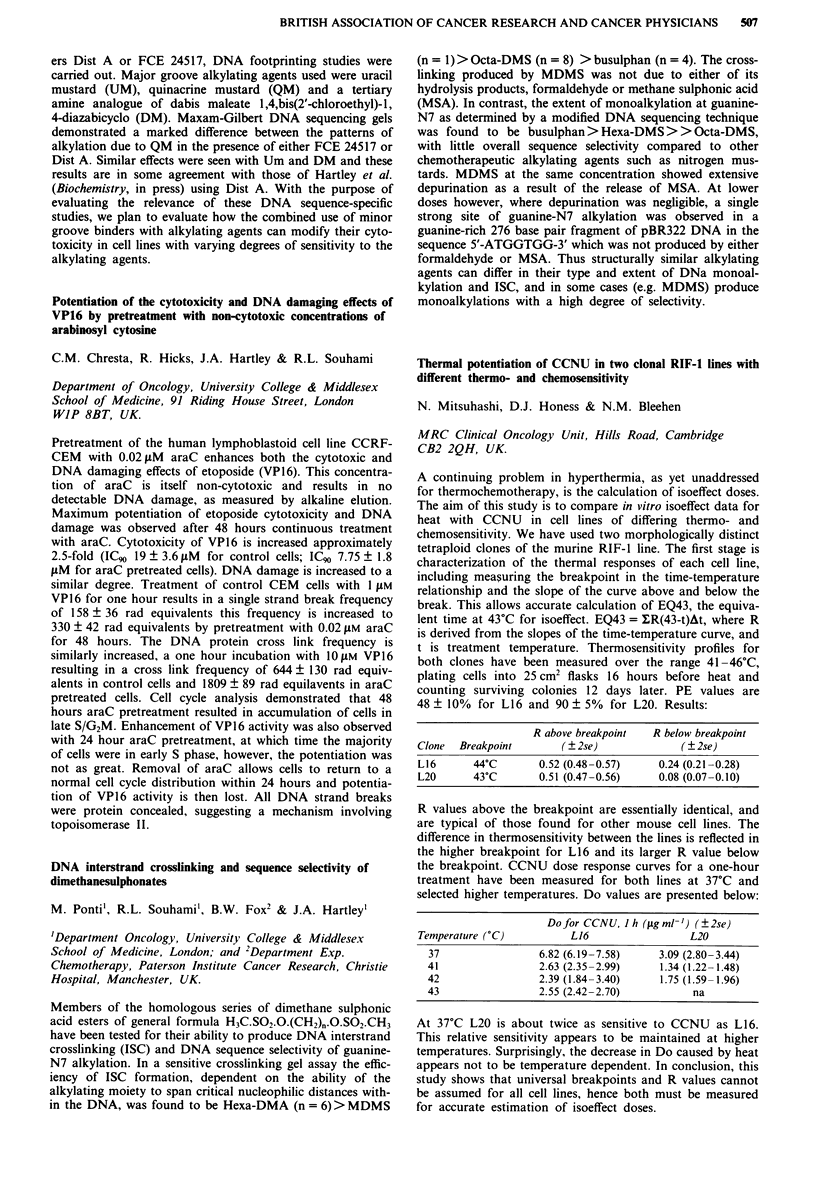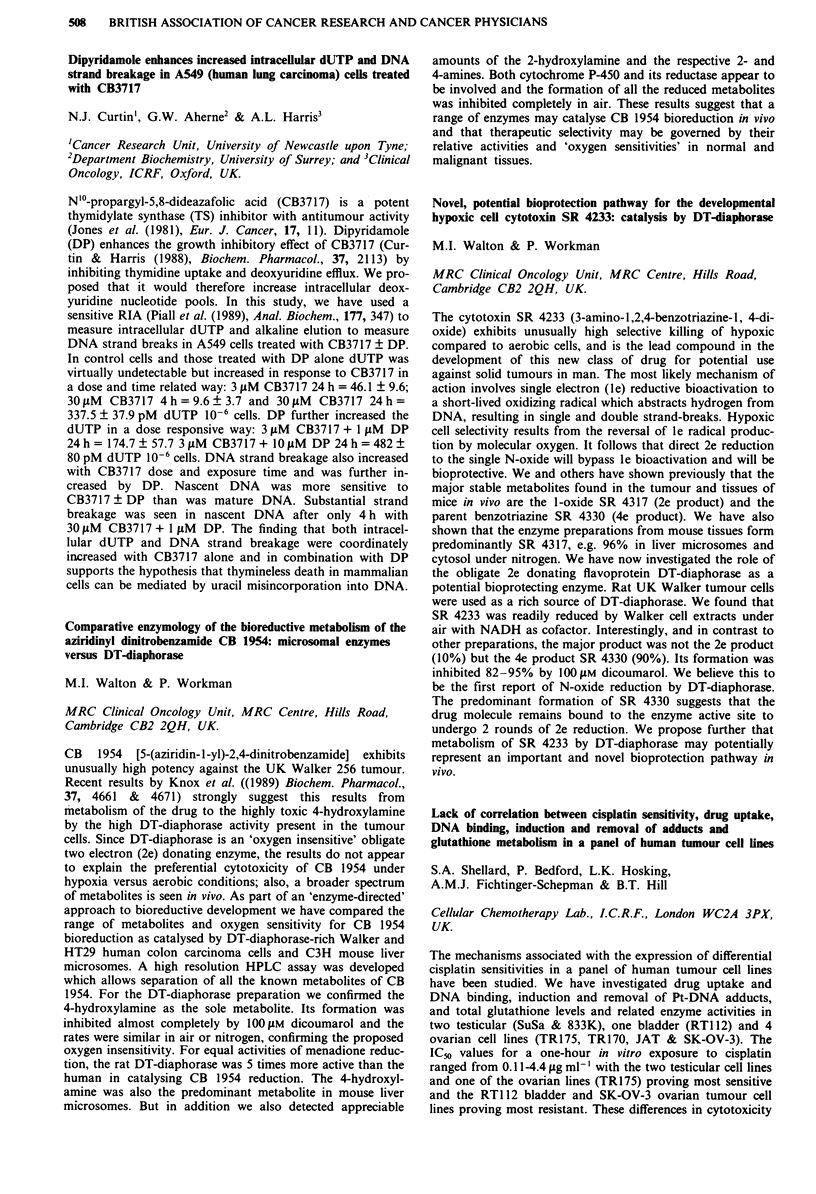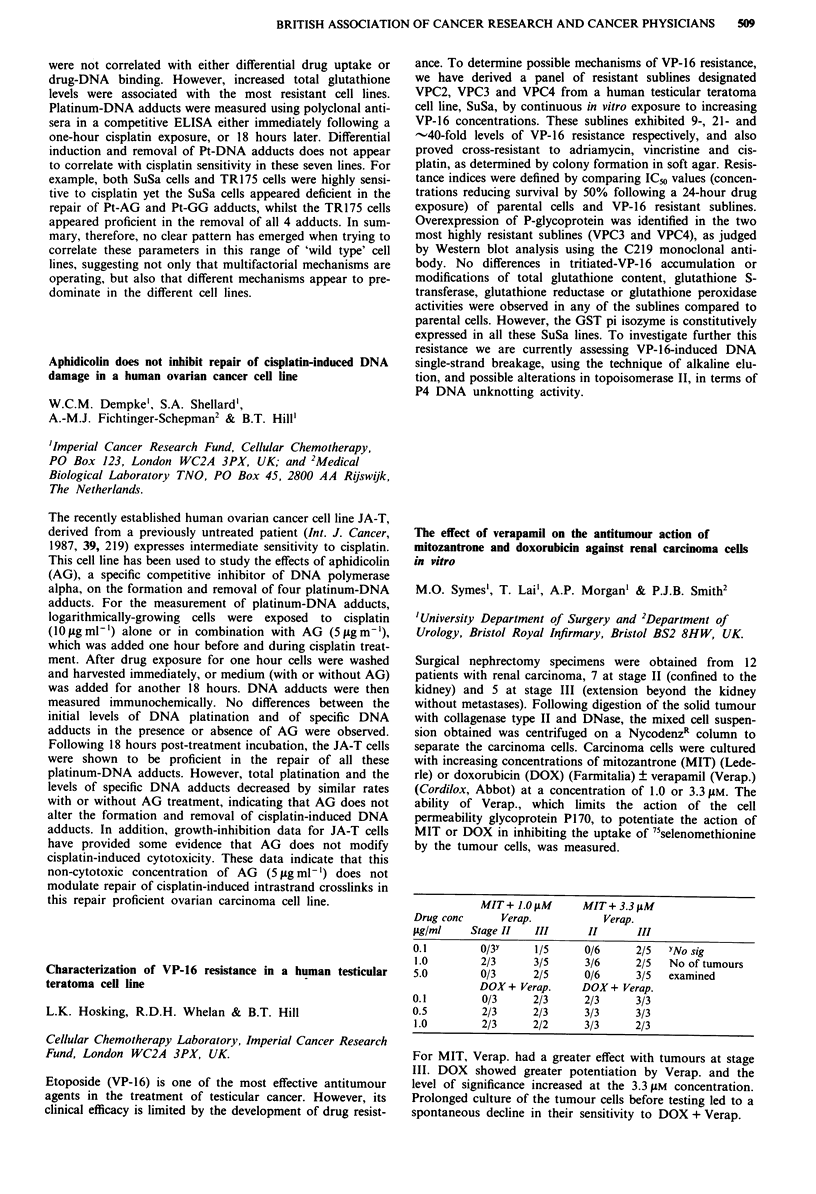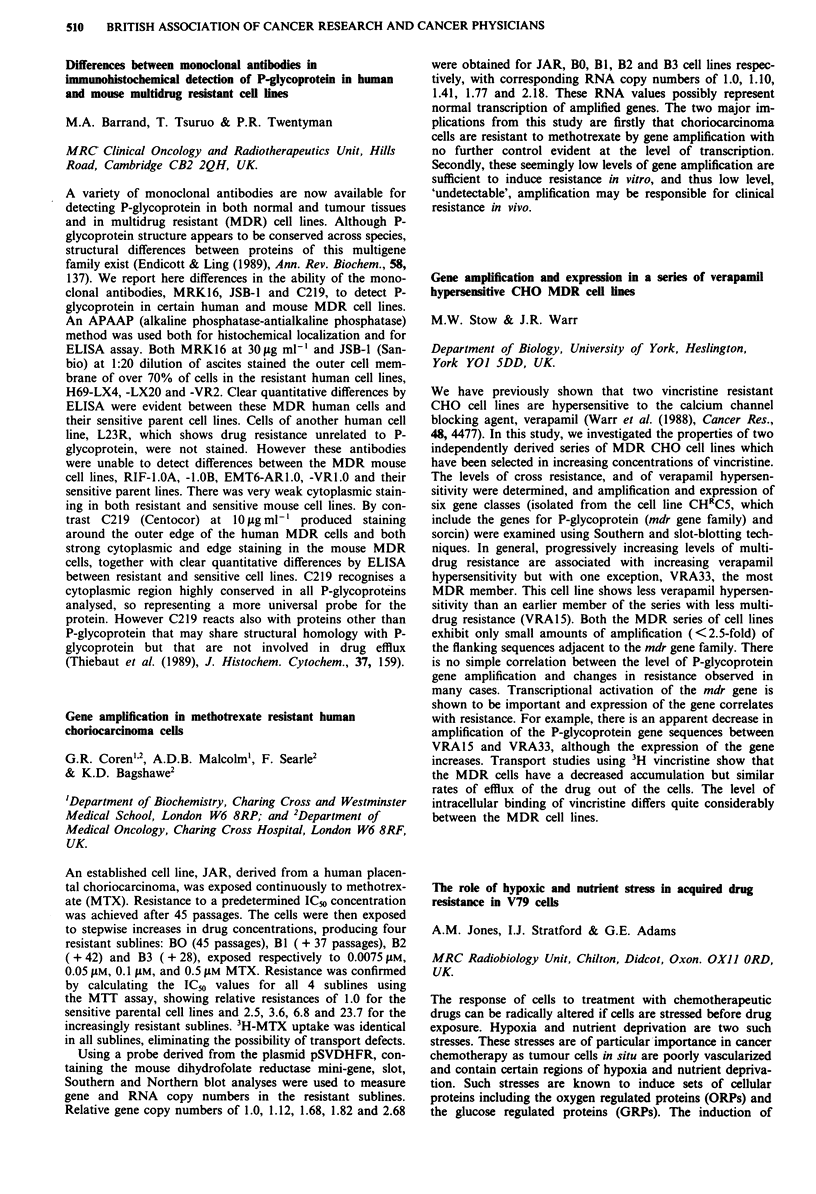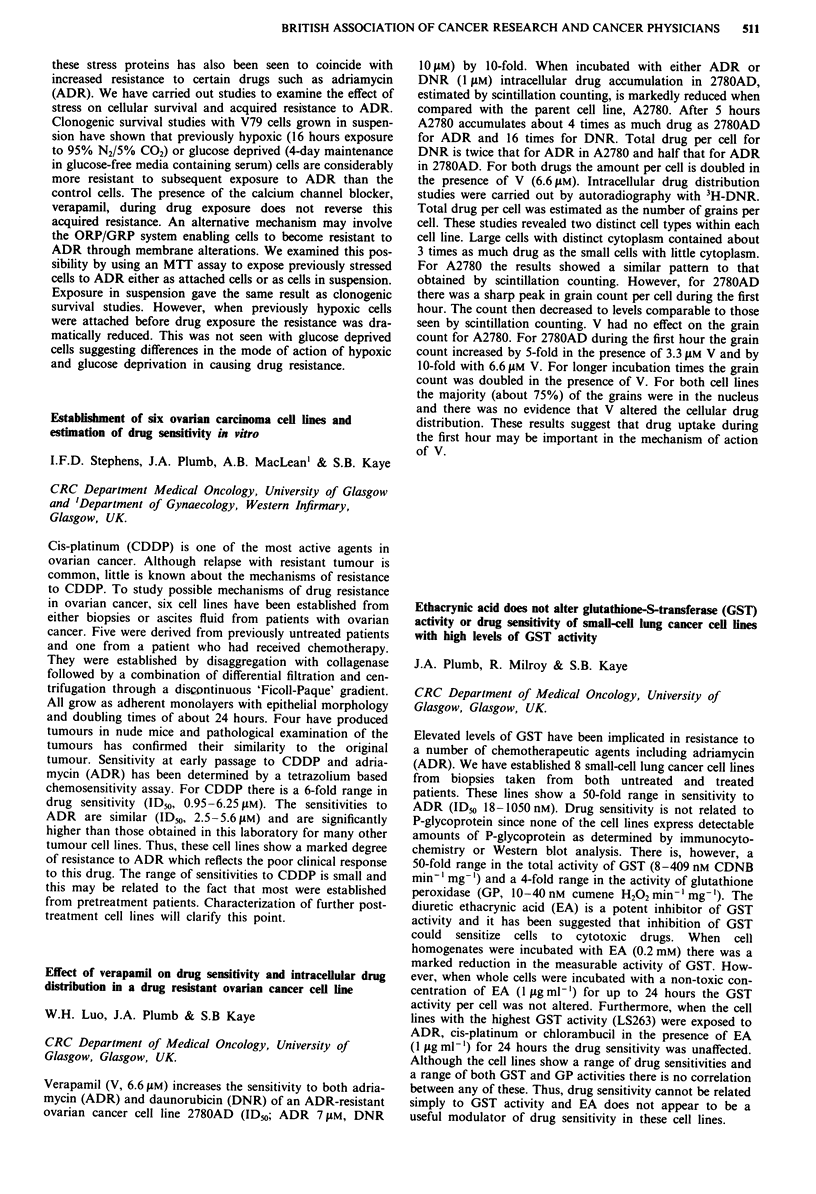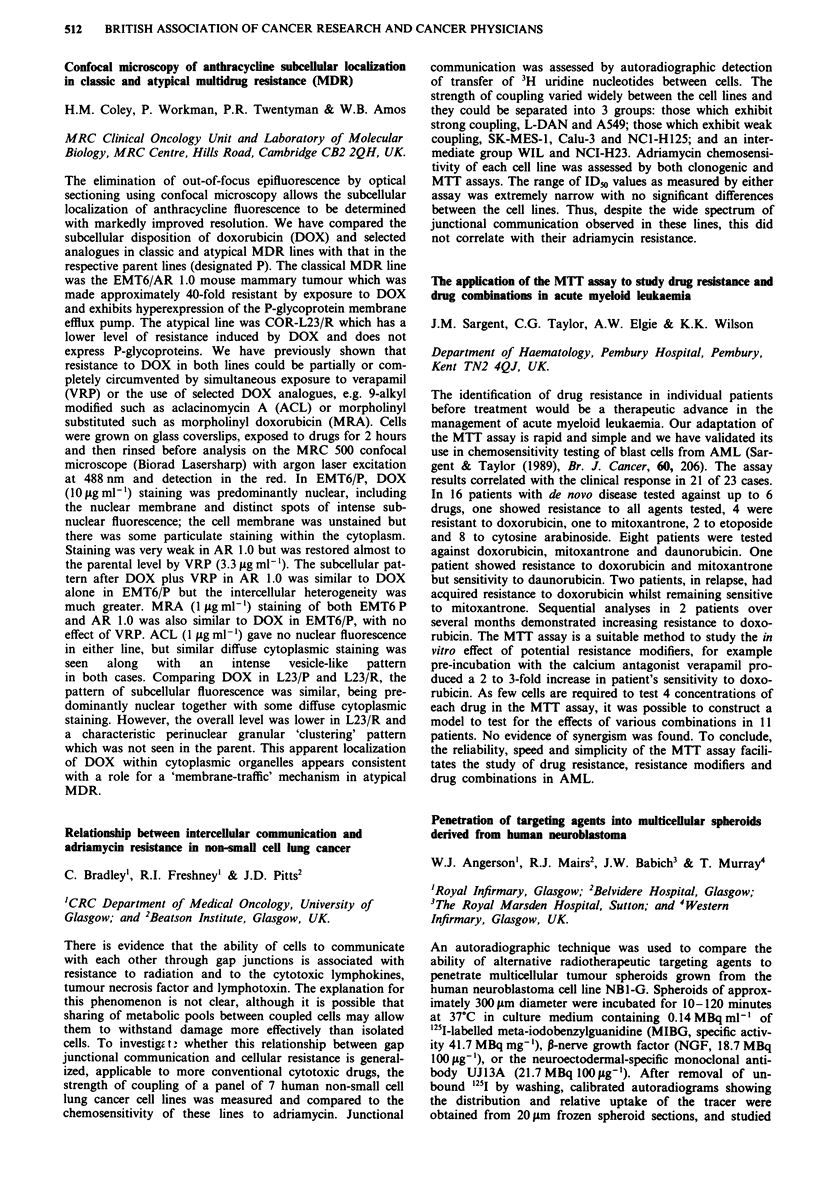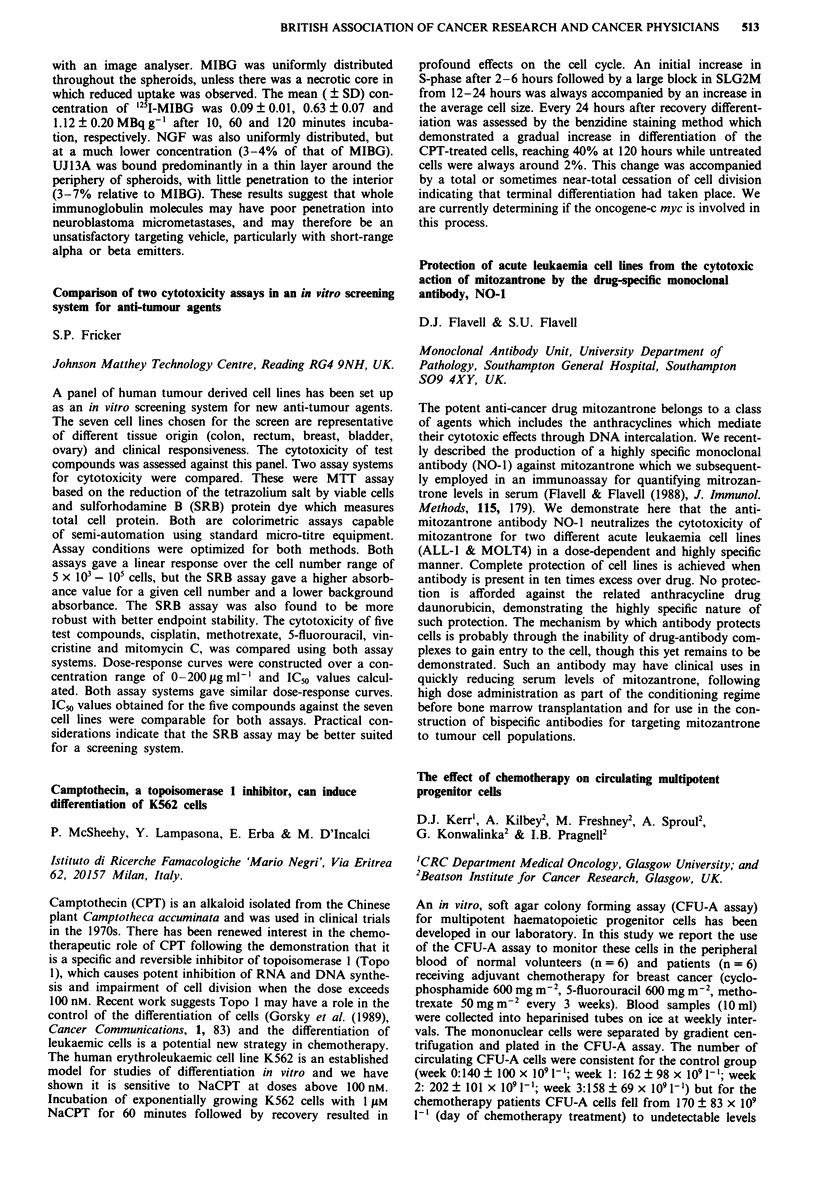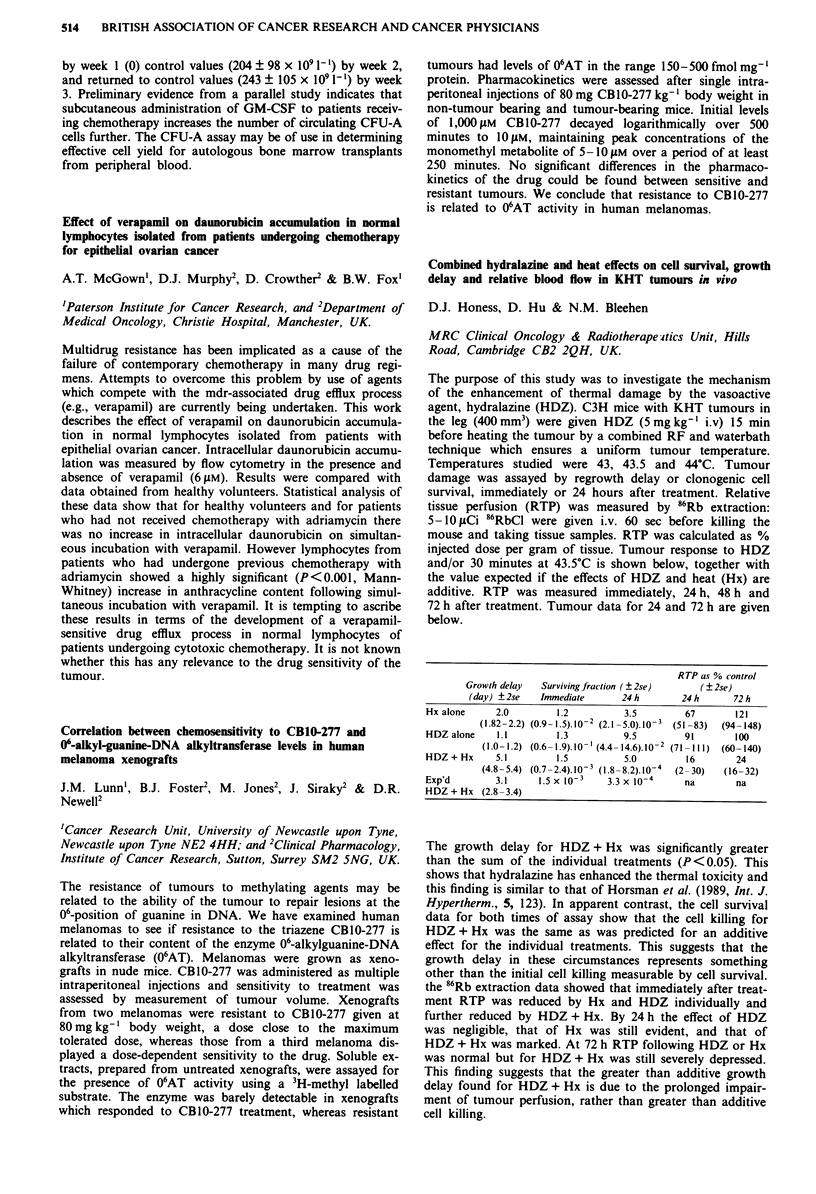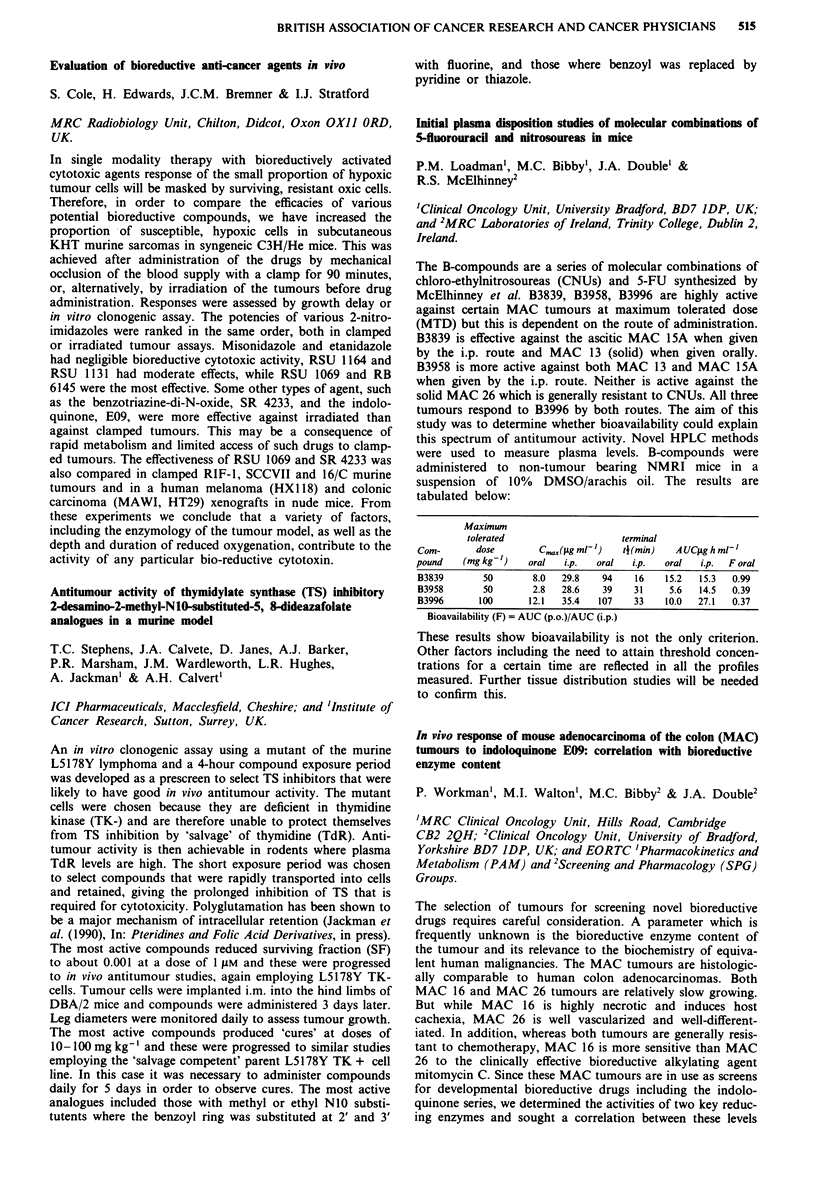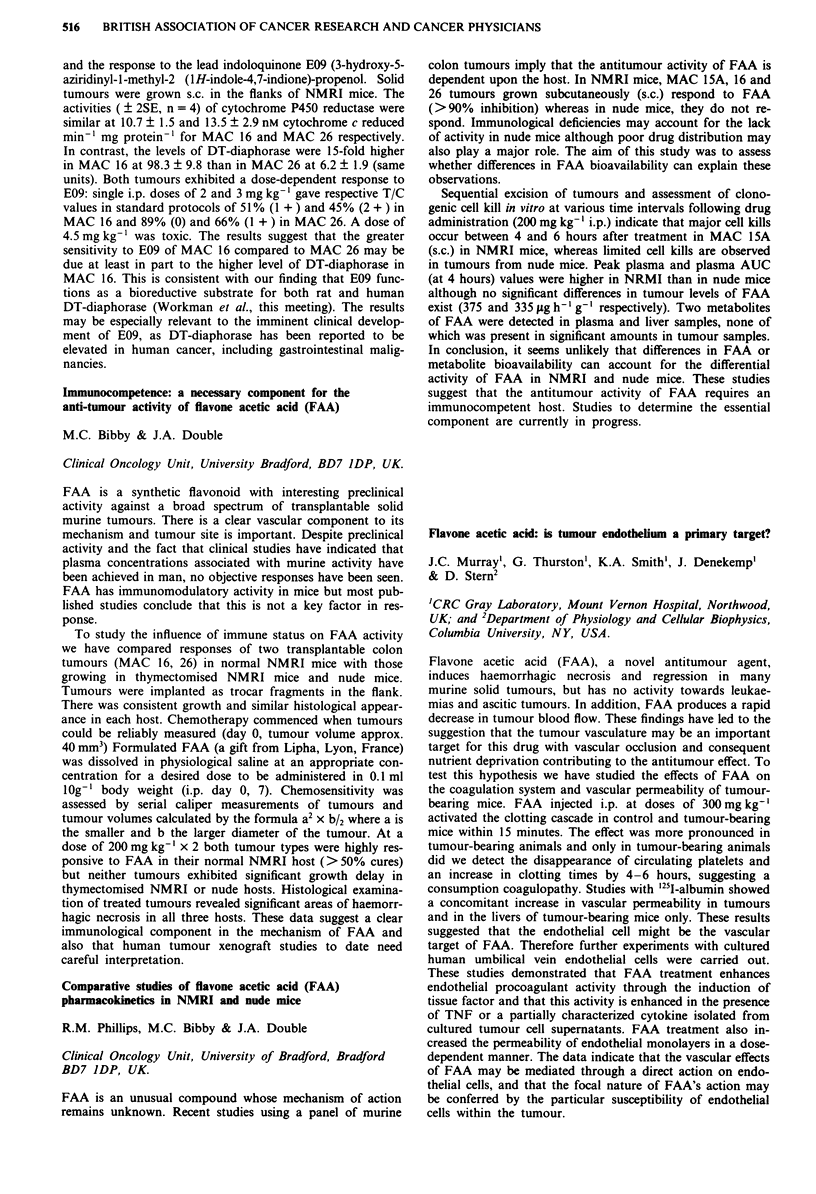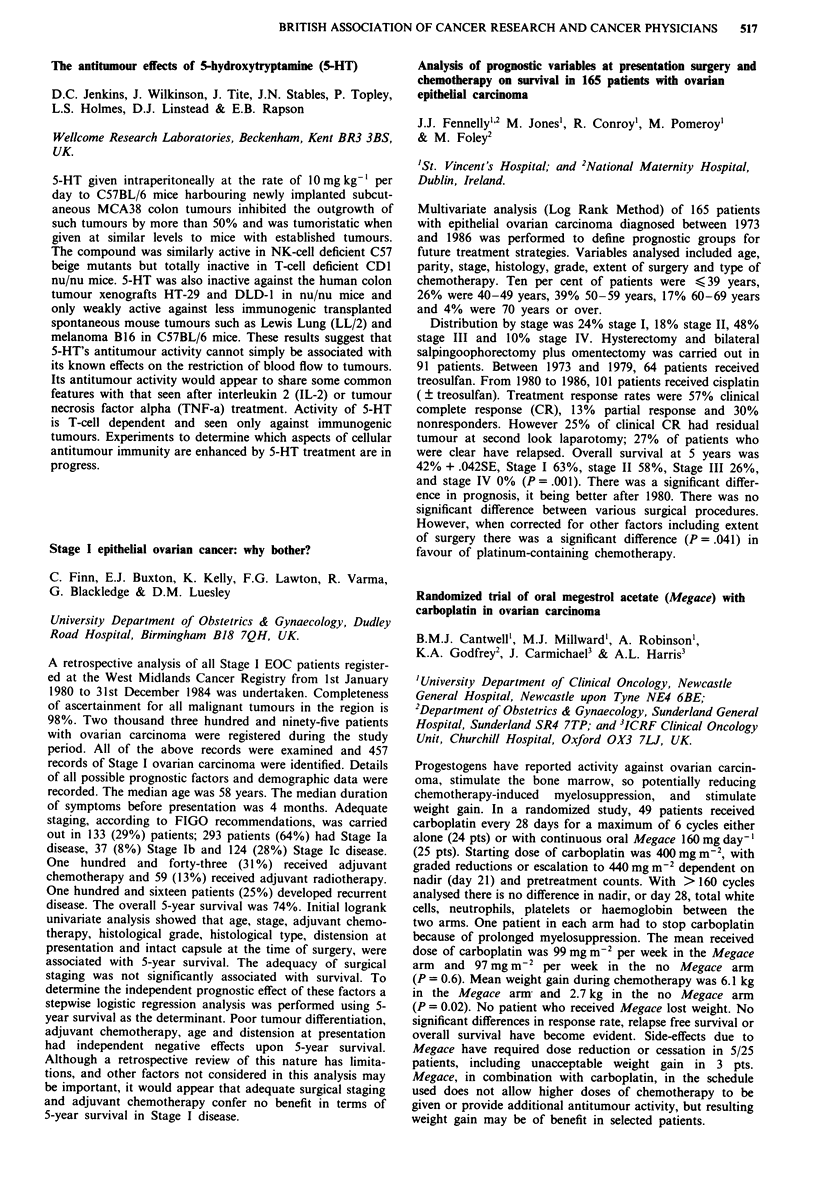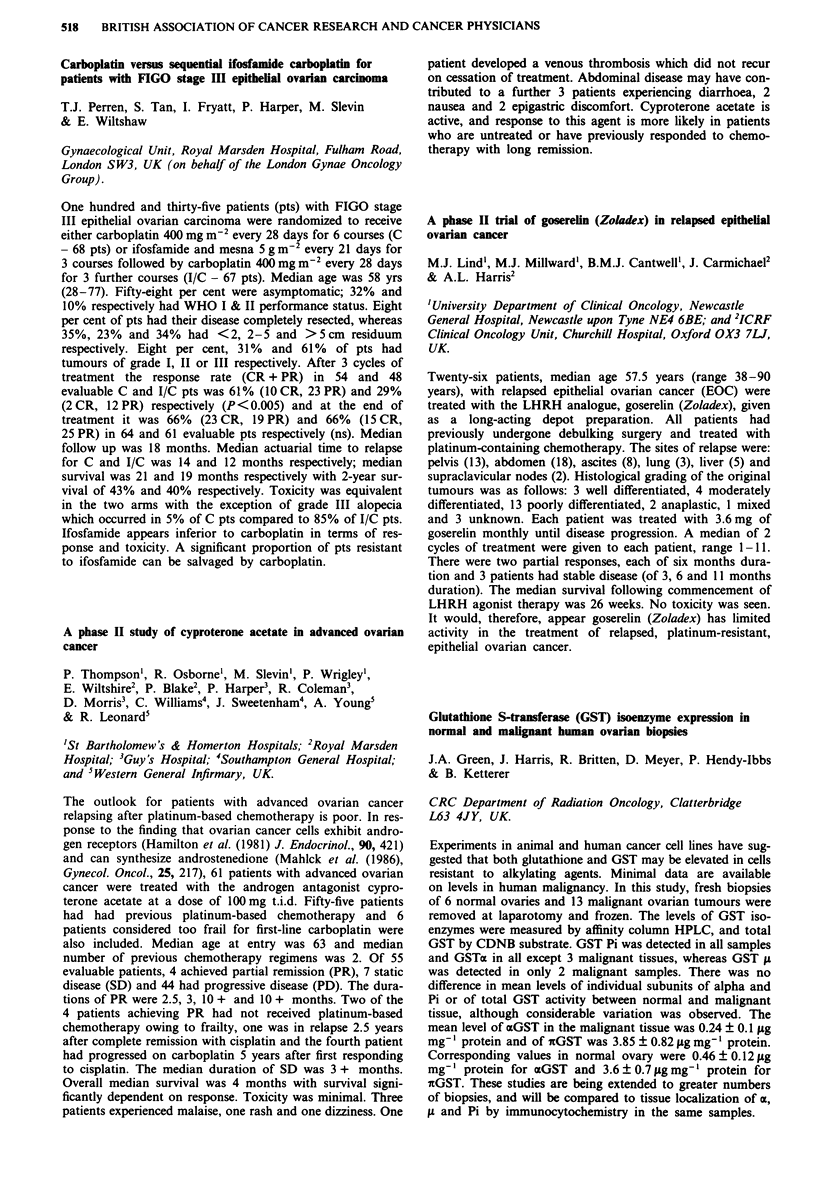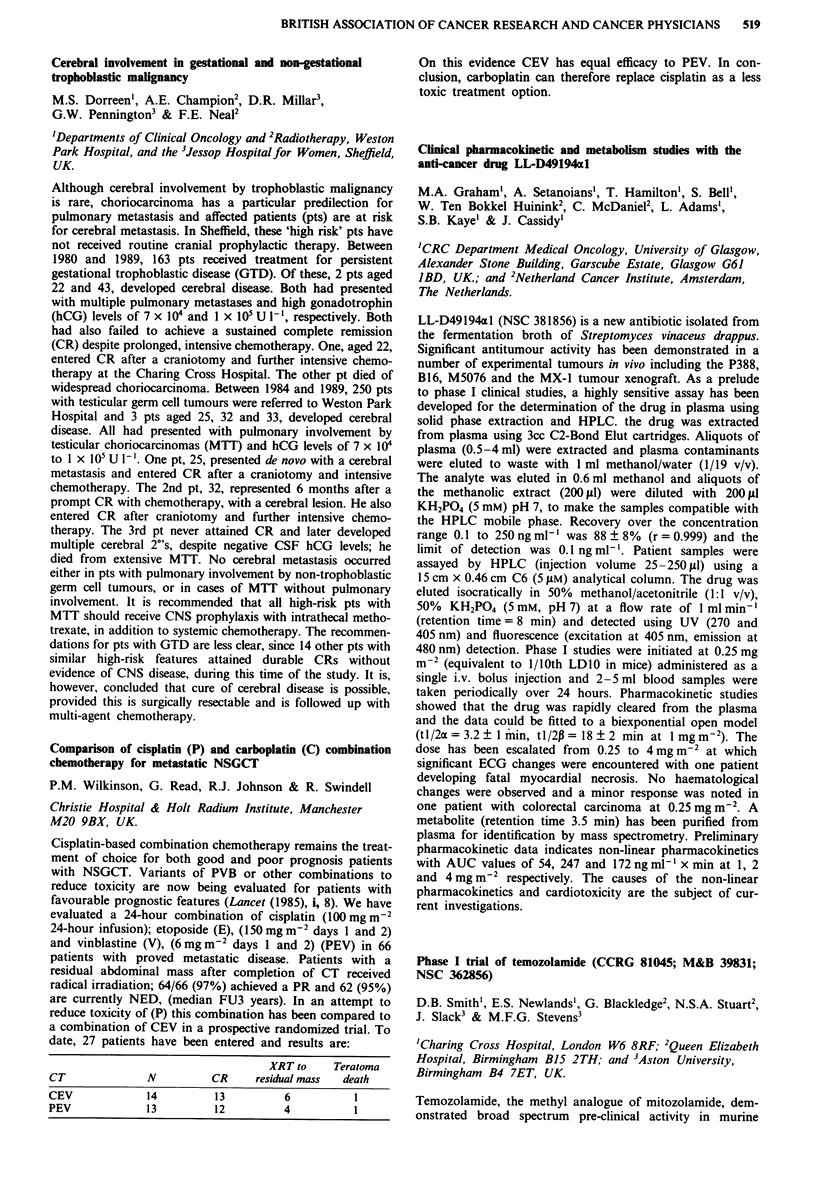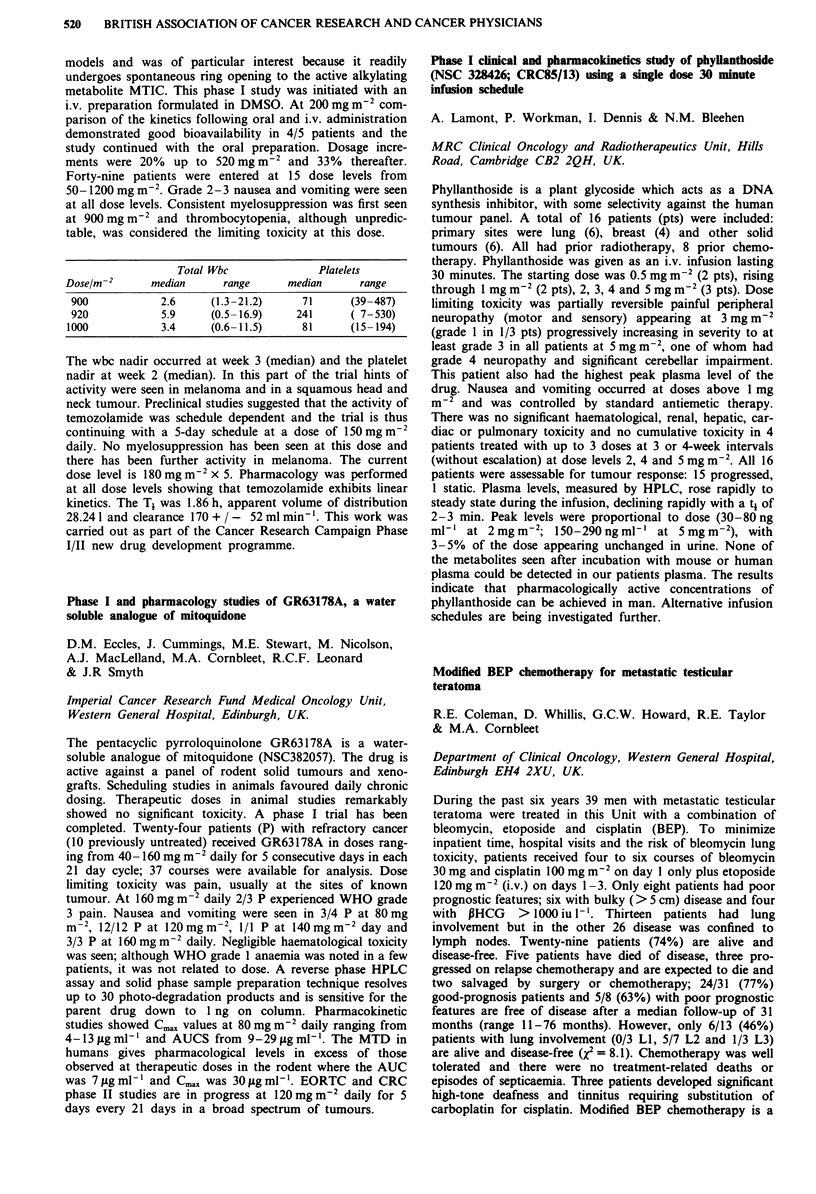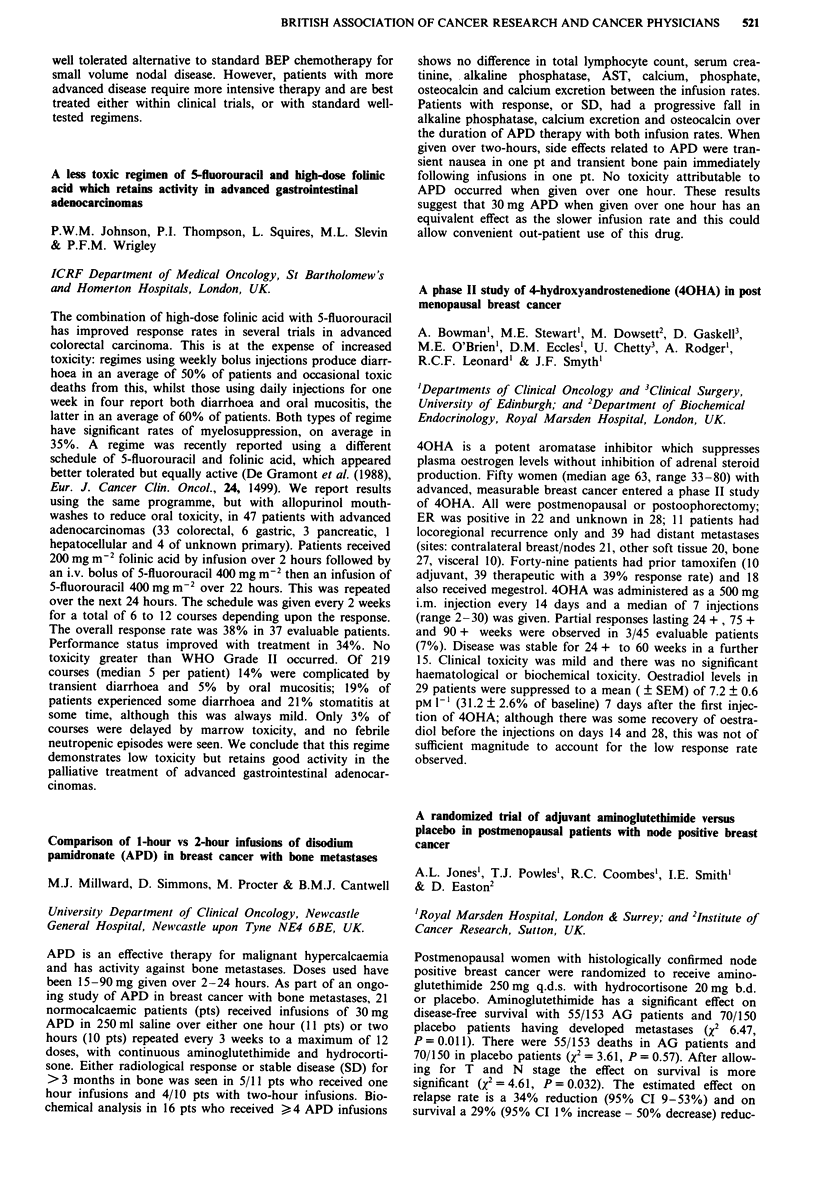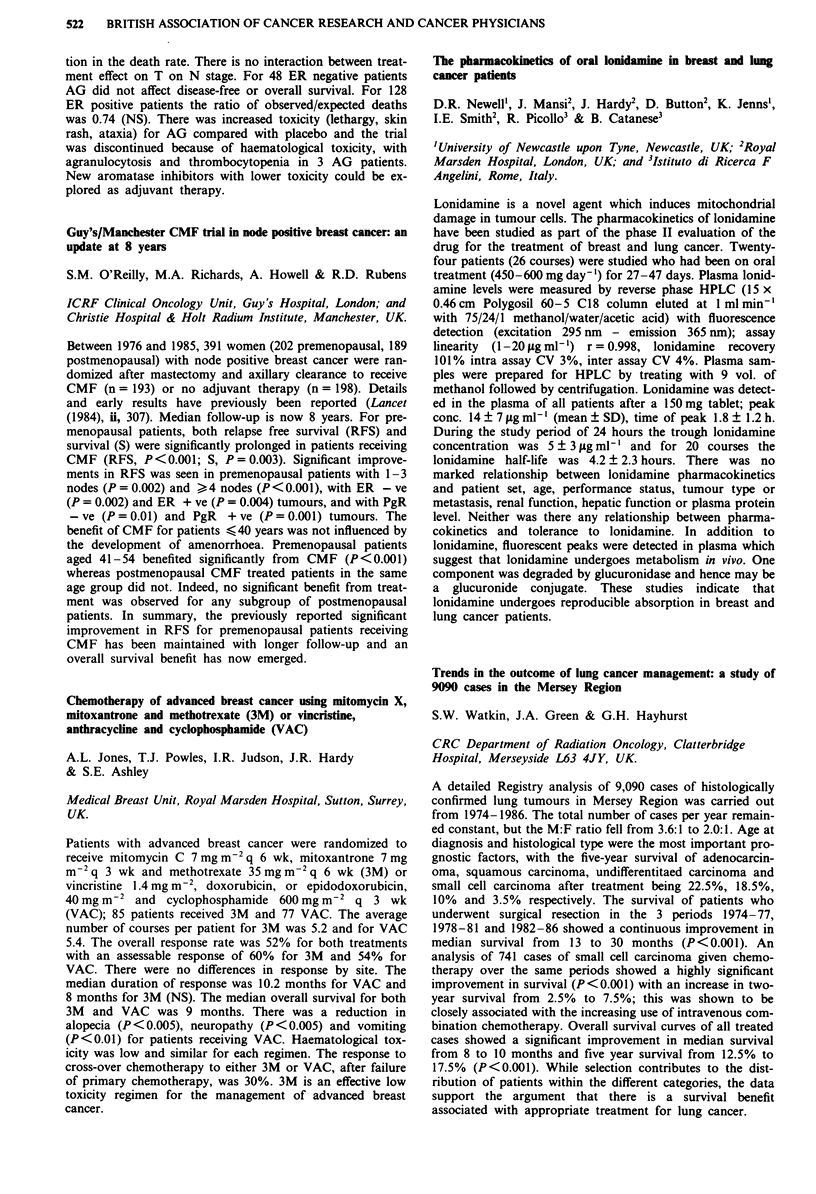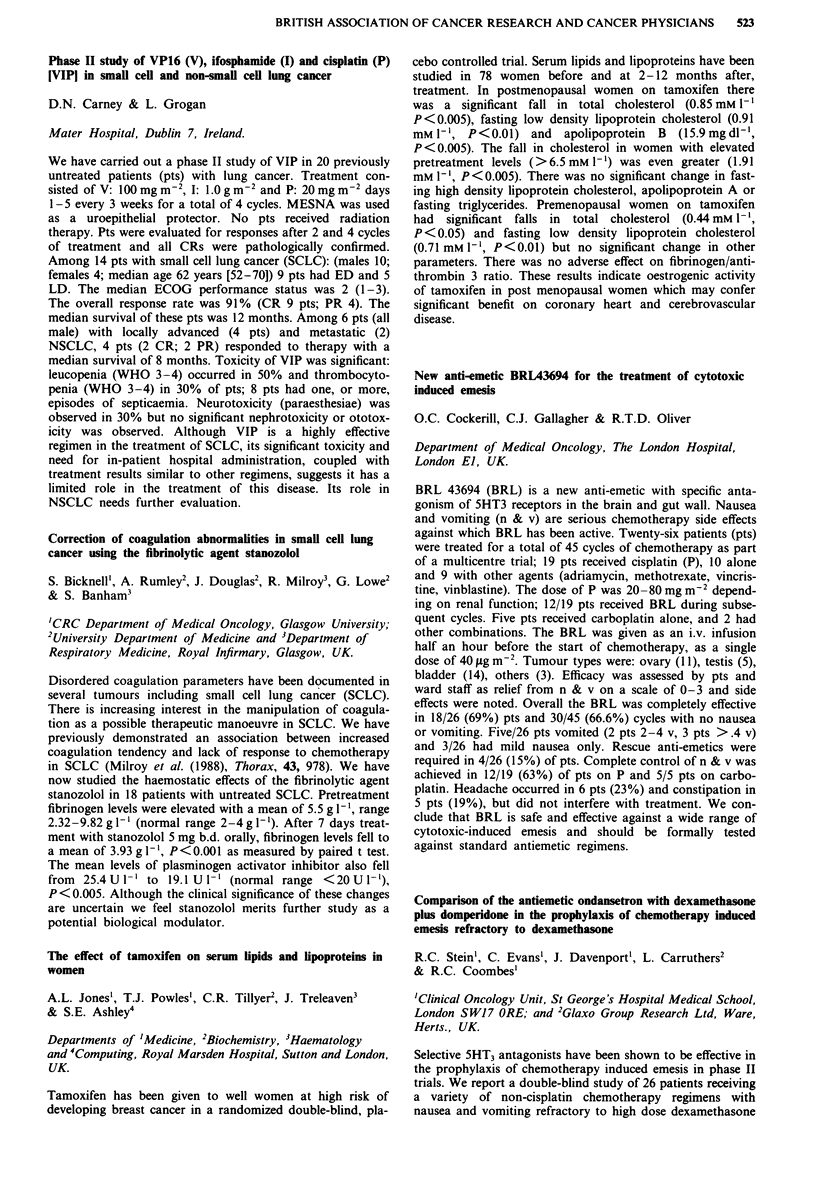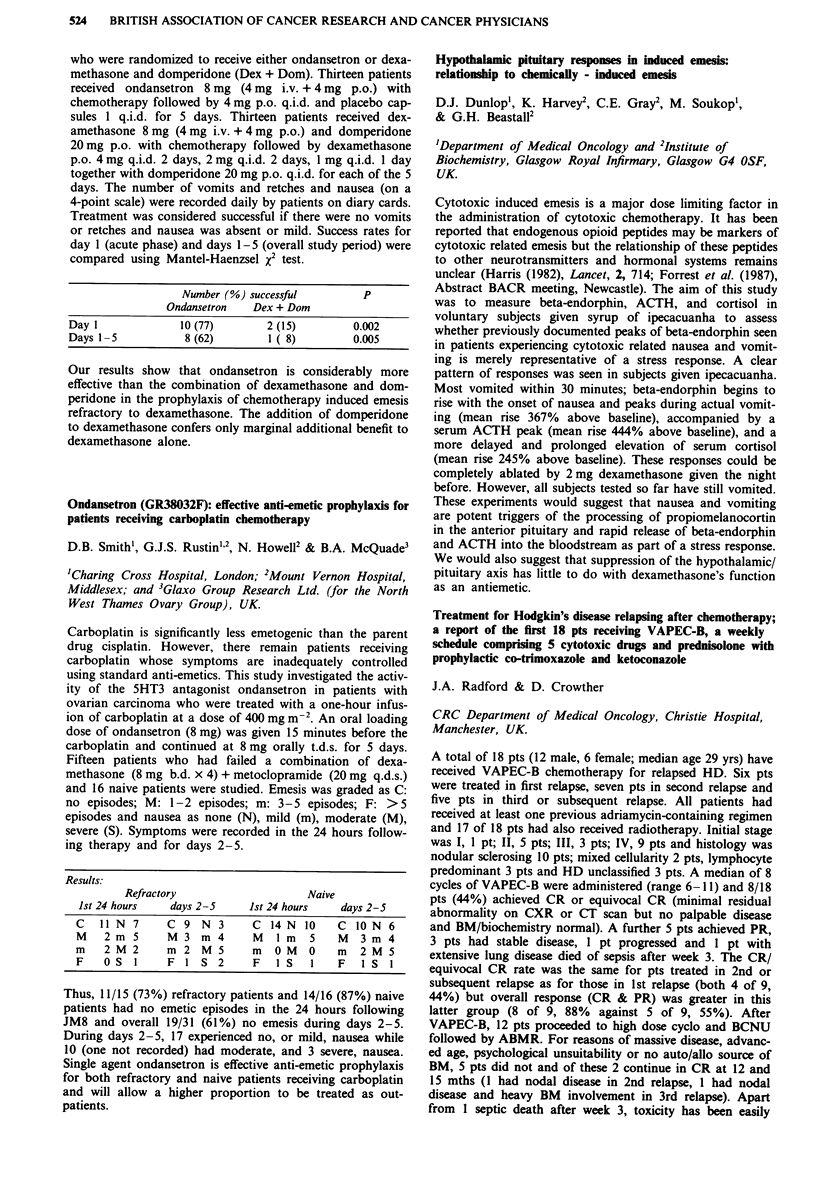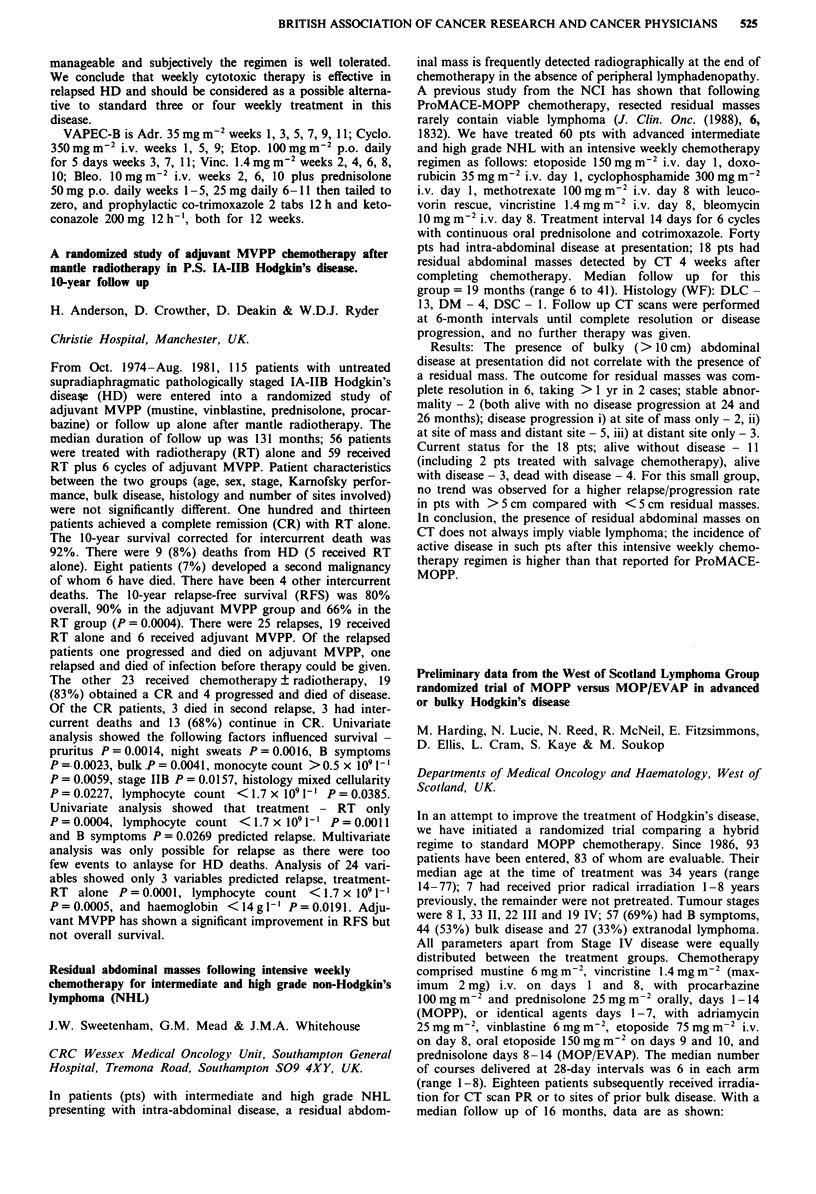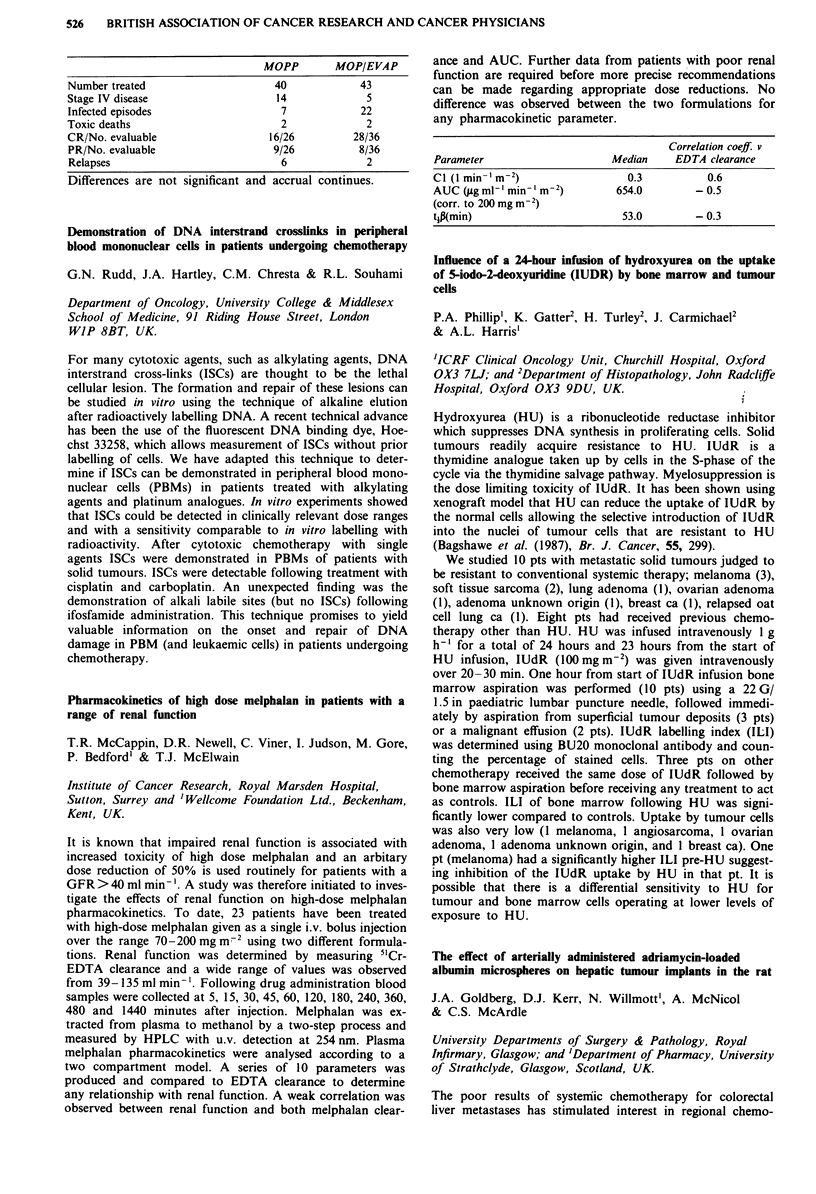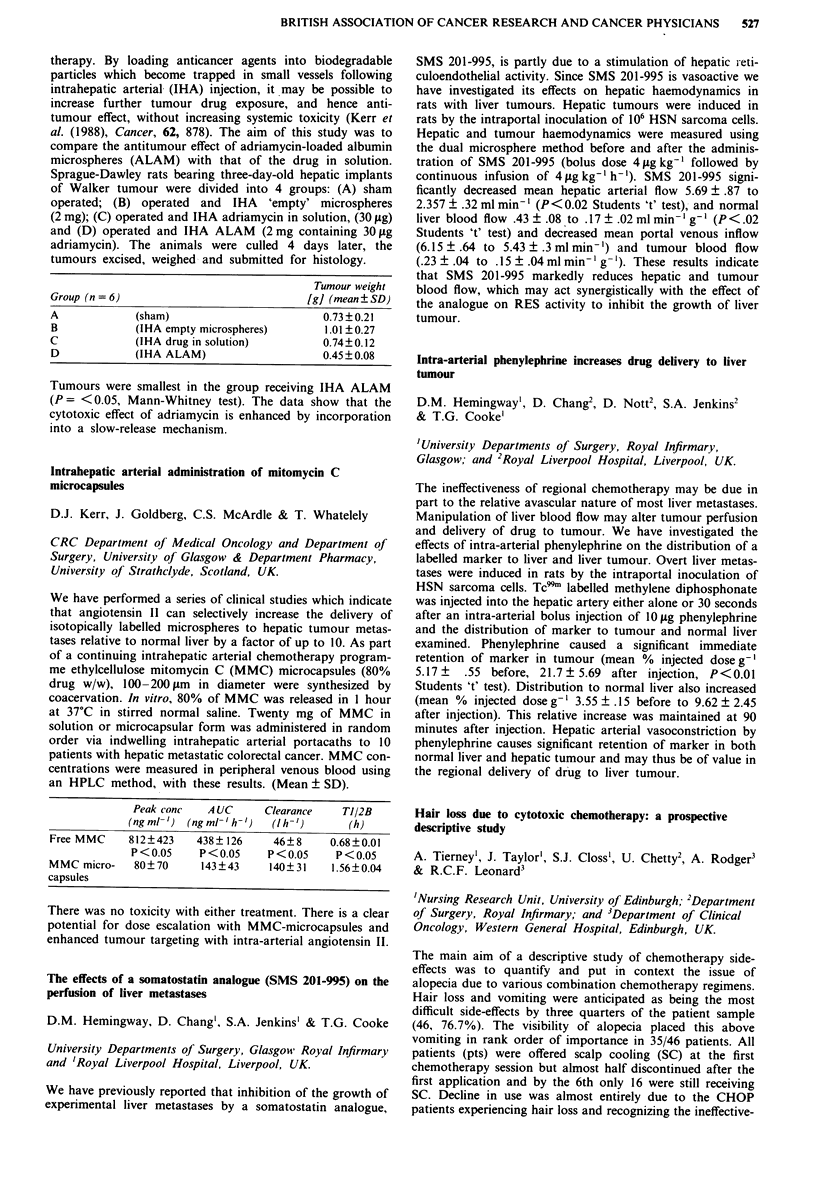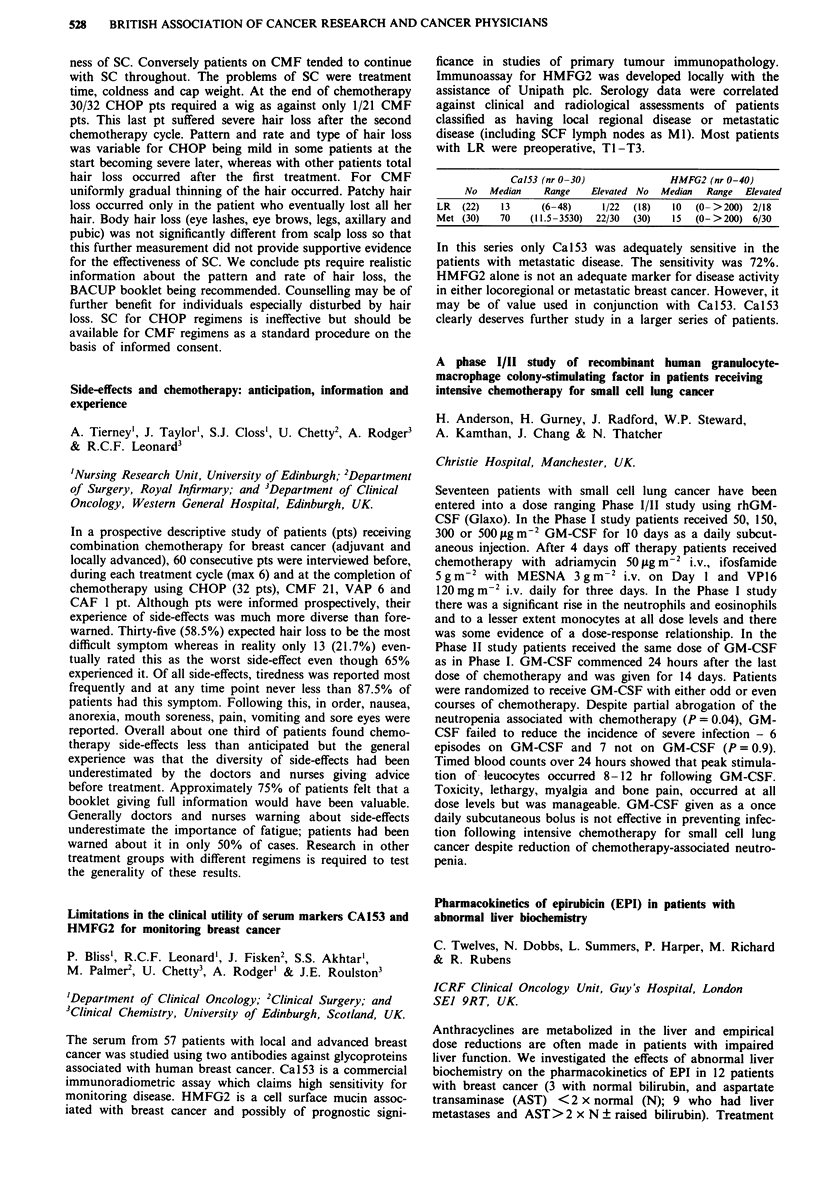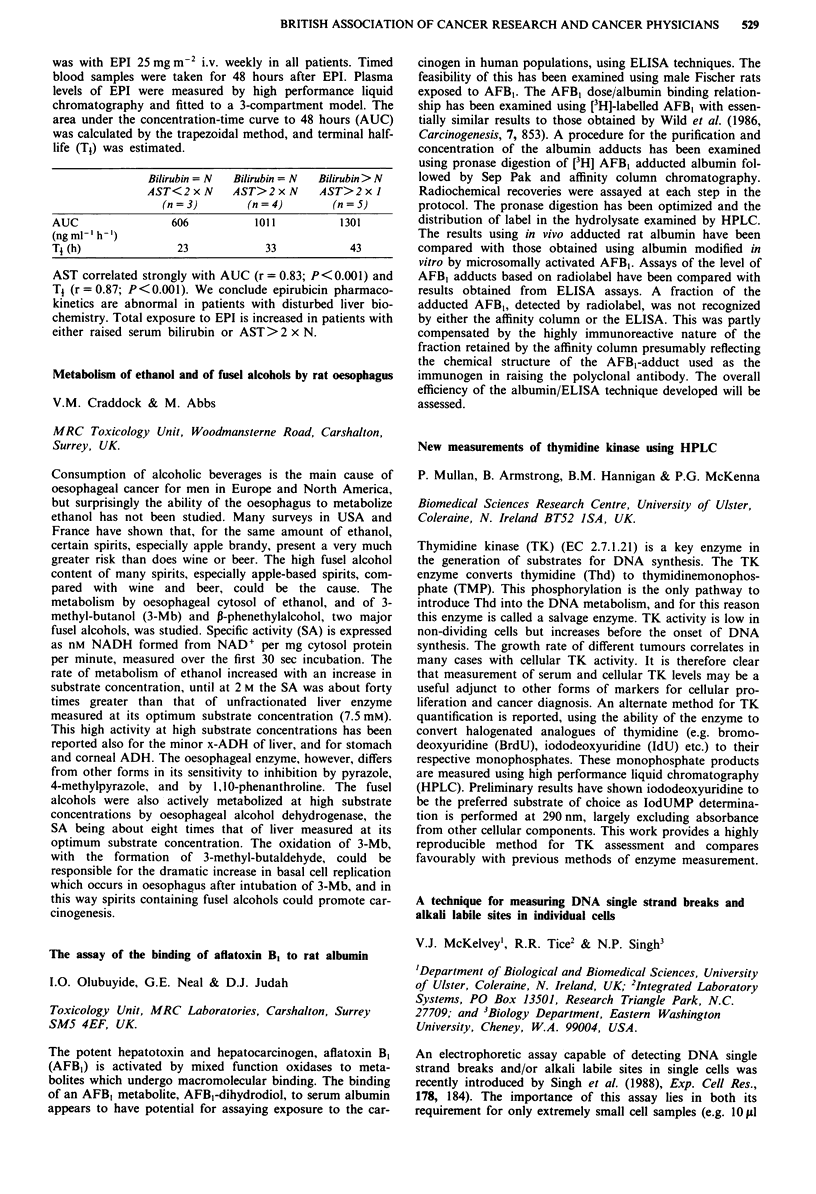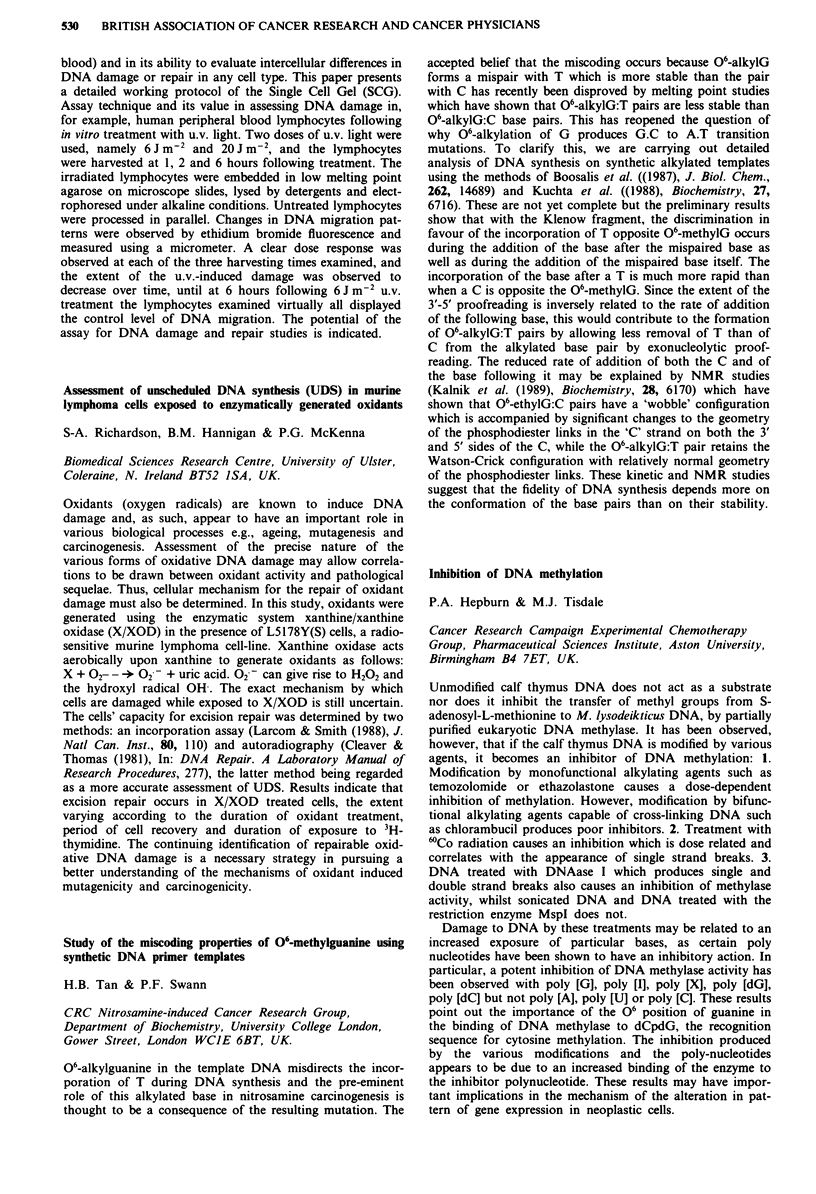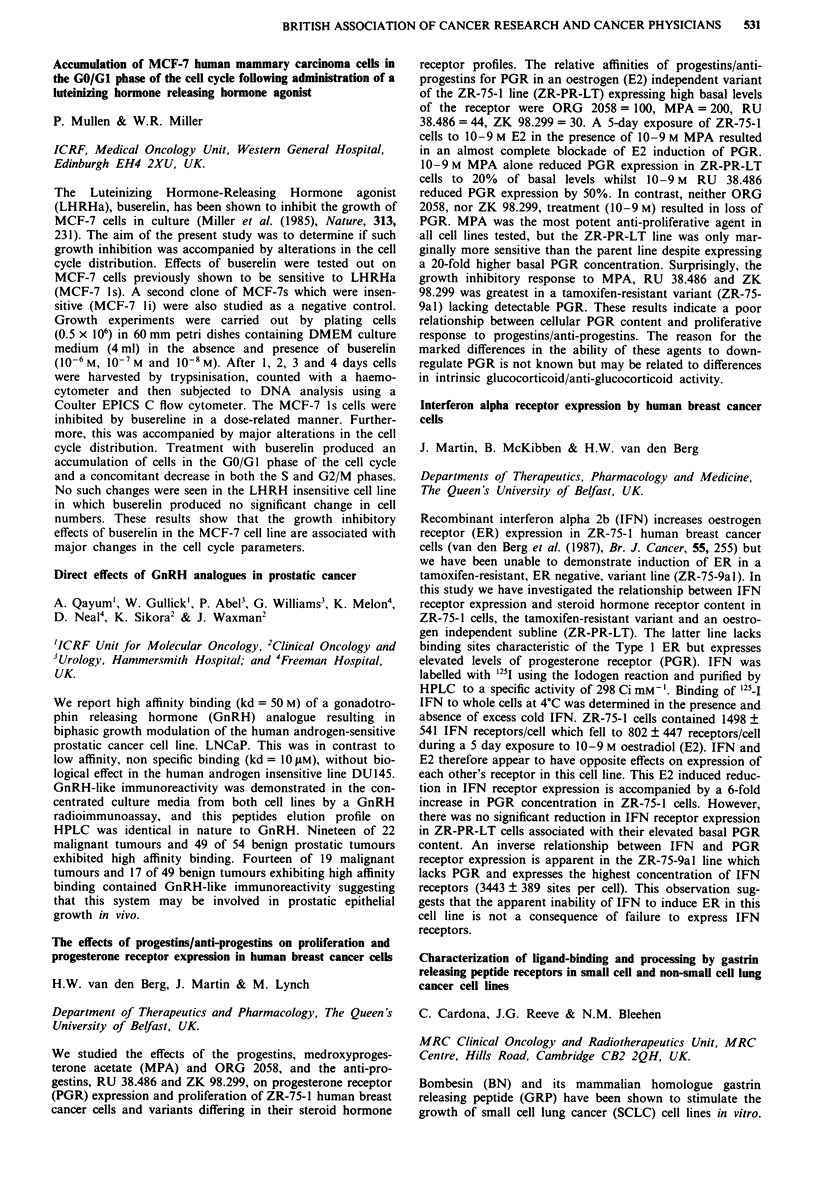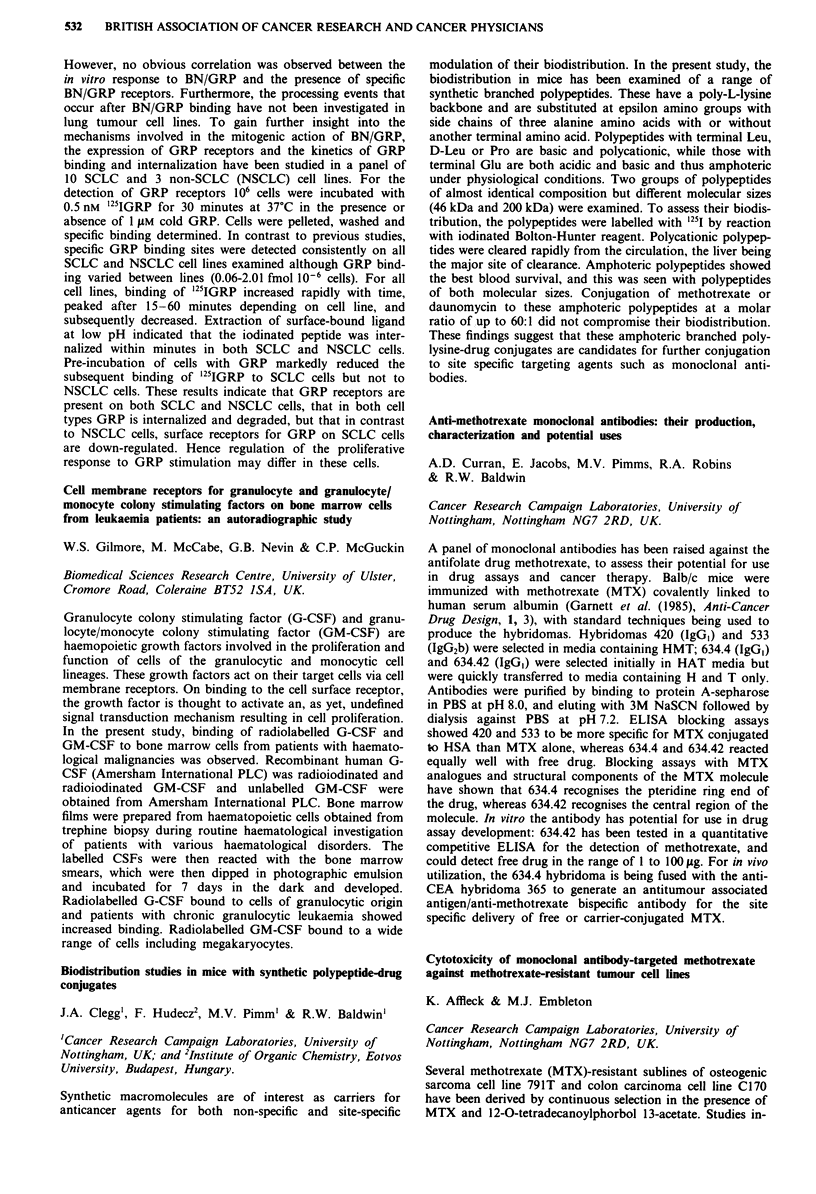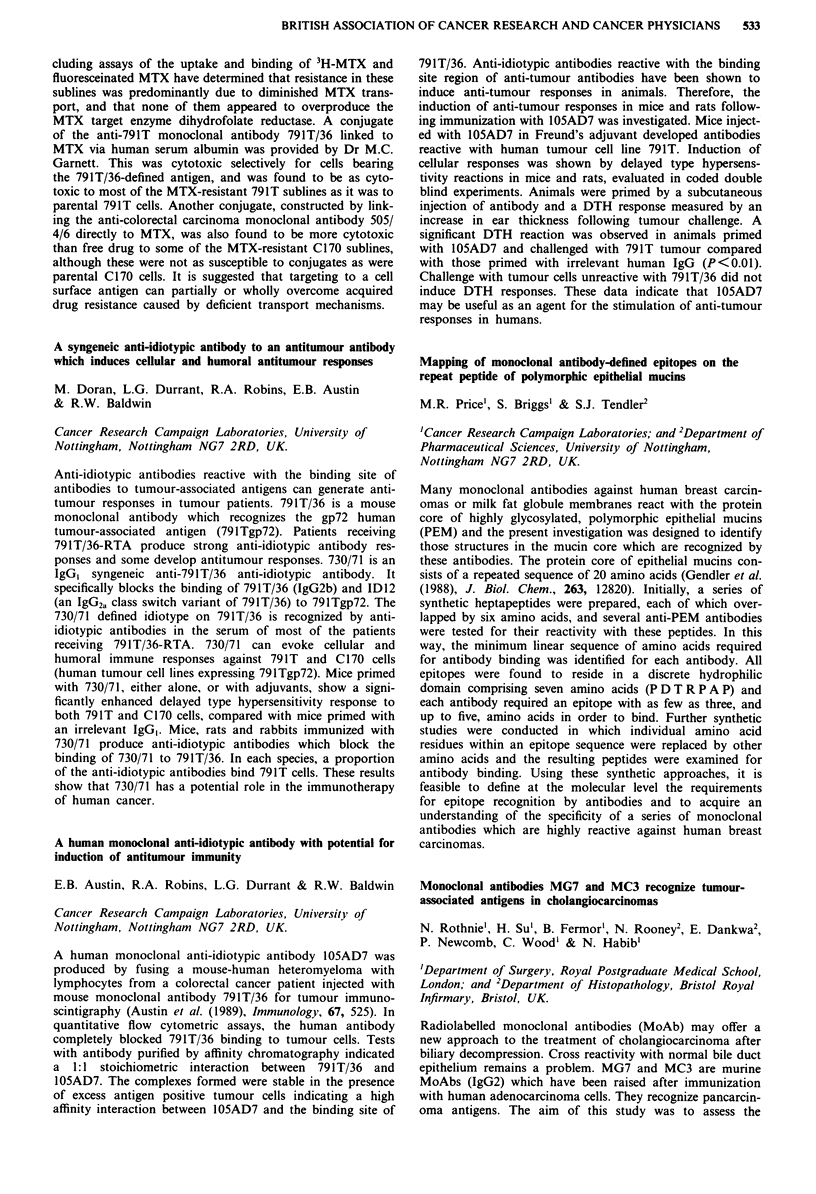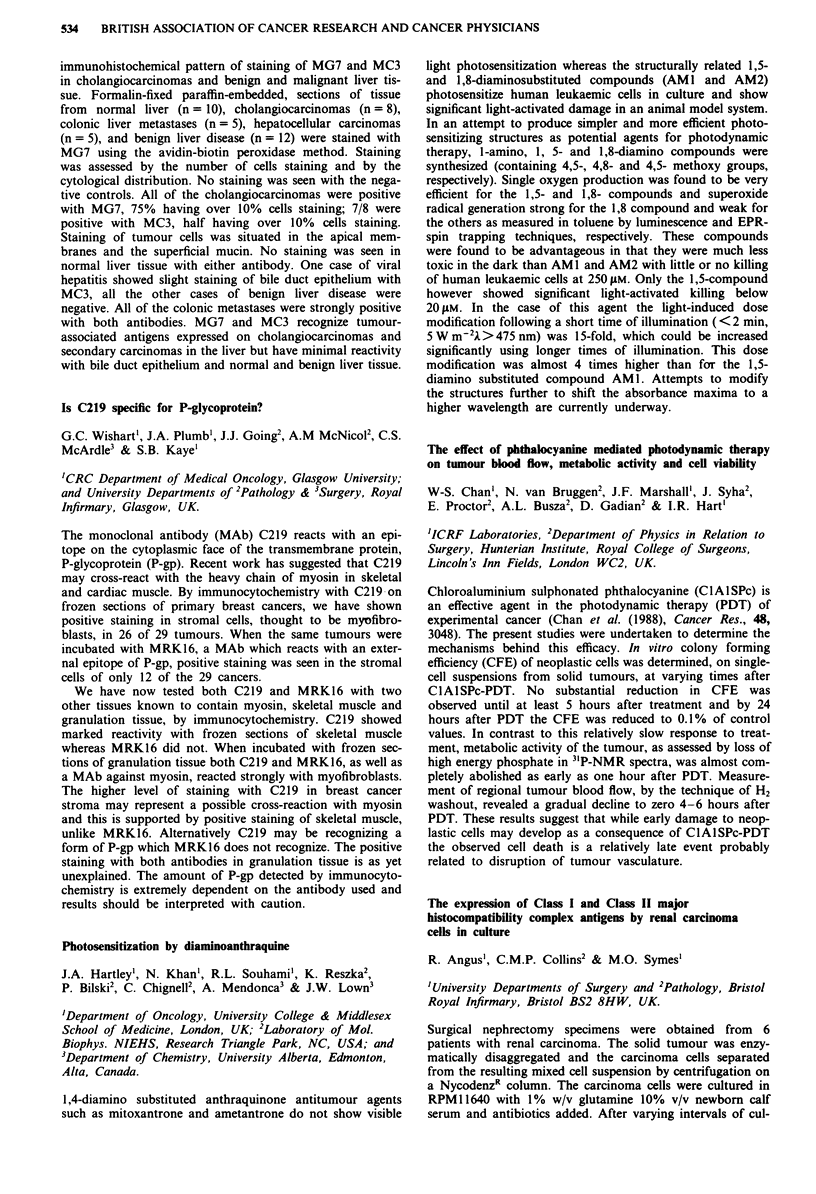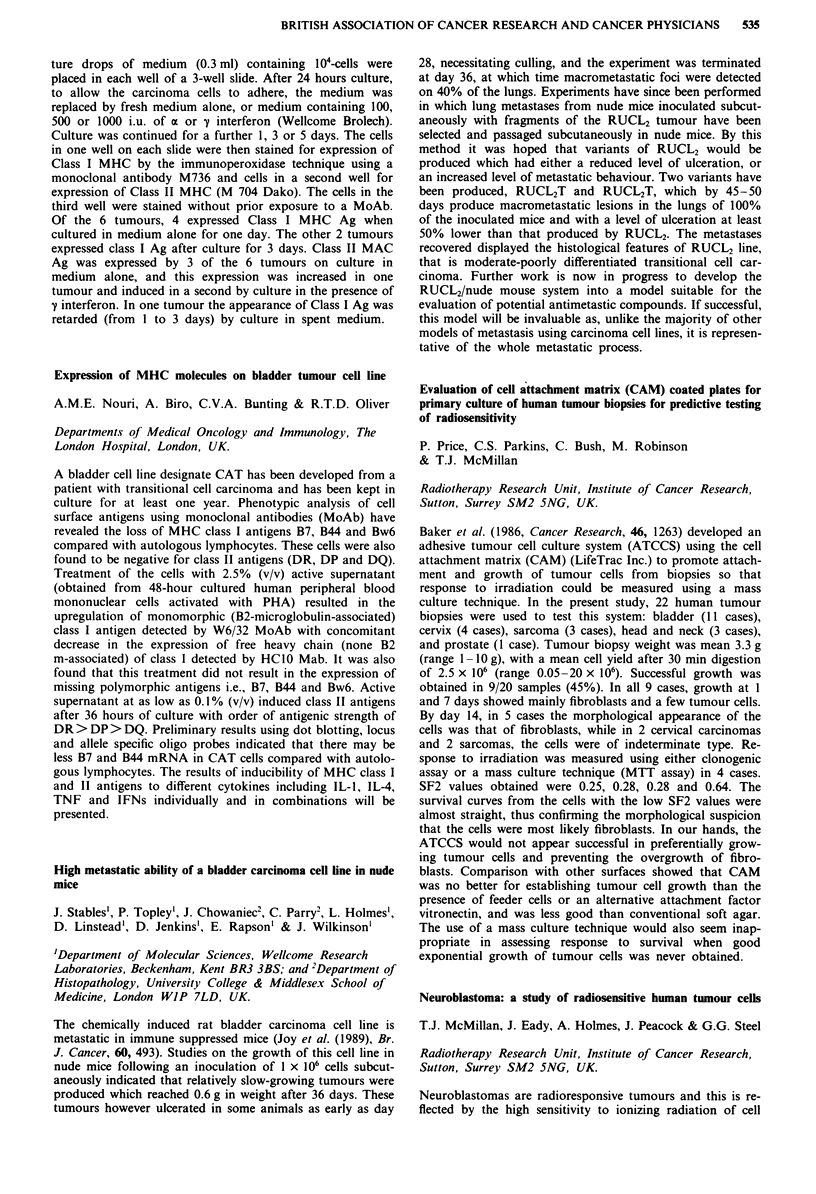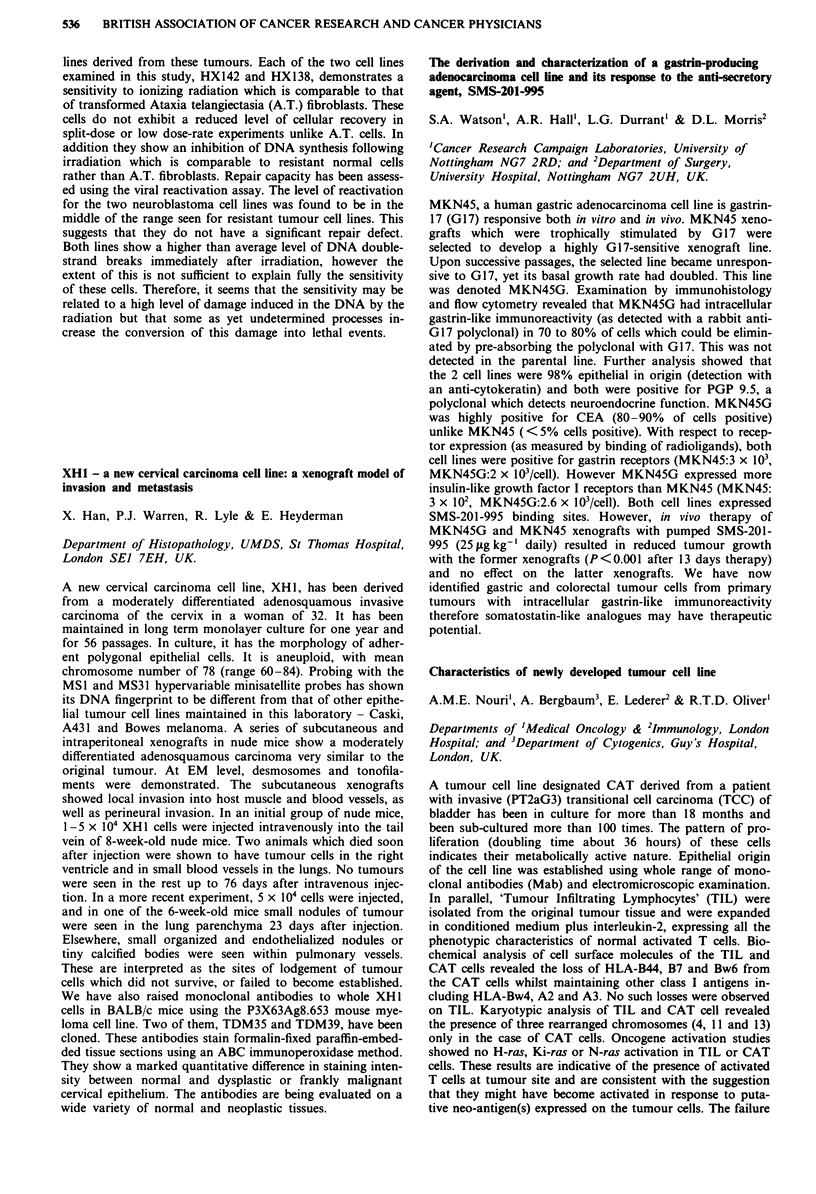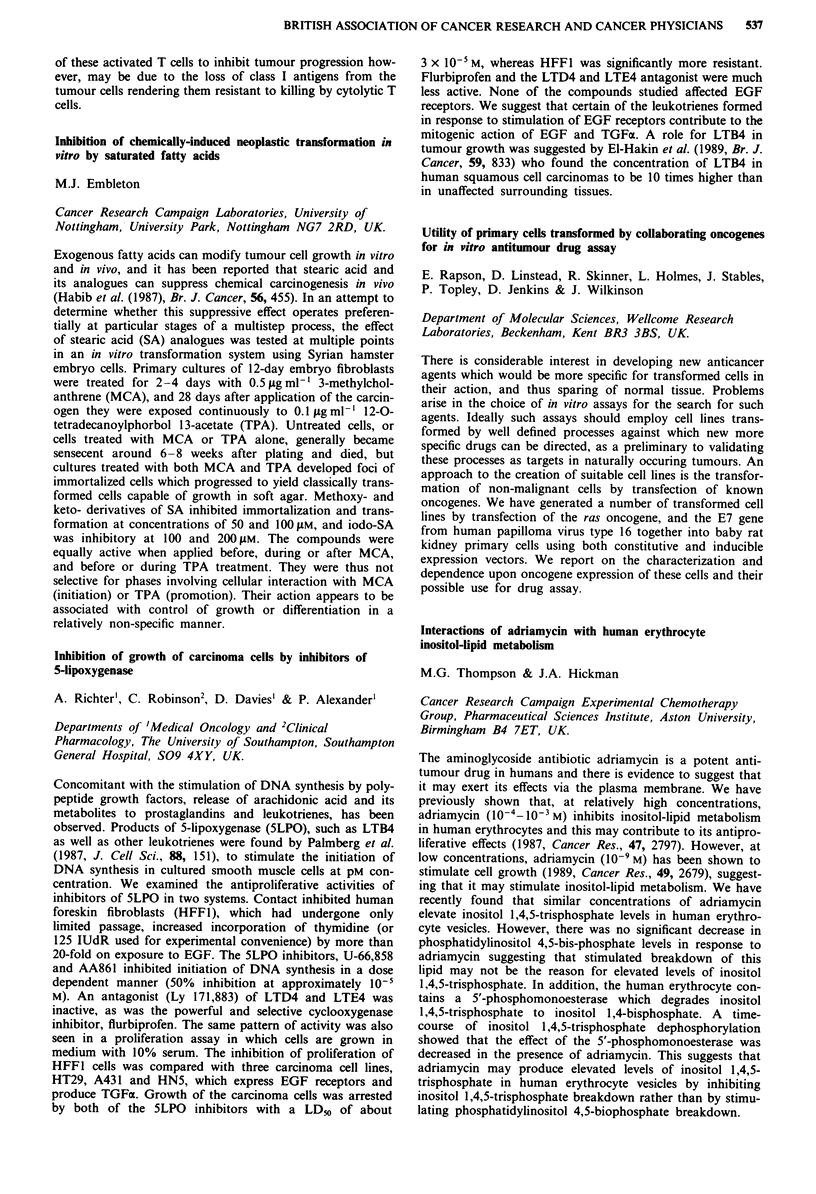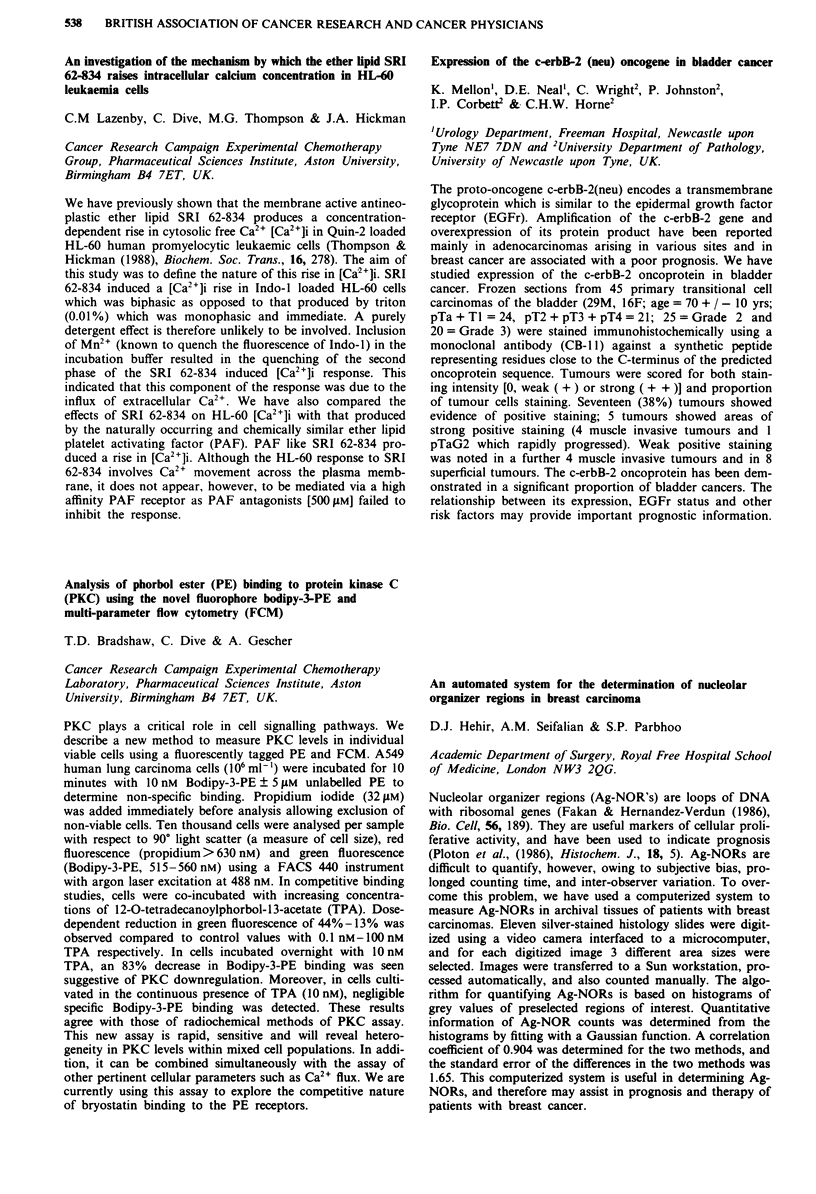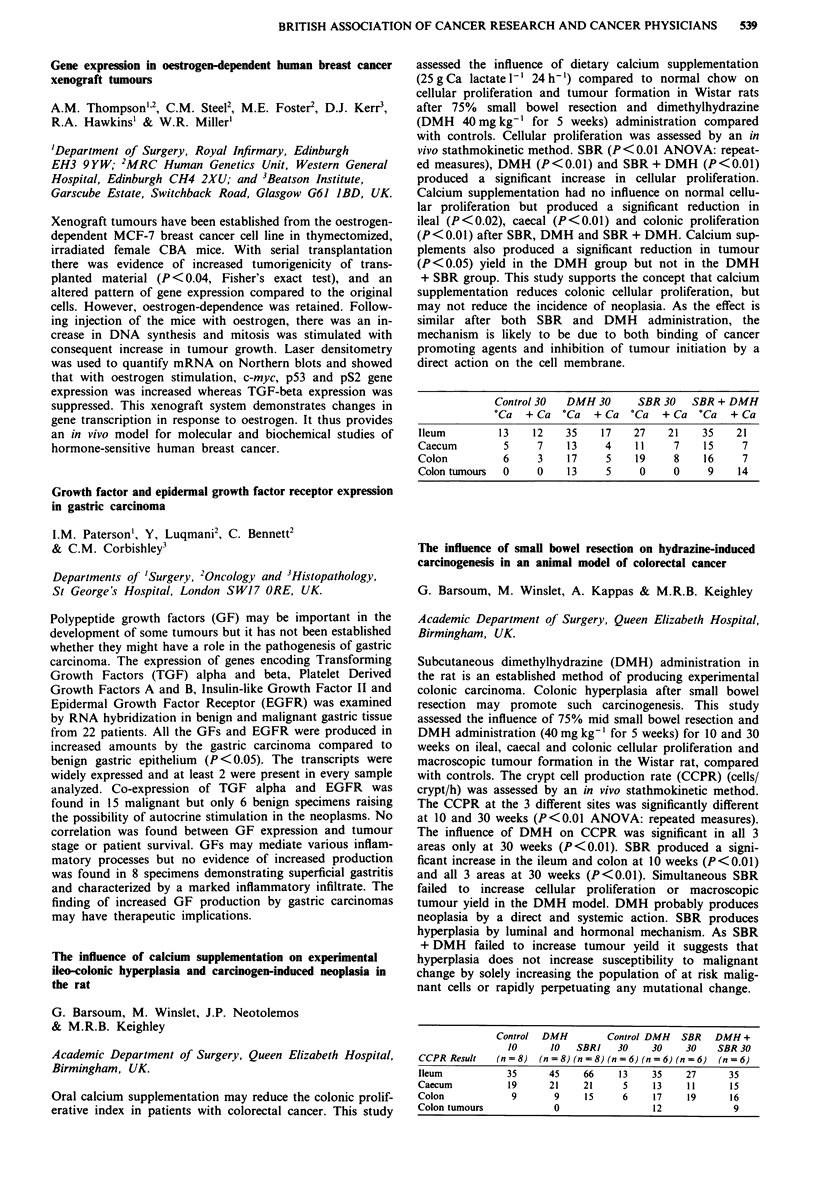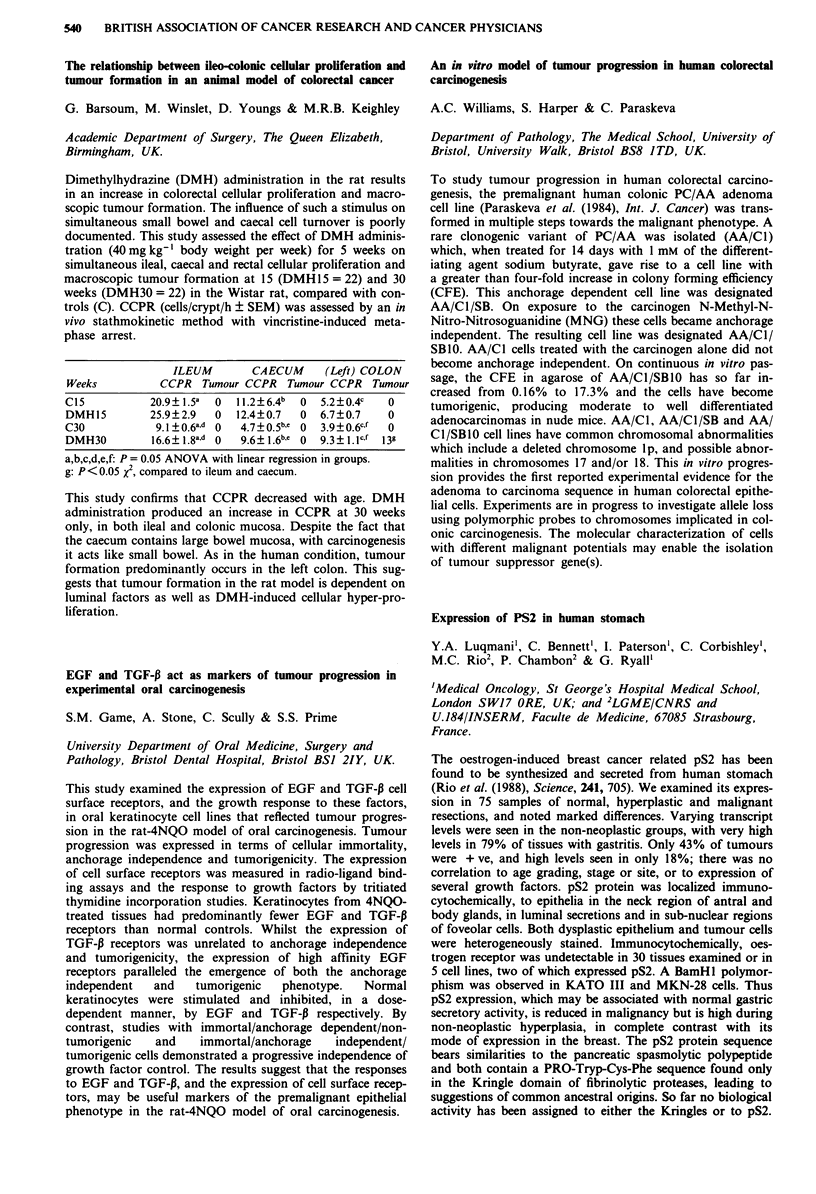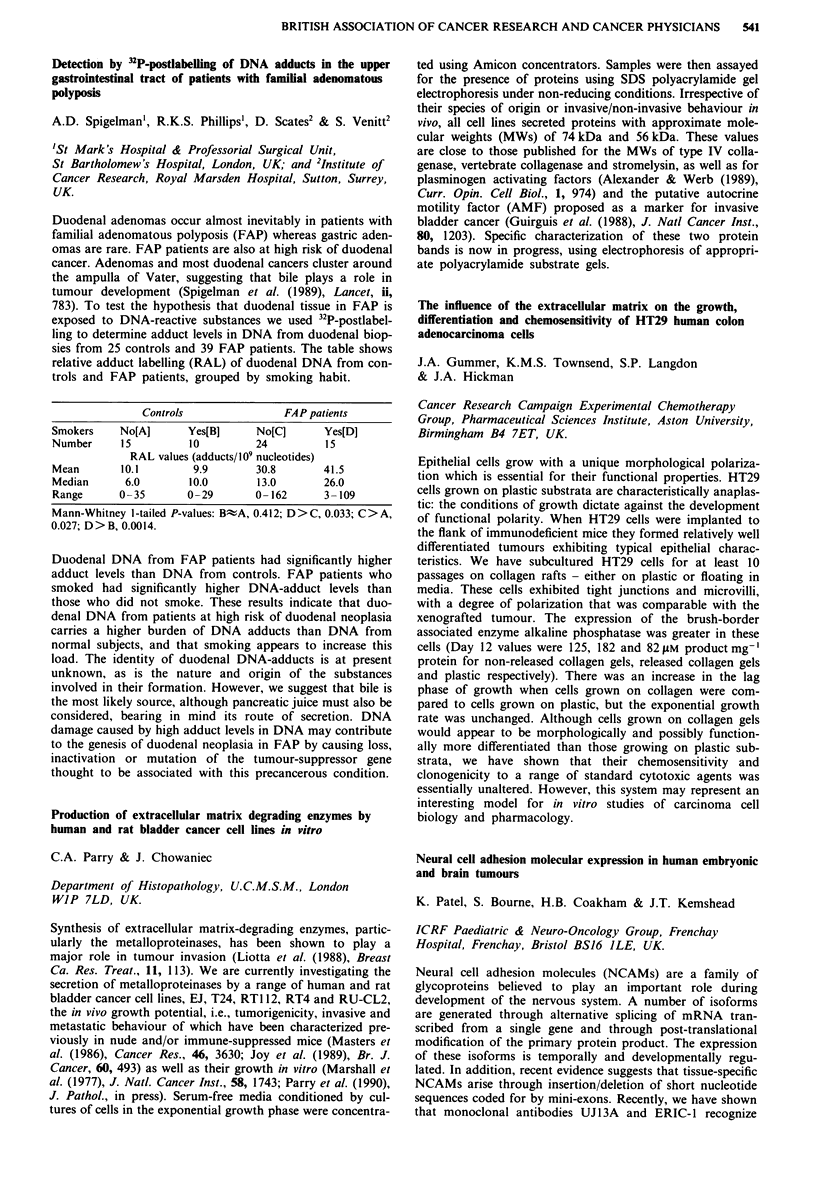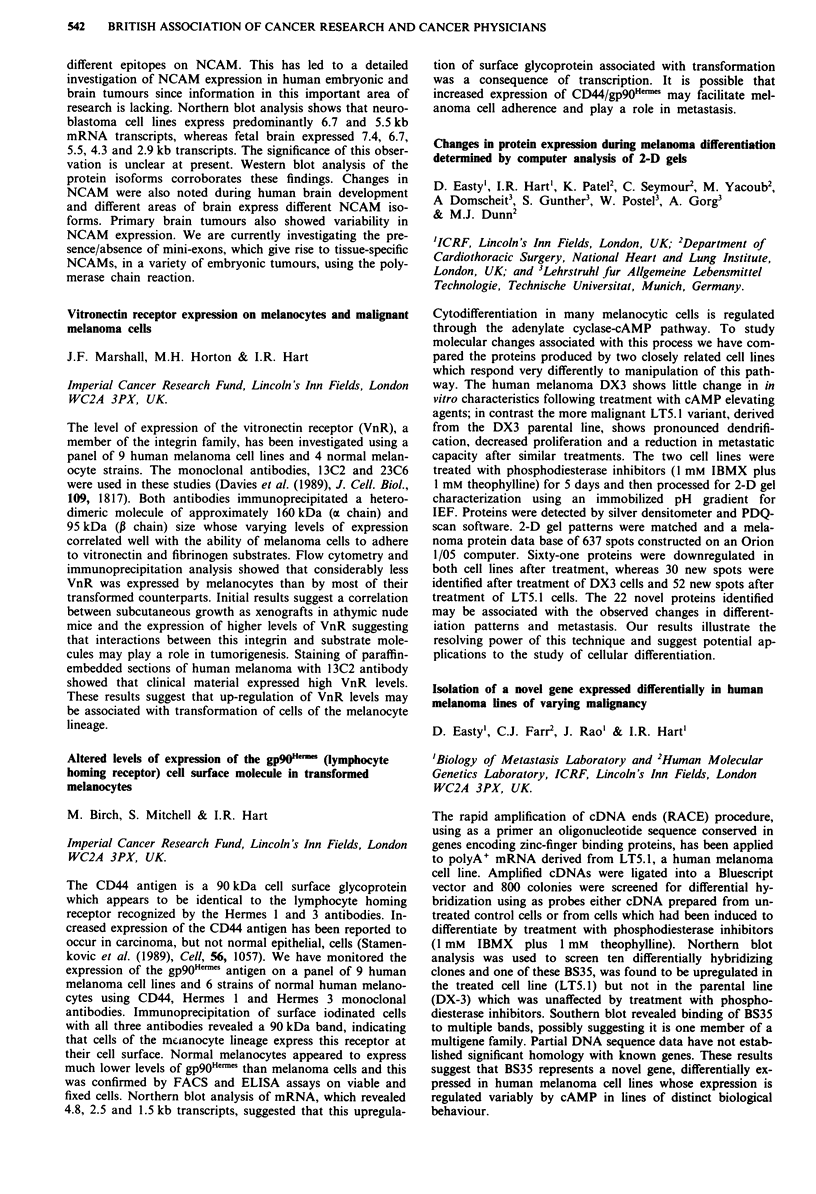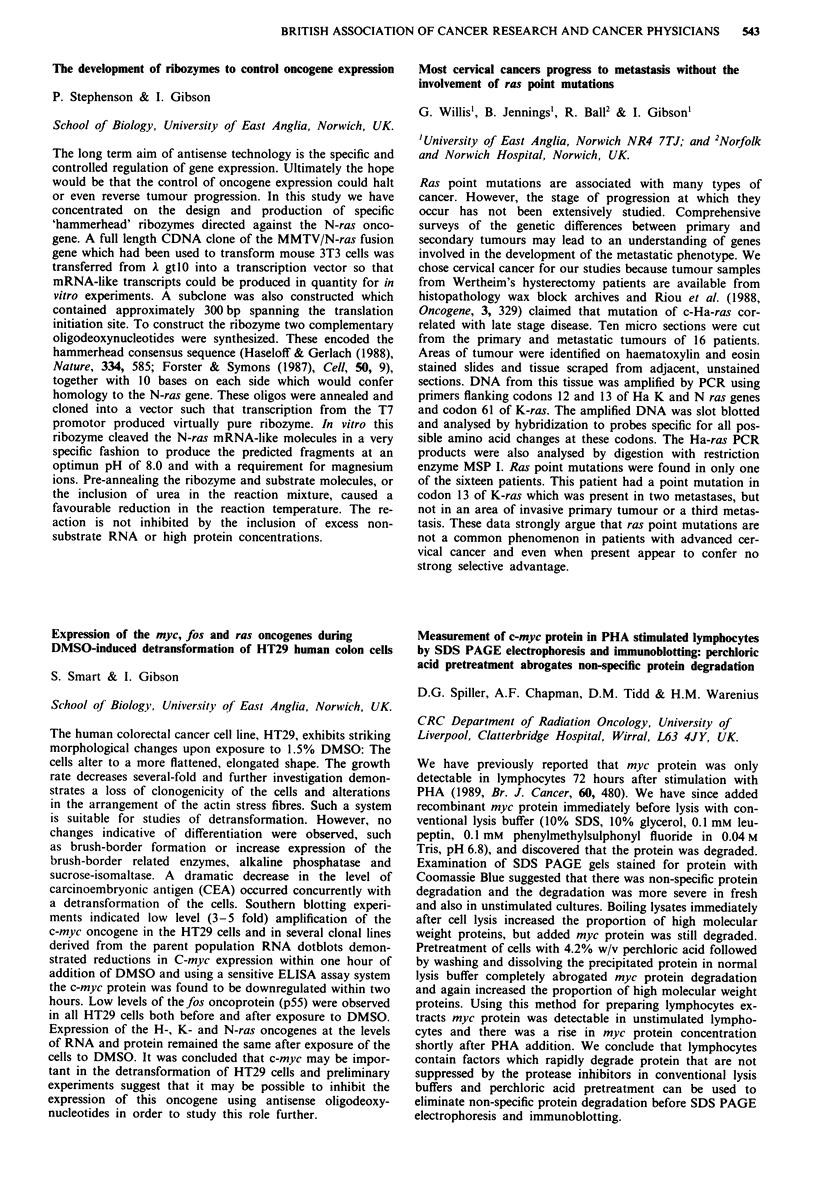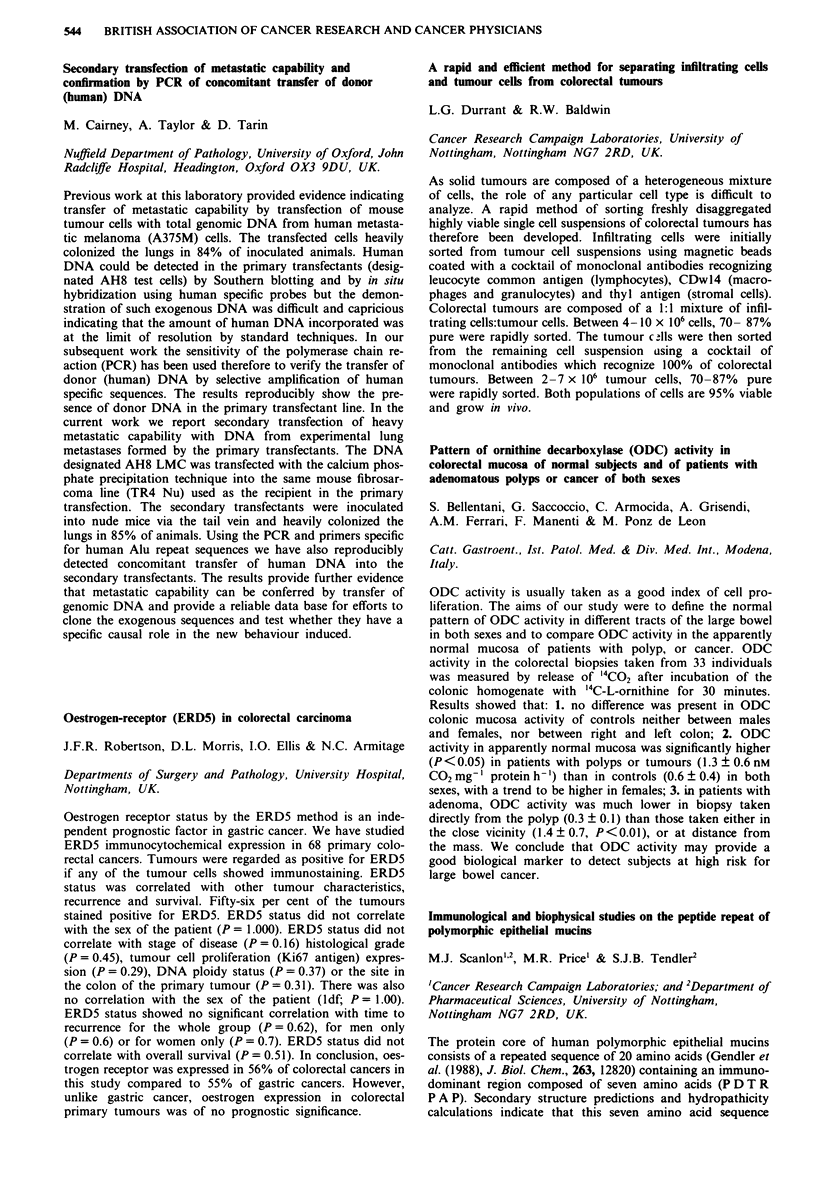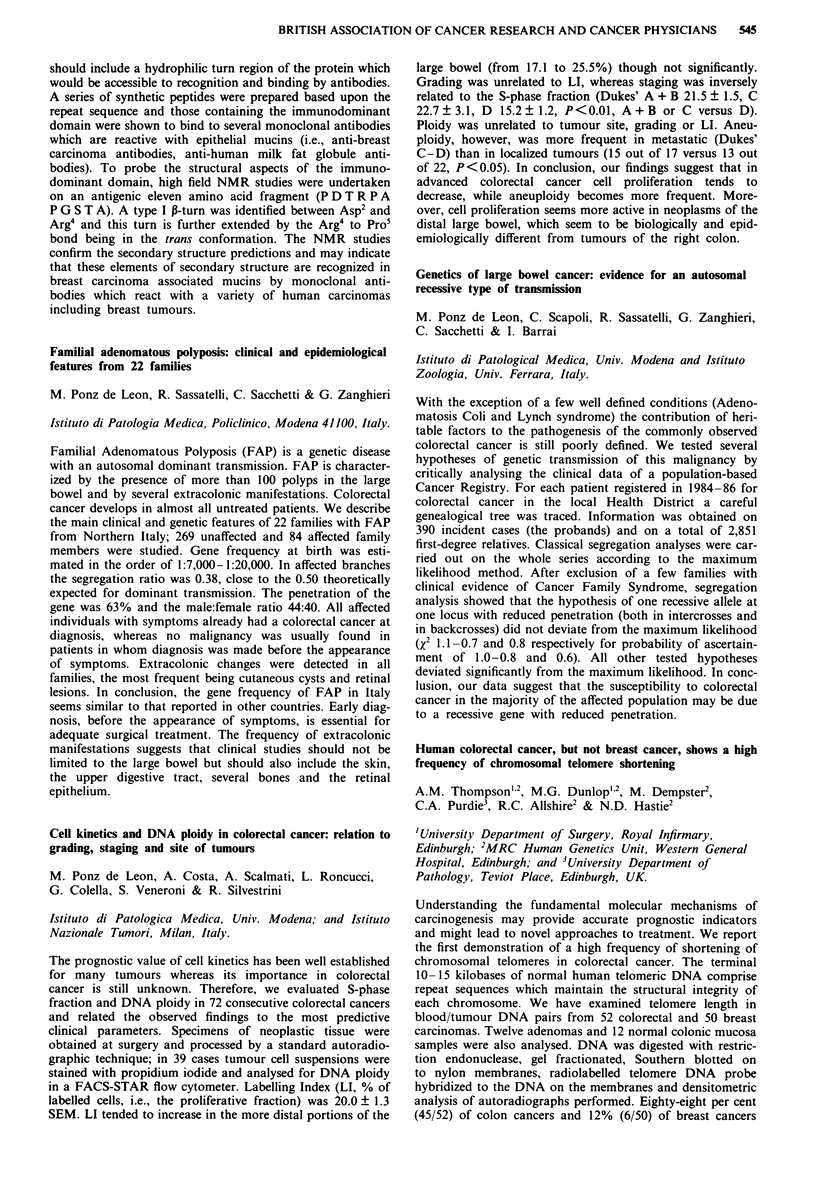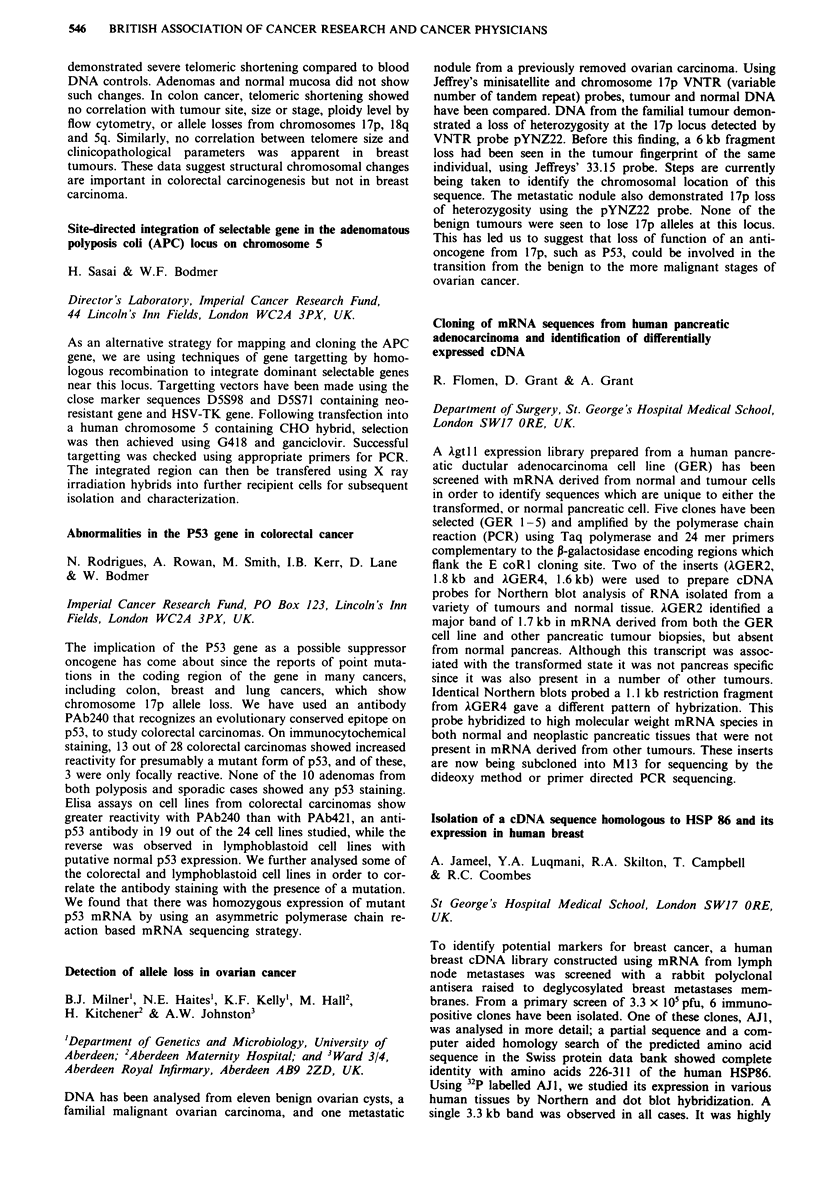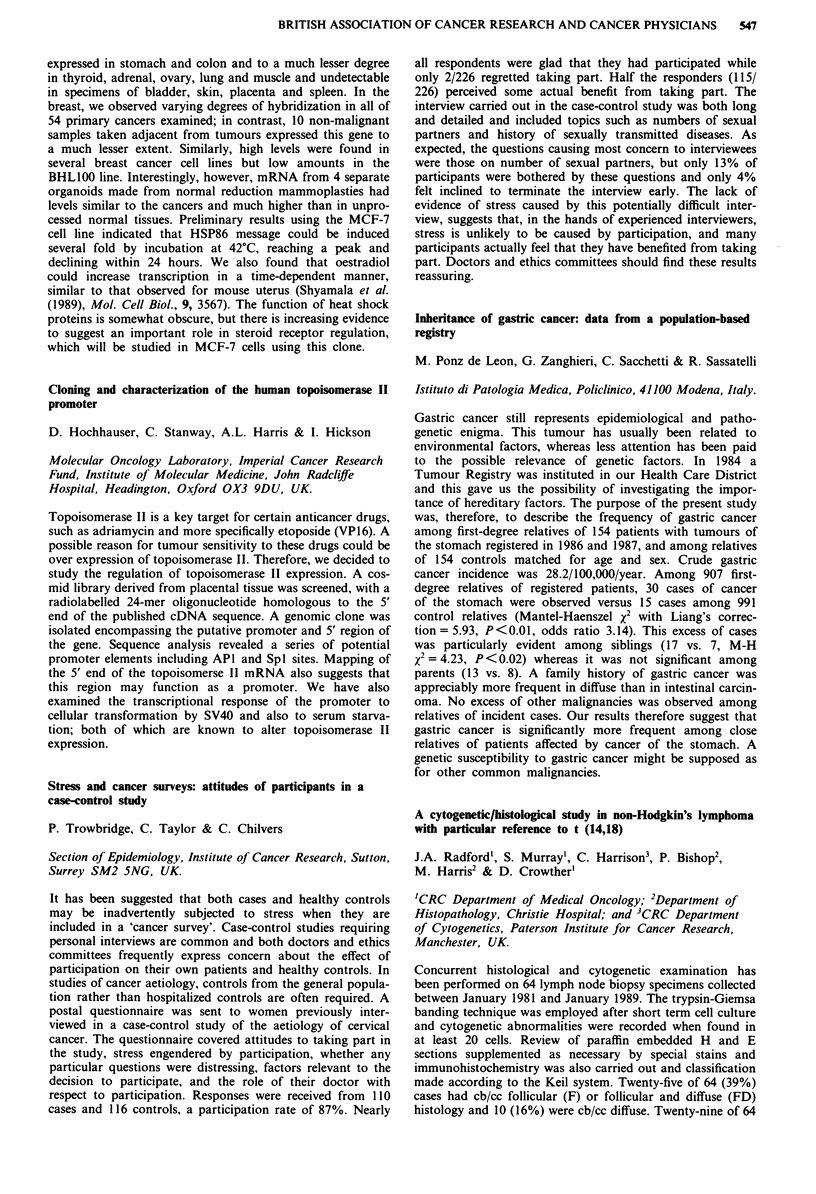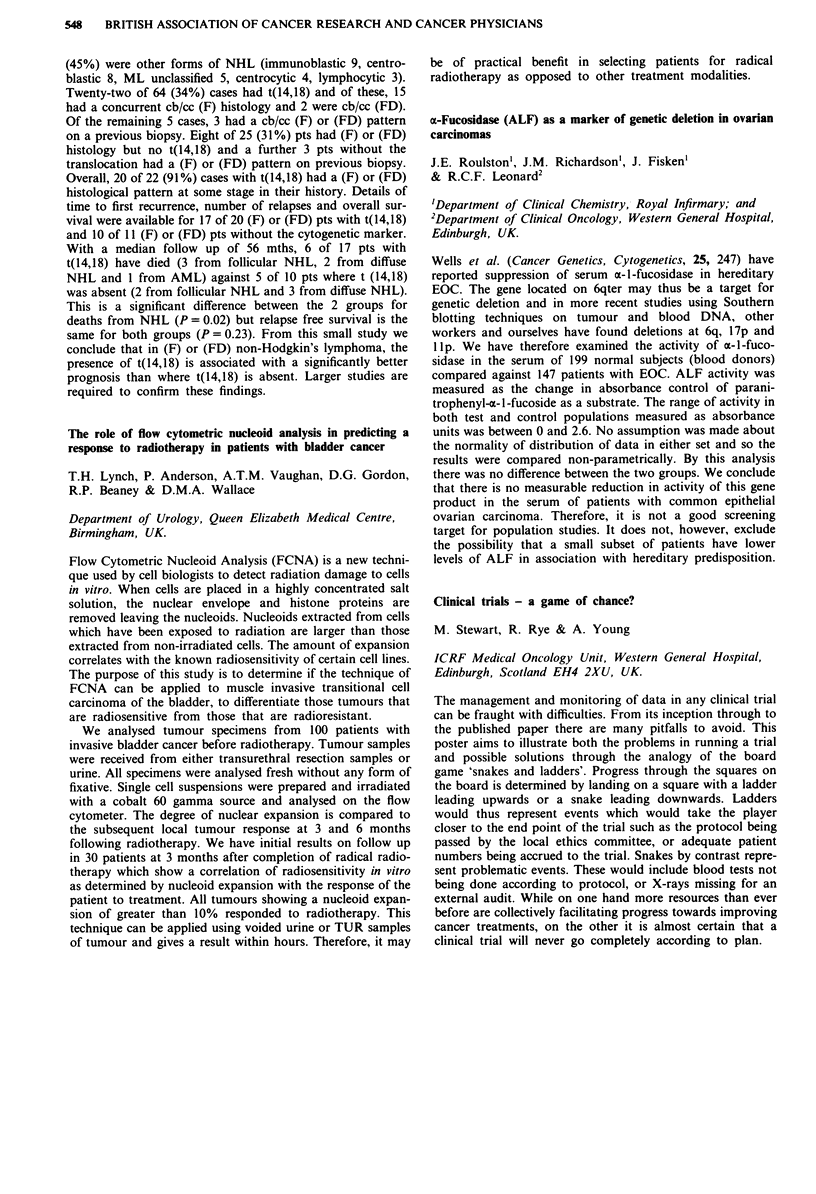# 31st annual meeting of the British Association of Cancer Research and the 5th annual meeting of the Association of Cancer Physicians. 19-22 March 1990, Sussex, UK. Abstracts.

**Published:** 1990-09

**Authors:** 


Br. J. Cancer (1990), 62, 474–548 © Macmillan Press Ltd., 1990

### 31st Annual Meeting of the British Association of Cancer Research and the 5th Annual Meeting of the Association of Cancer Physicians, 19–22 March 1990

*University of Sussex, Falmer, Sussex, UK.*

### Symposium on Tumour Suppressor Genes

### Functional analyses of candidate tumour suppressor genes

E.J. Stanbridge

*University of California, Irvine, California, USA.*

Evidence has been accumulating for loss of genetic information associated with various human malignancies. This evidence has been based upon cytogenetic analyses and restriction fragment length polymorphism (RFLP) analyses. Using these techniques, deletion of discrete regions of specific chromosomes have been detected. Human cancers have been associated with specific deletions on single chromosomes (for example, retinoblastomas) or multiple chromosomes (for example, colorectal carcinomas). These deletions have been interpreted as inactivation of tumour suppressor genes. With few exceptions (rb-1 and p53) the genes have not been cloned and linkage analysis data are not available. To provide functional evidence for unknown and putative tumour suppressor genes, we have developed techniques for single chromosome transfer. Data were presented indicating tumour suppression via monochromosome transfer. In most cases, the predicted chromosome had a tumour-suppressing effect. In others, for example, neuroblastoma, this was not the case. Furthermore, we have been able to dissociate the induction of differentiation from tumour suppression in neuroblastoma. Recent refinements of the microcell transfer technique have allowed us to transfer portions of single chromosomes, thereby, more finely mapping the region responsible for tumour suppression.

### p53 as an oncogene and a tumour suppressor gene. Different point mutations in p53 in human and murine cancer exert a common effect on the proteins conformation and function.

D.P. Lane, J. Gannon, J. Bartek, R. Greaves, A. Harris, K. Gatter & R. Iggo

*ICRF Clare Hall Laboratories, South Mimms, Potters Bar, Herts. EN6 3LD, UK.*

The human p53 gene is located on chromosome 17p. Recent studies have shown that this is a frequent site of allele loss in many human tumours including lung, colon, breast, and brain tumours. Histological staining using a panel of new anti-p53 antibodies isolated in our laboratory has shown that overexpression of the p53 protein is present in over 50% of primary lung and breast tumour biopsies. Similarly at least 30% of colon cancer samples showed high level expression of p53 protein. Work with cell lines has shown that overexpression of the p53 protein is synonymous with mutation.

Since using a new PCR based strategy, we found that six out of six breast cancer cell lines that overexpressed p53 were only expressing mutant RNA. In agreement with the results from Vogelstein's laboratory, all of the mutations were point missense mutations that altered evolutionarily conserved amino acids. These results were extended to direct sequencing of mRNA from lung biopsies. All histologically positive tumours were found to be expressing mutant p53 mRNA, whereas no mutation was found in a histologically negative tumour. These results suggest that mutation of p53 is the most common genetic event so far detected in human cancer. A new monoclonal antibody to p53 only immunoprecipitates p53 encoded by mutant genes. Using this antibody we established that the different mutations in p53 exert a common effect on the proteins conformation. The mutant proteins behave as both gain of function mutants, in that they have acquired a dominant transforming function, and loss of function mutations, in that they have lost tumour suppressor activity. One way to reconcile these findings is to suggest that the mutant proteins act directly to inactivate wild type p53 function. This leads to an attractive model of p53 mutation in human cancer. Results on the inhibition of SV40 DNA synthesis by mutant and wild type p53 provide a powerful paradigm for the normal function of p53 as a suppressor of cell growth.

### Some aspects of the genetics and biology of colorectal carcinomas

Sir Walter Bodmer

*Imperial Cancer Research Fund, UK.*

The gene for the dominantly inherited susceptibility to colon cancer, adenomatous polyposis coli (APC), has been localized to chromosome 5q21. Allele loss in tumour, compared to normal tissue, from sporadic cases of colorectal carcinomas has clearly implicated the APC gene in 40% or more of all cases. Two further clear cut examples of genetic changes involving loss or suppression of normal functions in colorectal carcinomas are for p53 on chromosome 17 and the DCC gene, described by Vogelstein, on chromosome 18.

The control of differentiation is likely to be one factor underlying such recessive genetic changes during carcinogenesis. A small proportion of human colon carcinoma derived cell lines can be induced to differentiate structurally when grown in a collagen type 1 gel. This differentiation is triggered by a cell surface collagen receptor of the integrin family. Somatic cell hybrid analysis shows that the ability to bind to collagen type 1, and so to differentiate, is controlled by a gene on chromosome 15. Hybrids which differentiate are essentially non tumorigenic in nude mice. This provides an explanation for recessive control of tumorigenesis, namely by lack of a receptor mediating differentiation induction. In agreement with these ideas, the expression of collagen receptors is often reduced in moderate and poorly differentiated colorectal adenocarcinomas. There is also evidence that one or more members of the CEA family of adhesion molecules may interact with certain integrins to mediate effective binding to collagen. The common expression of CEA in colorectal carcinomas may, therefore, be related to a disruption of this function.

Studies of the control of differentiation should eventually provide an explanation for the functional basis of the selection for recessive genetic mutations, such as for APC and other genes, during progression of colorectal carcinoma.

### 1990 Walter Hubert Lecture

### Genetic alterations underlying colorectal tumorigenesis

B. Vogelstein

*The Johns Hopkins Oncology Center, Baltimore, MD 21231, USA.*

Colorectal tumours develop through clinically and histologically defined stages which make these neoplasms an excellent system for investigating the genetic alterations responsible for tumour initiation and progression. Studies from our laboratory, and by others, have revealed the following principles underlying the development of colorectal neoplasia.

All colorectal tumours are monoclonal in composition, suggesting that neoplasia develops from the transformation of a cell(s) from within a single crypt. The presence of specific genetic alterations in early colorectal tumours additionally supports the idea that tumours of all sizes represent the clonal expansion of a single transformed cell.

The various stages of tumorigenesis appear to be the result of accumulated changes in both oncogenes and tumour-suppressor genes; the latter predominate. Mutations of at least 4-6 genes appear to be required, in general, for carcinoma formation, while smaller numbers of mutations suffice for benign tumorigenesis.

Although amplification of certain oncogenes occasionally occurs in colorectal carcinomas, K-*ras* is the only oncogene known to be involved in a large percentage of tumours. Codons 12 and 13 of the K-*ras* oncogene are frequent targets of mutations, and such mutations are found in approximately 50% of intermediate and late stage adenomas as well as in carcinomas.

DNA is consistently hypomethylated in colorectal tumours at all stages, and this represents a very early event in neoplasia. Hypomethylation has been demonstrated to lead to aberrant chromosome segregation *in vitro* and the hypomethylation found in tumours *in vivo* could lead to the loss of wild-type tumour-suppressor genes through similar segregation abnormalities.

Most of the genetic changes so far identified in colorectal tumours involve tumour suppressor genes, as inferred from allelic deletion analysis. Two chromosomal regions (17p and 18q) are lost in over 75% of colorectal carcinomas, and at least 6 additional loci (including 5q) are each lost in more than a quarter of the carcinomas.

The gene responsible for familial adenomatous polyposis has been mapped to chromosome 5q. The chromosomal region containing this gene is lost in over one-third of sporadic adenomas but is deleted infrequently in the adenomas of FAP patients.

The suppressor gene on chromosome 17p has been tentatively identified as the p53 gene through a combination of allelic deletion analysis and molecular cloning. A relatively small region of 17p was found to be consistently lost in colorectal tumours and this region contained the p53 gene. Detailed analysis of the residual p53 genes remaining in colorectal tumours showed that the residual p53 gene usually contained a missense mutation. These and other studies suggest that the wild-type p53 gene might suppress neoplastic growth, and that mutations in the p53 gene abrogate this suppression. A mutation of one p53 allele is often followed by loss of the wild-type allele, and this loss generally coincides with the transition from adenoma to carcinoma.

A small region of chromosome 18q was found to be consistently deleted in colorectal cancers, and a strategy employing chromosome walking and cross-species-hybridization eventually led to the identification of a candidate suppressor gene in the region. The gene, called DCC (for *d*eleted in *c*olorectal *c*arcinoma) was found to be expressed in many normal tissues, but its expression was reduced or absent in most colorectal carcinomas. Several examples of somatic mutations within the DCC gene were identified in colorectal cancers. Interestingly, the DCC gene has several structural characteristics which suggest it is a cell adhesion molecule. The cell adhesion mediated by DCC may be important in negatively controlling epithelial cell growth.

The genetic alterations noted above occur in a preferential order. Hypomethylation is present very early, *ras* gene mutations and chromosome 5q allelic losses generally precede 18q allelic losses, which in turn precede 17q allelic deletions. However, detailed analysis of individual tumours at different stages of development suggest that it is the total accumulation of changes, not their order with respect to one another, that is most important in determining biological behaviour.

This accumulation of changes has important practical implications. Carcinomas with greater than the median number of changes are much more likely to metastasize to distant organs than those with fewer mutations, irrespective of size or histopathological characteristics. Such information forms the basis for a prognostic test which may have clinical utility.

### Molecular basis of tumour suppression by the human retinoblastoma gene

Wen-Hwa Lee

*Department of Pathology, M-012 and Center for Molecular Genetics, School of Medicine, University of California, San Diego, La Jolla, CA 92093, USA.*

A class of cellular genes in which loss-of-function mutations are tumorigenic has been proposed. Such genes would normally act to suppress the cancer phenotype at the cellular or organism level. The gene determining susceptibility to hereditary retinoblastoma (RB) appears to operate in exactly this fashion, and is the first cancer suppressor gene to be molecularly cloned. The RB gene contains 27 exons dispersed over more than 200 kb and expresses a 4.7 kb mRNA ubiquitously. From the sequence analysis of the RB cDNA, the predicted RB protein has 928 amino acids with mol.wt. about 106 kDa. Several antibodies, prepared according to the sequence, recognized the putative RB protein (apparent m.s. 110 kDa) in normal cells, but do not detect this protein in retinoblastoma cells. The RB protein is a nuclear phosphoprotein capable of binding to DNA and to form a complex with oncoproteins of several DNA tumour viruses.

Consistent with its ubiquitous expression pattern, RB gene inactivation is not only limited to retinoblastomas. Many other cancers such as osteosarcoma, breast carcinoma, small cell lung carcinoma and prostatic carcinoma contain inactivated RB genes, suggesting that RB may act as a general cancer suppressor gene. We have introduced, through retroviral-mediated gene transfer, a cloned RB gene into retinoblastoma, osteosarcoma, prostate carcinoma and breast carcinoma cells that have inactivated endogenous RB genes. Expression of the exogenous RB gene consistently suppressed their tumorigenicity in nude mice. Each individual clone of these tumour cells with exogenous RB protein expression was non-tumorigenic in nude mice. However, after prolonged culturing some of the clones lost the expression of the RB protein and regained tumorigenic ability. This tight correlation strongly indicated that the RB gene is essential to tumorigenesis in these tumour cells.

In an attempt to address the potential cellular function of this gene, we have observed that Rb protein phosphorylation oscillates with cell-cycle and the unphosphorylated form is present predominantly in the $G_0/G_1$ phase. Furthermore, when cells were induced to differentiate only the unphosphorylated form of Rb could be detected, suggesting that RB protein was modulated through phosphorylation and may play an important role in these cellular functions. A working hypothesis is proposed to interpret how Rb participates in cell proliferation and differentiation in relation to its role in tumorigenesis.

### Transformation suppressor genes

M. Noda, H. Kitayama, S. Kanazawa, S. Murata, S. Kumar, T. Matsuzaki & Y. Ikawa

*Tsukuba Life Science Center, The Institute of Physical and Chemical Research (RIKEN), 3-1-1 Koyadai, Tsukuba, Ibaraki 305, Japan.*

To understand the molecular mechanism of malignant cell transformation, we have been isolating and characterizing suppressor mutants (flat revertants) from a v-Ki-*ras*- transformed N1H3T3 cell line, named DT. Our recent study has demonstrated that flat revertants could be isolated from DT cells after transfection of normal human fibroblast cDNA-expression library, and that this strategy is useful in detecting such genes that, when overexpressed, interfere with the transforming activity of the oncogene (we collectively call such genes as "transformation suppressor genes").

Through this method, we have isolated and molecularly characterized several biologically active cDNA species. One of the cDNAs, named K*rev*-1, was found to encode a *ras*-related protein and exhibit an activity of inducing flat revertants at frequencies of 2–5% of total transfectants when introduced into DT cells. Toward understanding the mechanisms of action of K*rev*-1 protein, we constructed a series of point mutants of K*rev*-1 cDNA as well as some chimeric genes consisting of fragments of H-*ras* and K*rev*-1 cDNAs, and tested their biological activities in DT cells as well as in HT1080 human fibrosarcoma cells harbouring the activated N-*ras* gene. Substitutions of the amino acid residues in the putative guanine nucleotide-binding regions ($Asp^{17}$ and $Asn^{116}$), in the putative effector-binding domain (residue 38), at the putative acylation site ($Cys^{181}$), and at the unique $Thr^{61}$ residue all decreased the transformation suppressor activity. On the other hand, substitutions such as $Gly^{12}$ to $Val^{12}$ and $Gln^{63}$ to $Glu^{63}$ were found to increase significantly the transformation suppressor/ tumour suppressor activity of K*rev*-1. These findings are consistent with the idea that K*rev*-1 protein is regulated like many other G-proteins by guanine triphosphate/guanine diphosphate-exchange mechanism, probably in response to certain negative growth-regulatory signals. Furthermore, study with the chimeric genes revealed that the determinants for the suppressor activity reside in the N-terminal one third of the K*rev*-1 encoded polypeptide.

The second cDNA we have characterized, named K*rev*-3 was found to encode the carboxy-terminal one third of 'tenascin', also known as cytotactin, hexabrachion, etc., including fibronectin type III repeats. Tenascin has been reported to exhibit mitogenic activities as well as cell adhesive activities. Study of the mechanism of action of the cryptic protein produced from the K*rev*-3 cDNA may yield an important clue to the understanding of the physiological functions of this extracellular matrix protein.

Our *in vitro* approach has been useful in isolating biologically interesting genes, and further studies on these and other genes and their products may yield important insights into the regulatory mechanisms of cell growth and, possibly, into the aetiology of some naturally occurring tumours as well.

### Wilms tumour; a developmental anomaly

N. Hastie

*MRC Human Genetics Unit, Western General Hospital, Crewe Road, Edinburgh EH4 2XU, UK.*

Wilms tumour (WT) is an embryonic kidney tumour which is thought to result from failure of normal mesenchymal stem cell differentiation. The tumour arises through loss of function of at least one so-called tumour suppressor(s) gene which maps to the short arm of chromosome 11.

The precise location of a WT predisposition gene is known through the association of WT aniridia (lack of an iris), genitourinary abnormalities and mental retardation in the WAGR syndrome. Individuals with this syndrome almost invariably have constitutional deletions of one chromosome 11 which always involve band 11p13. A combination of deletion mapping and analysis by pulsed field gel electrophoresis with a large number of DNA markers has led to a high resolution map of the 11p13 region so that the locations of the WT and aniridia genes are known. The gene responsible for the genital abnormalities in WAGR patients maps within the same 350 kb region as the WT gene, raising the possibility that the gonadal anomalies result from mutation or deletion of the WT gene itself.

We have been working with a candidate WT gene isolated in the laboratory of David Housman at MIT. This gene encodes a protein with four zinc fingers which is likely to play a role in transcriptional control. Consistent with a development role for the WT gene we show by *in situ* hybridization that this candidate is specifically expressed in the condensing mesenchyme and glomerular epithelium of developing metanephros and mesonephros. In addition, the gene is expressed in the genital ridge and fetal gonads, a finding which supports the notion that genital anomalies in WAGR patients result from mutation in the WT gene itself. In tumours, we find the cell types expressing the gene correspond to the cell types which express the gene during normal nephrogenesis.

### Symposium on Management of Cancer

### Screening for Cancer

J. Chamberlain

*Royal Marsden Hospital, Sutton, Surrey, UK.*

The purpose of screening is to detect presymptomatic neoplasia at a stage in its natural history at which treatment will arrest its progression. The success of screening must, therefore, be judged by a fall in mortality from the cancer in question. Although screening tests are available for many different cancers and have been shown to result in the diagnosis of a high proportion of cancers at an early stage, very few trials have assessed the impact of screening on mortality. At the present time, screening is of proved value only for breast cancer and cervix cancer. A number of well-conducted trials of screening for lung cancer have failed to show any benefit. Screening for other cancer sites, including large bowel, melanoma, ovary and stomach, is currently being investigated in research studies from which some preliminary results are available.

Even when it has been shown that screening can improve prognosis, doubt may remain about the cost-effectiveness of a public health screening programme. The physical, psychological and financial costs need to be measured and weighed against the size of benefit, for example, in years of life gained. Screening is likely to put an increased burden on diagnostic services in order to sort out people with false positive, from those with true positive, screening results.

### Analgesia for cancer pain

G.W. Hanks

*Royal Marsden Hospital, Fulham Road, London, SW3 6JJ, UK.*

Morphine is the strong opioid analgesic of choice for severe cancer pain and the use of morphine by the oral route is now being promoted around the world by the WHO. This is a relatively recent development; there was previously a widespread reluctance to use morphine in this way because of doubts about its efficacy (Rane *et al.* (1982) *Acta Anaesth. Scand. Suppl.* **74**, 97). Opinion gradually changed because of the extensive empirical experience with oral morphine in the United Kingdom. We can now attempt to explain why there were doubts about the activity of oral morphine and why morphine by this route is in fact very effective in the management of chronic cancer pain. Crucial to these explanations is the role of morphine-6-glucuronide (Hanks *et al.* (1987) *Lancet*, **2**, 723), a metabolite of morphine which is up to 45 time more potent than the parent drug in some animal models.

Alternatives to oral morphine were discussed: pethidine and methadone are best avoided; buprenorphine may have a limited role; phenazocine and epidural morphine may be indicated in patients suffering intolerable side effects from systemic morphine.

Adjuvant drugs or co-analgesics will invariably be required in conjunction with morphine. The most commonly used groups of drugs are the non-steroidal anti-inflammatories for bone pain, corticosteriods for nerve compression, or infiltration, and other pain caused by pressure from a tumour mass; anticonvulsants for stabbing dysaesthetic pain related to nerve damage and occasionally other dysaesthesiae; and muscle relaxants for muscle spasm pain.

Drugs are not, however, sufficient by themselves. Pain and suffering in the cancer patient are invariably compounded by anxiety, fear, depression, hopelessness and misunderstanding. Empathy, explanation and reassurance may have a profound pain-relieving effect. A variety of physical and other non-drug measures may also have an important part to play in the management of cancer pain.

### The potential for NMR spectroscopy in oncology

J.R. Griffiths

*The CRC Biomedical Magnetic Resonance Research Group, St George's Hospital Medical School, London SW17 ORE, UK.*

NMR spectroscopy (MRS) permits chemical analysis of living tissue without taking biopsies or administering exogenous substances. Numerous studies are in hand throughout the world to apply MRS both in the oncology clinic and the laboratory.

Potential uses of MRS in oncology fall into three main categories. The $^{31}P$ or $^{1}H$ spectra of the tumour itself give us information about metabolites such as ATP or lactate that can be used for diagnosis, prognosis, choice of therapy or monitoring therapy. Animal studies suggest that hypoxic tissue can be detected and that there are marked metabolic changes after successful therapies of various kinds. Exogenous probe substances may be administered and their spectra used to give similar information. For instance, fluorinated radiosensitizer compounds could be used to label hypoxic tissues and assist planning of radiotherapy. The spectra of anticancer drugs can be detected in tumours or in normal tissues and we may follow their activation and detoxification. Most work so far has been on 5-fluorouracil and its analogues; formation of fluoronucleotides in animal tumours has successfully predicted therapeutic response.

In practice, the application of MRS is limited by its low sensitivity. In animal studies, subcutaneous tumour of 1 $cm^3$, or less, can be observed but in patients the tumour is often much further from the surface and volumes of greater than 50 $cm^3$ usually have to be studied to obtain a spectrum in a reasonable time. In many cases this will mean that only primary tumours can be examined. Nevertheless, information obtained may guide subsequent therapy of metastatic disease.

### AIDS and malignancies

A. Dalgleish

*Medical Research Council, CRC, Watford Road, Harrow, Middlesex, UK.*

AIDS is the definition of terminal HIV infection. Kaposi's sarcoma (KS) and non-Hodgkin's lymphoma are prominent manifestations of AIDS. They are probably associated with profound immunosuppression induced by HIV and are not necessarily induced by other oncogenic agents. Two main types of lymphoma are seen and probably have different mechanisms of pathogenesis due to HIV infection. The clinical spectrum of KS is broad and the pathogenesis was reviewed in detail in the light of recent work at the NIH. Other malignancies such as Hodgkin's disease, and anal and oral squamous carcinoma have been reported in HIV infected people and their significance to HIV infection were reviewed.

### The management of primary bone tumours

R.L. Souhami

*Dept of Oncology, University College and Middlesex School of Medicine, Mortimer Street, London W1, UK.*

In large, multicentre trials the 5-year survival of operable osteosarcoma is now 60–65%. This is an improvement on the results of the early 1970s, when only 25% of cases were alive at 3 years. The major impact on survival has come from intensive combination chemotherapy which has also facilitated limb-sparing surgery which is now feasible in 70% of cases in the UK. It is probable that chemotherapy of short duration will produce as good results as the longer programmes. This, and the best use of drug combinations, is currently the subject of controlled trials. Improvements in chemotherapy are necessary for the 40% who relapse and for those who present with metastases. The management of pulmonary metastases remains a difficult problem and the criteria for metastasectomy are not clearly defined.

In Ewing's sarcoma, the main problem is the treatment of large tumours which are associated with a worse prognosis both for local relapse and pulmonary metastasis. Combinations of surgery and radiotherapy will probably be needed for optimum local control. In both Ewing's sarcoma and osteosarcoma, surgery of the axial skeletal tumours in specialized units is now more widely performed. Chemotherapy with new agents, such as ifosfamide, has not been shown to have an advantage over less intensive regimens. Randomized trials are, regrettably, seldom performed. Such trials are feasible if there is national and international collaboration.

It is now clear that high-grade spindle-cell sarcomas of bone are chemosensitive. Histological necrosis, often complete, is frequently found after pre-operative chemotherapy. The use of chemotherapy may improve survival in these tumours where surgery alone is associated with a 3-year survival of only 30%.

### 1990 Bagshawe Lecture

**Matching basic research to the management of cancer: the view from the other side of the fence**

J.A. Wyke

*CRC Beatson Laboratories, Beatson Institute for Cancer Research, Garscube Estate, Switchback Road, Bearsden, Glasgow G61 1BD, UK.*

SEE FULL PAPER IN THIS ISSUE

### Symposium on Supportive Care and the Control of Toxicity in Cancer Therapy

**Dose-response relationships in chemotherapy of malignant disease**

W. Hryniuk

*Ontario Cancer Foundation, Hamilton Regional Cancer Centre & McMaster University, Hamilton, Ontario, Canada.*

In animal model systems, outcome of chemotherapy depends upon drug dose, tumour sensitivity, and tumour burden. Animal models do not provide for reduced dosage or delay due to toxicity, and optimum treatment can be determined by treating beyond lethal toxicity. This is not possible in humans. Instead, attempts to define optimum treatment while avoiding toxicity have resulted in a plethora of schedules, combinations, and schemes to reduce doses and delay treatments. However, these schemes and schedules have obscured dose-response relationships. This becomes very important when the most toxic effect of treatment may be death from insufficient treatment.

We suggest dose-response relationships can be rediscovered and studied by reducing all to how much drug is given per unit time, as mg $m^{-2}$ weekly. This we refer to as dose intensity. Dose intensity may be calculated from intended drug doses ("projected dose intensity"), or from dose received after reductions and delays for toxicity ("received dose intensity"). To calculate received dose intensity, treatment delays are assumed equivalent arithmetically to dose reductions. For regimens containing only one drug, dose intensity may be calculated simply by disregarding the protocol schedule and expressing the treatment in the standard form: mg $m^{-2}$ weekly. For regimens containing more than one drug, dose intensity can still be calculated provided the drugs are of approximately equivalent activity. This is done by arbitrarily choosing one regimen as the standard and expressing all other regimens relative to the arbitrarily chosen standard. Dose intensity, either for single agents or combination regimens, correlates very well with outcome in various malignant diseases.

Data from animal model systems suggest that dose intensity and total dose are both determinants of antitumour effect but to different degrees; dose intensity appears to be relatively more important in treatment of faster-growing tumours while total dose may be more important in treatment of slower-growing tumours. There are insufficient data to determine whether this is also true in humans. There are no data from either model systems or the clinic which address the issue of whether dose intensity or total dose are more important according to the phase of treatment, that is, whether "induction" or "maintenance".

In order to integrate these concepts into a paradigm for cancer chemotherapy it is useful to recognize two parallel hierarchies of factors which determine outcome. Treatment outcome is primarily dependent on the sensitivity to cytotoxic drugs of individual tumour and normal cells, secondarily dependent upon the growth rate and rythm of both tumour and normal tissues, and finally dependent upon the size of tumour burden and size of the normal tissue reserves. This paradigm suggests three sequential broad treatment strategies. First, the most active least toxic drug(s) must be selected. Second, remission must be induced by an optimal combination of dose intensity and total dose of the chosen drug(s) given to a limit of toxicity specified at the outset of the investigation. Third, the residual tumour burden must be eradicated without incurring an unacceptable long-term deficit in normal tissue functioning. It is possible to specify formally the sequence of investigations required to implement these three broad treatment strategies.

### The clinical role of the haemopoietic growth factors

D. Crowther

*Department of Medical Oncology, Christie Hospital and Holt Radium Institute, Manchester M20 9BX, UK.*

Since the first publication in 1987 showing that G-CSF given by continuous intravenous infusion ameliorates the neutropenia and reduces the incidence of infection following intermittent combined chemotherapy for cancer (Bronchud *et al.*, 1987), there have been a number of reports indicating beneficial effects in this context using both G- and GM-CSF. In our first study, the period of neutropenia was significantly shortened (by a median of 80%) and the neutrophil count levels were above normal again by 14 days following chemotherapy. In view of these results, a further study was undertaken to examine the possibility of using intensive 2-weekly chemotherapy under cover of G-CSF. Treatment with doxorubicin at doses of 75, 100, 125 and 150 mg $m^{-2}$ was followed by infusion of G-CSF for 11 days. Again the neutrophil counts returned to normal within 12 to 14 days allowing the delivery of up to 3 cycles of high dose chemotherapy at intervals of 14 days. These studies demonstrated that intensive chemotherapy with dose limiting myelodepression can be given with increased frequency under cover of G-CSF. Our studies using GM-CSF have also shown that administration by continuous i.v. infusion can reduce the period of life-threatening neutropenia following high dose melphalan (120 mg $m^{-2}$) without resort to autologous bone marrow transplantation (ABMT). In this study, the period of granulocytopenia ($< 500$ g $\times 10^9 l^{-1}$) following melphalan was less than 15 days. This compares favourably with other series using high dose melphalan followed by ABMT without CSF where the duration of severe neutropenia was prolonged beyond 3 weeks. Enhanced neutrophil recovery has been demonstrated following conventional and high dose chemotherapy allowing the use of accelerated chemotherapy of higher dose intensity than would have been possible without the use of a myelopoietic growth factor.

Improvement in the neutrophil count using G- and GM-CSF has been observed in patients with marrow graft failure, bone marrow failure from a variety of causes, myelodysplastic syndrome, AIDS undergoing therapy with AZT, cyclic neutropenia, Kostman's syndrome and in patients undergoing chemotherapy for acute myelogenous leukaemia. To date, most of these studies have involved relatively few patients but major large randomized studies are underway to confirm these findings. Although enhanced platelet recovery has been observed following the use of GM-CSF, these effects have been relatively modest but early trials with IL-3 and the combined use of growth factors are showing more beneficial effects on platelet counts in patients with some forms of bone marrow failure. The administration of haemopoietic growth factors has increased the yield of peripheral blood stem cells allowing the use of these cells as rescue following ablative chemotherapy and it would appear that the combined use of growth factors including IL-3 is likely to increase the yield further.

Proteins and small peptides have been identified which are capable of inhibiting haemopoiesis and their future use in reducing bone marrow toxicity is an exciting prospect. Studies of the possible use of GM-CSF and M-CSF in enhancing host antitumour activity are only just beginning. Although continuous intravenous infusion has been accompanied by more pronounced effects than bolus intravenous or subcutaneous administration, optimal routes and schedules of administration have not been established for the various therapeutic indications. The recognition of periodic behaviour in biological systems and the introduction of new mathematics involving chaos theory is providing an explanation for some of these observations. New ways in which therapeutic benefits of a biological agent may be enhanced involve such considerations.

### The development of selective 5-$HT_3$ receptor antagonists as anti-emetic agents

G.J. Sanger

*SmithKline Beecham Pharmaceuticals Research Division, Coldharbour Road, The Pinnacles, Harlow, Essex CM19 5AD, UK.*

The original selective antagonists of the 5-hydroxytryptamine (5-HT) 5-$HT_3$ receptor (formerly the 5-HT M receptor) were developed for uses other than the control of cytotoxic-induced emesis (King & Sanger (1989), *Drugs of the Future* **14,** 875). However, the availability of such compounds enabled the link to be established between 5-$HT_3$ receptors and cytotoxicity-induced emesis (Sanger (1990) *Can. J. Physiol. Pharmacol.*, **68**, in press).

High doses of metoclopramide reduce cisplatin-evoked emesis in patients with cancer. We hypothesized that these high doses might exert an action which was different to that of the lower doses of metoclopramide, which are used to increase gastric motility and to prevent milder forms of emesis. Examination of the pharmacology of metoclopramide showed that high concentrations antagonize 5-$HT_3$ receptors (and have other actions), whereas lower concentrations enhance gut cholinergic activity, increasing gastric motility, and antagonize dopamine $D_2$ receptors, preventing mild emesis. The possibility that 5-$HT_3$ receptors were involved in the mechanism of action of high-dose metoclopramide was then strengthened by the finding that BRL 24924 antagonized cisplatin-induced emesis in ferrets. This compound is a selective stimulant of gastric motility and also an antagonist of 5-$HT_3$ receptors. Therefore, we repeated the experiments with MDL 72222, a more selective 5-$HT_3$ receptor antagonist, demonstrating for the first time that cisplatin-evoked emesis could be selectively and completely prevented (Miner & Sanger (1986), *Br. J. Pharmacol.*, **88,** 497).

Since these observations were made, several selective 5-$HT_3$ receptor antagonists have been described. Granisetron (BRL 43694) was developed as both an anti-emetic agent and as a 5-$HT_3$ receptor antagonist. Earlier compounds, such as ICS 205-930 and ondansetron (GR 38032F) were quickly switched to anti-emetic usage. By using these new drugs, several additional features emerged. First, the ferret is a good model of cytotoxicity-induced emesis in cancer patients (Sanger, 1990). Second, changes in ferret behaviour can be used as an index of nausea, and granisetron will prevent both of these events (Bermudez *et al.* (1988), *Br. J. Cancer*, **58,** 644). Third, granisetron will prevent emesis when given prophylactically, or after emesis has begun. The latter activity occurs within seconds after intravenous dosing (Bermudez *et al.*, 1988) and has also been demonstrated in cancer patients (Cassidy *et al.* (1988), *Proc. ASCO.*, **7,** 356). In the ferret experiments, the abolition of emesis was followed by a normal pattern of eating behaviour. Fourth, 5-$HT_3$ receptor antagonists are selective in their action and do not cause sedation or prevent emesis evoked by apomorphine and morphine in ferrets (Sanger, 1990) or by abnormal motion in man (Stott *et al.* (1989), *Br. J. Clin. Pharmacol.*, **27,** 147). Finally, granisetron does not interfere with the antitumour activity of cisplatin (Goddard *et al.* (1990), *Cancer Chemother. Pharmacol.*, **25,** in press), further illustrating the selectivity of anti-emetic activity for at least this compound.

5-$HT_3$ receptor antagonists may prevent cytotoxicity-evoked emesis by acting at the level of the abdominal vagus (Andrews, Rapeport & Sanger (1988), *Trends in Pharmacol. Sci.*, **9,** 334). However, the location of 5-$HT_3$ receptors within the human dorsal vagal complex (Reynolds *et al.* (1989), *Eur. J. Pharmacol.*, **174,** 127) now suggests that either terminal of the vagus could be an important site of action.

Future evaluation of selective 5-$HT_3$ receptor antagonists promises to be exciting. An important area of investigation is the role 5-$HT_3$ receptors might play in the changes in appetite evoked by the anticancer therapy or perhaps even by the cancer itself. In support of this possibility, we have shown that granisetron reduces the conditioned taste aversion evoked by radiation therapy in rats (Boyle & Sanger, (1988), *Proc. 'Nausea & Vomiting: A multidisciplinary perspective.* Ottawa, Canada, November 12–13. Abstr A6, 25). In the context of anticancer treatment, 5-$HT_3$ receptor antagonists could, therefore, exert a wider range of activities than currently envisaged.

### Review of clinical results with $5HT_3$ antagonists in controlling emesis

D. Cunningham

*Institute of Cancer Research, Royal Marsden Hospital, Downs Road, Sutton, Surrey SM2 5PT, UK.*

Nausea and vomiting are considered by patients to be the worst side-effects of chemotherapy. Conventional antiemetic agents such as dopamine-receptor antagonists, steroids and benzodiazipines are usually only partially successful in controlling these symptoms, particularly where there is a significant emetogenic challenge such as is associated with cisplatin. In conventional doses metoclopramide antagonizes the dopamine receptor but in higher, more therapeutically useful, doses it also antagonizes $5HT_3$ receptors. The development of specific $5HT_3$ receptor antagonists has resulted in significant improvement in emesis control without the unwanted side effects associated with dopamine-receptor antagonists such as extrapyramidal reactions, sedation and drowsiness.

The most widely studied drugs in this class are ondansetron, granisetron and ICS 205-930 and since the initiation of clinical studies in 1987 several thousand patients have received these compounds. They are extremely safe, well tolerated and side effects, which are infrequent, include headache and constipation. The clinical efficacy suggested by pilot studies have now been confirmed by randomized trials against conventional antiemetics. With chemotherapy regimens of low to moderate emetogenicity, complete control of emesis is possible in 80–85% of patients but with cisplatin there is still a significant proportion of patients, probably in the region of 40%, where complete control of emesis is not possible. The number of treatment failures is dependent on the dose of cisplatin administered, also the sex of the patient, such that complete control of emesis is possible in 70–75% of males but in only 35–45% of females. Randomized comparative studies have shown ondansetron and granisetron to be superior, or at least equivalent, to high dose metoclopramide, with or without dexamethasone, in the management of cisplatin-induced emesis, with predictably fewer side-effects. There have been no studies comparing the $5HT_3$ antagonists with each other but on the basis of published phase II and III studies they appear to be broadly similar. The optimum scheduling for these compounds has not yet been established; they have high receptor affinity and therefore simple pharmacodynamic modelling is inappropriate. A single intravenous dose will probably provide adequate cover for 12–24 hours and thereafter oral therapy will be given. To prevent delayed emesis the duration of therapy is likely to be 3–5 days. For treatment failures the addition of dexamethasone to ondansetron has already been shown to improve emetic control and the combination of a $5HT_3$ receptor antagonist and steroid will be the subject of randomized studies in the future.

### Frank Rose Memorial Lecture

### Cancer chemotherapy in the 1990s: achievements and prospects

B.A. Chabner

*Division of Cancer Treatment, National Cancer Institute, USA.*

Cancer chemotherapy enters the 1990s with a history of a few dramatic successes and many unfulfilled expectations. The most striking successes have been realized in uncommon tumours, such as the childhood leukaemias, choriocarcinoma and testicular cancer, while the common solid tumours have proved resistant, except in the adjuvant setting. Nonetheless, notable advances have recently occurred in the adjuvant therapy of colon cancer and breast cancer and there is now good reason to hope that our rapidly expanding knowledge of mechanisms of drug resistance and drug interactions will lead to effective, rationally designed regimens, using currently available drugs. The most important of these concepts, and the therapeutic strategies likely to result from their application, are discussed and critically evaluated. Foremost among these concepts are the modulation of 5-fluorouracil by leucovorin, PALA (N-phos-phonoacetyl-L-aspartic acid), and cisplatin; the reversal of multidrug resistance with calcium channel blockers and other complex cyclic amines; and dose intensification strategies.

In addition to considering clinical strategies for enhancing the effectiveness of chemotherapy, new strategies for discovering anticancer drugs are described. The National Cancer Institute (USA) has recently implemented a significant change in screening strategy based on the use of a panel of multiple human cell lines, against which will be tested a variety of synthetic chemicals as well as natural products derived from plants and marine animals, and recombinant biologicals. The rationale, strategy and early results of this effect were described, and alternatives, such as screening and synthesis directed at key biochemical targets and design based on molecular modelling, were considered.

Finally, the position that cancer chemotherapy will remain the primary tool for treatment of disseminated cancers but will be used in more creative regimens with radiation therapy and biological therapies was defended. Chemotherapy in the year 2000 will use many of the current drugs, but will integrate these agents with radiation therapy at the earliest stages of disease for such tumours as breast cancer, head and neck cancer and lung cancer, and will employ biological agents to kill tumour cells (monoclonal antibodies), ablate specific toxicities (colony-stimulating factors), promote sensitivity to chemotherapy (growth factors and differentiating agents), and discourage the development of drug resistance. It is my rash prediction that a high percentage of patients with non-metastatic presentations of colon, breast, head and neck, bladder and oesophageal cancer, and perhaps even malignant melanoma, will be cured by mixed-modality therapy, each element enhancing the basic chemotherapeutic regimen.

### Symposium on Novel Agents in Development

### Chemical modulation of toxicity

P.S. Schein

*US Bioscience Inc., Union Meeting Corporate Center, 920-B Harvest Drive, P.O. Box 220, Blue Bell, PA 19422, USA.*

Chemoprotection is an emerging discipline in cancer pharmacology, with two principal goals, reduction of the toxicity of conventional doses of chemotherapy and allowance for higher than usual doses to be administered safely and with improved efficacy. The protector must not concurrently interfere with the cytotoxic action on neoplastic tissue. Much effort has been directed toward reducing toxicity for a principal organ at risk: mesna-ifosfamide urothelial injury; ICRF-187-doxorubicin cardiac toxicity; sodium thiosulphate-intraperitoneal administered cisplatin renal toxicity; glutathione-cisplatin renal toxicity; ACTH (4-9) analogue-cisplatin neuropathy. Of increasing interest are the attempts to achieve systemic protection where multiple organs are subject to damage as with the use of high dose combination chemotherapy.

Diethyldithiocarbamate (DDTC) provides protection against many forms of cisplatin toxicity without inhibiting tumour response in murine models. However, in clinical trials DDTC produced treatment-limiting adverse autonomic effects, including severe diaphoresis, chest discomfort and agitation, while the life-threatening haematological toxicity of high dose carboplatin therapy was not apparently ameliorated.

Ethiofos (WR-2721) is a thiol that was selected from a series of 4400 chemicals screened for radioprotective properties because of its superior efficacy and relative safety. High tissue concentrations of the protective thiol are found in normal organs within minutes after intravenous administration, whereas tumours have a limited uptake and therefore remain vulnerable to chemotherapeutic attack. The profile that has emerged in animal studies and Phase II and III clinical trials is reduced toxicity to bone marrow, kidney, peripheral nervous system and eighth nerve after treatment with high doses of cisplatin and/or alkylating agents, with no loss of antitumour efficacy. Hospitalizations resulting from complications from intensive drug treatment are also decreased. In addition, preclinical studies have demonstrated that ethiofos can reduce the mutagenic and carcinogenic activities of radiotherapy and chemotherapy. Studies of combinations of chemoprotectors and bone marrow colony stimulatory factors are now planned as a new approach for achieving dose-intensification with chemotherapy.

### Clinical results with interferon and tumour necrosis factor

P. Selby

*Institute for Cancer Studies, University of Leeds, St James's Hospital, Leeds LS9 7TF, UK.*

Despite the great success of interferon alpha in the management of hairy cell leukaemia and its more modest success in the management of other haemopoietic cancers, the place of interferon in the management of solid tumours remains limited. In collected experience in the management of renal cancer in almost 800 patients the response rate is only 16% (95% confidence intervals, R 14–18%) and most responses are for less than one year. A detailed analysis of the relationship between dose and response does not suggest that further dose escalation will increase response rates and the route of administration does not influence outcome. No trials have been completed which show a prolongation of survival with interferon alpha although historical comparisons suggest that there may be a prolongation of survival for a few patients. Although the drug shows some activity in malignant melanoma, neuroendocrine tumours, lymphoma, ovarian cancer and carcinoid the effects are relatively trivial when interferon is used to treat advanced disease. The advent of interferon gamma and interferon beta has not resulted in improved clinical results to date.

Greater promise exists for the use of interferon alpha to maintain remissions after their induction by chemotherapy. Significant results are reported in multiple myeloma and indolent non-Hodgkin's lymphoma and trials are in progress to evaluate this approach in ovarian cancer and lung cancer. Adjuvant studies for renal cancer and malignant melanoma are also in progress and in these areas the drug remains of considerable interest.

Tumour necrosis factor was introduced enthusiastically as the result of encouraging results in experimental animal models and great public exposure. Phase I trials have been completed using a number of routes and schedules and the maximum tolerated dose in most studies is between 200–400 $\mu g\ m^{-2}$ and limiting toxicities are hypotension, hepatic enzyme changes and thrombocytopenia. At maximum tolerated doses in man the plasma concentrations are considerably less than those required to produce regression of mouse tumours. Clinical results are disappointing. Although targeted phase II studies are not yet reported in detail, cumulative unpublished experience suggests that tumour regression is uncommon and that no consistent pattern is emerging. The value of tumour necrosis factor in combination with cytotoxic drugs or other biological treatments remains to be explored.

### Is there still a future for the small molecule in developmental cancer chemotherapy?

M.F.G. Stevens

*CRC Experimental Chemotherapy Group, Pharmaceutical Sciences Institute, Aston University, Birmingham B4 7ET, UK.*

Compounds which evolved from the *in vivo* screening programmes of the 1960s (S180-based), the 1970s (L1210, P388) and 1980s (human xenografts) have, in general, failed in the clinic and programmes directed towards the development of analogues of these compounds should not have high priority.

Small molecule chloroethylating agents (nitrosoureas, imidazo-tetrazinones, chloroethyl sulphonates) are a case in point where inherent resistance of human tumours with a capacity to repair guanine O(6)-alkylations (the $Mer^+$ phenotype) cannot be overcome, even at drug doses eliciting life-threatening thrombocytopenia. The actions of small molecule 'antimetabolites', which interfere with DNA replication and cellular proliferation by inhibiting key DNA synthesizing and processing enzymes can be subverted by the tumour cell's ability to amplify genes coding for these essential proteins. Looking into the future, new generations of drugs designed to interfere with the potein products of oncogenes (growth factors, growth factor receptors) could be similarly flawed by the target cell's simple resort to amplify the appropriate gene(s). Only in the specific area of hormonal dependent tumours is it clear that new small molecular weight drugs (i.e. mol. wt. $<500$) might still make a major impact in the treatment of common disease.

There is a school of thought that subscribes to the view: 'To cure a complicated disease requires a complicated molecule'. However, clinical experience with some of the new products of biotechnology (interferons, interleukins, tumour necrosis factor, differentiation enhancers, tumour suppressor factors, monoclonal antibodies alone, or conjugated to cytotoxins) does not engender overwhelming optimism. Undeniably, these products elicit remarkably selective biological effects in specific cell types. Certain of these properties can also be promoted by small molecules, the prototype differentiating agents N-methylformamide (mol. wt. 59) and hexamethylene bisacetamide (mol. wt. 200) and the immune-stimulant bropirimine (mol. wt. 260) being cases in point. New research programmes directed to the discovery of small synthetic agents which mimic the properties of the natural 'biological response modifiers' could have exciting outcomes.

In the longer term the prevailing pharmacological revolution, driven by opportunities presented by the products of biotechnology, will be supplanted by a new era of chemical synthesis. Close encounters of the molecular kind between the new pharmacological tools and their targets rely on just a few hydrogen bond contacts, or ionic or hydrophobic interactions to trigger the biological effects; the bulk of the molecule is irrelevant except insofar as it is essential to bring these microstructures into functioning juxtaposition. Opportunities will soon emerge for the synthetic chemist to design surrogate small(ish) molecules with appropriately-positioned groups to mimic the properties of these natural molecules. If the essence of the cancer problem concerns controlling the expression of tumour-associated genes, then new generations of molecules capable of selectively recognizing specific nucleotide sequences in the major groove of DNA will be required.

**Realistic goals for the pharmaceutical industry in anticancer drug development**

R.C. Jackson

*Parke-Davis Pharmaceutical Research Division, Warner-Lambert Company, Ann Arbor, Michigan, USA.*

Continued progress in anticancer drug development will require both improved agents for the treatment of currently sensitive malignancies, and the development of new kinds of agent for those diseases that are at present almost totally refractory to chemotherapy, such as metastatic colon carcinoma, and non-small cell lung cancer. Major advances in this second category will require approaches to drug development that exploit recent advances in the basic biology and biochemistry of cancer. Areas under intensive study in the pharmaceutical industry include agents directed at oncogene products, and their associated growth factor receptors and signal transduction pathways; novel topoisomerase inhibitors; and new non-DNA-targeted compounds active against cells with the multi-drug resistant (MDR) phenotype. While macromolecular agents may be active against micrometastases, because of drug delivery problems it seems likely that small molecules will continue to be the most effective approach against established solid tumours. These ideas will be illustrated by four approaches to the development of new drugs for colon carcinoma.

A high proportion of colon tumours are intrinsically MDR-positive, they have high DNA-repair activity and a high fraction of non-cycling (Q) cells. These three properties in combination confer resistance to all existing drugs. Thus, one approach is to develop new cytotoxic agents active against targets other than DNA, active against Q-cells and able to overcome the MDR phenotype. With increasing knowledge of tumour cell growth factor requirements, the possibility arises of developing synthetic antagonists of growth factor receptors. One growth factor for colon cancer cells is gastrin, and synthetic antagonists (e.g. proglumide) are known. Newer antagonists with high specificity and nanomolar potency have been shown to inhibit growth of colon cancer cells. Another class of agents, exemplified by dinoline, have marked activity against colon carcinoma xenografts, with very low toxicity. The possibility exists that the antitumour activity of this kind of compound is mediated by effects on ion channels. Finally, kinetic studies predict the existence of novel kinds of antimetabolite that act on sites of positive feedback regulation. These compounds may switch off pathways at substoichiometric concentrations, may demonstrate "all-or-nothing" dose-response relationships, and their target pathways may "remember" being inhibited, i.e. stay switched off after inhibitor is removed.

These latter three classes of agents have more in common with central nervous system drugs than with traditional cytotoxic agents. Pharmaceutical industry laboratories, with extensive experience (and large compound collections) in CNS pharmacology are in a strong position to explore these novel approaches to selective growth regulation.

**Circulatory catabolic factors produced in cancer cachexia**

S.A. Beck, T. McDevitt & M.J. Tisdale

*Cancer Research Campaign Experimental Chemotherapy Group, Pharmaceutical Sciences Institute, Aston University, Birmingham B4 7ET, UK.*

We have studied a transplantable colon adenocarcinoma of the mouse (MAC16) as a model of human cancer cachexia. This tumour produces extensive mobilizing of body fat at small tumour burdens and without a reduction in food intake. As a model of fat mobilization, we have used murine epididymal adipocytes *in vitro*. Both FFA and glycerol release from mouse adipocytes were enhanced by MAC16 adenocarcinoma extracts, suggesting the possibility of lipolytic factors which may be responsible for the cachexia seen with this tumour model. Using DEAE cellulose and exclusion chromatography, the lipolytic factors were separable and resolved into a number of fractions. Following SG50 exclusion chromatography, the lipolytic factor(s) was resolved into three fractions of apparent molecular weights of 3, 1.5 and 0.7 kDa. Lipolytic factors of the same molecular weight and charge characteristics on DEAE cellulose were found in the serum and urine of cachectic cancer patients, but not in normal serum or urine even after acute starvation. A study of 26 cancer patients with various degrees of cachexia showed that the amount of lipolytic activity in the serum correlated ($R = 0.77$) with the amount of weight loss they had experienced. These results suggest that the lipolytic factors may be responsible for the cachexia seen in experimental animals and humans.

**Alterations in glucose metabolism in tumour-induced cachexia**

H.D. Mulligan & M.J. Tisdale

*Cancer Research Campaign Experimental Chemotherapy Group, Pharmaceutical Sciences Institute, Aston University, Birmingham B4 7ET, UK.*

Alterations in host glucose metabolism with the development of cachexia were investigated using a murine colon adenocarcinoma (MAC16). This tumour produces about 33% loss of body weight in male mice when the tumour burden represents only 2% of the total body mass. Weight loss occurs without a decrease in food and water intake. This tumour has been compared with another murine adenocarcinoma (MAC13) of the same series in which cachexia does not accompany tumour growth. Occasionally, growth of the MAC16 tumour does not result in cachexia. Non-cachectic MAC16 tumour bearing mice were therefore investigated as an internal control.

From *in vitro* studies, we have shown that the MAC16 cell line uses less glucose and produces more lactate and less $CO_2$ per cell than the MAC13 cell line. We used a double-labelling tracer technique to investigate the rate of glucose utilization *in vivo*. The rate of glucose utilization by the MAC16 tumour was significantly less than that of the MAC13 tumour ($p < 0.05$). The rate of glucose utilization by the testes, colon, spleen and kidneys of cachectic mice was lower than the corresponding organs of mice bearing the MAC13 tumour. Animals bearing the MAC16 tumour that were cachectic also had a lower rate of glucose utilization than mice bearing the

MAC16 tumour that were not cachectic. We have shown that the rate of glucose utilization by the brain is significantly less in all tumour bearing animals when compared to non-tumour bearing controls. These results suggest that cachexia, which occurs as a result of MAC16 tumour growth, involves a decrease in the rate of glucose utilization by the host suggesting that there may be a change to alternative substrates such as lipids. The effect on host tissues may be due to a defect in one or more of the enzymes involved in glucose metabolism.

**Infective complications during chemotherapy for small cell lung cancer (SCLC) in a population not subject to dose reduction after severe or life-threatening sepsis**

J.A. Radford[1], D. Ryder[2], D. Dodwell[1], A.G. Kamthan[1] & N. Thatcher[1]

[1]*CRC Dept of Medical Oncology and* [2]*Dept of Medical Statistics, Christie and Wythenshawe Hospitals, Manchester, UK.*

Following severe (WHO grade 4) or life-threatening (WHO grade 5) sepsis it is common practice to reduce doses of cytotoxic drugs in subsequent cycles of treatment. This is based on the assumption that fatal sepsis may result if full doses are continued. However, such reduction in dose intensity may adversely affect the outcome of chemotherapy. We have studied a cohort of 383 patients with SCLC who received treatment between May 1984 and June 1988 according to five consecutive chemotherapy protocols administered at the two hospitals comprising the Manchester Lung Group. None of the patients were subject to any reduction in protocol dose although in occasional cases treatment was delayed to allow full recovery from episodes of severe infection or myelosuppression. A total of 1,966 cycles of chemotherapy were administered to 383 patients and of these 54 (7%) had at least one episode of severe or life-threatening sepsis (SLTS). Seventeen (4.4%) patients died of infection; 4 deaths occurred in the group of 54 experiencing previous SLTS ((7.4%) and 13 in the 329 patients with no previous SLTS (3.9%)). Although statistically this is a highly significant difference ($P<0.005$), only 4 deaths in this series were potentially avoidable by dose reduction, the majority (13) having occurred without the prior warning of SLTS in earlier cycles of therapy. We consider that the concept of dose reduction in cancer chemotherapy should be re-examined.

**Treatment of tumour-induced hypercalcaemia (TIH) with pamidronate (APD): effect of infusion rate and scheduling**

D. Dodwell[1], A. Howell[2], & A. Morton[2]

[1]*Dept. of Medical Oncology, Christie Hospital, Wilmslow Road, Manchester M20 9BX, UK and* [2]*Dept. of Nephrology, Wellesley Hospital, Ontario, Canada.*

Although a variety of mechanisms may contribute to the pathogenesis of tumour-induced hypercalcaemia (TIH), the most important pathophysiological process is excessive bone resorption. The bisphosphonates, structural analogues of pyrophosphate, are potent osteoclast inhibitors and slow the rate of osteoclast-mediated bone resorption. They are reported to be effective in over 80% of cases of TIH. Pamidronate is one of the most active of currently available drugs, but optimum dose rates and scheduling are not yet known. We have performed three randomized studies in patients with TIH (corrected serum $Ca^{++} > 3.0$ mM) using a dose of 60 mg: to compare the results of an infusion given over one day (8 hours), two days (4 hours each) or four days (2 hours each) (30 patients); to determine the comparative efficacy of a 2 vs 4 hour infusion, (20 patients); and to compare 8 vs 24 hour infusions (25 patients). All patients were rehydrated before treatment. Seventy-five patients (20% breast cancer, 37% non- smallcell lung cancer, 16% myeloma, 27% others) are assessable to date. All but 4 patients achieved normocalcaemia after pamidronate (71/75, 95%). No patient was completely refractory to pamidronate. The mean time to achieve normocalcaemia was 5 days and there were no significant differences between treatment groups in terms of time to achieve a normal serum calcium, in the rate of fall or the nadir. The hypocalcaemic effect of pamidronate was noticeable the day after infusion, but there was a tendency for those patients having a 24-hour infusion to have a short lag phase. Urinary calcium excretion was markedly reduced with no significant differences between treatment groups. Alterations in systemic treatment and early death in some patients, despite attaining a normal serum calcium, meant that it was difficult to evaluate 'duration of normocalcaemia'. However, there appeared to be little difference between treatment groups. Median time for hypercalcaemia to recur was 23 days. Median survival of patients for whom no antitumour treatment was available was less than 3 months. Toxicity was minimal; rarely asymptomatic fever occurred after treatment. These studies have shown that pamidronate is a safe and effective drug in the treatment of TIH and that a single infusion of 60 mg over 2 or 4 hours is as effective as 24 hours or daily infusion regimens and is therefore to be preferred because of its simplicity. It is clear however that the prognosis for such patients remains poor, unless specific antitumour therapy is available.

**Dose intensity in ovarian cancer**

J.R. Hardy*[1], W.M. Hryniuk[2] & E. Wiltshaw[1]

[1]*Dept. of Gynaecologic Oncology, Royal Marsden Hospital, London SW3, UK and* [2]*Hamilton Regional Cancer Centre, Hamilton, Ontario, Canada.*

The relationship between outcome and projected dose intensity (DI) has previously been analysed for first-line chemotherapy of advanced ovarian cancer. The DI of the CHAP regimen (cyclophosphamide, hexamethylmelamine, adriamycin, cisplatin) was used as the standard, DI = 1.0 (Levin, L. & Hryniuk, W.M. (1987), *J. Clin. Oncol.*, **5**, 756). When published combination regimens were analysed, the relative DI of cisplatin correlated significantly with response and survival. The median DI of 5 courses of standard carboplatin (GFR based, equivalent to 367 mg $m^{-2}$) and/or cisplatin 100 mg $m^{-2}$ in the treatment of 96 patients with stage 3 or 4 ovarian cancer in this unit was 0.4 (range 0.38–0.42). The response rate of 55% seen with this regimen corresponds to that predicted by Levin and Hryniuk at this DI. We have therefore analysed the DI of cisplatin (50 mg $m^{-2}$) when used in combination with high dose carboplatin (920 mg $m^{-2}$) in the treatment of 18 patients with stage 4 ovarian cancer. The median received DI for this regimen is 1.05 (range 0.83–1.05). This predicts for a response rate of >90%, whereas the response rate seen was only 67%. This may reflect the inclusion of only stage IV patients in the study. Alternatively the relationship between DI and response may not be linear at high dose.

**Need nephrotoxicity limit dose intensity strategies in ovarian cancer?**

J. Hardy, S. Tan, I. Fryatt & E. Wiltshaw

*Dept. of Gynaecologic Oncology, Royal Marsden Hospital, London SW3 6JJ, UK.*

The relative lack of cross toxicity between cisplatin and carboplatin may enable dose intensity escalation not possible with either agent alone. We have evaluated the renal toxicity of both drugs when used as a single agent and in combination. The glomerular filtration rate (GFR) using $^{51}$Cr EDTA has been measured before and following treatment in patients with ovarian cancer receiving: standard dose carboplatin (CB), calculated according to GFR; AUC 6, equivalent to 300–400 mg m$^{-2}$; cisplatin (CS) 100 mg m$^{-2}$; high dose carboplatin (CB H-D), AUC 11, equivalent to 900–1000 mg m$^{-2}$; and high dose carboplatin in combination with cisplatin (CB-HD + CS) 30–50 mg m$^{-2}$. The Wilcoxon one-sided test of difference was used to calculate the median reduction in EDTA.

| Group | No. of patients | Median EDTA pre-treatment | Median EDTA post-treatment | Median reduction | |
|---|---|---|---|---|---|
| CB | 80 | 67.5 | 59.0 | −3.0 | NS |
| CS | 66 | 82.5 | 49.0 | −35.5 | $p<0.001$ |
| CB H-D | 26 | 80.5 | 66.0 | −12.5 | $p<0.001$ |
| CB H-D + CS | 24 | 88.5 | 64.0 | −15.5 | $p<0.001$ |

The greatest reduction in EDTA was seen following single agent cisplatin. High dose but not conventional dose carboplatin resulted in a significant reduction in EDTA but this has been shown to be reversible when repeated >3 months following completion of therapy. The addition of cisplatin at doses up to 50 mg m$^{-2}$ in order to increase dose intensity did not add to the nephrotoxicity of high dose carboplatin alone.

### Comparative opiate receptor affinities of morphine and its active metabolite morphine 6-glucuronide

P. Thompson[1], D. Hucks[2], L. McLoughlin[2], A. Grossman[2], L. Rees[2] & M. Slevin[1]

*Depts. of [1]Medical Oncology and [2]Chemical Endocrinology, St Bartholomew's and Homerton Hospitals, London EC1A 7BE, UK.*

Morphine-6-glucuronide (M6G) is a metabolite of morphine (M) with greater analgesic activity. It has been suggested that the $\mu$ 2 receptor may be responsible for much of the toxicity of M (Pasternak & Wood (1986), *Life Sciences*, **38**, 1889). Preliminary studies in man suggest M6G causes less respiratory depression and less nausea (unpublished data). Specific opiate ($\mu$, $\mu$1, $\mu$2 and $\partial$) receptor-binding assays have been performed to determine if this observed difference is due to differing opiate receptor subsite affinities.

The methodology of Yoburn *et al.* ((1988), *Life Sci.*, **43**, 1319) using tritiated and cold enkephalin ligands was adapted to give an $IC_{50}$ value for both compounds at each of the specific opiate receptors. Opiate receptors were prepared using brains from Wistar rats. Opiate receptor-binding assays were performed in Tris-HCl buffer (pH 7.4) at 37°C. Assays were in triplicate for cold ligand, M and M6G over the concentration range $10^{-5}$ to $10^{-9}$. All assay tubes were incubated for 5 minutes at 37°C before the addition of cold ligand, M or M6G, and for 15 minutes after. The bound ligand was separated from the free by filtration under vacuum through Whatman GF/B glass fibre filters. The filter was then subjected to scintillation counting. The $IC_{50}$ was defined as the concentration which inhibits 50% of specific binding of tritiated ligand. Results are shown in the table.

| Receptor | Morphine $IC_{50}$ (nM) ± SEM | M6G $IC_{50}$ (nM) ± SEM |
|---|---|---|
| $\mu$ | 14 ± 1 (n = 4) | 84 ± 16 (n = 4) p = 0.03 |
| $\mu$1 | 291 ± 73 (n = 4) | 219 ± 18 (n = 5) NS |
| $\mu$2 | 13 ± 2 (n = 4) | 60 ± 8 (n = 5) p = 0.02 |
| $\partial$ | 495 ± 78 (n = 4) | 240 ± 35 (n = 5) p = 0.03 |

These results suggest that M has a higher affinity for the $\mu$2 receptor than M6G and that the $\mu$2 receptor may be principally involved in mediating the toxicity of M thereby accounting for the toxicity difference between the two compounds. The increased affinity of M6G for the $\partial$ receptor may in part explain its increased analgesic potency.

### Comparison of the respiratory depression induced by morphine and its active metabolite morphine-6-glucuronide

P.I. Thompson*[1], L. John[2], J.A. Wedzicha[2] & M.L. Slevin[1]

*[1]Dept. of Medical Oncology, St Bartholomew's and Homerton Hospitals, London EC1A 7BE; and [2]Dept. of Thoracic Medicine, London Chest Hospital, London E2 9JX, UK.*

Morphine-6-glucuronide (M6G) is a metabolite of morphine (M) and has been shown to have more potent analgesic activity than M in animals (Tohuku (1971) *J. Exp. Med.*, **104**, 45). Animal studies suggest an improved therapeutic/toxic ratio. To determine if this hypothesis holds true for man, 10 normal volunteers took part in a double-blind randomized study comparing the effect of M 10 mg 70 kg$^{-1}$ i.v. with M6G 1.0, 3.3 and 5.0 mg 70 kg$^{-1}$ i.v. on respiratory function. Studies on each volunteer were carried out at least one week apart. Resting respiratory function was monitored for 10 minutes at 15 minutes before injection and at 0.25, 0.75, 1, 2, 3, and 4 hours after injection. The following respiratory parameters were assessed: minute ventilation ($V_E$), respiratory rate, tidal volume ($V_T$), expired $CO_2$ and $O_2$ concentrations ($FeCO_2$ and $FeO_2$), $PaCO_2$ levels 15 minutes before and 45 minutes after injection, and continuous transcutaneous $pCO_2$ and $pO_2$ and $O_2$ saturation. An ischaemic model of pain, using a sphygmomanometer cuff inflated to 250 mmHg for 5 minutes, was used to assess comparative analgesic potency at 30 minutes before and 0.5, 1.5, 2.5 and 3.5 hours after injection. Analgesic potency equivalence to M 10 mg 70 kg$^{-1}$ was approximately 3.3 70 kg$^{-1}$ M6G. Mean changes in respiratory parameters at 15 minutes are shown in the table. P-values compare M6G doses with M 10 mg 70 kg$^{-1}$.

| | M 10mg | M6G 1.0mg | M6G 3.3mg | M6G 5.0mg |
|---|---|---|---|---|
| Change $PaCO_2$ (mmHg) | 4.6 | −0.6 ($p<0.005$) | −0.52 ($p<0.005$) | 0.80 ($p<0.05$) |
| % Change $V_E$ | −22.2 | −2.7 ($p<0.05$) | −7.5 ($p<0.05$) | −7.7 ($p=0.09$) |
| % Change $FeCO_2$ | 5.9 | −4.6 ($p<0.01$) | −4.4 ($p<0.01$) | −7.1 ($p<0.05$) |

Nausea or vomiting was experienced by all but one subject receiving M but was not seen with M6G. All subjects experienced mild to moderate drowsiness with M compared with none for M6G. M6G is a potent analgesic and this study suggests it causes less respiratory depression than M as shown by a lack of rise in $PaCO_2$ and % change $FeCO_2$, and lesser fall in $V_E$. This difference may be explained by the lower binding affinity of M6G to the $\mu$2 opiate receptor (unpublished observations) thought to be implicated in the respiratory depression but not in the analgesia seen with M (*Life Sci.* (1986), **38**, 1889).

### High dose prochlorperazine infusion for cisplatin (P) induced emesis: double-blind randomized cross-over comparison with high dose metoclopramide

S.S. Akhtar, Z. Akhter, M.A. Bhat & G. Mohammed

*Dept. of Medical Oncology S.K.I.M.S., Post Bag 27, Srinagar, Kashmir, India 190011.*

This double-blind, randomized, cross-over study was conducted to compare the safety and efficacy of high dose prochlorperazine and dexamethasone (HDPD) with an effective and safe combination of high dose metoclopramide and dexamethasone (HDMD) in controlling P-induced emesis. All patients entered had received no prior chemotherapy (CT). CT was administered on an in-patient basis and all the patients received high dose P(100 mg m$^{-2}$) as a part of their treatment. Either prochlorperazine (40 mg loading dose as an infusion, 30 minutes before CT followed by 80 mg infusion over 8 hours) or metoclopramide (3 mg kg$^{-1}$ loading dose as an infusion, 30 minutes before CT followed by 4 mg kg$^{-1}$ infusion over 8 hours) was administered. Dexamethasone 20 mg, common to both the arms, was administered 30 minutes before CT. Of the 20 evaluable patients complete control of emesis was observed in 4(20%) with HDPD and 1(5%) with HDMD. Significantly fewer vomiting and retching episodes were observed with HDPD than with HDMD ($P<0.01$). The overall severity of nausea was significantly less with HDPD ($P<0.05$). There was no significant difference in the incidence of adverse reactions and the treatment was well tolerated. We conclude that HDPD combination is a well tolerated and more potent antiemetic combination for P-induced emesis.

### An increase in subclinical follicular lymphoma (FL) can be detected by the polymerase chain reaction (PCR) and may be the best predictor of clinical relapse

C.G.A. Price, S. Grant, E.L. Dorey, F.E. Cotter, B.D. Young, A.Z.S. Rohatiner & T.A. Lister

*ICRF Dept. of Medical Oncology, St. Bartholomew's Hospital, London EC1, UK.*

PCR has been used to examine peripheral blood (PB) and/or bone marrow (BM) for the presence of t(14;18) cells in patients at low risk of recurrence of FL. The following results were obtained: 5/8 patients with stage I disease (after conventional staging investigations, including BM examination) had subclinical BM infiltration at presentation; and 3 patients had circulating t(14;18) cells despite being in continuous clinical remission for 10, 11 and 15 years respectively. These findings raise general questions about the prognostic value of small numbers of t(14;18) cells detected during remission of FL. Of greater importance may be quantitative information about the level of infiltration within the range exclusively detected by PCR, i.e. approx. 1 cell in $10^6$ to 1 cell in 100. We have used a quantitative adaptation of PCR, which incorporates a limiting dilution assay, to compare levels of infiltration between patients and in sequential samples from the same patients in follow up. The results in a group of patients who went on to receive intensive chemoradiotherapy supported by autologous transplantation (ABMT) after achieving complete remission from recurrent FL are illustrative: (i) 10 patients with t(14;18) +ve FL all had evidence of infiltration in harvested BM although this was morphologically and phenotypically normal. The level ranged from 1 cell in $10^6$ to 5%; (ii) one patient had increasing subclinical infiltration of BM detected in annual 'surveillance' samples before relapse 2½ years after ABMT; (iii) 3 patients who remain in remission >18 months after ABMT have circulating t(14;18) cells which have increased in number at least 10-fold in one year. Further follow up is required, but it seems possible that such quantitative evaluation will be more useful in predicting clinical outcome than mere demonstration of PCR positivity.

### Disease assessment with the polymerase chain reaction (PCR) in follicular small cleaved cell lymphoma

T. Hickish[1], M. Soukop[2], T.J. McElwain[1] & D. Cunningham[1]

*[1]The Royal Marsden Hospital, Sutton, Surrey; and [2]Glasgow Royal Infirmary, Scotland, UK.*

A characteristic in 80% of follicular small cleaved cell lymphomas (FSCCL) is the 14:18 translocation which juxtaposes the bcl2 gene on chromosome 18 to 5′ of one of immunoglobulin junctional exons ($J_H$) of the heavy chain locus. The breakpoint in bcl2 may occur through the major breakpoint region (MBR), the minor cluster region (MCR) or rarely 5′ to the bcl2 gene. The resulting DNA sequence is unique to the malignant clone and can be used as a template for the PCR.

Currently we are evaluating the role of PCR in monitoring disease status in FSCCL. PCR is performed using a bcl2-$J_H$ concensus sequence oligonucleotide primer pair. For one pair the bcl2 primer is directed approximately 200 bp upstream compared to that of another pair (220 bp for the MBR and 150 bp for the MCR). Reaction products are run on an agarose gel stained with ethidium bromide and visualised under UV light. A fragment is authentic, that is, is due to PCR across the 14;18 breakpoint, if between reactions its size difference corresponds to the difference in the priming positions of the bcl2 directed primers. Our system enables PCR across 1.3 Kb of both MBR and MCR and allows for the detection of the majority of bcl2 rearrangements.

Results: (sensitivity 1 in $10^5$ cell – determined using a cell line (SU-DHL4) carrying the t(14:18))
Tumours: 7/14 MBR +ve
3/14 MCR +ve
4/14 no rearrangement detected
Marrows: 7/14 PCR +ve
PCR +ve/histology −ve = 2
PCR = ve/histology +ve = 0
Blood: 2/19 PCR +ve in patients in relapse

These data show PCR is able to upgrade the marrow staging in a proportion of patients (14%). Furthermore PCR of blood may be of value in providing additional staging information. In this series the instance of bcl2 rearrangements is lower than reported by others. We are exploring other targets for PCR which may be more generally applicable to B cell malignancies.

### The relationship between C-erbB-2 expression, S-phase fraction (SPF) and clinical outcome in breast cancer

S.M. O'Reilly, D.M. Barnes, R.S. Camplejohn, R.R. Millis, J. Bartkova, R.D. Rubens & M.A. Richards

*ICRF Clinical Oncology Unit, Guy's & Richard Dimbleby Dept for Cancer Research, St Thomas' Hospitals, London, UK.*

Overexpression of the c-erbB-2 oncogene has been associated with a shorter relapse-free survival (RFS) and survival (S) in breast cancer patients. To test whether the adverse prognosis associated with gene expression is due to more rapid proliferation of c-erbB-2 positive tumours, the relationship between c-erbB-2 expression (assessed immunohistochemically), SPF (measured by flow cytometry) and prognosis was analysed for 172 women with primary breast cancer. c-erbB-2 staining was independent of tumour size, nodal status, tumour grade and DNA ploidy but was more common in ER negative tumours ($P=0.02$) and PgR-ve tumours ($P=0.03$). Only a weak correlation between c-erbB-2 staining and SPF was observed ($r=0.18$). For women with node negative disease, c-erbB-2 staining did not significantly influence either RFS or S. In contrast, women with node positive disease whose tumours were scored as c-erbB-2 positive had a significantly shorter RFS ($P=0.006$) and S ($P=0.02$) than their c-erbB-2 negative counterparts. Multivariate analysis showed that c-erbB-2 staining and SPF gave independent information on RFS for women with node positive disease. We conclude that the adverse prognosis associated with c-erbB-2 expression is not solely attributable to more rapid proliferation of the primary tumour as measured by SPF.

### Prognostic significance of C-erbB-2 oncoprotein expression in breast carcinomas

A.H. McCann, P.A. Dervan, M. Codd, W. Gullick & D.N. Carney

*Mater Hospital, Dublin & Hammersmith Hospital, London, UK.*

Using the 21N polyclonal antibody, we have immunohistochemically stained 314 primary breast carcinomas to identify tumours overexpressing the C-erbB-2 oncoprotein. Positive membrane staining was present in 17% (n = 53) of these carcinomas. Using the $\chi^2$ test C-erbB-2 positive tumours were significantly associated with poorer grade ($P < 0.01$) and with a poorer degree of differentiation ($P < 0.03$) but not with any other prognostic factors investigated. C-erbB-2 positive patients had a shorter disease free interval ($P < 0.001$) and overall survival ($P < 0.001$). Stratifying patients on the basis of ER status indicated that C-erbB-2 + /ER − patients had an overall worse prognosis than the other subgroups ($P < 0.001$). Over-expression of this oncoprotein also predicted for a worse prognosis in patients with axillary node metastases ($P < 0.001$) and in patients who had a grade II tumour ($P < 0.001$). We conclude that the C-erbB-2 oncoprotein may be a valuable predictor of disease outcome in defined subsets of patients.

### Chimeric human/mouse bispecific monoclonal antibodies

S. Songsivilai, P.M. Clissold & P.J. Lachmann

*Molecular Immunopathology Unit, MRC Centre, Cambridge CB2 2QH, UK.*

The production of murine bispecific antibodies (BsAbs) has concentrated mainly on two methods: fusion of two hybridomas or hybridomas and immune cells; and chemical linking of two antibody molecules or their derivatives. For *in vivo* immunotherapy, human immunoglobulins would be preferred since the rodent monoclonal antibodies induce an anti-globulin response. In a recent development, murine antibodies have been genetically modified by replacing the constant regions with their human counterparts. These chimeric human antibodies have been shown to be less immunogenic than rodent antibodies.

We describe a new strategy for producing bispecific antibodies by introducing two sets of chimeric human/mouse immunoglobulin genes with two different specificities into murine myeloma cells. Variable regions of the heavy and light chain immunoglobulin genes of a murine hybridoma secreting an IgG1,k antibody to hepatitis B surface antigen (HBsAg) were amplified by the polymerase chain reaction (Orlandi *et al.* (1989) *Proc. Natl Acad. Sci. USA*, **86**, 3833), and cloned into expression plasmids containing human constant region genes, gamma 1 and kappa, respectively. These plasmids were co-transfected with the expression plasmids containing chimeric human/mouse immunoglobulin genes for anti-NIP specificity (Bruggemann *et al.* (1987), *J. Exp. Med.*, **166**, 1351), into a murine myeloma cell line. Bispecific anti-ABsAg-anti-NIP antibodies were identified in the cell culture supernatants. This approach can be used to produce bispecific antibodies for *in vivo* immunodiagnosis and therapy, and will enable BsAbs to be made, that cannot be produced by conventional techniques.

### Ricin toxin a chain cytotoxicity against tumour cells mediated by a bispecific monoclonal antibody, and its potentiation by ricin toxin B chain

M.J. Embleton, M.V. Pimm, R.A. Robins, R.W. Baldwin, A. Charleston & A. Markham

*Cancer Research Campaign Laboratories, University of Nottingham, University Park, Nottingham NG7 2RD, UK.*

A bispecific monoclonal antibody recognizing both carcinoembryonic antigen (CEA) and ricin toxin A chain (RTA) was investigated for its ability to target RTA to CEA-expressing tumour cells *in vitro*. The antibody, 636, induced highly significant cytotoxicity against gastric carcinoma line MKN45 in the presence of recombinant or minimally glycosylated RTA used at a concentration below that known to be intrinsically toxic. Anti-CEA and anti-RTA monospecific antibodies did not mediate increased RTA cytotoxicity, and 636 was not inhibitory in the absence of RTA. Cytotoxicity was dependent on 636 antibody concentration and was selective for CEA-expressing cells; it did not occur with CEA-negative cells. The addition of ricin toxin B chain (RTB) markedly potentiated cytotoxicity in the presence of both RTA and 636. It could also potentiate the effect of RTA alone, but was much less efficient than when 636 antibody was included. The bispecific antibody also substantially restored the cytotoxic activity of whole ricin in the presence of galactose at a concentration which blocked binding of the toxin to target cells. It was concluded that the 636 bispecific antibody was highly effective in targeting the toxic moiety of the molecule to CEA-expressing cells, and did not interfere with the ability of the B chain to facilitate cellular incorporation.

### Endocytosis of immunotoxin H65-RTA in relation to its cytotoxicity for CD5-bearing target cells

V.S. Byers[1,2], I.Z.A. Pawluczyk[1], D. Fishwild[2], S. Carroll[2], M.R. Price[1], R.A. Robins[1], A. Clarke[1] & R.W. Baldwin[1]

[1]*Cancer Research Campaign Laboratories, University of Nottingham, Nottingham NG7 2RD, UK and* [2]*XOMA Corporation, Berkeley, CA, USA.*

Monoclonal antibody H65 reacts with an epitope on the CD5 molecule expressed upon the majority of human T lymphocytes. An immunotoxin constructed by conjugating this antibody to the A-chain subunit of ricin toxin (RTA) is specifically cytotoxic *in vitro* for normal and leukaemic T cells and has proved efficacious for the treatment of T cell mediated diseases. This includes suppression of graft versus host disease in bone marrow transplantation and treatment of rheumatoid arthritis. Analysis of the *in vitro* cytotoxicity of H65-RTA for normal and malignant T cells has demonstrated different pathways of immunotoxin action. In the initial phase (2 to 3 hours) of target cell exposure to immunotoxin, cytotoxicity is greatly enhanced by potentiators, whereas, following further incubation cytotoxicity is effected without the requirement for potentiators. The relationship between immunotoxin H65-RTA endocytosis and target cell cytotoxicity has been investigated to provide a clearer understanding of the influence of potentiators. Under the conditions selected, the amount of cell bound H65-RTA endocytosed by MOLT-4 cells over a 60-minute incubation period was determined to be in the region of 20 to 35%. This process was sensitive to acidification of the cytosol by treatment with ammonium chloride and regulation of the $Na^+/H^+$ exchanger with amiloride. Inhibition of endocytosis of H65-RTA by cytosol acidification is consistent with uptake being by receptor mediated endocytosis. Internalised immunotoxin proceeds to be rapidly transferred to the lysosomes when degradation processes may render it non-functional. This is consistent with the finding that H65-RTA

cytotoxicity for MOLT-4 cells is markedly enhanced by the presence of ammonium chloride during the initial period of immunotoxin endocytosis (Byers *et al.*, in preparation) since this treatment is considered to slow lysosomal trafficking. Although receptor mediated endocytosis appears to be a major pathway for entry of H65-RTA into target cells, some immunotoxin is also endocytosed by other routes not inhibited by cytosol acidification. This is viewed as the component active following prolonged exposure of target cells to immunotoxin.

**New monoclonal antibodies recognizing colorectal tumour associated epitopes derived by stimulation of idiotypic networks**

L.G. Durrant, E. Jacobs, M.R. Price, R.A. Robins, E.B. Austin & R.W. Baldwin

*Cancer Research Campaign Laboratories, University of Nottingham, Nottingham NG7 2RD, UK.*

Stimulation of the host idiotypic network to produce antibodies which recognize autologous tumour is a new approach for the therapy of colorectal cancer. The monoclonal antibody C14 is an IgM antibody which recognizes the Y haptenic blood group antigen, which is a very useful colorectal tumour associated epitope. In a search for an IgG variant of C14, for new useful tumour associated epitopes and to investigate the potential network that would be stimulated by an anti-C14 idiotypic antisera we have used the C14 monoclonal antibody to stimulate idiotypic networks.

Rats were immunized with this monoclonal antibody and the serum was purified by removal of anti-mouse antibodies on an irrelevant mouse IgM column and then the anti-idiotypic antibodies were bound and eluted from C14 monoclonal antibody. The resultant antiserum was highly specific for C14 monoclonal antibody and inhibited binding of C14 to Y hapten. This antiserum was used to induce mice to produce anti-anti-idiotypic antibodies to the C14 monoclonal antibody some of which should recognize the Y hapten (this is highly expressed on the C14 antigen purified from saliva). However, after 4 injections, although a weak anti-C14 antigen response could be detected, the predominant response was the induction of mouse anti-rat antibodies. To boost the specific response the mice were then given C14 antigen. High titres of anti-C14 antigen antibodies were induced. Following cell fusion 5 new monoclonal antibodies recognizing C14 antibody have been derived. One of these antibodies binds to Y hapten. All of the monoclonal antibodies preferentially bind to colorectal tumour rather than normal tissue and they recognize a cell surface and a secreted antigen. They all recognize related epitopes as determined by competition and bridging assays and they all share idiotypic determinants as recognized by the purified rat anti-C14 idiotypic anti-sera. Stimulation of idiotypic networks not only can lead to the generation of new monoclonal antibodies recognizing tumour associated epitopes, but also they allow characterization of the potential networks that can be stimulated by idiotypic immunotherapy.

**Antibody directed enzyme prodrug therapy (ADEPT) in human tumour xenograft models**

S.K. Sharma[1], K.D. Bagshawe[1], G.T. Rogers[1], P.J. Burke[1], C.J. Springer[1], P. Antoniw[1], R.W. Boden[1], F. Searle[1], R. Melton[2] & R. Sherwood[2]

*[1]CRC Labs, Med. Oncology, Charing Cross Hospital, London W6 and [2]PHLS, CAMR, Porton, Hants., UK.*

The targeting of enzymes to human tumours where they can convert relatively harmless prodrugs into potent cytotoxic alkylating agents has been established in a human choriocarcinoma (CC3) and a human colon adenocarcinoma xenograft (LS174T). Our preliminary studies have been carried out with carboxypeptidase G2 (CPG2) targeted to either CC3 or to LS174T xenografts after linking to F(ab')2 fragments of anti-hCG or anti-CEA. Optimal localization occurred by 24 hours. In the CC3 model, clearance of enzyme activity from plasma occurred within 72 hours allowing administration of the prodrug and elimination of the tumour. In the LS174T model, enzyme activity persisted in blood which resulted in conversion of prodrug with lethal effects. A safe level of enzyme in blood was reached only after six days when efficiency of targeting was impaired. Injection of prodrug under these conditions did not cause significant growth delay. Reproducible growth delay was, however, obtained when a galactosylated monoclonal antibody (SB43), raised against CPG2, was injected 19 hours after the conjugate. SB43 inactivated residual enzyme activity in blood and accelerated clearance without diminishing localized enzyme, enabling the prodrug to be given within 24 hours without toxicity. Use of SB43 allows the cycle of conjugate and prodrug to be repeated to achieve an enhanced therapeutic effect.

**Phase I/II study of chimeric B72.3 antibody in radioimmunotherapy of colorectal carcinoma**

R.H.J. Begent, J.A. Ledermann, K.D. Bagshawe, A.J. Green, A.M.B. Kelly, D. Lane, D.S. Secher, M.R. Dewji & T.S. Baker

*Cancer Research Campaign Laboratories, Charing Cross Hospital, London and Celltech Ltd., Slough, UK.*

The toxicity, immunogenicity and distribution of a chimeric antibody with mouse B72.3 variable regions and human IgG4 constant regions (Whittle *et al.* (1987), *Prot. Eng.*, **1**, 499) has been investigated in patients with colorectal carcinoma. Six patients have received 10–20 mg of the antibody labelled with 20–50 mCi $^{131}I$. Treatment was repeated after 3–4 weeks provided that no human antimouse $IgG_1$ nor human antichimeric antibody, was found in the patients' serum. One to three (mean two) treatments have been given and two patients remain eligible for further treatment. One patient produced human antichimeric antibody and human antimouse antibody after the first treatment. No anti-antibody response has been found in the other five patients. No toxicity attributable to the treatment was seen. Serial measurements of activity in tumour, blood, liver and lung were made using single photon emission tomography with scatter correction (Green *et al.* (1990) *Eur. J. Nucl. Med.* in press). Mean beta half-life of clearance of antibody from blood was 116 hours (range 37–166), from tumour 226 hours (range 162–256), from liver 155 hours (range 141–173) and from lung 166 hours (range 135–182). Antibody localization was seen in tumours where radioactivity was retained longer than in other tissues. Repeated therapy with chimeric B72.3 antibody is feasible in some patients.

**The role of degradable starch microsphere (DSM) in antibody targeted therapy for hepatic metastases from colorectal cancer: a preliminary study**

A. Jewkes, S. Darby, M. Winslet, W.H. Allum & J.W. Fielding

*Academic Dept. of Surgery, Queen Elizabeth Hospital, Birmingham, UK.*

Hepatic metastases occur in 30–50% of patients and $<10\%$ are resectable. Treatment by chemotherapy, or radiolabelled monoclonal antibody (MAb), is controversial. Even after regional perfusion, MAb is taken up in minute proportions requiring lethal body doses for therapy. Temporary cessation of hepatic perfusion by DSM with synchronous regional perfusion may, however, increase tumour MAb uptake and minimise body irradiation.

Eleven patients (mean age 60 [39–80] years) with hepatic metastases (replacement = 45% [30–55%] from a gastro-intestinal primary [colorectal = 8, gastric = 3]) underwent CT scanning, liver biopsy and hepatic angiography after informed consent. DSM (300 mg) was then administered ± anti CEA MAb L11-285-14 via the hepatic artery. Biodistribution was monitored with a Spherex Monitoring System. Angiography failed in 4 patients. In 7, after DSM alone, the hepatic passing fraction was reduced to 66.3 ± 11.5%. After DSM + whole antibody MAb (200 μg) (n = 3), there was no hepatic antibody retention after 3 minutes. After DMA + antibody fraction (Fab 300 mg) (n = 4), a median of 8% (0–27%) of injected dose was retained for a minimum of 3 (3–20) minutes with a subsequent plateau.

Co-administration of DSM with whole antibody is unlikely to increase intrahepatic MAb targeting. In conjunction with $FAb_2$ fragments, however, DSM may reduce monoclonal washout and have a role in the targeting of fractionated MAb therapy for hepatic metastases.

### Properties of c-*myc* methylphosphonodiester/phosphodiester chimeric antisense oligonucleotides

D.M. Tidd, D.G. Spiller, C.C. Goodwin & H.M. Warenius

*CRC Dept of Radiation Oncology, University of Liverpool, Clatterbridge Hospital, Wirral, L63 4JY, UK.*

A normal phosphodiester oligodeoxynucleotide 15-mer complementary in sequence to the first 5 codons of the c-*myc* gene has been reported to inhibit MYC protein synthesis in HL60 cells and mitogen stimulated lymphocytes when presented exogenously in culture media. However, several UK laboratories, including our own, have been unable to reproduce these results and we have ascribed this failure, at least in part, to the rapid degradation of the oligonucleotide by serum nucleases, predominantly a 3′-phosphodiesterase, present in the culture media. Methylphosphonate oligonucleotide analogues are resistant to nucleases but are poorly soluble, exhibit weak hybridization affinity and are unable to activate degradation of target RNA by ribonuclease-H. Progressive replacement of terminal phosphodiester linkages by methylphosphonates in the *myc* antisense 15-mer resulted in stepwise reductions in the melting temperature (Tm) of hybrids formed with a synthetic target RNA from 54°C to 38°C. However, the chimeric oligonucleotide with three methylphosphonodiester linkages at each end was freely soluble, exhibited reasonable hybridization efficiency (Tm = 48°C) and being exonuclease–resistant was considerably more stable in culture media than the all-phosphodiester molecule. In addition, the chimeric oligonucleotides retained the capacity to direct degradation of target RNA by ribonuclease-H. We conclude that by combining the desirable properties of both structures, chimeric oligonucleotides may offer some advantage over either type of oligonucleotide alone. However, considerably more work is required to define the parameters which govern the efficacy of antisense oligonucleotides in intact cells.

### Single step transformation of human epithelial cells by mutant H-*ras* oncogene

D. Wynford-Thomas[1], J. Bond[1], S. Staddon[2] & N.R. Lemoine[2]

*[1]CRC Thyroid Tumour Biology Group, UWCM, Cardiff; and [2]ICRF Molecular Oncology Group, Hammersmith Hospital, London, UK.*

Although there is overwhelming correlative evidence for a role of *ras* oncogene mutation in human tumorigenesis, experimental proof has been surprisingly problematic. In human, in contrast to rodent, cells, there has been striking failure to demonstrate transforming effects of *ras in vitro*, despite its apparent involvement at an early stage in many tumours such as colon and thyroid. Using the thyroid as a model, we have now re-examined this question using amphotropic retroviral vectors. Primary epithelial (follicular) cell cultures obtained from normal human thyroid were infected with medium from the following amphotropic retroviral producer cells: 1) psi-CRIP-DOEJ, containing the human H-*ras* val 12 mutant driven by a MoMuLV LTR; 2) psi-CRIP-MTT24, containing the same gene driven by a zinc-inducible metallothionein promoter; and 3) psi-CRIP-RASβGAL, containing viral H-*ras*. One to two weeks after infection, rapidly-growing colonies were visible against the quiescent normal background epithelium (~50 per dish of $10^5$ infected). Two distinct morphologies were seen: a) a typical cobblestone epithelial pattern; b) fusiform/strap-like cells not evident in the normal culture. Representative clones of both types showed positive immunostaining for epithelial cytokeratins 8 and 18, and for the thyroid-specific protein thyroglobin. Expression of *ras* protein, assessed immunocytochemically and by Western blotting using Ab Y13-259, was increased relative to the normal epithelium. Preliminary data suggest a high degree of anchorage independence (P.E. in methocel ~20%). Clonal growth ceased at around 20 p.d.; no immortalized derivatives have been observed. We conclude that expression of mutant c- or v-H-*ras* can induce partial transformation of a differentiated normal human epithelial cell while preserving tissue-specific functions.

### Multiple events in tumour development and progression in colorectal carcinogenesis

C. Paraskeva[1], S. Harper[1], A. Hague[1], K. Audcent[1], R.A. Mountford[2], D.C.C. Bartolo[3] & A.C. Williams[1]

*[1]Dept. Pathology, The Medical School, University Walk, Bristol BS8 1TD; and Depts. [2]Medicine & [3]Surgery, Bristol Royal Infirmary, Bristol BS2 8HW, UK.*

To understand the function of tumour suppressor genes it is important to have suitable model cell culture systems for the re-introduction of these genes into cells whether by transfection, infection or microcell fusion and to know at which stage (precancer or cancer) in the multistage process of carcinogenesis these genes act. We have isolated a series of cell lines from colorectal adenoma, premalignant tumours sometimes called polyps, and carcinomas from familial adenomatous polyposis and sporadic patients (Paraskeva *et al.* (1984), *Int. J. Cancer*, **34**, 49; Paraskeva *et al.* (1989), *Cancer Res.*, **49**, 1282). Analysis of these cell lines indicated that chromosome 1 may be involved in *in vitro* immortalization. Monosomy for chromosomes 17 and 18 are important and central steps in colorectal carcinogenesis but are insufficient for the tumorigenic phenotype. Potential biological markers to study tumour progression and the possible role of these suppressor genes include: aneuploidy, *in vitro* immortalization, abnormal differentiation, clonogenicity, *ras* gene activation, production of proteinases and tumorigenicity. We have used these markers in experiments to transform a diploid premalignant adenoma cell line through a series of sequential steps to a tumorigenic phenotype. This is the first experimental evidence for the adenoma carcinoma sequence and provides an *in vitro* model to study multistage carcinogenesis. In collaboration with Eric Stanbridge we are reintroducing by microcell fusion back into precancer and cancer cell lines chromosomes implicated in colorectal carcinogenesis and examining the cells for the suppression of the transformed phenotype.

### Clonal composition of primary tumours and their metastases studied by genetic tagging with a neomycin resistance gene B

F. Moffett, D.F. Badan, J.B. Taggart & D. Tarin

*Nuffield Department of Pathology, Oxford University, John Radcliffe Hospital, Headington, Oxford OX3 3DU, UK.*

When eukaryotic cells are transfected, the site of integration of the donor DNA into the genome is random, unless special steps are taken to target its insertion. Previous work has indicated that integration patterns for any transfectant, visualized as a specific arrangement of bands by probing Southern blots of its DNA, are characteristic and stable (Talmadge *et al.* (1987), *Invasion Metastasis*, **7**, 197). Using DNA integration patterns to tag cells is a potentially powerful technique in tumour biology to study the behaviour of cell populations. The present work was undertaken to re-examine questions related to the clonal composition of tumours during their growth and spontaneous metastasis. Data suggest that the stability of banding patterns is not absolute and may prompt caution in the analysis of lineage and behaviour with this method. A mouse fibrosarcoma line (KHT) was transfected with a cosmid construct carrying a neomycin resistance gene. DNA from resistant clones was screened using pSV2 neo as a probe. Four cell lines, each with a different banding pattern, were injected (s.c.) both individually and as a mixture, into immunosuppressed mice. After four weeks, DNA from primary tumours and their associated metastases were analysed. It was found that, in agreement with previous work, most tumours arising from the mixed population gave a banding pattern consistent with that of one of the original cell lines, CB16. However, the results also indicated that the integration pattern of some cell lines can change *in vivo*. Compared to their primary tumours, fewer bands were present in DNA from individual metastases and in some cases new bands were observed. We conclude that in most primary tumours arising from the inoculation of mixed tagged clones the majority of cells are likely to be the progeny of only one of the original cell lines. However, the integration patterns of DNA from metastases suggested secondary deposits can develop from variants not detected in the injected cell population, but which arise subsequently by genetic recombination and selection events. In a second series of experiments using a non-metastatic line it was apparent that a large number of clones in the inoculum may contribute to tumorigenicity. The different results with two lines may reflect intrinsic properties of the cells.

### Inhibition of $^{125}$I-EGF binding to quiescent Swiss 3T3 fibroblasts and XB/2 keratinocytes by UVB light

G. Brooks, M.W. Goss & I.R. Hart

*Imperial Cancer Research Fund, Lincoln's Inn Fields, London WC2A 3PX, UK.*

Ultraviolet (UV) radiation is a major causative agent in human skin cancer. Exposure of cells *in vitro* to high doses of UV radiation leads to severe cytotoxic effects resulting from DNA damage, whereas lower doses (1 – 100 J m$^{-2}$) produce a variety of more subtle biological effects, including DNA synthesis and cell proliferation. The biochemical mechanisms leading to these low dose effects currently are unknown. We now show that exposure of Swiss 3T3 fibroblasts and XB/2 keratinocytes to UVB (302 nM) irradiation causes a dose-dependent inhibition of $^{125}$I-EGF binding which is maximal 60 minutes after initiation of the assay. Doses required to evoke a 50% inhibition of binding in the fibroblasts and keratinocytes are 81 J m$^{-2}$ and 85 J m$^{-2}$ respectively. Trypan blue exclusion assays showed this inhibition was not due to a cytotoxic effect. Scatchard analyses of normal and UVB-treated cells demonstrated that UVB exposure resulted in a decrease in the number of high affinity binding sites with no change in affinity constant. EGF receptor transmodulation was mediated via a protein kinase C (PKC)-independent pathway since UVB failed to stimulate phosphorylation of the 80 kDa substrate protein of PKC. Furthermore, down-regulation of PKC levels failed to affect the extent of UVB induced inhibition of $^{125}$I-EGF binding. $^{125}$I-PDGF binding to these cells was unaffected by light doses up to 280 J m$^{-2}$, indicating that inhibition of $^{125}$I-EGF binding is a specific event. Thus, the transmodulation effect of UVB on the EGF receptor appears to be mediated through a novel signal transduction pathway.

### The role of serum in the inhibition of growth by activators of protein kinase C (PKC) in human A549 lung carcinoma cells

T.D. Bradshaw & A. Gescher

*CRC Experimental Chemotherapy Group, Pharmaceutical Sciences Institute, Aston University, Birmingham B4 7ET, UK.*

The growth of A549 cells cultured in medium with 10% fetal calf serum (FCS) is arrested for 6 days by the PKC activator 12-0-tetradecanoyl-phorbol-13-acetate (TPA) (Dale & Gescher (1989), *Int. J. Cancer*, **43**, 158). Cells grown in media supplemented with 2% Ultraser (US), a defined serum substitute, underwent only a very transient growth inhibition of $<$24 h following TPA treatment. The hypothesis was tested that serum constituents play a role in the maintenance of growth arrest. Cells were cultured with either FCS or new born calf serum (NBCS). Growth was assessed by measuring incorporation of [$^3$H]-thymidine into the cells after incubation for 48 hours with TPA (10 nM). In the presence of 10, 5, 1 or 0% FCS TPA reduced growth to $10 \pm 2$, $28 \pm 10$, $43 \pm 6$ and $87 \pm 6$% of control respectively (mean $\pm$ SD, n = 9). In cells supplemented with NBCS (10%) TPA decreased cell proliferation by only $48 \pm 2$% (n = 6). Total PKC activity was similar in naive cells maintained in FCS or NBCS, but in medium the US PKC activity was only $32 \pm 3$% (n = 9) of that seen in cells with FCS. Exposure to TPA under all conditions caused translocation of PKC from the cytosolic to the particulate fraction and its subsequent downregulation. The PKC activator and experimental antitumour drug bryostatin 1 inhibited cell growth only transiently in the presence or absence of FCS, but it caused translocation and down-regulation of PKC similar to TPA. It appears that maintenance of TPA-induced growth inhibition involves complex PKC-independent processes mediated by serum factors.

### Effects of oestradiol, TPA and bryostatin on growth, the transcriptional regulation of oestrogen responsive genes and the expression of TGF-$\beta$ in human breast tumour lines

J. Nutt, A.L. Harris & J. Lunec

*Cancer Research Unit, University of Newcastle upon Tyne, UK.*

The phorbol ester, TPA (10 nM), produced a marked reduction in the growth of MCF7 cells in full growth medium but had only a small effect on MDA and T47D cells. The effect of TPA on MCF7 cells was partially reversed by bryostatin, suggesting bryostatin does not mimic TPA in this system even though both are believed to act via effects on protein kinase C. When the oestrogen receptor + ve MCF7 and T47D cells were maintained in charcoal-stripped serum, the increase in DNA synthesis on stimulation with oestradiol was inhibited with 50 nM TPA in MCF7 cells but not in T47D cells. The effects of these treatments on the expression of the oestrogen responsive genes pNR2 and pNR100 (Cathepsin-D) were examined. Rather than preventing transcription of these oestrogen responsive genes, TPA alone increased pNR2 and pNR100 mRNA levels in MCF7 cells and the combined effect of oestradiol and TPA had a marked synergistic effect in increasing the mRNA levels of these genes. In T47D cells pNR2 transcripts were not detected and the increase in pNR100 expression was not affected by TPA.

We conclude that the inhibitory effect of TPA on the growth stimulation of MCF7 cells by oestradiol was not due to a general inhibition of the expression of oestrogen responsive genes.

An alternative possibility examined was that the inhibitory effects of TPA on MCF7 cells might be due to the stimulation of TGF-$\beta$, acting as an autocrine inhibitory growth factor. Oestradiol treatment of MCF7 cells reduced the levels of TGF-$\beta$ mRNA, whereas TPA produced a marked increase. The combined effect of TPA and oestradiol further increased TGF-$\beta$ mRNA above the levels seen with TPA alone. Bryostatin had little effect of TGF-$\beta$ expression either alone or in combination with oestradiol. These observations suggest that the inhibitory effect of TPA on MCF7 cells is due to an autocrine inhibition by TGF-$\beta$.

### Synthesis and release of TGFα from the myeloid leukaemia cells HL60 treated with phorbol ester

D. Davies, S. Farmer & P. Alexander

*Department of Medical Oncology, The University of Southampton, Southampton General Hospital, SO9 4XY, UK.*

We had previously shown, immunohistologically, that macrophages which had infiltrated human carcinomas contained TGFα. To learn more about the synthesis and release of TGFα by macrophages we investigated, as a model system, HL60 cells that had been induced to differentiate and express a macrophage phenotype. In the absence of the phorbol ester, PMA, HL60 cells did not stain for TGFα. After culturing for 24 hours in the presence of PMA, the cells became adherent and also expressed the monocyte-macrophage surface marker CD11 as well as TGFα. The amount of TGFα observed, immunohistologically, increased with further culturing and plateaued after 72 hours. Concomitantly, we detected TGFα-like activity in the supernatant of the cultures. The TGFα was measured after the culture supernatants had been separated chromatographically and the fractions assayed for their ability to initiate, in confluent and quiescent foreskin fibroblasts, DNA synthesis which was capable of being inhibited by antibodies directed against the EGF receptor, but not by antibodies against EGF. Acid as well as detergent extracts of PMA treated HL60 cells yielded TGFα-like activity, the majority of which co-chromatographed with 6 kDa TGFα on a monoQ ion exchange column. The amount of TGFα-like activity associated with the cells was, however, much less than that found in the culture supernatant. When these experiments were repeated in the presence of protease inhibitors, the TGFα-like activity in the culture was reduced by 80%, whereas the cell associated TGFα-like activity was increased four-fold, or more, and showed a different pattern on chromatography. Since the production of diffusible TGFα can be blocked by inhibiting protease activity in the differentiated HL60 cells, it seems likely that the tumour growth promoting properties of macrophages may require not only the production of TGFα, but also the action of proteases.

### Multiple neuropeptides mobilise calcium in small cell lung cancer

P.J. Woll & E. Rozengurt

*Imperial Cancer Research Fund, PO Box 123, Lincoln's Inn Fields, London WC2A 3PX, UK.*

Small cell lung cancer (SCLC) typically secretes a variety of peptides and hormones. Of these, the bombesin-like peptides including gastrin releasing peptide (GRP), have attracted attention as putative autocrine growth factors. Mobilization of cytosolic calcium is an early event in the action of GRP and other growth factors. Using the fluorescent calcium indicator, Fura-2-AME, in five independent SCLC cell lines, we now show that the neuropeptides bradykinin, cholecystokinin, glanin, neurotensin and vasopressin, in addition to GRP, stimulate rapid, transient increases in cytosolic calcium in SCLC, at nanomolar concentrations. Responsiveness to individual peptides is heterogeneous between the diverse cell lines, but the ability to respond to regulatory peptides is a general phenomenon. Peptide responses demonstrate homologous desensitisation and are blocked by ligand-specific antagonists, indicating that they are mediated by distinct receptors. We suggest that multiple calcium-mobilizing neuropeptides may regulate the growth of SCLC.

### *In vitro* and *in vivo* response of A549 cells to a factor partially purified from steroid-treated human fetal lung fibroblasts

V. Speirs & R.I. Freshney

*CRC Department of Medical Oncology, University of Glasgow, Glasgow G12 9LX, UK.*

We have purified, partially, a factor from steroid-treated fetal lung fibroblast conditioned medium. This factor, designated MOG-FDF, is heat and alkali stable, but acid labile and partially protease labile. Incubation of A549 alveolar lung carcinoma cells with MOG-FDF induced pulmonary surfactant synthesis, a marker associated with differentiation in this cell line. MOG-FDF had no effect on A549 cell growth in monolayer, but reduced clonogenicity in suspension ($p < 0.02$) and plasminogen activator activity ($p < 0.002$) by 30%. There was also a 40% increase in total cellular and secreted glycosaminoglycans. These observations imply that the cells are becoming more differentiated and potentially less malignant. Intraperitoneal administration of MOG-FDF to nude mice bearing A549 xenografts markedly reduced the growth of the xenograft ($p < 0.01$). This was accompanied by an alteration in tumour histology, with extensive tissue reorganization and the formation of glandular-like structures, suggesting movement towards a more differentiated phenotype. Preliminary observations with xenografts of three other tumours suggest that the carcinostatic effect of MOG-FDF may be specific, at least to lung.

### Inherent sensitivity to chemotherapeutic drugs of testis tumour cell lines

G.R. Coren, E.J. Osborne, M.C. Walker, D.I. Harris, T.A. Maheady, C.N. Parris & J.R.W. Masters

*University College London, St Pauls Hospital, 24 Endell Street, London WC2H 9AE, UK.*

Continuous cell lines derived from non-seminomatous testicular germ cell tumours are hypersensitive to cisplatin and adriamycin (Walker *et al.* (1987), *J. Natl Cancer Inst.*, **79**, 213) and gamma-radiation (Parris *et al.* (1988), *Int. J. Radiat. Biol.*, **53**, 599), reflecting the curability of these tumours in patients. To determine if testis tumour cell lines are hypersensitive to chemotherapeutic drugs with different mechanisms of action (antimetabolites, topoisomerase II inhibitors, antibiotics, alkaloids and alkylating agents), we compared the sensitivities of five bladder cancer cell lines with those of six testis tumour cell lines to twelve anticancer agents (adriamycin, m-amsacrine, bleomycin, cisplatin, colchicine, 5-fluorouracil, methotrexate, methylnitrosourea, mitomycin-C, mitozolomide, vinblastine, VP-16). Dose-response curves were obtained using a MTT assay, with a minimum of three replicate experiments for each drug and cell line. Comparing mean $IC_{50}$ (concentration of drug causing a 50% inhibition of cell growth), the testis tumour cell lines were more sensitive to every agent. For the drugs most frequently used to treat testicular tumours, the mean $IC_{50}$ of the bladder cancer cell lines was greater than that of the testis tumour cell lines by a factor of 1.9 for vinblastine, 4.3 for cisplatin, 8.3 for VP-16 and 16.2 for bleomycin. These data confirm that testis tumour cell lines are hypersensitive to anticancer agents with various mechanisms of action, and indicate that these human cancer cell lines provide a model system with which to investigate the molecular basis of drug sensitivity.

### Can culture of A549 carcinoma cells under conditions conducive to differentiation alter the chemosensitivity of the cells to adriamycin?

V. Speirs & R.I. Freshney

*CRC Department Medical Oncology, University of Glasgow, Glasgow G12, UK.*

A549 cells were cultured on filter wells with, or without, foetal lung fibroblasts in the presence and absence of 0.25 μM dexamethasone (DX), since these conditions are known to enhance differentiation in this particular cell line, and the response of the cells to adriamycin (ADR) was determined. With ADR alone (no fibroblasts), there was an increase in plating efficiency of about 10%, and an increase in colony size, at sub-toxic drug concentrations ($\leqslant$0.1 μM). DX alone reduced the number of colonies by approximately 20%. Combining DX with various concentrations of ADR gave a greater stimulation of clonal growth (50%) at sub-toxic concentrations. On the addition of fibroblasts transfilter, control plating efficiency increased from 50 to 59% and from 41 to 66% in the presence of DX. Low concentrations of ADR had no growth stimulatory effect in the presence or absence of DX. While there was no difference in the $ID_{50}$ in the absence of fibroblasts, in the presence of fibroblasts DX reduced the $ID_{50}$ from 3 μM to 0.2 μM. These results suggest that conditions conducive to maximum induction of differentiation may increase chemosensitivity at pharmacological concentrations. As the anomalous increase in plating efficiency in low concentrations of ADR was abolished by culture in fibroblasts, this may be a nutritional artefact.

### Overexpression of P-glycoprotein in Chinese hamster ovary cells following fractionated X-irradiation *in vitro*

R.D.H. Whelan[1,2], K. Deuchars[2], L.K. Hosking[1], V. Ling[2] & B.T. Hill[1]

*[1]Imperial Cancer Research Fund Laboratories, London WC2A 3PX, UK; and [2]Ontario Cancer Institute, Toronto, Canada.*

Clinical drug resistance has been observed in patients treated not only with chemotherapy, but also with prior radiotherapy. We have previously shown that *in vitro* exposure of a series of mammalian tumour cell lines to fractionated X-irradiation resulted in the expression of resistance to vinca alkaloids and epipodophyllotoxins, but not to anthracyclines or to X-rays (NCI Monograph (1988), **6**, 177). The basis of this resistance has been studied with a series of X-ray-treated Chinese hamster ovary cell lines. Consistent with the classic multidrug resistance phenotype these lines: **i** were resistant to multiple drugs, but not to X-rays; **ii** had increased levels of P-glycoprotein (Pgp), as judged by Western immunoblotting using the C219 monoclonal antibody; **iii** showed reduced tritiated-vincristine accumulation; and **iv** were sensitive to reversal of vincristine resistance by verapamil. Pgp overexpression occurred without Pgp gene amplification or significant alteration in Pgp mRNA levels, implicating an alteration at the translational and/or post-translational level. These data provide evidence that exposure to X-irradiation can influence Pgp expression and screening programmes designed to correlate Pgp expression with clinical drug resistance should therefore be extended to include irradiated tumours.

### Sensitivity and resistance to platinum compounds in human lung cancer cell lines

P.R. Twentyman, K.A. Wright & T. Rhodes

*MRC Clinical Oncology and Radiotherapeutics Unit, Hills Road, Cambridge CB2 2QH, UK.*

Cisplatinum (CP) is one of the most effective drugs in the treatment of small cell and non-small cell lung cancer as well as other malignancies. A major objective of analogue synthesis is the development of compounds which retain activity in CP-resistant cells. We have developed a panel of human lung cancer cell lines with a range of sensitivities to CP and, from three of them, sublines with acquired CP-resistance. Dose response curves have been obtained using a 6–8 day MTT assay with continuous drug exposure. Seven small cell lines gave $ID_{50}$ values for CP ranging from 0.034 to 0.50 μg $ml^{-1}$ whilst the values for large cell line COR-L23 and adenocarcinoma line MOR were 0.12 and 0.85 μg $ml^{-1}$ respectively. Resistant sublines of small cell NCI-H69, COR-L23 and MOR, derived by *in vitro* exposure to increasing doses of CP, showed 9-fold, 4-fold and 3-fold resistance to CP respectively. For CP, carboplatin (CB), iproplatin (CHIP) and tetraplatin (TET) the range of $ID_{50}$ values for the small cell panel was 14.7, 10.0, 6.0 and 3.6 respectively. Resistance factors ( = $ID_{50}$ resistant/$ID_{50}$ parent) for the lines with acquired resistance were:

| *DRUG* | *NCI-H69* | *COR-L23* | *MOR* |
|---|---|---|---|
| CP | 8.8 | 4.3 | 3.5 |
| CB | 3.9 | 6.5 | 1.5 |
| CHIP | 2.3 | 5.3 | 1.0 |
| TET | 1.0 | 5.6 | 0.9 |

It is clear that the CP-resistant subline of COR-L23 has a much broader resistance profile than the other lines. In NCI-H69 and MOR, total lack of cross-resistance to TET is seen. Similarly the range of $ID_{50}$ values in the small cell panel is the lowest for TET. CP-resistance in this panel of cell lines cannot be accounted for by variations in glutathione or glutathione S transferase activity and further mechanistic studies are in progress. The lines provide an appropriate model system for screening new platinum analogues for use in the clinical therapy of lung cancer.

### A multinuclear NMR investigation of biochemical differences between sensitive and multidrug resistant cell lines

M.R. Müller[1], T.A. Moore[2], P.G. Morris[2] & P.R. Twentyman[1]

*[1]MRC Clinical Oncology and Radiotherapeutics Unit, MRC Centre, Hills Road, Cambridge CB2 2QH; and [2]Department of Biochemistry, University of Cambridge, Tennis Court Road, Cambridge CB2 1QW, UK.*

Multinuclear NMR spectroscopy is a powerful technique for the study of the metabolism of sensitive and drug resistant cells. As a model, we used the established human lung cancer cell line H69/P and its multidrug resistant counterpart H69/LX10. We compared the $^{1}H$ and natural abundance $^{13}C$ spectra of perchloric acid extracts of the parent resistant cell line. The most striking difference was one new peak in each of the spectra from resistant cells which has been identified as formate. The new peaks have identical chemical shifts to those produced by spiking with pure sodium formate even when the pH was increased from 7.0 to 11.0. A non-acid extraction method also showed the presence of large amounts of formate in resistant cells indicating that formate was not an artefact of the perchloric acid extraction procedure. Furthermore, comparison of $^{1}H$ and $^{13}C$ spectra from a multidrug resistant variant EMT6/AR1.0 of the mouse tumour cell line EMT6/P also showed the presence of an equivalent formate peak. Studies are underway to explain the possible role of formate in multidrug resistance.

### Repair of platinum-induced DNA damage in cell lines and human tumours

P. Harnett, I. Hickson & A.L. Harris

*Molecular Oncology Laboratory, Imperial Cancer Research Fund, John Radcliffe Hospital, Headington, Oxford OX3 9DU, UK.*

Human tumours exhibit a spectrum of response to platinum compounds, ranging from marked sensitivity (e.g. testicular carcinoma) to *de novo* resistance (e.g. pancreatic carcinoma). We are investigating the potential role of DNA excision repair mechanisms in the spectrum of tumour response to platinum. Protein extracts prepared from cell lines or direct biopsies of human tumours are reacted with plasmid DNA containing intrastrand platinum–DNA adducts. Platinum excision repair events are detected by the incorporation of radioactive nucleotides into repair patches. Protein extracts from a platinum sensitive testicular carcinoma cell line show considerably less excision repair capacity than an extract made from normal human testis. Preliminary results suggest that extracts from direct biopsies of bladder and ovarian carcinoma (platinum sensitive tumour types) possess less platinum repair capacity than extracts from HeLa or lymphoblastoid cell lines. Platinum sensitive CHO cell lines deficient in repair of inter- and intrastrand platinum crosslinks have also been defined using this assay. The results suggest that DNA repair mechanisms may play a role in tumour response to platinum chemotherapy. This *in vitro* assay may also allow the characterization of DNA repair mechanisms at the protein or genetic level.

### Heterogeneous expression of P-glycoprotein in breast cancer using two monoclonal antibodies, C219 and MRK16

G.C. Wishart[1], J.A. Plumb[1], J.J. Going[2], A.M. McNicol[2], C.S. McArdle[3] & S.B. Kaye[1]

*[1]CRC Department Medical Oncology, [2] Glasgow University, University Departments Pathology and [3]Surgery, Royal Infirmary, Glasgow, UK.*

A previous study showed detectable levels of mdr-1 mRNA in primary breast cancers using a cDNA probe to mdr-1. We have now investigated P-glycoprotein (P-gp) expression by immunocytochemistry on frozen sections of 28 primary breast cancers (all untreated) using two monoclonal antibodies against different epitopes of P-gp (C219 and MRK16) and an indirect alkaline phosphatase method. Staining with C219 revealed heterogeneous expression in epithelial cells in 21 of 29 tumours with marked stromal staining in 25. Although levels of staining were lower with MRK16 the same pattern was observed with expression in both epithelial (16 of 28) and stromal (12 of 29) cells. The stromal cells were confirmed as non-epithelial by positive staining with a monoclonal antibody against vimentin and the absence of staining when incubated with the anti-cytokeratin Cam 5.2. These results confirm P-gp expression in untreated primary breast cancers and suggest that stroma may play a role in drug resistance in breast cancer.

### The identification of drug resistance in ovarian malignancy using the MTT assay

J.M. Sargent, J.K. Wilson, A.W. Elgie, C.G. Taylor & J.G. Hill

*Departments of Haematology and Gynaecology, Pembury Hospital, Kent TN2 4QJ, UK.*

The prognosis of ovarian cancer (FIGO Stage III or IV) is very poor. A predictive drug sensitivity test before each course of chemotherapy could improve long-term survival. We describe a feasibility study using the MTT assay to assess the sensitivity of individual ovarian tumours to cytotoxic drugs. Ascitic fluid (38) and/or tumour biopsies (27) were obtained from 46 patients. There was a linear relationship between cell numbers and the OD of formazan produced. Incubating $1 \times 10^{5}$ cells with 100 µg MTT for 4 hours gave optimum formazan production. Formazan crystals were dissolved in DMSO. Cells obtained from ascitic fluid and solid tumour in the same patient showed the same sensitivity profile. Good reproducible results were obtained from 41 patients. Drug effect was remarkably varied between patients. The platinum derivatives produced the greatest cytotoxic effect. *De novo* resistance to all drugs tested was found in 15% of cases. On comparison of the effects of the platinum analogues and anthracycline analogues, similar effects were found in 62% and 75% of samples respectively. Cross-resistance patterns were comparable to the clinical response rates. In view of the variation in drug effect between patients, the proximity to clinical response rates, the ease of testing and high percentage of successful assays, we believe this method is suitable for identifying drug resistance in ovarian cancer.

### Nifedipine plus etoposide in the treatment of metastatic solid tumours: a phase I study

P.A. Philip[1], K. Tonkin[1], S. Monkman[2], J.R. Idle[2], J. Carmichael[1] & A.L. Harris[1]

*[1]ICRF Clinical Oncology Unit, Churchill Hospital, Oxford OX3 7LJ; and [2]Department of Pharmacological Sciences, University of Newcastle, Framlington Place, Newcastle upon Tyne, TE2 4HH, UK.*

Nifedipine (NF) is a calcium channel blocker commonly used in the treatment of hypertension and angina. *In vitro* studies have shown that it can reverse multidrug resistance (MDR) phenotype. Etoposide (E) is active in a variety of solid tumours. We investigated 14 patients with different solid tumours who were judged to be resistant to conventional systemic therapy; renal cell ca (5), breast ca (3), hepatoma (2), ovarian adenoma (2), soft tissue sarcoma (1), melanoma (1). Nine patients had received prior chemotherapy with agents other than E. Patients were treated with NF slow-release in 6-day courses repeated every 3 weeks. NF dose was raised from 40 mg to 80 mg twice daily, with only one dose escalation permitted per patient. E was administered i.v. on day 2 (100–200 mg $m^{-2}$) and orally on days 3 and 4 (400–600 mg per day). Plasma NF levels were determined in 10 patients at the third dose of NF;

| *Dose* mg | *mean Cmax* $\mu g\ l^{-1}$ | *mean AUC* $\mu g\ h^{-1}\ ml^{-1}$ | *no.* |
|---|---|---|---|
| 40 | 155.0 | 1.0 | 2 |
| 60 | 208.7 | 2.4 | 5 |
| 80 | 261.3 | 2.7 | 3 |

Median number of courses received was two. Side effects of NF were significant at the 80 mg dose level and correlated well with the pharmacokinetic data; 3 out of 6 patients experienced headaches and dizziness, 2 of whom had to have the dose of NF reduced because of a drop in systolic BP

greater than 30 mmHg. Myelosuppression was encountered with E, with 4 patients developing WHO grade 3/4 leucopenia and 1 patient grade 3 thrombocytopenia. These toxicities were greater than expected and could refer to altered E pharmacokinetics which has been investigated in 10 patients. There were no objective responses to this regimen. The predominant mode of action of E is via topo II isomerase. However, E has been shown to be involved in MDR phenotype. Although the clinically achievable levels of NF ($<1\ \mu M$) may have an effect on other parameters such as tumour blood flow, these levels are significantly lower than those that modify MDR *in vitro* ($>5\ \mu M$). Therefore, NF is unsuitable for clinical trials assessing modulation of MDR because of its toxicity.

### A clinical pharmacological assessment of the potential clinical application of a single isomer D-verapamil as a resistance modifier

J.L. Godden, J.H. Ahmed, P.A. Meredith & H.L. Elliott

*Department of Medicine & Therapeutics, Stobhill General Hospital, Glasgow G12 3UW, UK.*

It is widely accepted that the calcium antagonist verapamil, which is a racemic mixture of D- and L-isomers, is able to increase chemosensitivity in several multidrug-resistant cell lines but clinical applicability has been restricted by the cardiotoxic side effects of the high doses required to produce concentrations equivalent to *in vitro* activity (2–6.6 μM; 900–2700 ng $ml^{-1}$). There is experimental evidence that D-verapamil may be less cardiotoxic than racemic verapamil but with comparable activity as a drug resistance modifier (Plumb *et al. J. Biochem. Pharmacol.* in press). Therefore, this study investigated the cardiovascular side effects and plasma concentrations of D-verapamil in man.

In a double-blind, crossover, partially randomized study of 8 healthy male volunteers (age $27 \pm 7$ years, weight $74.5 \pm 6.5$ kg) single doses of placebo, 240 mg racemic verapamil and 3 doses (250, 500 and 1000 mg) of D-verapamil were assessed during a series of study days, at weekly intervals, with measurements of cardiac conduction (PR interval on ECG), plasma drug concentrations, blood pressure and heart rate at frequent intervals up to 12 hours after dosing. Significant prolongation of the PR interval by $53 \pm 21$ ms occurred with 240 mg racemic verapamil and was not exceeded by any dose of D-verapamil: $17 \pm 6$ with 250 mg, $29 \pm 19$ with 500 mg and $41 \pm 28$ ms with 1000 mg. However, transient postural hypotension was observed with 1000 mg D-verapamil. Peak plasma drug levels in the target range were obtained with both 500 mg and 1000 mg D-verapamil and predictions of steady state concentrations indicate that 500 mg three times a day would produce the concentrations desired for drug resistance modulation (i.e. over 1000 ng $ml^{-1}$) for most of the 24 hours without serious cardiovascular adverse effects.

### The *in vitro* and *in vivo* testing of pyrrolidino-iodo-tamoxifen, a new analogue of the antioestrogen tamoxifen

S.K. Chander[1], R. McCague[2] & R.C. Coombes[1]

*[1]St George's Hospital Medical School, London SW17, and [2]Institute of Cancer Research, Sutton, Surrey SM2 5NG, UK.*

Biological activity of a new analogue of the antioestrogen tamoxifen, pyrrolidino-iodo-tamoxifen (PIT – R. McCague, Halogenated Tamoxifens, *Brit. Pat. Appl.* 8621908/1986), was investigated *in vitro* using the MCF-7 cell line and *in vivo* using the hormone responsive, oestrogen receptor expressing NMU induced rat mammary tumour. Oestrogen receptor binding studies using rat uterus cytosol showed that PIT had at least five times greater relative binding affinity for the oestrogen receptor compared with tamoxifen. Cell culture studies were carried out to investigate the effects of PIT on the growth of MCF-7 cells. These cells were grown in Dulbecco's Modified Eagles medium without phenol red and supplemented with 5% steroid depleted fetal calf serum. Cell counts were made after 7-days treatment with 100 nM drug. PIT caused a two-fold greater inhibition of cell growth, which was significantly different compared to tamoxifen ($P<0.001$). The *in vivo* activity of PIT was investigated using the rat model. PIT was given at a dose of 0.2 mg per rat in 0.2 ml peanut oil intramuscularly for 5 days a week for 4 weeks. Tumour measurements were made once a week. PIT caused regression of all tumours and 58% regressed by $>50\%$. In comparison, tamoxifen, given at the same dose as PIT, did not cause regression of all tumours. Of those which did regress, only 42% regressed by $>50\%$. In conclusion, our preliminary studies on PIT have shown it to be a more effective inhibitor of oestrogen than tamoxifen both *in vitro* and *in vivo*, consistent with its greater binding affinity for the oestrogen receptor.

### *In vitro* and *in vivo* inhibitory effects of the gastrin receptor (GR) antagonist L-365,260 on the rat pancreatic tumour cell line, AR42J

S.A. Watson[1], P.J. Elston[1], L.G. Durrant[1] & D.L. Morris[2]

*[1]Cancer Research Campaign Laboratories and [2]Department of Surgery, University of Nottingham, Nottingham NG7 2RD, UK.*

The pancreatic tumour cell line AR42J was found to be positive for GR as assessed by a radio ligand binding assay using $^{125}I$ Gastrin-17 (G17). The affinity of receptors ($5 \times 10^4$ $cell^{-1}$) for binding human G17 was $1 \times 10^{-9}$ M. L-365,260 (Merck, Sharpe and Dohme) is a benzodiazepine and a selective GR antagonist binding to both GR and $CCK_B$ receptors. It was found to bind competitively to GR on AR42J cells, with the concentration inducing 50% inhibition of $5 \times 10^{-10}$ M $^{125}I$ G17 binding being $4.5 \times 10^{-8}$ M for L-365,260 compared with $6 \times 10^{-9}$ M for G17 itself. Inhibition of $^{125}I$ G17 binding to AR42J cells induced by prior incubation (1 hour) with unlabelled G17 began to reverse after 3 hours from 80% to 33% and 23% after 4 hours. Prior incubation with L-365,260 induced greater inhibition of binding of $^{125}I$ G17; 60% at 3 hours and 51% at 4 hours which was significant ($P<0.05$). G17 was found to be mitogenic for AR42J cells as assessed by $^{75}Se$-selenomethionine uptake and cell counting (growth stimulation 170 to 250% of untreated control at the optimal concentration of $5 \times 10^{-10}$ M G17). L-365,260 at $2.5 \times 10^{-6}$ M and $2.5 \times 10^{-7}$ M (55 × and 5.5 × the dose required to displace $5 \times 10^{-10}$ M $^{125}I$ G17) reduced optimal G17-stimulated mitogenesis in 3/3 experiments. The growth of AR42J xenografts in nude mice was increased to 280% of PBS treated controls by administration of G17 (10 μg per mouse daily by osmotic mini-pump, $P<0.027$). This stimulation was blocked by co-administration of L-365,260, dosed orally (5 mg kg daily, $P<0.034$). We have shown that 71% of human colorectal and 100% of human gastric primary tumour cells possess GR ($5 \times 10^2$ to $10^4$ per cell), thus therapy with potent GR antagonists may have therapeutic potential.

### Growth modulation of two lymphoma cell lines by a novel calcitriol analogue MC903

T. Hickish[1], M. Soukop[2], B.C. Millar[1], T.J. McElwain[1] & D. Cunningham[1]

*[1]Royal Marsden Hospital, Sutton, Surrey and [2]Glasgow Royal Infirmary, Scotland, UK.*

After apparently successful induction chemotherapy almost all patients with low grade non-Hodgkin's lymphoma relapse, therefore there is a requirement to develop novel treatments for application to the maintenance phase. We have previously shown that alfacalcidol, which is metabolized to calcitriol, can produce tumour regression in 30% of patients with low grade non-Hodgkin's lymphoma. The dose-limiting toxicity of alfacalcidol is hypercalcaemia but the analogue MC903 has similar antiproliferative effects to calcitriol but 100 times less of an effect on mineral metabolism. We have studied the growth modulating effect of calcitriol and MC903 (Leo laboratories) on two diffuse histocytic cell lines SU-DHL8 and SU-DHL4. The latter has a t(14;18) characteristic of 80% of follicular small cleaved cell lymphoma. Toxicity was measured using the incorporation of $^{3}H$ thymidine and by the growth of colonies in soft agar. Results were:

| *Cpm Min$^{-1}$* | *Controls Mean* | *SD* | *Calcitriol Mean* | *SD* | *MC903 Mean* | *SD* | | *Surviving fraction molar $10^{-6}$* | *$10^{-7}$* | *$10^{-8}$* |
|---|---|---|---|---|---|---|---|---|---|---|
| SU-DHL4 | 22079 | 4564 | 706 | 346 | 112 | 46 | MC 903 | .46 | .50 | .53 |
| SU-DHL8 | 3815 | 1218 | 229 | 134 | 202 | 103 | calci-triol | .48 | .52 | .54 |

These data show MC903 and calcitriol produce a similar reduction in the thymidine uptake suggesting an antiproliferative effect on both cell lines. The clonogenic assay shows an antiproliferative effect that is similar for the two drugs on the clonogenicity of SU-DHL4 cells. These data suggest MC903 may be an effective antiproliferative agent in lymphoma and indicate a role for a calcitriol analogue in the maintenance phase of low grade lymphoma.

### The role of the vasculature in the anti-tumour action of flavone acetic acid

L.J. Zwi[1], B.C. Baguley[2], J.B. Gavin[1] & W.R. Wilson[1]

[1]*Department of Pathology, University of Auckland and* [2]*Auckland Cancer Research Laboratory, Auckland, New Zealand.*

Flavone acetic acid (FAA), an experimental anti-cancer agent with a broad spectrum of activity against solid tumours in mice, appears to act by an indirect mechanism. Using a double label fluorescent marker technique, we showed that FAA caused discrete foci of perfusion loss within 15 minutes of administration in Colon 38 and EMT6 tumours. In addition, FAA caused more than 100 times the level of cell kill in vascularized intramuscular EMT6 tumours compared to avascular EMT6 intraperitoneal spheroids (Zwi *et al.* (1989), *J. Natl Cancer Inst.*, **81,** 1005). Site- or size-related factors other than vascularization were excluded by examining the effect of FAA on EMT6 spheroids undergoing vascularization in the mouse peritoneum. Surprisingly, the outer zones of the spheroids remained avascular after the necrotic cores were replaced by vascularized tumour tissue, allowing comparison of the responses of vascular and avascular tumour tissue within the same tumour mass. FAA treatment resulted in haemorrhagic necrosis of the vascularized core, while the outer avascular zone remained viable. The results indicate that a major part of FAA-induced tumour cell killing is dependent on the presence of blood vessels, and that the mode of cell death is predominantly ischaemic. FAA appears to belong to a class of agents (including endotoxin, tumour necrosis factor-α, interferons and interleukin-1) which cause tumour necrosis in association with vascular effects, although FAA is the only synthetic or small molecule known to act in this way.

### Mechanism of flavone acetic acid-induced tumour blood flow inhibition

V. Mahadevan, S.T.A. Malik, A. Meager & I.R. Hart

*Imperial Cancer Research Fund, Lincoln's Inn Fields, London, WC2A 3PX, UK.*

Flavone acetic acid (FAA), an investigational antitumour agent which is active against several subcutaneously (s.c.) transplanted murine tumours, causes early shutdown of blood flow in these tumours. Indeed, this effect is believed to be a prime determinant in FAA's antitumour action. However, the mechanism whereby FAA induces this vascular shutdown is unknown. By measuring the clearance of intratumourally injected radiolabelled Xenon ($^{133}Xe$) as an index of tumour blood flow, we have demonstrated an early, progressive and sustained reduction of blood flow in a s.c. Colon 26 tumour in Balb/c mice treated with intraperitoneal (i.p.) FAA (200 mg kg$^{-1}$). This reduction, noticeable as early as one hour after FAA injection, peaked at 3 hours ($T\frac{1}{2}$ (min) for $^{133}Xe$ clearance $117.3 \pm 36.4$ vs $7.8 \pm 0.85$, for controls n = 5/ group). Significant inhibition was still apparent 48 hours after a single injection of drug. Intravenous pretreatment of tumour bearing mice with an antiserum against recombinant murine tumour necrosis factor-α prevented this observed blood flow inhibition ($T\frac{1}{2}$ (min) $10.8 \pm 1.2$ vs $65.6 \pm 8.0$ for controls pre-treated with non-immune sheep serum. n = 7/ group). Furthermore, *in vitro*, FAA induced TNF secretion from murine peritoneal cells and splenocytes. Our results indicate that FAA-induced vascular shutdown in Colon 26 tumours is mediated by tumour necrosis factor.

### Induction of tumour hypoxia by TNF and FAA: interaction with bioreductive drugs

H.S. Edwards, J.C.M. Bremner & I.J. Stratford

*MRC Radiobiology Unit, Chilton, Didcot, Oxon OX11 0RD, UK.*

Physical clamping occludes the blood supply to subcutaneous murine tumours thus decreasing the supply of oxygen and producing 100% radiobiological hypoxia. Tumour necrosis factor (TNF) modulates the activity of certain immune system components leading ultimately to a reduction in tumour blood flow. We studied how TNF treatment influences the radiobiological hypoxic fraction using an *in vivo/in vitro* clonogenic assay. The radioprotective effect of clamping was compared to that produced by various concentrations of TNF administered to mice at various times before irradiation. When TNF was given at a dose of $2.5 \times 10^{5}$ U kg$^{-1}$ an increase in tumour hypoxia was seen after 30 minutes. Close to 100% radiobiological hypoxia (equivalent to that resulting from clamping) was reached one hour after treatment and lasted for up to 16 hours. The minimum dose of TNF that produced a level of hypoxia comparable to clamping was $1 \times 10^{5}$ U kg$^{-1}$ when administered three hours before irradiation. TNF was combined with the bioreductive drugs RSU 1069 and SR 4233 in the KHT and RIF-1 murine sarcomas and the antitumour effect was assessed by a regrowth delay assay. When an 80 mg kg$^{-1}$ dose of RSU 1069 was administered to mice bearing the KHT sarcoma 15 minutes before TNF ($1 \times 10^{5}$ U kg$^{-1}$) a mean specific growth delay of $3.9 \pm 0.6$ was seen. When the second bioreductive drug SR 4233 (50 mg kg$^{-1}$) was combined with the same TNF treatment a mean specific growth delay of $3.5 \pm 0.9$ was produced. TNF alone at this dose and both RSU 1069 and SR4233 alone have no effect on tumour growth. Qualitatively similar results were obtained with flavone acetic acid (FAA), although the lowest dose that induced close to 100% tumour hypoxia, 200 mg kg$^{-1}$, also provided a substantial effect when used alone in the RIF-1 tumour.

### Enzyme-directed bioreductive drugs: metabolism of developmental indoloquinone E09 and related agents by DT-diaphorase

P. Workman, M.I. Walton, M. Binger, S.M. Bailey & N.R. Suggett

*MRC Clinical Oncology Unit, MRC Centre, Hills Road, Cambridge CB2 2QH, UK.*

The indoloquinone series is undergoing preclinical development under the auspices of EORTC. The lead compound E09 [3-hydroxy-5-aziridinyl-1-methyl-2-(*H*-indole-4,7-indione) -propenol] is expected to enter clinical trial in 1990. The chemistry of this agent suggests it would function as a potent bioreductive alkylating agent. Indeed, it has excellent hypoxic cell specificity for a quinone, together with preferential activity for solid tumours versus leukaemias *in vitro*. In many cases the cytotoxicity profile is superior to that of the benchmark bioreductive quinone alkylating agent mitomycin C (MMC). The role of one versus two electron (1e vs 2e) reduction in the bioactivation of quinone alkylating agents remains controversial. The obligate 2e donating enzyme DT-diaphorase may potentially bioactivate or detoxify quinones, but recent results suggest that mitomycin C is not metabolized by this enzyme (Workman *et al.* (1989), *Br. J. Cancer*, **60**, 800). Therefore, we have examined the ability of E09 and related agents to act as substrates for reduction by DT-diaphorase. Human HT29 colon carcinoma and rat UK Walker 256 tumour cells were used as rich sources of DT-diaphorase. Activity was measured spectrophotometrically by reduction of cytochrome *c* or by loss of drug absorbance at 510 nm. Both preparations were able to metabolize E09 much more efficiently than MMC, the latter being barely detectable. For equal menadione reduction rates, the rat DT-diaphorase was about 10-fold more efficient at E09 metabolism compared to the human enzyme. Km values were 20–30 $\mu$M. For a methoxy analogue the relative rates were similar to those for E09. With the aziridine ring-opened derivative E05A the reduction rates were markedly lower. We conclude that DT-diaphorase does metabolize the indoloquinones, and the biological significance of this is now under scrutiny.

### Demonstration of low and biologically insignificant bioreduction rates for mitomycin C (MMC) by DT-diaphorase

M.I. Walton, P.J. Smith & P. Workman

*MRC Clinical Oncology Unit, MRC Centre, Hills Road, Cambridge CB2 2QH, UK.*

DT-diaphorase [NAD(P)H; (quinone-acceptor) oxidoreductase, EC. 1.6.99.2] is an obligate two electron (2e) donating enzyme. It detoxifies simple quinones by direct reduction to hydroquinones, bypassing the reactive semiquinone free radical. There has been considerable interest in DT-diaphorase involvement in the bioactivation/detoxification of the more complex quinone bioreductive alkylating agent MMC, and recent results suggest that MMC is not a substrate for the enzyme (Workman *et al.* (1989), *Br. J. Cancer*, **60**, 800). We have now used increased sensitivity assays to assess this reaction and to determine its biological significance. Preparations used included extracts of the enzyme-rich UK Walker 256 rat tumours and HT29 human colon carcinoma cells, and also purified Walker (from R.J. Knox) and human kidney (from G. Powis) enzymes. No activity could be determined by MMC loss or metabolite formation on HPLC with multidiode assay detection. However, very low, dicoumarol-inhibitable rates could be detected using transfer of electrons from enzyme-reduced MMC to cytochrome *c* (cyt *c*). For both the rat and human enzyme, rates were about 200-fold lower than for the standard substrate menadione. With the rat enzyme the Km was high at 1 mM and the $V_{max}$ low at 6 nmol cyt *c* reduced $min^{-1}$ Unit $enzyme^{-1}$. Using an agarose gel mobility assay, we could demonstrate no conversion of purified, negatively supercoiled, circular, double-stranded pBR322 plasmid DNA to either relaxed circular, linear or degraded forms by DT-diaphorase-MMC mixtures. Relaxation indicative of single-strand nicking was however seen in the positive controls combining DT-diaphorase with indoloquinone EO9. We conclude that DT-diaphorase can reduce MMC, but at extremely low rates, and this is unlikely to be of biological significance.

### *In vitro* antitumour activity of membrane-active analogues of platelet activating factor (PAF)

P. Workman, C. Dive, M. Lohmeyer & J. Donaldson

*MRC Clinical Oncology Unit, MRC Centre, Hills Road, Cambridge CB2 2QH, UK.*

Various agents structurally related to PAF are in development as antitumour agents. The cytotoxicity of these agents at pharmacological concentrations may involve multiple effects on membrane structure and signal transduction, including changes in membrane fluidity, inositol lipids, calcium signalling and protein kinase C. We have compared the cytotoxic effects of PAF, ET-18-$OCH_3$, SRI 62-834 and hexadecyl phosphocholine (HPC) on various cell lines. These were the EMT6 mammary tumour and its multidrug resistant (MDR) variant AR1.0; the UK Walker 256 rat tumour; the HT29 human colon carcinoma; and the HL60 human leukaemia. The MTT tetrazolium dye reduction assay was used. $ID_{50}$ values varied between the different agents. SRI 62-834 and ET-18-$OCH_3$ were the most potent and PAF the least, with HPC intermediate. Variation between cell lines was also seen. For example with SRI 62-834 the $ID_{50}$ was 0.3 $\mu$M in HL60, 3 $\mu$M in HT29, 15 $\mu$M in Walker and 75 $\mu$M in the EMT6 parent line. The $ID_{50}$ value of 100 $\mu$M in the MDR variant indicated that there was minimal cross-resistance to adriamycin (ADM). In view of the membrane activity of ADM and the membrane localization of the P170 MDR drug efflux pump, we determined the effect of SRI 62-834 on ADM sensitivity in the EMT6 lines. At 10 and 20 $\mu$m SRI 62-834, there was minimal effect on $ID_{50}$ values for ADM (0.05 $\mu$g $ml^{-1}$ in the EMT6 parent and 2 $\mu$g $ml^{-1}$ in AR1.0. For 1 and 2 $\mu$M SRI 62-834 the ADM $ID_{50}$ in HT 29 was also unchanged at 0.03 $\mu$g $ml^{-1}$. We conclude that the various PAF analogues have varying cytotoxic potencies in the cell lines used; wide differences in activity occur across various cell lines; and analogue SRI 62-834 does not modulate ADM sensitivity in the parent or MDR lines tested.

### A limited sampling strategy for the calculation of etoposide pharmacokinetics

S.P. Joel, L. Heap, S. Robbins, P.I. Clarke & M.L. Slevin

*ICRF Department of Medical Oncology, St. Bartholomew's and Homerton Hospitals, London EC1A 3BE, UK.*

Recent interest has focused on the calculation of pharmacokinetic parameters of cytotoxic drugs from a limited number of blood samples (Egorin *et al.* (1989), *ASCO*, 243). In response to this a limited sampling model has been developed for the calculation of etoposide pharmacokinetics in patients receiving etoposide 100 mg $m^{-2}$ as a 2-hour infusion. Blood samples for etoposide estimation were taken at 18 time points over a 24-hour study period. The model was derived from stepwise forward regression analysis of etoposide concentration (C) at 18 time points (independent variable) against $AUC_{0-\infty}$, (dependent variable) in 25 sets of data. The final model used 3 time points (1.5 hours (h), 10 h and 24 h) to predict AUC ($r^2 = 0.97$). Further, stepwise forward regression analysis of elimination rate constant ($k_{el}$) against $\log_e$ concentration produced only 2 significant time points, 10 h and 24 h, accounting for 91% of the variability in $k_{el}$. From the predicted AUC and $k_{el}$ the volume of distribution (Vd) and clearance (Cl) could also be predicted. The ability of this 3 time point model ($AUC = 12.7C_{10h} + 32.0C_{24h} + 3.26C_{1.5h} - 3.771$) to predict AUC, $k_{el}$, Vd and Cl was tested in a further 28 data sets. The model proved highly predictive for AUC (vs measured AUC r = 0.95) and was unbiased (mean percentage error (MPE), (± SEM), − 1.2 ± 1.4%) and precise (mean absolute percentage error (MAPE), (± SEM), 5.4 ± 1.0%). The model also proved highly predictive for $k_{el}$ (r = 0.91, MPE = 2.7 ± 1.7%, MAPE = 7.5 ± 1.0%) and Cl (r = 0.98, MPE = 3.3 ± 1.6%, MAPE = 6.4 ± 1.2%), and slightly less predictive for Vd (r = 0.87, MPE = 1.6 ± 2.7%, MAPE = 11.6 ± 1.6%). This study demonstrates that the AUC of etoposide after i.v. infusion can be reliably predicted from a limited number of samples, and that the model can reasonably be extended to include other pharmacokinetic parameters.

### Biochemical effects, antitumour activity and pharmacokinetics of oral and intravenous pamidronate (APD) in the treatment of skeletal breast cancer

D. Dodwell[1], A. Howell[1], A.R. Morton[2], P. Daley-Yates[3] & J. Ford[4]

[1]*Department of Medical Oncology, Christie Hospital, Wilmslow Road, Manchester, M20 9BX, UK;* [2]*Department of Nephrology, Wellesley Hospital, Ontario, Canada;* [3]*Department of Pharmacy, University of Manchester, UK; and* [4]*Ciba-Geigy, Basle, Switzerland.*

We have studied the antitumour and biochemical effects, toxicity, and pharmacokinetcs of both intravenous and an enteric-coated oral preparation of pamidronate (Ciba-Geigy) in two phase I/II trials in patients with bone metastases from breast cancer. In the intravenous trial, 22 assessable patients with progressive bone metastases from breast cancer (16 of whom had elevated calcium excretion with a fasting urine calcium/creatinine ratio (UCCR) >0.4 mmol/mmol before treatment) were treated with 30 mg intravenous pamidronate weekly, for 4 weeks and thereafter fortnightly for six months or until disease progression. Responses, as defined by sclerosis of previous lytic metastases, occurred in 4 patients and an additional 7 patients achieved stable disease. There was a significant reduction in the level of calcium excretion. There was also a significant improvement in pain symptoms. Median response duration (inc. 'no change') was 9 months. Toxicity was minimal.

In the oral study, 17 women with progressive bone metastases from breast cancer (16 with elevated calcium excretion) were given oral pamidronate at doses from 150 mg to 600 mg daily (cohorts of 4 patients) for four weeks. Thereafter all patients were continued on 300 mg daily. There have been no responses and only one patient has achieved stable disease. Pain symptoms were unaffected. There was, however, a similar fall in mean calcium excretion to that seen in the intravenous study. There were no significant differences in efficacy between treatment groups within the oral study, but gastrointestinal toxicity was dose limiting at 600 mg daily. In addition we have studied the bioavailability of pamidronate in 7 patients taking 300 mg daily of the oral preparation. Three sequential 24-hour urine samples were collected from each patient and the concentration of drug estimated according to the method of Daley-Yates *et al.* (*J. Chromatography*, 1989). Mean absorption (assuming a similar renal excretion to that seen after an intravenous infusion) varied from 0.16% to 0.41%. Despite similar effects on calcium excretion the oral enteric-coated preparation of pamidronate was poorly absorbed and this may explain the lack of any antitumour effect. More active bisphosphonates may circumvent the problem of poor bioavailability.

### Improving drug delivery to liver metastases: the effects of angiotensin II

D.M. Hemingway[1], D. Chang[2], J.A. Goldberg[1], S.A. Jenkins[2] & T.G. Cooke[1]

[1]*University Departments of Surgery, Royal Infirmary, Glasgow; and* [2]*Royal Liverpool Hospital, Liverpool, UK.*

Angiotensin II, a hepatic artery vasoconstrictor, has been shown to increase the tumour:liver blood flow ratio in patients with colorectal liver metastases. We have tested the effects of regional or systemic angiotensin II on the distribution of a marker resembling chemotherapeutic drugs to relate these blood flow changes to drug delivery in a hypovascular model of liver tumour. Metastases were induced by the intraportal inoculation of $10^6$ HSN sarcoma cells. $^{99m}Tc$ labelled methylene diphosphonate was injected into the hepatic artery after either an intravenous infusion, or an intra-arterial bolus of angiotensin II.

Results, expressed as % injected dose per gram of tissue × $10^1$, were:

| | *Controls* | | *IA Angiotensin* | | *IV Angiotensin* | |
|---|---|---|---|---|---|---|
| | *Liver* | *Tumour* | *Liver* ** | *Tumour* * | *Liver* ** | *Tumour* |
| 1 min | 3.55±0.15 | 5.17±0.55 | 11.1±1.1 | 23±5.7 | 16±5.4 | 9.6±3.0 |
| 90 min | 0.79±0.07 | 0.61±0.04 | 6.4±1.4 | 3.3±0.07 | | |

*$P<0.01$ **$P<0.02$

These results indicate that Ia angiotensin causes significant retention of marker in tumour, but that intravenous angiotensin causes greater retention in normal liver. Angiotensin should be given intra-arterially to maximize delivery of chemotherapeutic drug to liver tumour.

### Antioxidant vitamins (A, E and C) and lactulose in the prevention of the recurrence of adenomatous polyps: preliminary results of a controlled study

M. Ponz de Leon, L. Roncucci, P. Di Donato & M. Perini

*Instituto Patologia Medica and Instituto Semeiotiva Medica, Univ. Modena 41100, Italy.*

It is now generally accepted that adenomatous polyps represent the natural precursor of many colorectal cancers. Although polyps can easily be removed at endoscopy, they tend to reappear in approximately 30% of the patients at one year. We evaluated the long-term administration of vitamins, because of their antioxidant effect, or lactulose, which lowers the intestinal pH, thus limiting the degradation of primary bile acids to the more toxic secondary bile acids, in the reduction of the recurrence rate of colonic polyps. After endoscopic removal of polyps, 253 patients were randomized into 3 groups: **a** vitamins (A 30,000 U, E 70 mg and C 1 g daily); **b**. lactulose (20–40 g daily); and **c**. no treatment. Control endoscopies were carried out after 6, 12, 18 and 24 months. Among the 129 patients followed for at least 12–18 months, the cumulative recurrence of polyps was 4 in group **a**, 8 in group **b** and 24 in group **c**. The difference was statistically significant by Life-Tables and Log-Rank tests ($\chi^2$ 15.0, $P<0.001$). These preliminary results, therefore, suggest that either vitamins or lactulose may reduce the recurrence rate of adenomatous polyps, though the effect seems more marked for antioxidant vitamins. Both agents deserve to be further investigated for the prevention of colorectal cancer.

### Outcome of patients relapsing after treatment of non-seminomatous testicular germ cell tumours

J.A. Ledermann, L. Holden, E.S. Newlands, G.J.S. Rustin, R.H.J. Begent & K.D. Bagshawe

*Cancer Research Campaign Laboratories, Department of Medical Oncology, Charing Cross Hospital, London W6 8RF, UK.*

Progression of metastatic non-seminomatous testicular tumour during treatment generally carries a poor prognosis but the outcome of patients (pts) who relapse or progress after a partial or complete remission is less well known. Between 1977 and 1988 we treated 48 pts who had been off treatment for a median of 8 months (range 1-85); 28 pts had been in complete remission (11 after surgery) and 20 had residual radiological abnormalities. Twenty-three pts (47.9%) were at 'low risk' of relapse (MRC criteria 1989) as they had low serum tumour markers (hCG$<$10,000 iu $l^{-1}$; AFP$<$ 1,000 ku $l^{-1}$; $<$20 lung metastases; and no brain, bone or liver metastases). Radiotherapy had been given to 11 of this group and in 6 it was the only treatment. Twenty-seven/48 pts (58.2%) had previously been treated with the POMB/ACE regimen and 18 (66.7%) of these were in a 'high risk' group; 9 pts had been treated with PVB and 6 with other chemotherapy regimens. Relapse therapy comprised etoposide 150 mg $m^{-2}$ and cisplatin 75 mg $m^{-2}$ alternating each week with vincristine 1.2 mg $m^{-2}$, methotrexate 300 mg $m^{-2}$, folinic acid rescue and a 24-hour infusion of bleomycin 15 mg in 28 pts. POMB/ACE was given to 12 and other platinum regimens to 8 pts. The median survival is 23.5 months with a predicted survival at 10 years of 33%. Ten pts have been followed for more than 5 years and none has relapsed. Resection of tumour was performed in 15/20 survivors; 6 of whom had active tumour at surgery. Nineteen of the survivors (8 from low risk and 11 from high risk group) had initially received chemotherapy; provided their tumours retained chemosensitivity long term survival is possible.

### Node negative breast cancer: prognostic subgroups defined by tumour size and flow cytometry

S.M. O'Reilly, R.S. Camplejohn, D.M. Barnes, R.R. Millis, R.D. Rubens & M.A. Richards

*ICRF Clinical Oncology Unit, Guy's Hospital, London SE1 9RT; and Richard Dimbleby Department for Cancer Research, St Thomas' Hospital, London SE1 7EH, UK.*

Adjuvant therapy for patients with node negative breast cancer is most easily justified for those at highest risk of relapse. We have examined the impact of tumour size, tumour grade, oestrogen receptor (ER) status, tumour ploidy and S-phase fraction on relapse-free survival (RFS) for 169 patients with node negative breast cancer in order to identify groups of patients at high and low risk of relapse. Patients with small tumours ($\leqslant$1.0 cm) had a significantly longer RFS than those with larger tumours ($P = 0.005$), with 96% remaining relapse free at 5 years. Patients with tumours $<$1.0 cm were thus excluded from analysis when attempting to define a group with a poor prognosis. Within the group of patients with tumours $>$1.0 cm, neither ploidy ($P = 0.63$) nor ER status ($P = 0.3$) predicted for RFS. Patients with grade 1 or 2 infiltrating ductal tumours had a significantly better prognosis than those with grade 3 tumours ($P = 0.04$). Patients whose tumours were $>$1.0 cm with an SPF $\leqslant$10% had a 5 year RFS of 78% compared with a 5 year RFS of 52% for those with an SPF$>$10% ($P = 0.006$). We have combined tumour size and SPF to identify 3 prognostic groups:

| Patients | No | 5 year RFS |
|---|---|---|
| Tumour $\leqslant$1.0 cm | 33 | 96% |
| Tumour $>$1.0 cm + SPF $\leqslant$10% | 45 | 78% |
| Tumour $>$1.0 cm + SPF $>$10% | 71 | 52% |

These prognostic groupings may help identify patients most suitable for adjuvant therapy.

### High dose epirubicin as primary chemotherapy in advanced breast carcinoma: a phase II study

D.W. Miles, M.A. Richards[1], R.D. Rubens[1], J. Carmo-Pereira[2], F.O. Costa[2] & E. Henriques[2]

*[1]ICRF Clinical Oncology Unit, Guy's Hospital, London, UK; and [2]Instituto Portuges de Oncologia Francisco Gentil, Lisbon, Portugal.*

A linear dose response relationship has been reported for doxorubicin (D) in the treatment of advanced breast cancer. However, the dose of D which can be given safely is limited by both myelosuppression and cardiotoxicity. We have therefore conducted a phase II study using epirubicin (E), an analogue of D which is reported to be less cardiotoxic, at high dose (120 mg $m^{-2}$) in patients (pts) with locally advanced or metastatic breast cancer. Forty pts (median age 52, range 29-65, previous non-anthracycline containing adjuvant chemotherapy 6/40) were treated every three weeks for a median of 8 courses (range 2-10, median cumulative dose 960 mg $m^{-2}$, range 240-1200 mg $m^{-2}$). Response rate was 26/40 (65%) with a complete response rate of 9/40 (23%). Median duration of response was 7 months (range 1-15), median survival was 13 months (range 2-20). Toxicity: WHO grade IV leucopenia was observed in 10/10 pts in whom nadir counts were measured on day 10 but only 1/40 pts developed neutropenic sepsis. Left ventricular ejection fraction fell by $>$15% in 5/40 pts but reverted to normal after stopping E. As the dose of E was limited by myelosuppression, the use of colony stimulating factors to allow further dose escalation is now being investigated.

### Mitomycin, ifosfamide and cisplatin: an effective, pre-operative treatment for oesophageal carcinoma

H. Matthews, S. Walker, A. Steel & M.H. Cullen

*East Birmingham Hospital & Queen Elizabeth Hospital, Birmingham, UK.*

In 1986 we began evaluating systemic therapy for oesophageal carcinoma (OC) and reported the difficulty, noted by others, of assessing response to chemotherapy (CT) in this tumour. To resolve this we studied the effect of brief, pre-operative ('neo-adjuvant') CT in patients with apparently operable squamous or undifferentiated tumours, not requiring urgent surgery, to allow assessment of direct operative and microscopic response. Mitomycin (M), and cisplatin (C) are active in OC. Cullen *et al.* (1988) have shown that MC with ifosfamide (I) is an active and acceptable combination in squamous lung cancer, and so MIC was chosen for this study. Eleven previously untreated cases of obstructing OC (9 squamous, 2 undifferentiated) aged 55-73 (median 63) were treated with 2 courses of MIC (M 6 mg $m^{-2}$, C 50 mg $m^{-2}$). All had pre- and post-treatment CAT scans and barium swallows. Toxicity was as reported for MIC in lung cancer (Cullen *et al.* (1988), *Br. J. Cancer*, **58**, 359). Complete resection (left-sided subtotal oesophagectomy) of macroscopic tumour was possible in all 11 cases and there were no operative or post-operative deaths. Evidence of macroscopic response to chemotherapy was seen in all resected specimens.

In six this was complete with normalization of mucosal appearances with residual scarring. In the remaining five cases there was epithelialization of ulcerated mucosa and flattening of ulcer margins. Histological examination revealed no evidence of malignancy in three cases. Two patients died of OC (at 4 and 20 months) and 8 cases are still in remission (median FU 8 months).

### Prolonged administration of single-agent oral etoposide in small cell lung cancer (SCLC)

P.I. Clark[1], B. Cottier[1], S.P. Joel[2], P. Thompson[2] & M.L. Slevin[2]

[1]*Mersey Lung Cancer Group, Clatterbridge Hospital, Merseyside L63 1XG; and* [2]*Department of Medical Oncology, St. Bartholomew's and Homerton Hospitals, London EC1A 7BE, UK.*

Pharmacological studies in man suggest that the schedule-dependent efficacy of etoposide in SCLC is related to the prolonged maintenance of low blood concentrations of drug. Thirty elderly or unfit patients with SCLC received single-agent oral etoposide 50 mg twice daily for the first 14 days of a 21-day cycle, for a maximum of 6 cycles. The median age was 65 and the median Karnofsky performance score was 60; 23 patients had extensive disease and 7 limited disease. All 30 patients are evaluable for initial response and toxicity; 27 have responded, response rate 90%, 25 achieving a partial and 2 a complete response. Remissions range from 1 + to 9 + months. All patients developed alopecia. Gastrointestinal toxicity was mild. The median white cell and neutrophil counts in cycle 1 were 4.8 and 3.0 on day 15 and 3.4 and $1.8 \times 10^9 l^{-1}$ on day 22. There was no thrombocytopenia. The mean peak plasma concentration of etoposide after a 50 mg dose was 2.3 $\mu g\, ml^{-1}$ (range 0.9–3.9), compared with a mean peak value of 15.0 $\mu g\, ml^{-1}$ after a 2-hour infusion of 100 mg $m^{-2}$. Prolonged durations of administration of low dose oral etoposide are thus clearly very active in SCLC, yet bone marrow toxicity is mild. This study supports the hypothesis that the schedule-dependency of etoposide is related to the maintenance of low blood concentrations of drug.

### Epirubicin, cisplatin and 5-fluorouracil is effective treatment in advanced gastric cancer

D. Cunningham, A. Cahn, N. Menzies-Gow, R.D. Rosin, J. Mansi & H.A.F. Dudley

*Royal Marsden Hospital, Sutton, Surrey; St Mary's Hospital and The Central Middlesex Hospital, London. UK.*

This schedule was devised to reduce the myelosuppression related to 5-fluorouracil and theoretically to increase the amount of tumour topoisomerase II, one of the targets for the anthracyclines. Fourteen patients, mean age 56 (range 41–78), with histologically confirmed locally advanced or metastatic gastric carcinoma were treated with cisplatin (C) 60 mg $m^{-2}$ i.v., epirubicin (E) 50 mg $m^{-2}$ i.v. day 1 and 5-fluorouracil 200 mg $m^{-2}$ daily as a continuous i.v. infusion via a central line using an ambulatory pump for a maximum of 21 weeks (8 courses of CE). All patients had measurable disease and 2 had received prior chemotherapy. Performance status was 1 in 11 and 2 in 3. There were 9 PR and 1 CR (confirmed at laparotomy) with an overall response rate of 71%. A positive response to treatment was associated with improved PS in all patients. The median survival of responding patients had not been reached. Median WBC nadir was $3.4 \times 10^9 l^{-1}$ (range 1–6.8) and median platelet nadir $160 \times 10^9 l^{-1}$ (range 60–413). WHO grading of leucopenia was 4 gd 0, 3 gd 1, 5 gd 2, 2 gd 3 and platelets 10 gd 0, 2 gd 2, 2 gd 1. One patient had diarrhoea gd 2, 1 patient had rhinorrhoea, 2 patients complained of loss of taste and 3 patients had plantar-palmar erythema. Alopecia was gd 3 in 7, gd 2 in 3, gd 1 in 4. Overall, CEF was well tolerated on an out-patient basis with significant activity in gastric cancer and deserves further investigation in phase III studies.

### A phase II study of anthrapyrazole CI 941, a highly active new drug against advanced breast cancer

J.L. Mansi, I.E. Smith, D. Talbot, D. Button, I. Judson & A.H. Calvert

*The Royal Marsden Hospital, Fulham Road, London, SW3 6JJ, UK.*

CI 941 is one of a number of anthrapyrazole compounds synthesized in an attempt to produce a drug at least as active as adriamycin but less toxic. In particular, it does not possess the paraquinone grouping associated with free radical generation and thus has less potential for cardiotoxicity. In experimental murine tumours, it was shown to have activity similar to adriamycin and better than mitozantrone. In phase I studies its major toxicity was myelosuppression with a maximum tolerated dose of 55 mg $m^{-2}$. Fourteen patients (pts) with advanced breast cancer have so far received CI 941, 50 mg $m^{-2}$ by i.v. bolus, 3 weekly, in a phase II study. The median age was 55 (range 23–66), median ECOG performance status 1 (range 0–2); 6 pts had received previous chemotherapy, but not an anthracycline. Seven (64%) of 11 pts so far assessable for response (at least 2 courses) achieved an objective partial response, including 3/5 (60%) previously treated pts, and 4 (36%) stable disease (SD). Response by site: soft tissue 6/9; lung 3/3; liver 3/4; and bone 1/5. Toxicity (WHO) was minor: alopecia grade 1 in 10 and grade 2 in 4, nausea and vomiting grade 0 in 3, 1 in 5, and grade 2 in 6 pts, and lethargy in 8. One pt had a dose reduction because of a suspected neutropenic infection; there were no other dose reductions, no deaths and no evidence of cardiotoxicity as assessed by left ventricular ejection fraction. Median response duration has not been reached. These early results suggest that CI 941 is a highly active and well-tolerated new agent in advanced breast cancer and warrants further study.

### A comparison of response, survival and quality of life (QoL) using 2 doxorubicin (Dox) schedules of equal dose intensity in metastatic breast cancer

C.J. Twelves[1], A.J. Ramirez[1], M.A. Richards[1], P. Hopwood[2], J. Ferguson[2], W. Gregory[1], R. Swindell[2], W. Scrivener[1], J. Miller[2], A. Howell[2] & R.D. Rubens[1]

[1]*ICRF, Guy's Hospital, London; and* [2]*Christie Hospital, Manchester, UK.*

Doxorubicin is the single most active cytotoxic agent in patients (pts) with advanced breast cancer. This randomized study compared 2 Dox schedules of equal planned dose intensity in 59 pts. Twenty-eight received 75 mg $m^{-2}$ every 3 weeks (Dox q3w) × 4, and 31 received 25 mg $m^{-2}$ weekly (Dox q1w) × 12. Median age was 52 years (range 41–74); none of the pts had received prior chemotherapy for advanced disease.

The dose intensities administered for Dox q3w and q1w were similar (84% and 75% of that planned respectively). Response was assessed by UICC criteria in all pts. The response rate for Dox q3w was 50% and for Dox q1w 58% ($P > 0.05$). Median survival with Dox q3w did not differ from Dox q1w (8 vs. 6.1 months; $P > 0.05$). Treatment toxicity assessed by WHO criteria was the same with the 2 schedules. Quality of life was assessed at 0, 6 and 12 weeks in 54 of the pts using the Rotterdam Symptom Checklist (RSCL). In the 21 pts randomized to Dox q3w who completed all 3 RSCL there was a significant improvement in psychological adjustment ($P = 0.001$). No such improvement was seen in the 21 pts randomized to Dox q1w ($P = 0.48$). These differences in psychological adjustment between the treatment arms were also apparent for pts who completed treatment as planned. We conclude that although the two schedules were of equal dose intensity, efficacy and WHO toxicity, psychological adjustment was better in those receiving Dox every 3 weeks.

**Fludarabine in chronic lymphocytic leukaemia (CLL) and non-Hodgkin's lymphoma (NHL)**

J.S Whelan[1], C.L. Davis[1], S.A.N. Johnson[3], A.J. Norton[2], A.Z.S. Rohatiner[1] & T.A. Lister[1]

*[1]ICRF Department of Medical Oncology, and [2]Department of Pathology, St Bartholomew's Hospital, London; and [3]Musgrove Park Hospital, Taunton, Somerset, UK.*

Fludarabine (FLU) is a fluorinated analogue of adenine arabinoside which is resistant to metabolism by deamination while retaining cytotoxicity. It has been reported to have particular activity in low grade lymphoid malignancies. Twenty-seven patients (9 CLL, 18 NHL) were treated with FLU at $25\,mg\,m^{-2}$ by i.v. push daily for 5 days, repeated every 21–28 days. All patients had previously received both single agent and combination therapy. The median number of previous treatments was 3. Six of 9 patients with CLL are evaluable for response, 2 patients having concurrent second malignancies and one still in treatment. A partial response was achieved in 2/6, static or progressive disease in 3/6 and 1 patient with recurrent pulmonary infections died of pneumonia after 2 cycles of FLU, the lymphocyte count having fallen from $248 \times 10^9\,l^{-1}$ to $3.4 \times 10^9\,l^{-1}$.

Ten patients with follicular lymphoma have been treated, of whom 7 are evaluable for response and 3 are still receiving FLU; 5/7 had bone marrow infiltration, 2 as the only site of disease, 2/7 had stage III disease. PR was achieved in 3 patients, 2 had no response and 1 patient had progressive disease. One patient died of probable fungal infection after 2 cycles when clinical evidence of response was apparent. Seven out of 8 patients with diffuse lymphoma (6 lymphoplasmacytoid and 2 small cell centrocytic lymphomas in the Kiel classification) are evaluable, 1 patient currently being treated. All patients had stage IV disease. Complete remission was achieved in 1/7, no response was seen in 4/7 and 2 patients died, 1 of pulmonary embolus and the other of lymphoma. FLU has been well tolerated, the major toxicity being infection related to myelosuppression. One patient had an episode of disorientation soon after receiving treatment while in complete remission of lymphoplasmacytoid lymphoma after 3 cycles of FLU. No evidence of neurological deficit was found and CT scan and EEG were normal. Despite selection of patients who had been heavily pretreated, FLU was active in both CLL and follicular lymphoma. FLU seems to be only occasionally effective in diffuse lymphoma. Further studies are needed to define the therapeutic role of this agent earlier in the natural history of these diseases.

**Efficacy and safety of the non-steroidal antiandrogen, *Casodex*, in early clinical trials for advanced prostate cancer**

B. Waymont[1], G.R.P. Blackledge[1], C.J. Tyrell[2], M.R.G. Robinson[3] & J.L. Williams[4]

*[1]Clinical Trials Unit, Queen Elizabeth Hospital, Birmingham; [2]Plymouth General Hospital; [3]Pontefract General Hospital; and [4]Royal Hallamshire Hospital Sheffield, UK.*

*Casodex* is a non-steroidal antiandrogen which seems to be peripherally selective and is without the side effects of progestational antiandrogens. On this basis it is at present undergoing clinical trials for the treatment of advanced prostatic cancer. Initial pharmacokinetic studies indicate a half-life of about seven days so a once daily oral dose is suitable with better patient acceptability.

In a dose finding study, 174 patients with advanced prostatic cancer were recruited. The acid phosphatase at entry had to be more than twice normal and patients received *Casodex* for 12 weeks. Response, for this study, was defined as a 50% reduction in acid phosphatase at 8 weeks. At an initial dose of 10 mg daily, 11 of 39 patients (28%) responded. At a dose of 30 mg daily, 23 of 42 patients (55%) responded and at a dose of 50 mg daily, 61 of 93 patients (66%) responded. Objective response was also assessed using modified National Prostatic Cancer Project (NPCP) criteria after 12 weeks treatment. A partial response was evident in 23% of patients receiving 10 mg *Casodex* daily, 42% of patients receiving 30 mg daily and 45% of patients receiving 50 mg daily. Adverse reactions associated with *Casodex* therapy included breast swelling and tenderness in approximately 50% patients and hot flushes in approximately 20% patients receiving the 50 mg dose, due to pharmacological effects. Two patients were also reported to show an elevation in liver enzymes following 50 mg daily. No other serious side effects or significant gastrointestinal toxicity was noted. The efficacy and safety of the 50 mg dose is currently being evaluated in Phase II clinical studies and will be further assessed in randomized Phase III clinical studies.

**Pattern of intraperitoneal spread in epithelial ovarian cancer (EOC): a new prognostic factor?**

R. Varma, G. Blackledge, D. Luesley, K.K. Chan & K. Kelly

*Cancer Research Campaign Clinical Trials Unit, Queen Elizabeth Hospital, University of Birmingham, Edgbaston, Birmingham B17, UK.*

The majority of patients with EOC present when the disease has already spread beyond the ovaries and its management is based upon surgery to achieve complete macroscopic clearance followed by active platinum-containing chemotherapy. There are two distinct patterns of intraperitoneal spread, a predominantly bulky spread of disease or seedling spread throughout the peritoneal cavity, the prognostic implications of this are unclear. In an attempt to determine this a retrospective analysis was carried out on 107 patients with stage II–IV disease who were entered into a randomized study of EOC carried out by the West Midlands Ovarian Cancer Group from Nov '81 to Nov '86. The overall median survival of the study group was 20 months. Sixty-four patients were found to have predominantly bulky disease and 43 had seedling spread. Survival for the patients with bulky disease was significantly longer than for those with seedling disease, (O/E 0.76; $\chi_1{}^2 = 10.3$; $P = 0.001$). This difference remained significant after controlling for the effect of pathology, differentiation, age and performance status using Cox multiple regression analysis. The amount of residual disease following primary laparotomy was also highly significant ($\chi_2{}^2 = 40.8$, $P < 0.0001$). This study suggests that disease in the absence of seedlings has a favourable prognosis owing to the increased likelihood of optimal surgery in this group of patients. It is never possible to achieve complete macroscopic clearance in the patients with seedling spread. Since surgical resection was always attempted in this study, it is unclear whether this survival difference is an effect of surgery or due to disease biology. A randomized trial of surgery is under way to investigate this and other factors, such as the response to chemotherapy.

### Does tumour necrosis factor (TNF) determine the pattern of metastasis of ovarian cancer?

S.T.A. Malik, M.S. Naylor, A. Oliff & F.R. Balkwill

*Imperial Cancer Research Fund, Lincoln's Inn Fields, London WC2A 3PX, UK; and Merck, Sharpe & Dohme, West Point, Philadelphia, USA.*

We have examined the effect of intraperitoneal TNF therapy on three intraperitoneal human ovarian cancer xenografts in nude mice. Parameters studied included: **a**. survival of mice; **b**. analysis of peritoneal cell populations; **c**. post-mortem appearances; and **d** histological analysis of the peritoneum. The results show that while moderate dose, 1 μg TNF, increased the survival of mice bearing two out of three xenografts, it also promoted the adherence and establishment of peritoneal metastases. This effect was independent of the antitumour activity of TNF, as peritoneal micrometastases were also observed in the TNF-resistant xenograft. Attenuation of the marked neutrophil influx into the peritoneum seen after i.p. TNF, by pretreatment of mice with an anti-murine CR3 receptor antibody, did not affect tumour implantation. To examine the possible role of endogenous tumour production of TNF in determining metastasis, we have studied the effects of intraperitoneal injection of Chinese Hamster Ovary (CHO) cells transfected with the TNF gene, in nude mice. The pattern of metastasis was studied using standard histology, immunohistology, *in situ* hybridization, and localization of radiolabelled cells *in vivo*. Our results show that the CHO transfectants give rise to peritoneal, liver, and lung metastases, and that these can be specifically inhibited by anti-TNF antibody. Neutralization of TNF activity may therefore be a realistic therapeutic option in the treatment of some cancers.

### Growth stimulation of human ovarian carcinoma cell lines by 17 β-oestradiol or epidermal growth factor

S.P. Langdon, A.J. Crew & W.R. Miller

*ICRF Medical Oncology Unit, Western General Hospital, Edinburgh, UK.*

The mechanisms of growth control in human ovarian carcinoma cells are undefined. The majority of ovarian tumours contain receptors for oestrogen (ER) (Slotman (1988), *Anticancer Res.*, **8,** 147) and 35% contain receptors for epidermal growth factor (EGFr) (Bauknecht (1988), *Gynecol. Oncol.*, **29,** 147). It is feasible that oestrogen or EGF might modulate growth of these tumours.

The effects of 17 β-oestradiol ($E_2$) and EGF have therefore been tested against the ER + ve PEO4 and ER − ve PEO14 human ovarian tumour lines *in vitro*. Addition of $E_2$ (0.1 or 10 nM) to ER + ve PEO4 cells in phenol red-free RPMI 1640 containing 10% fetal calf serum stripped of oestrogen, markedly stimulated growth and this effect could be inhibited by the anti-oestrogen, tamoxifen (1 μM). Neither $E_2$, nor tamoxifen, at these concentrations had any measurable effect on the growth of the ER − ve PEO14 cell line. EGF at concentrations of 1 pM − 10 nM stimulated growth of PEO14 cells grown in either 0.5% or 5% serum-containing medium, or in serum-free medium (RPMI + hydrocortisone, insulin, transferrin and selenium). The PEO4 cell line was not stimulated by EGF under any of these conditions. These studies indicate that, in culture, human ovarian carcinoma cells may show sensitivity to either $E_2$ or EGF.

### Trophic responses of colorectal tumour cultures to TGFα IGF-I, IGF-II and gastrin

L.G. Durrant[1], S.A. Watson[1] & D.L. Morris[2]

*[1]Cancer Research Campaign Laboratories, University of Nottingham NG7 2RD; and [2]Department of Surgery, University Hospital, Nottingham NG7 2UH, UK.*

The factors controlling the growth of colorectal tumours are not well understood. We have previously shown that gastrin plays a central role in the stimulation of gut tumour proliferation. This study shows a synergistic response to gastrin and the growth factors TGFα, IGF-I and IGF-II in promoting tumour cell proliferation.

Colorectal tumours are disaggregated with collagenase to produce a highly viable single cell suspension of cells. These are then placed in culture in the presence of varying doses of gastrin or growth factors before assessing proliferation by incorporation of $^{75}$Se-selenomethionine. Four out of 9 tumours responded trophically to gastrin and TGFα alone but 6/9 responded to the combination of both, 4 with a synergistic increase in growth and 2 with an additive response. Four out of 9 tumours responded trophically to IGF-I and 3/4 tumours responded to IGF-II. There was a synergistic growth response to TGFα and IGF-I in 2 tumours and to IGF-I and gastrin in two tumours. The only tumour tested which failed to respond trophically to IGF-II, showed a synergistic response to the presence of IGF-II and gastrin. These results suggest that gastrin antagonists may be even more effective at inhibiting gastrointestinal tumour cell proliferation than would be predicted by the response of tumours to gastrin alone. Furthermore drugs such as the somatostatin derivative, *Sandostatin*, which prevents the secretion of gastrin and TGFα should be very effective at inhibiting colorectal tumour growth.

### Relationship between growth inhibition by cytokines (interferons and tumour necrosis factor) and receptor binding in human lung cancer cell lines

S.A.B. Jabbar & P.R. Twentyman

*MRC Clinical Oncology and Radiotherapeutics Unit, Hills Road, Cambridge CB2 2QH, UK.*

Interferons (IFNs) and tumour necrosis factor (TNF) bind to specific receptors on the cell surface. We have investigated the relationship between the responsiveness to cytokines in terms of growth inhibition and the extent of receptor binding in a panel of human lung cancer cell lines. Growth inhibition was assessed after 6–8 days of growth of cells in RPMI 1640 medium with the cytokines added at the beginning of this period. Receptor analysis was carried out using $^{125}$I Iodotyrosyl human recombinant IFN-γ or TNF-α (Amersham). Small cell lines POC, COR-L47 and COR-L88 showed 60, 50, and 10% growth inhibition respectively by 1 kU ml$^{-1}$ of IFN-γ. However, all 3 lines gave similar maximum $^{125}$I IFN-γ binding at 15 minutes. At 37°C, however, the rate of decrease of binding (presumably due to receptor internalization and degradation) was less in COR-L88 than in the other two lines. A multidrug resistant variant (COR-L23/R) of the large cell line COR-L23 showed greater sensitivity to growth inhibition by IFN-γ than the parent. Receptor binding of $^{125}$I IFN-γ was also 50% greater in the resistant than the parent line. Similarly, increased sensitivity to TNF-α in multidrug resistant variant MOR/R of adenocarcinoma line MOR was accompanied by increased binding of $^{125}$I TNF-α. Scatchard analysis showed that the number of receptors/cell was similar in MOR and MOR/R (6 k) but that the affinity constant is 50% higher in the latter. In six cell lines, the effect of IFN-γ pretreatment on $^{125}$I TNF-α was measured. In none of the cell lines was there a significant change in the binding pattern as a result of the pre-incubation, although in one cell line, growth inhibition by TNF-α was increased following IFN-γ pre-incubation. It appears unlikely from the data that the responsiveness of lung cancer cells to cytokines can be predicted from a simple measure of receptor binding. Cells showing significant specific binding can be quite refractory to growth inhibition by the cytokine in question.

### TGF-beta is implicated in the failure of tamoxifen treatment in breast cancer

A.M. Thompson[1,2], C.M. Steel[2], D.J. Kerr[3] & U. Chetty[1]

*[1]Department of Surgery, Royal Infirmary, Edinburgh EH3 9YW; [2]MRC Human Genetics Unit, Western General Hospital, Edinburgh EH4 2XU; and [3]Beatson Institute, Garscube Estate, Switchback Road, Glasgow G61 1BD, UK.*

The peptide growth factor transforming growth factor beta (TGF-beta) inhibits breast epithelial cell growth *in vitro*. The anti-oestrogen tamoxifen in these cells increases TGF-beta levels and is also inhibitory *in vitro*. Using densitometry of Northern blots the expression of TGF-beta mRNA has been quantified in breast tumour tissue from 45 untreated and 11 tamoxifen-treated patients. In the 56 tumours, TGF-beta mRNA expression was significantly higher in premenopausal women ($P = 0.05$) but was independent of oestrogen receptor status and other clinical and pathological parameters. Within tamoxifen-treated patients high expression was demonstrated in all 6 patients with progressive tumour growth whereas low levels were present in 4 of 5 with static tumour. These data suggest that failure of tamoxifen treatment in breast tumours is associated with high, rather than low, levels of TGF-beta, contrary to expectations from *in vitro* studies.

### Interleukin 6 is a growth factor for myeloid but not myeloma cells from human bone marrow aspirates *in vitro*

B.C. Millar, J.B.G. Bell, J.K. Joffe, A. Montes, J.L. Millar, T.R. Bradley, R. Gooding[1], P. Riches[1] & T.J. McElwain

*Institute of Cancer Research and Royal Marsden Hospital, Sutton, Surrey; and [1]Westminster Hospital, London, UK.*

Several groups have claimed that interleukin 6 (IL-6) is a growth factor for human myeloma cells *in vitro*. We examined bone marrow aspirates from 13 myeloma patients to determine whether rhuIL-6 affects the clonogenicity of myeloma *in vitro*. Myeloma cells from 11/13 patients produced colonies (MY-CFUc) *in vitro* in our assay system, which uses a heavily irradiated feeder layer of HL60 cells as an underlay in soft agar. The addition of rhuIL-6 (100 units per plate) resulted in 6/13 of the same samples producing myeloma colonies in this culture system. Furthermore, addition of anti-IL-6 (1 μg per plate) failed to inhibit MY-CFUc from 6/7 samples.

Conditioned medium from human peripheral blood mononuclear cells (PB-CM) contains approximately 4 ng $ml^{-1}$ IL-6 and is necessary for the growth and maintenance of the murine B9 hybridoma cell line, which will not replicate in medium containing rhuIL-6. Mononuclear cells from a second group of 10 myeloma patients were cultured in soft agar (0.3% final concentration) in a mixture of 50:50 PB-CM and fresh growth medium (α modification of Eagles MEM + 20% FCS + 1% BSA + 1.0 mM Gln). Nine of the 10 samples produced myeloid colonies which consisted of macrophages, monocytes and granulocytes. In no instance were MY-CFUc produced. Addition of anti IL-6 to this culture system reduced the number of myeloid colonies in 7/7 samples. Conditioned medium from the human bladder carcinoma cell line 5637, which is used as a source of granulocyte macrophage colony stimulating factor (GM-CSF), contains approximately 4 ng $ml^{-1}$ IL-6. Addition of anti IL-6 (0.5 μg 1.5 $ml^{-1}$) to cultures of GM-CFUc from normal donor bone marrow reduced the number of colonies by 80%. These data provide evidence that IL-6 is a growth factor which affects the proliferation of myeloid but not myeloma cells *in vitro*.

### The effects of prolonged treatment of patients with advanced cancer with low-dose subcutaneous interleukin 2 (IL2)

R.C. Stein[1], V. Malkowska[2], S. Morgan[2], C. Aniszewski[1], A. Galazka[3], D. Bevan[2], E.C. Gordon-Smith[2] & R.C. Coombes[1]

*[1]Clinical Oncology Unit and [2]Department of Haematology, St George's Hospital Medical School, London SW17 ORE, UK; and [3]Glaxo IMB, Geneva, Switzerland.*

IL2 has been shown to have useful activity in the treatment of some advanced cancers in phase II studies. Existing regimens involving i.v. administration are cumbersome and treatment toxicity is substantial, especially at high doses. We have investigated the effects of prolonged outpatient treatment with self-administered, low-dose IL2 given once daily by s.c. injection. Thirty patients with advanced cancer have been given 0.1, 1, 10 or 100 μg (16–16,000 units) IL2 (*Bioleukin*, Glaxo) daily for 8 weeks, 5 days weekly, followed by a 4-week observation period. No systemic side effects were observed at the 3 lower doses and only 3/13 patients required dose reduction from 10 μg daily for intolerance (fever, rash, nausea and vomiting, and lethargy). Minor skin reactions occurred at all doses. Immunological and clinical activity were observed at 100 μg daily. Immunological effects in 8 patients consisted of: a modest sustained lymphocytosis from week 2; eosinophilia in 6 patients; and a rise in natural killer (NK) and IL2-stimulated lymphocyte activated killer (LAK) cell activity to a mean of 2.1 and 2.0 times pretreatment levels ($P < 0.01$). Two of six patients with renal cell carcinoma treated with 100 μg daily had partial responses of durations 3+ and 9 months, and 2 had stable disease for >6 months. Low-dose, long-term s.c. IL2 is clinically and immunologically active and in comparison with other IL2 regimens it has only minor toxicity and is easy to administer. These characteristics make it suitable for study in the adjuvant setting.

### Interferon-$\alpha_{2b}$ (IFN-$\alpha_{2b}$) as initial therapy in combination with chlorambucil (CB) and as maintenance therapy in follicular lymphoma (FL)

C. Price, A. Rohatiner, W. Steward[2], J. Wright, A. Norton[1], D. Deakin[3], N. Bailey[4], G. Blackledge[4], D. Crowther[2] & T.A. Lister

*ICRF Department of Medical Oncology & [1]Department of Histopathology, St Bartholomew's Hospital, London EC1; Departments of [2]Medical Oncology & [3]Radiotherapy, Christie Hospital, Manchester; and [4]West Midlands CRC Clinical Trials Unit, Queen Elizabeth Hospital, Birmingham, UK.*

Since 1985 the combination of CB (10 mg daily; initially for 6 weeks, then alternating fortnights for 12 weeks) and IFN-$\alpha_{2b}$ (Schering-Plough; $2 \times 10^6$ units $m^{-2}$ three times weekly s.c. continuously for 18 weeks) has been compared in a randomized trial with CB alone (as above) in previously untreated patients with stage III and IV FL. Responding patients have subsequently been randomized to maintenance IFN-$\alpha_{2b}$ (M-IFN), or no further treatment (NT). To date, 111 patients have been treated and 104 are evaluable for response (57 CB, 47 CB + IFN-$\alpha_{2b}$), with a median follow up of 28 months. There was no significant difference in response rate. The major toxicity of the initial therapy was myelosuppression, which was more frequent with CB + IFN-$\alpha_{2b}$; 32 patients (65%) required a delay in treatment, or dose modification, vs. 10 (18%) with CB alone ($P < .01$). Five patients were intolerant of the systemic toxicity of IFN-$\alpha_{2b}$. There was no mortality related to treatment. Actuarial survival at 3 years is 75% for all patients, regardless of therapy. For the 68 patients who have entered the second phase of the trial, there was a significant difference in duration of remission in favour of M-IFN (median not yet reached vs. 9 months for NT, $P = .014$). Fewest relapses have been seen in patients who received IFN-$\alpha_{2b}$ in both phases of the study. Accrual to the trial continues; this preliminary analysis indicates that M-IFN may extend remission duration in FL.

### A phase II study of ifosfamide and α2 interferon (*Intron A*) in advanced non-small cell lung cancer

M.J. Lind[1], N. Thatcher[2], H. Gurney[2] & A. Kamthan[2]

[1]*University Department of Clinical Oncology, Newcastle General Hospital, Newcastle upon Tyne NE4 6BE; and* [2]*CRC Department of Medical Oncology, Christie Hospital, Manchester M20 9BX, UK.*

Forty-five patients with advanced non-small cell lung cancer who were symptomatic from their disease, or clinically progressing, were treated with a combination of $\alpha_2$ interferon (*Intron A*) and ifosfamide. Each patient received $\alpha_2$ interferon (*Intron A*) 3 megaunits three times a week until disease progression, or to a maximum of 12 weeks. In addition, patients received ifosfamide $1.5\,g\,m^{-2}$ given as a thirty minute i.v. infusion on days 1–5 with equidose mesna given as a 12-hour infusion. Courses were repeated three weekly until disease progression, or to a maximum of 4 courses. A total of 128 cycles of ifosfamide were administered to 45 patients. Haematological toxicity was generally mild with median nadir white blood cell counts as follows: $2.95 \times 10^9\,l^{-1}$ (course 1), $2.40 \times 10^9\,l^{-1}$ (course 2), $2.85 \times 10^9\,l^{-1}$ (course 3), $2.10 \times 10^9\,l^{-1}$ (course 4). Intravenous antibiotics were administered on five occasions. Non-haematological toxicity was mild and reversible. There were no instances of haemorrhagic cystitis, and only two episodes of ifosfamide encephalopathy. Toxicity attributable to interferon consisted of mild myalgia and pyrexia in 10% of patients. There were two complete responders and seven partial responders giving an objective response rate of 20%. Twenty-three patients had stable disease, eight progressive disease and there were three early deaths. The median survival following the start of therapy was 8 months. In conclusion, this study does not demonstrate potentiation of the antitumour action of ifosfamide by $\alpha_2$ interferon (*Intron A*).

### The role of oestrogen receptors and C-erbB$_2$ in the long term prognosis of breast cancer

J.H.R. Winstanley, S.M. Holt,. R.S. Rudland, B.R. Baraclough, A.M. Platt-Higgins, W.D. George, K. Griffiths, R. Nicolson & T.G. Cooke

*University Departments of Surgery & Biochemistry, Liverpool & Glasgow, and the Tenovus Institute, Cardiff, UK.*

Oestrogen receptor status was one of the earliest cellular markers of prognosis to be described in breast cancer and our group was one of the first to observe its short term prognostic significance. Since then a number of other markers have been described, one of the most recent being the proto-oncogene C-erbB$_2$ whose protein product is expressed at the cell surface and which shares significant sequence homology with the EGFR. Initial short term studies of both mRNA and cell surface protein have indicated an expression of up to 30% and an association with tumours of poor prognosis.

We have re-evaluated our original group of patients, in whom oestrogen receptor was studied, to assess the long term significance of oestrogen receptors and, using an immunohistochemical technique, evaluated the expression and significance of C-erbB$_2$ in the same group of patients. Oestrogen receptor status was evaluated in 708 patients with stage I and II breast cancer treated between the years 1975 and 1981 with either modified radical mastectomy or simple mastectomy with axillary sampling. We used a radiolabelled ligand binding technique, and paraffin sections from 423 of this group were stained using an immunoperoxidase technique with the 21N polyclonal C-erbB$_2$ antibody. Although a highly significant survival advantage was seen in the oestrogen receptor positive patients ($\chi^2 = 6.69$; $P = <0.009$) this was entirely accounted for by a short term improvement in stage II disease. Presence of C-erbB$_2$ though was associated with significantly poorer survival which was present throughout the study and affected both stage I and II disease. ($\chi^2 = 7.674$; $P = <0.0056$). Although ER status can now only be regarded of short term significance in stage II disease, C-erbB$_2$ significantly predicts long term prognosis for both stage I and II disease.

### Allele loss from chromosome 17p and p53 gene expression is important in the pathogenesis of breast cancer

A.M. Thompson[1,2], J. Prosser[2], G. Cranston[2], C.M. Steel[2], U. Chetty[1] & W.R. Miller[1]

[1]*Department of Surgery, Royal Infirmary, Edinburgh EH3 9YW; and* [2]*MRC Human Genetics Unit, Western General Hospital, Edinburgh EH4 2XU, UK.*

Allele loss from the short arm of chromosome 17 (17p) and mutations of the p53 gene on 17p from tumour suppressor gene to oncogene have been demonstrated in colorectal and lung cancer. The aim of the present study was to determine whether similar abnormalities may be detected in breast cancer. In 80 primary breast tumours we examined allele loss from 17p13 with Southern blots of paired blood and tumour DNA, p53 gene mRNA expression by laser densitometry of Northern blots and used the polymerase chain reaction and DNA sequencing to identify p53 gene mutations. 17p allele loss was demonstrated in 42 of 76 informative tumours (64%). No p53 mRNA was demonstrated in 33 of the 76 tumours, whereas there was over-expression of p53 mRNA compared to normal breast tissue in 24 tumours. Allele loss with probe YNZ 22.1 correlated with low oestrogen receptor protein ($P = 0.029$) and over-expression of p53 mRNA ($P = 0.042$). Mutations in exons 5 and 6 of the p53 gene were detected in some tumours with over-expression of p53 mRNA. These data suggest that chromosome 17p is important in breast cancer. Absence of p53 tumour suppressor gene expression or deletion of a regulatory locus (adjacent to YNZ 22.1) allowing over expression of mutant, oncogenic p53 are proposed as key events in the pathogenesis of breast cancer.

### An immunocytochemical method for detection of progesterone receptors in breast tumours

E. Anderson, K. Healy, L. Jones & A. Howell

*Departments of Clinical Research and Medical Oncology, Christie Hospital and Holt Radium Institute, Manchester M20 9BX, UK.*

An immunocytochemical method for detecting progesterone receptors (PR) has been developed and used to determine the PR status of 325 breast tumour specimens too small for ligand binding assay. Briefly, after blocking endogenous peroxidase activity, 5 µm fixed, frozen sections were incubated with the primary antibody (Transbio, France) overnight at 4°C. Binding of primary antibody to the receptor was visualized using peroxidase-conjugated secondary and tertiary antibodies followed by the chromogen (diaminobenzidine). Finally, the sections were lightly counterstained, dehydrated and mounted. The oestrogen receptor (ER) status of all the tissue samples was determined using the ER-ICA kit (Abbott Labs). Receptor content was expressed as the percentage of tumour cells that showed specific staining and a level of 5% was used as the cut-off between positive and negative tumours. Of the 325 samples assayed, 45% were ER+/PR+, 27% were ER−/PR− with 15% and 13% being ER+/PR− and ER−/PR+ respectively. In a subgroup of 51 tumours, PR was additionally assayed by the iso-electric focusing (IEF) method previously used in this laboratory for receptor assessment in small tumour samples. Whilst there was a statistically significant correlation between the two methods ($P<0.001$ by Spearman's Rank Correlation test), it was clear that the IEF method seriously underestimated the proportion of PR+ tumours (49% vs 69%; $P<0.001$ by $\chi^2$ test). To our knowledge this is the largest group of tumours to date to have their PR content assayed by an immunocytochemical method. We conclude that the PR immunocytochemical assay is a relatively simple and reliable technique and is the method of choice for the estimation of PR in small breast tumour samples.

### Prognostic factors in breast carcinoma

D.J. Hehir, A. McCann, K. Cronin, D. Carney, P. Dervan, W.P. Hederman & S.J. Heffernan

*Departments of Surgery, Oncology, and Pathology, Mater Hospital, Dublin 7, Ireland.*

Nucleolar organizer regions are loops of DNA which possess ribosomal RNA genes, and are readily demonstrable by silver staining. The argyrophilia can be assayed on routinely processed histological tissue by direct counting (Ag-NORs), and is an indicator of mitotic activity (Smith & Crocker (1988), *Histopathology*, **12,** 113). Recent studies have demonstrated significantly increased Ag-NORs in malignant vs benign breast tumours (Giri *et al.* (1989), *J. Pathol.*, **157,** 307). The aim of this study was to correlate Ag-NOR activity with survival in a group of women previously treated for breast carcinoma and currently under review. Fifty patients treated by partial mastectomy and radiotherapy were serially reviewed for signs of recurrent disease. [Mean 60 ± 94 months]. Original paraffin-embedded histological specimens were sectioned, stained and coded. Ag-NOR's were counted in 100 cells per slide.

| **Results** | a<br>*Disease recurrence @ 36 months* | | b<br>*Disease free @ 60 months* | | c<br>*Non Survivors* | |
|---|---|---|---|---|---|---|
| Ag-NORs | 24.8 | 6.7 | 19.7 | 7 | 27 | 6.7 |
| | **a** vs **b** $P<.05$ | | **b** vs **c** $P<.01$ | | | |

Our findings suggest that nucleolar organizer regions are of prognostic significance in breast carcinoma. They may have clinical application in dictating therapeutic options for breast carcinoma.

### Mutant p53 is a common genetic abnormality in human breast cancer and associated with EGF receptor and neu expression

A.L. Harris[1], E. Horak[2], K. Smith[1], L. Hurley[2], S. LeJeune[1], M. Greenall[3] & D. Lane[4]

*[1]ICRF Molecular Oncology Laboratory, Institute of Molecular Medicine, John Radcliffe Hospital, Headington, Oxford OX3 9DU; [2]Nuffield Department of Pathology, John Radcliffe Hospital, Headington, Oxford OX3 9DU; [3]Nuffield Department of Surgery, John Radcliffe Hospital, Headington, Oxford OX3 9DU; and [4]ICRF Clare Hall, Blanche Lane, Potter's Bar, South Mimms, Herts EN6 3LD, UK.*

Deletions of chromosome 17p have been reported in a high proportion of breast cancers. To investigate the role of p53 activation in primary human breast cancer and follow up the observation of frequent 17p deletions, we used a monoclonal antibody that recognized a specific isotope expressed by mutated forms of p53, to stain primary breast cancers. This has been correlated with the expression of other gene markers that are associated with a poor prognosis, EGFr and neu; 23/29 (80%) of EGFr positive tumours expressed mutant p53, and only 7/25 EGFr negative tumours (27%, $P<.05$). There was no association with lymph node status or ER status, but there was an association with poorly differentiated tumours. Neu was expressed in 6/16 p53 positive tumours with cytoplasmic staining, whereas only 1/18 p53 negative tumours had such staining.

### The anti-metastatic gene *NM23*: its expression in human breast cancers

C. Hennessy[1], J.A. Henry[2], F.E.B. May[2], B.R. Westley[2], B. Angus[2] & T.W.J. Lennard[1]

*Departments of [1]Surgery and [2]Pathology, University of Newcastle-upon-Tyne, Newcastle-upon-tyne, NE2 4HH, UK.*

Recently attention has been focused on the genetic basis of the metastatic phenotype. The *ras* oncogene and its protein p21 are implicated in the complex process of tumour metastasis. If tumour metastasis could be accurately predicted, the therapeutic options for patients might be optimized, which may possibly lead to improved long term survival. A recently described antimetastatic gene may provide part of the answer. *nm*23 expression has been investigated in animal experimental systems and found to have an inverse correlation with tumour metastatic potential. When its expression in 27 human primary breast cancers, 10 by Northern blots and 17 by *in situ* hybridization, was assessed it showed strong correlation with lymph node involvement (Bevilacqua *et al.* (1989), *Cancer Research*, **49,** 5185). We have assessed the level of *nm*23 mRNA expression in a series of 59 human primary breast cancers by Northern transfer. Hybridization was carried out to RNA using a digested fragment of the murine *pnm*23-1 plasmid, with hybridization to the human cell line HeLa RNA as internal control. Hybridization to a mRNA band of 0.8 kb was identified, confirming the observations of other authors. After quantitative analysis, by scanning densitometry, the *pnm*23 mRNA levels were correlated with lymph node involvement. Of 35 tumours from lymph node positive patients, 28 (80%) demonstrated low levels of expression, whereas only 9 out of 20 tumours (37.5%), from lymph node negative patients, had similarly low levels. When the distribution was assessed by the $\chi^2$ test it showed that these differences were significant, ($\chi^2 = 12.47$ with $P<0.02$). *nm*23 is a useful indicator of the metastatic potential of human cancers and requires further study in relation to the long term survival of these patients.

### Argyrophil nucleolar organizer regions in colorectal cancer

Z. Rayter[1], P. Surtees[1], G. Tildsley[2] & C. Corbishley[2]

*[1]Department of Surgery, St Peter's Hospital, Chertsey and [2]Department of Pathology, St George's Hospital, London SW17, UK.*

Argyrophil nucleolar organizer regions (AgNORs) are increased in a variety of malignant cells compared with their normal counterparts. A recent study has claimed that AgNORs have prognostic value in Dukes C colorectal cancer (Moran *et al.* (1989), *Br. J. Surg.*, **76,** 645). We have studied the AgNOR counts in 95 tumours resected from patients in whom a minimum 5-year follow-up was available. In 71 specimens, adjacent normal mucosa was also examined. Formalin-fixed paraffin wax-embedded sections were stained according to the method of Crocker & Nar [(1987), *J.*

*Pathol.*, **151**, 111]. AgNORs were counted in 100 normal and 100 carcinoma cells, and the results expressed as AgNORs per cell. There was a significant difference between AgNORs per cell in tumour cells (median 1.92, range 1.42–2.95) compared with normal cells (median 1.46, range 1.10–1.80, $P<0.001$). There was no significant difference in AgNORs per cell in each Dukes stage, or between tumours which were well –, moderately –, or poorly – differentiated. Finally, there was no correlation of AgNORs with prognosis.

### Cyclic AMP binding proteins in human colorectal cancer and mucosa

A.W. Bradbury, D.C. Carter & W.R. Miller

*Department of Surgery, Royal Infirmary, Edinburgh EH3 9YW, UK.*

Cyclic AMP analogues inhibit growth and promote differentiation of human colorectal cancer cell lines *in vitro*. These effects are mediated through specific cyclic AMP binding proteins (cAMP-BP) (Tagliaferri *et al.* (1988), *Cancer Res.*, **48**, 1642). The aim of the present study was to measure levels of cAMP-BP in human colorectal cancer and mucosa. Specimens were obtained from 50 patients with colorectal cancer. In each case tissue was removed from tumour (T), adjacent (MA) and distant (MD) histologically benign mucosa. Cytosols were prepared by homogenization and high speed centrifugation. Levels of cyclic AMP-BP were then determined using a $^{3}$H-cAMP – competitive binding assay (Miller *et al.* (1988), *Br. J. Cancer*, **52**, 531) and results expressed as fmol cAMP bound $mg^{-1}$ cytosol protein. Tumours had significantly higher levels of cAMP binding (3453 ± SD 1772) than either MA (2286 ± 906) or MD (2666 ± 1086) ($P<0.0001$ by paired t-test). Binding was also significantly lower in MA than in MD ($P<0.001$ by paired t-test). Furthermore, binding was significantly influenced by tumour stage and grade. Levels were higher in Dukes B (n = 30, 3948 ± 1677) than Dukes C (n = 17, 2232 ± 856) tumours and well- and moderately-differentiated cancers (n = 35, 3654 ± 1724) as compared to those which were poorly differentiated or mucinous (n = 15, 2595 ± 1185). In conclusion, cAMP-BP are over-expressed in colorectal cancer compared with normal mucosa, particularly that adjacent to the tumour, and levels correlate with stage and grade of disease.

### Detection of allele loss in bronchial biopsies of lung tumours by use of the polymerase chain reaction

P.S. Ganly & P.H. Rabbitts

*MRC Clinical Oncology and Radiotherapeutics Unit, MRC Centre, Hills Road, Cambridge CB2 2QH, UK.*

One way of searching for tumour suppressor genes important in lung carcinoma is to look for frequently occurring chromosomal deletions. This may be done at the molecular level by comparing DNA obtained from paired tumour and normal patient tissue, and observing loss of polymorphic alleles by Southern blotting. The disadvantage to this approach is that it relies upon obtaining more tumour tissue than is provided by small bronchial biopsy samples, which do not usually yield sufficient DNA for conventional Southern blotting. The biopsy specimen is frequently the only material obtained during a patient's treatment course and therefore many tumours cannot contribute any information to this deletion mapping approach. We are, therefore, attempting to use the Polymerase Chain Reaction (PCR) to amplify DNA at loci from chromosome 3p which are commonly deleted in lung tumours. After sequencing around the Msp 1 polymorphic site at D3S2, primers were constructed which directed PCR amplification of a 473 bp product from D3S2, which could be cut at the polymorphic site to give two fragments of equal length. Heterozygotes thus had two alleles of 473 and 236 bp, which were analysed on agarose gels. Normal and tumour tissue from 15 patients who were informative (heterozygous) at D3S2, and had been studied for allele loss at this locus by Southern blotting, were studied using PCR. Results obtained by the two methods were identical. DNA was then successfully amplified from bronchial mucosal biopsies of 5 informative patients, and partial allele loss was detected in one who had large cell anaplastic carcinoma. This method may be useful in identifying allele loss in small samples, and thus permit study of many more patients than is now possible, facilitating location by deletion mapping of the tumour suppressor gene on 3p.

### Deletion mapping of chromosome 3 in small cell lung carcinoma patients

M.C. Daly[1], P.H. Rabbitts[1], N.M. Bleehan[1], P. Hasleton[2], S. Spiro[3], B. Carritt[4], R. Souhami[3] & J. Bergh[5]

[1]*CORU, MRC Centre, Hills Road, Cambridge;* [2]*Department of Pathology, Wythenshawe Hospital, Manchester;* [3]*Department of Histopathology, The Brompton Hospital, London;* [4]*MRC Human Biochemical Genetics Unit, University College, London, UK.; and* [5]*Department of Oncology, University of Uppsala, Uppsala, Sweden.*

The tumours of patients with small cell lung carcinoma (SCLC) frequently show deletions on the short arm of chromosome 3. The region of the chromosome commonly deleted may harbour a tumour suppressor gene. Despite reports of the loss of polymorphic alleles on 3p, the number of probes used in each case have been insufficient to allow the delineation of the deletion in molecular terms. We present the genotype analysis of 9 SCLC patients obtained using 14 probes which spanned the entire length of chromosome 3 and which identified 18 restriction fragment length polymorphisms. We established that 7 out of the 8 pairs of normal and tumour DNA samples, showed reduction to homozygosity in the tumour DNA at each locus that was informative for a 3p polymorphism. In addition, a single unmatched tumour sample was shown to be homozygous at every 3p locus tested. By contrast, 2 out of 3 informative patients plus the unpaired tumour sample retained heterozygosity at loci which mapped to the long arm of chromosome 3. The extent of the deletion varied among patients ranging from loss of part of the short arm to deletion of an entire homologue. One of the patients has proved particularly useful in setting a distal boundary to the deletion. This patient was heterozygous at both the THR (3p24) and D3S2 (3p14-p21) loci, but showed allelic loss at other loci on 3p proximal to band 3p21. Using comparative densitometric analysis, we have demonstrated that this patient contains 2 copies of the D3F15S2 (3p21) locus, the latter of which is almost consistently subject to allelic loss in SCLC tumours. In order to compare effectively the extent of the the deletion in this patient with those of other studies, further physical evidence is required for the relative localizations of probes within the band 3p21. By the use of a large repertoire of chromosome 3 specific probes, we have detected a very informative patient for defining the boundaries of the 3p deletion.

### Production of insulin-like growth factor -I (IGF-I) and IGF-I binding proteins by human lung tumours

J.G. Reeve, J.A. Payne & N.M. Bleehen

*MRC Clinical Oncology and Radiotherapeutics Unit, MRC Centre, Hills Road, Cambridge CB2 2QH, UK.*

The production of insulin-like growth factor I (IGF-1) and IGF-I binding proteins (BPs) by human lung tumours cell lines *in vitro* has been examined and the levels of these substances in the serum of lung cancer patients investigated. Radioimmunoassay and immunoradiometric assay were used to measure IGF-I in acid extracted cell-conditioned media and serum samples. For the detection of IGF-BPs in conditioned media and serum $^{125}$I-IGF-I was cross linked to proteins using disuccinimidyl suberate in the presence or absence of excess cold IGF-I. Samples were electrophoresed on 7–15% linear gradient gels and IGF-BPs detected by subsequent autoradiography. Whilst small cell lung cancer (SCLC) cell lines secreted both IGF-I and low molecular weight BPs (25–30 kDa), the non-small cell lung cancer cell (NSCLC) lines examined secreted BPs only. No evidence of increased serum IGF-I levels was obtained in a cohort of 52 lung cancer patients having SCLC and NSCLC histologies. In contrast, serum levels of IGF-BPs were markedly raised in 25 of 27 of lung cancer patients studied. The frequency with which elevated BPs are detected in lung cancer patients raises the possibility that IGF-BPs may be clinically useful tumour markers for both SCLC and NSCLC.

### Increased expression of mutant forms of the p53 oncogene in primary lung cancer

R. Iggo[1], K. Gatter[2], J. Bartek[1,3], D. Lane[1] & A.L. Harris[4]

*[1]ICRF Molecular Immunochemistry Laboratory, Clare Hall, Blanche Lane, Potter's Bar, South Mimms, Herts EN6 3LD, UK; [2]University Department of Pathology, John Radcliffe Hospital, Oxford OX3 9DU, UK; [3]Research Institute of Clinical & Experimental Oncology, Brno, Czechoslovakia; and [4]ICRF Oncology Unit, Molecular Oncology Laboratory, Institute of Molecular Medicine, John Radcliffe Hospital, Oxford OX3 9DU, UK.*

Lung cancer is the commonest fatal cancer in men in the western world. p53 is a tumour suppressor gene which maps to chromosome 17p, a common site of allele loss in lung cancer. Therefore, we examined primary lung cancer samples of the major histological types for alteration of p53 expression using new antibodies to p53. Immunohistochemical abnormalities in p53 expression were found in 28 out of 40 carcinomas, 82% of squamous tumours showing abnormal p53 expression, whereas p53 expression was undetectable in 7 carcinoid tumours and in all normal lung examined. We obtained direct evidence for homozygous expression of mutant p53 mRNA in representative carcinomas by using an asymmetric polymerase chain reaction (PCR) based mRNA sequencing strategy. This method allowed us to sequence the mRNA without any cloning step. All the mutations were G>T transversions resulting in missense mutations in evolutionarily conserved amino acids. Mutation of the p53 gene is the most frequently identified genetic change in human lung cancer and our results suggest that simple immunohistological methods can be used to provide strong evidence that such mutation has occurred.

### Triazene *N*-oxides, synthesis and antitumour activity of some triazene 1-oxides

P.M. Goddard & D.E.V. Wilman

*Drug Development Section, Institute of Cancer Research, Cancer Research Campaign Laboratory, Cotswold Road, Sutton, Surrey SM2 5NG, UK.*

The imidazoletriazene, DTIC is well established in melanoma therapy and CB 10-277 [1-(4-carboxyphenyl)-3,3-dimethyltriazene] is currently undergoing clinical investigation. These compounds require activation by oxidative metabolism to display their antitumour effect. As an extension of our work in this area, we have investigated the antitumour activity of some triazene 1-oxides, which may have alternative metabolic requirements.

We report here on the synthesis and antitumour activity of 3-(4-R-phenyl)-1-methyltriazene 1-oxides (I), 3-(4-carbamoylphenyl)-1,3-dimethyltriazene 1-oxide (II) and 1-(4-R-phenyl)-3,3-dimethyltriazene 1-oxides (III) towards the AdjPC6/A murine plasmacytoma; CB 10-277 is included for comparison.

| *Compound* | $LD_{50}$ | $ED_{90}$ | *TI* |
|---|---|---|---|
| I, $R = CONH_2$ | 330 | 15.5 | 21.3 |
| I, R = COOH | >400 | 270 | >1.5 |
| II, $R = CONH_2$ | 285 | 195 | 1.5 |
| III, R = H | 35.5 | 21 | 1.7 |
| III, $R = CONH_2$ | 141 | 18 | 7.8 |
| CB 10-277 | 180 | 11 | 16.4 |

### The iodine atom in 4-iodotamoxifen reduces the extent of metabolism by isolated rat hepatocytes

R. McCague, I.B. Parr & B.P. Haynes

*Drug Development Section, Institute of Cancer Research, Sutton, Surrey SM2 5NG, UK.*

The search for an analogue of tamoxifen (TAM) possessing improved *in vitro* activity, like 4-hydroxytamoxifen but lacking propensity to deactivating metabolic conjugation, led to 4-iodotamoxifen (4-IT). We have reported that this has improved oestrogen receptor binding and effectiveness as an inhibitor of MCF-7 breast tumour cell growth in culture compared to TAM (McCague *et al.* (1989), *J. Med. Chem.*, **32**, 2527). But what about its metabolism?

We have now found that after an hour's incubation with hepatocytes isolated from rats pretreated with phenobarbital 4-IT was metabolized (extent of 18%) four times more slowly than TAM (75%). Metabolites formed from 4-IT and measured by HPLC were modified by demethylation (5%), *N*-oxidation (5%), α-hydroxylation with demethylation (1%), and 4′-hydroxylation (c. 0.5%). All of the metabolites identified retained the iodophenyl ring. The iodine atom not only prevented metabolism in its vicinity, but also reduced side-chain modification, since tamoxifen gives demethylation and *N*-oxidation each at 15% (Parr *et al.* (1987), *Biochem. Pharmacol.*, **36**, 1513). The reduced rate of metabolism of 4-IT by rat hepatocytes is predictive of an increased duration of action *in vivo*, a feature which is likely to be beneficial in breast cancer treatment where sustained drug levels are needed.

### Molecular similarity as a basis for ATP-competitive kinase inhibition

C.E. Sansom, C.J. McCall & M.F.G. Stevens

*Department of Pharmaceutical Sciences, Aston University, Aston Triangle, Birmingham B4 7ET, UK.*

Tyrosine kinase activity is exhibited by a number of oncogene products. Uncontrolled expression of these proteins may lead to and help maintain the transformed state (Cromoglio *et al.* (1987), *Ann. N.Y. Acad. Sci.*, **511**, 256). Quercetin, genistein and amiloride are inhibitors of tyrosine kinases which are competitive with the phosphate donor, ATP (Kenyon & Garcia (1987), *Med. Res. Rev.*, **7**, 389). Using the crystal structure of ATP bound to phosphoglycerate kinase (Watson *et al.* (1982), *EMBO J.*, **1**, 1635) and semi-empirical molecular orbital calculations with MOPAC IV (QCPE 455; Stewart, *QCPE Bull.*, **3**, 43) we have constructed a model of a kinase-bound ATP complexed to two magnesium ions. Comparison of this structure with those of the known tyrosine kinase inhibitors has shown a significant similarity between the molecular surface of the adenine moiety and both the bicyclic portions of the flavone derivatives and the heteroylguanidine fragment of amiloride. The electrostatic potentials of ATP and its competitive inhibitors are similar about the 6-$NH_2$ group and about N3 of the adenine residue. These techniques may be applied to future crystal structures of ATP-dependent tyrosine kinases leading to a greater understanding of the specificity of ATP-competitive kinase inhibitors.

### Synthesis and biological properties of 2-phenylbenzothiazole derivatives

C.J. McCall & M.F.G. Stevens

*Pharmaceutical Sciences Institute, Department of Pharmaceutical Sciences, Aston University, Aston Triangle, Birmingham B4 7ET, UK.*

Molecular modelling studies have shown that 2-phenylbenzothiazoles have the potential to mimic both D-16726, an anticancer anti-oestrogen with a 2-phenylindole nucleus (von Angerer *et al.* (1985), *Eur. J. Cancer Clin. Oncol.*, **21**, 531), and genistein, a naturally occurring oestrogenic isoflavone. Genistein also inhibits tyrosine kinases (Akiyama *et al.* (1987), *J. Biol. Chem.*, **262**, 5592). A number of hydroxylated 2-phenylbenzothiazoles have been prepared and tested for oestrogen receptor binding affinity, epidermal growth factor receptor tyrosine kinase inhibitory activity and cytotoxicity against the 3T3 and ANN-1 cell lines. ANN-1 cells are derived from 3T3 cell transformed by the Ableson virus and express a greater than normal level of tyrosine kinase activity. Oestrogen receptor binding studies confirmed that dihydroxy-substituted 2-phenylbenzothiazoles can bind to the oestrogen receptor. 2-(4-hydroxyphenyl)-6-hydroxy-benzothiazole showed the highest relative binding affinity (RBA = 0.70). This value is low relative to oestradiol (RBA = 100) but comparable to that of genistein (RBA≈1). The compounds tend to become more cytotoxic with each additional hydroxy group but none show differential cytotoxicity towards ANN-1 or 3T3 cells.

### Chemistry and stereochemistry of non-classical lipophilic dihydrofolate reductase inhibitors

W.K. Chui

*Pharmaceutical Sciences Institute, Department of Pharmaceutical Sciences, Aston University, Aston Triangle, Birmingham B4 7ET, UK.*

4,6-Diamino-2,2-dialkyl-1,2-dihydro-1-(substituted phenyl)-*1, 3,5*-triazines exhibit antitumour activity by inhibiting the enzyme dihydrofolate reductase. It has been found that the inhibitory activity of these triazines depends on the position of the substituents on the aryl ring. *Ortho*-substituted triazine antifolates show reduced inhibitory activity (Kim *et al.* (1980), *J. Med. Chem.*, **23**, 1248). Large *ortho*-substituent restricts rotation about the C-N bond between the aryl and the triazine rings. This gives rise to chiral atropisomers which may exhibit different affinity for the enzyme. Such triazine antifolates have been synthesized by using the 'Three-component Method' (Modest (1956), *J. Org. Chem.*, **21**, 1). Energy barrier to rotation about the C-N bond has been calculated by molecular modelling technique. Enrichment of the chiral atropisomers has been attempted by diastereomeric salt formation and diastereomeric derivatisation. Separation of the diasteromeric atropisomers has been achieved by semi-preparative high performance liquid chromatography. The separated diastereomeric atropisomers were tested for its inhibitory action against DHFR.

### DNA damaging effects of the benzotriazine-*N*-oxides

N.S. Virk, J.H. Tocher & D.I. Edwards

*Chemotherapy Research Unit, Polytechnic of East London, Romford Road, London E15 4LZ, UK.*

The DNA damaging ability of a number of benzotriazine-di-*N*-oxides, a novel class of hypoxic cell cytotoxins/radiosensitizers, has been examined. The drugs were electrolytically reduced under anoxic conditions in the presence of *E. coli* DNA and a trace amount of OX174 DNA. The damage was assessed using a double transfection assay. Under oxic and anoxic non-reductive conditions all the drugs tested showed no or very little damage to DNA. However, under anoxic reductive conditions damage was observed. The lead compound, SR4233, was found to be the most effective drug with damage being enhanced at acidic pH. At pH 7 a lag phase of about 1.5 hours was observed before any significant damage occurred. Slight shoulders were also seen at pH 6 and 5, whereas at pH 4 no delay occurred. This shows that the damaging species is probably protonated. The reduction potential, $E_{1/2}$, increases, i.e. become less negative, and the pH is decreased and this correlates well with the increased damage observed under these conditions. The results indicate that these compounds could be useful as hypoxic cell cytotoxins or used as a basis to make more potent and more specific hypoxic cell cytotoxins/radiosensitizers.

### Electrolytic properties of the benzotriazine-*N*-oxides

J.H. Tocher, N.S. Virk & D.I. Edwards

*Chemotherapy Research Unit, Polytechnic of East London, Romford Road, London E15 4LZ, UK.*

The electrochemical characteristics of a range of benzotriazine di-*N*-oxides, important as new cytotoxic agents with a high specificity for hypoxic cells, have been examined by a number of techniques. Reduction of the *N*-oxide groups results in the formation of the parent benzotriazine heterocycle, which is also redox active, involving 4- and 2-electron additions respectively. At alkaline pH these separate stages can be identified, but at acidic conditions only a single step is observed. The reduction potentials are found to shift to less negative values i.e., the drug shows an increased electron affinity, as the pH is lowered. This trend can be described by an equation of the form E = apH + b. The mono-*N*-oxide shows only a single reduction step, although the reduction of the *N*-oxide group can be identified, giving the zero-*N*-oxide. This is also confirmed by coulometric measurements. The variation of the mono- and zero-*N*-oxides with pH is identical. The importance of pH in the biological activity of the di-*N*-oxides is illustrated by an increased DNA damaging capability of the reduced drug at acidic pH.

### Studies on the novel *Distamycin* compound FCE 24517 with respect to DNA interactions and sensitivity to alkylating agents

H. Coley, M. Broggini & M. D'Incalci

*Istituto di Ricerche Farmacologiche Mario Negri, Via Eritrea 62, 20157 Milan, Italy.*

FCE 24517, the benzoic acid mustard derivative of *Distamycin A* (Dist A) has previously been shown to possess broad spectrum antitumour activity, in marked contrast to the lack of activity seen for the latter. Unlike most alkylating agents the N7 position of guanine in isolated DNa is not the target for the alkylating moiety of FCE 24517. Present studies indicate the possibility of alkylation on the N3 positon of adenine. To study any concomitant alteration of the DNA major groove due to the presence of the minor groove binders Dist A or FCE 24517, DNA footprinting studies were carried out. Major groove alkylating agents used were uracil mustard (UM), quinacrine mustard (QM) and a tertiary amine analogue of dabis maleate 1,4,bis(2'-chloroethyl)-1, 4-diazabicyclo (DM). Maxam-Gilbert DNA sequencing gels demonstrated a marked difference between the patterns of alkylation due to QM in the presence of either FCE 24517 or Dist A. Similar effects were seen with Um and DM and these results are in some agreement with those of Hartley *et al.* (*Biochemistry*, in press) using Dist A. With the purpose of evaluating the relevance of these DNA sequence-specific studies, we plan to evaluate how the combined use of minor groove binders with alkylating agents can modify their cytotoxicity in cell lines with varying degrees of sensitivity to the alkylating agents.

**Potentiation of the cytotoxicity and DNA damaging effects of VP16 by pretreatment with non-cytotoxic concentrations of arabinosyl cytosine**

C.M. Chresta, R. Hicks, J.A. Hartley & R.L. Souhami

*Department of Oncology, University College & Middlesex School of Medicine, 91 Riding House Street, London W1P 8BT, UK.*

Pretreatment of the human lymphoblastoid cell line CCRF-CEM with 0.02 μM araC enhances both the cytotoxic and DNA damaging effects of etoposide (VP16). This concentration of araC is itself non-cytotoxic and results in no detectable DNA damage, as measured by alkaline elution. Maximum potentiation of etoposide cytotoxicity and DNA damage was observed after 48 hours continuous treatment with araC. Cytotoxicity of VP16 is increased approximately 2.5-fold ($IC_{90}$ 19 ± 3.6 μM for control cells; $IC_{90}$ 7.75 ± 1.8 μM for araC pretreated cells). DNA damage is increased to a similar degree. Treatment of control CEM cells with 1 μM VP16 for one hour results in a single strand break frequency of 158 ± 36 rad equivalents this frequency is increased to 330 ± 42 rad equivalents by pretreatment with 0.02 μM araC for 48 hours. The DNA protein cross link frequency is similarly increased, a one hour incubation with 10 μM VP16 resulting in a cross link frequency of 644 ± 130 rad equivalents in control cells and 1809 ± 89 rad equilavents in araC pretreated cells. Cell cycle analysis demonstrated that 48 hours araC pretreatment resulted in accumulation of cells in late $S/G_2M$. Enhancement of VP16 activity was also observed with 24 hour araC pretreatment, at which time the majority of cells were in early S phase, however, the potentiation was not as great. Removal of araC allows cells to return to a normal cell cycle distribution within 24 hours and potentiation of VP16 activity is then lost. All DNA strand breaks were protein concealed, suggesting a mechanism involving topoisomerase II.

**DNA interstrand crosslinking and sequence selectivity of dimethanesulphonates**

M. Ponti[1], R.L. Souhami[1], B.W. Fox[2] & J.A. Hartley[1]

*[1]Department Oncology, University College & Middlesex School of Medicine, London; and [2]Department Exp. Chemotherapy, Paterson Institute Cancer Research, Christie Hospital, Manchester, UK.*

Members of the homologous series of dimethane sulphonic acid esters of general formula $H_3C.SO_2.O.(CH_2)_n.O.SO_2.CH_3$ have been tested for their ability to produce DNA interstrand crosslinking (ISC) and DNA sequence selectivity of guanine-N7 alkylation. In a sensitive crosslinking gel assay the efficiency of ISC formation, dependent on the ability of the alkylating moiety to span critical nucleophilic distances within the DNA, was found to be Hexa-DMA (n = 6) > MDMS (n = 1) > Octa-DMS (n = 8) > busulphan (n = 4). The cross-linking produced by MDMS was not due to either of its hydrolysis products, formaldehyde or methane sulphonic acid (MSA). In contrast, the extent of monoalkylation at guanine-N7 as determined by a modified DNA sequencing technique was found to be busulphan > Hexa-DMS >> Octa-DMS, with little overall sequence selectivity compared to other chemotherapeutic alkylating agents such as nitrogen mustards. MDMS at the same concentration showed extensive depurination as a result of the release of MSA. At lower doses however, where depurination was negligible, a single strong site of guanine-N7 alkylation was observed in a guanine-rich 276 base pair fragment of pBR322 DNA in the sequence 5'-ATGGTGG-3' which was not produced by either formaldehyde or MSA. Thus structurally similar alkylating agents can differ in their type and extent of DNa monoalkylation and ISC, and in some cases (e.g. MDMS) produce monoalkylations with a high degree of selectivity.

**Thermal potentiation of CCNU in two clonal RIF-1 lines with different thermo- and chemosensitivity**

N. Mitsuhashi, D.J. Honess & N.M. Bleehen

*MRC Clinical Oncology Unit, Hills Road, Cambridge CB2 2QH, UK.*

A continuing problem in hyperthermia, as yet unaddressed for thermochemotherapy, is the calculation of isoeffect doses. The aim of this study is to compare *in vitro* isoeffect data for heat with CCNU in cell lines of differing thermo- and chemosensitivity. We have used two morphologically distinct tetraploid clones of the murine RIF-1 line. The first stage is characterization of the thermal responses of each cell line, including measuring the breakpoint in the time-temperature relationship and the slope of the curve above and below the break. This allows accurate calculation of EQ43, the equivalent time at 43°C for isoeffect. $EQ43 = \Sigma R(43\text{-}t)\Delta t$, where R is derived from the slopes of the time-temperature curve, and t is treatment temperature. Thermosensitivity profiles for both clones have been measured over the range 41–46°C, plating cells into 25 $cm^2$ flasks 16 hours before heat and counting surviving colonies 12 days later. PE values are 48 ± 10% for L16 and 90 ± 5% for L20. Results:

| *Clone* | *Breakpoint* | *R above breakpoint (± 2se)* | *R below breakpoint (± 2se)* |
|---|---|---|---|
| L16 | 44°C | 0.52 (0.48–0.57) | 0.24 (0.21–0.28) |
| L20 | 43°C | 0.51 (0.47–0.56) | 0.08 (0.07–0.10) |

R values above the breakpoint are essentially identical, and are typical of those found for other mouse cell lines. The difference in thermosensitivity between the lines is reflected in the higher breakpoint for L16 and its larger R value below the breakpoint. CCNU dose response curves for a one-hour treatment have been measured for both lines at 37°C and selected higher temperatures. Do values are presented below:

| *Temperature (°C)* | *Do for CCNU, 1 h (μg ml⁻¹) (± 2se)* L16 | L20 |
|---|---|---|
| 37 | 6.82 (6.19–7.58) | 3.09 (2.80–3.44) |
| 41 | 2.63 (2.35–2.99) | 1.34 (1.22–1.48) |
| 42 | 2.39 (1.84–3.40) | 1.75 (1.59–1.96) |
| 43 | 2.55 (2.42–2.70) | na |

At 37°C L20 is about twice as sensitive to CCNU as L16. This relative sensitivity appears to be maintained at higher temperatures. Surprisingly, the decrease in Do caused by heat appears not to be temperature dependent. In conclusion, this study shows that universal breakpoints and R values cannot be assumed for all cell lines, hence both must be measured for accurate estimation of isoeffect doses.

**Dipyridamole enhances increased intracellular dUTP and DNA strand breakage in A549 (human lung carcinoma) cells treated with CB3717**

N.J. Curtin[1], G.W. Aherne[2] & A.L. Harris[3]

[1]*Cancer Research Unit, University of Newcastle upon Tyne;* [2]*Department Biochemistry, University of Surrey; and* [3]*Clinical Oncology, ICRF, Oxford, UK.*

$N^{10}$-propargyl-5,8-dideazafolic acid (CB3717) is a potent thymidylate synthase (TS) inhibitor with antitumour activity (Jones *et al.* (1981), *Eur. J. Cancer*, **17**, 11). Dipyridamole (DP) enhances the growth inhibitory effect of CB3717 (Curtin & Harris (1988), *Biochem. Pharmacol.*, **37**, 2113) by inhibiting thymidine uptake and deoxyuridine efflux. We proposed that it would therefore increase intracellular deoxyuridine nucleotide pools. In this study, we have used a sensitive RIA (Piall *et al.* (1989), *Anal. Biochem.*, **177**, 347) to measure intracellular dUTP and alkaline elution to measure DNA strand breaks in A549 cells treated with CB3717 ± DP. In control cells and those treated with DP alone dUTP was virtually undetectable but increased in response to CB3717 in a dose and time related way: 3 μM CB3717 24 h = 46.1 ± 9.6; 30 μM CB3717 4 h = 9.6 ± 3.7 and 30 μM CB3717 24 h = 337.5 ± 37.9 pM dUTP $10^{-6}$ cells. DP further increased the dUTP in a dose responsive way: 3 μM CB3717 + 1 μM DP 24 h = 174.7 ± 57.7 3 μM CB3717 + 10 μM DP 24 h = 482 ± 80 pM dUTP $10^{-6}$ cells. DNA strand breakage also increased with CB3717 dose and exposure time and was further increased by DP. Nascent DNA was more sensitive to CB3717 ± DP than was mature DNA. Substantial strand breakage was seen in nascent DNA after only 4 h with 30 μM CB3717 + 1 μM DP. The finding that both intracellular dUTP and DNA strand breakage were coordinately increased with CB3717 alone and in combination with DP supports the hypothesis that thymineless death in mammalian cells can be mediated by uracil misincorporation into DNA.

**Comparative enzymology of the bioreductive metabolism of the aziridinyl dinitrobenzamide CB 1954: microsomal enzymes versus DT-diaphorase**

M.I. Walton & P. Workman

*MRC Clinical Oncology Unit, MRC Centre, Hills Road, Cambridge CB2 2QH, UK.*

CB 1954 [5-(aziridin-1-yl)-2,4-dinitrobenzamide] exhibits unusually high potency against the UK Walker 256 tumour. Recent results by Knox *et al.* ((1989) *Biochem. Pharmacol.*, **37**, 4661 & 4671) strongly suggest this results from metabolism of the drug to the highly toxic 4-hydroxylamine by the high DT-diaphorase activity present in the tumour cells. Since DT-diaphorase is an 'oxygen insensitive' obligate two electron (2e) donating enzyme, the results do not appear to explain the preferential cytotoxicity of CB 1954 under hypoxia versus aerobic conditions; also, a broader spectrum of metabolites is seen *in vivo*. As part of an 'enzyme-directed' approach to bioreductive development we have compared the range of metabolites and oxygen sensitivity for CB 1954 bioreduction as catalysed by DT-diaphorase-rich Walker and HT29 human colon carcinoma cells and C3H mouse liver microsomes. A high resolution HPLC assay was developed which allows separation of all the known metabolites of CB 1954. For the DT-diaphorase preparation we confirmed the 4-hydroxylamine as the sole metabolite. Its formation was inhibited almost completely by 100 μM dicoumarol and the rates were similar in air or nitrogen, confirming the proposed oxygen insensitivity. For equal activities of menadione reduction, the rat DT-diaphorase was 5 times more active than the human in catalysing CB 1954 reduction. The 4-hydroxylamine was also the predominant metabolite in mouse liver microsomes. But in addition we also detected appreciable amounts of the 2-hydroxylamine and the respective 2- and 4-amines. Both cytochrome P-450 and its reductase appear to be involved and the formation of all the reduced metabolites was inhibited completely in air. These results suggest that a range of enzymes may catalyse CB 1954 bioreduction *in vivo* and that therapeutic selectivity may be governed by their relative activities and 'oxygen sensitivities' in normal and malignant tissues.

**Novel, potential bioprotection pathway for the developmental hypoxic cell cytotoxin SR 4233: catalysis by DT-diaphorase**

M.I. Walton & P. Workman

*MRC Clinical Oncology Unit, MRC Centre, Hills Road, Cambridge CB2 2QH, UK.*

The cytotoxin SR 4233 (3-amino-1,2,4-benzotriazine-1, 4-dioxide) exhibits unusually high selective killing of hypoxic compared to aerobic cells, and is the lead compound in the development of this new class of drug for potential use against solid tumours in man. The most likely mechanism of action involves single electron (1e) reductive bioactivation to a short-lived oxidizing radical which abstracts hydrogen from DNA, resulting in single and double strand-breaks. Hypoxic cell selectivity results from the reversal of 1e radical production by molecular oxygen. It follows that direct 2e reduction to the single N-oxide will bypass 1e bioactivation and will be bioprotective. We and others have shown previously that the major stable metabolites found in the tumour and tissues of mice *in vivo* are the 1-oxide SR 4317 (2e product) and the parent benzotriazine SR 4330 (4e product). We have also shown that the enzyme preparations from mouse tissues form predominantly SR 4317, e.g. 96% in liver microsomes and cytosol under nitrogen. We have now investigated the role of the obligate 2e donating flavoprotein DT-diaphorase as a potential bioprotecting enzyme. Rat UK Walker tumour cells were used as a rich source of DT-diaphorase. We found that SR 4233 was readily reduced by Walker cell extracts under air with NADH as cofactor. Interestingly, and in contrast to other preparations, the major product was not the 2e product (10%) but the 4e product SR 4330 (90%). Its formation was inhibited 82–95% by 100 μM dicoumarol. We believe this to be the first report of N-oxide reduction by DT-diaphorase. The predominant formation of SR 4330 suggests that the drug molecule remains bound to the enzyme active site to undergo 2 rounds of 2e reduction. We propose further that metabolism of SR 4233 by DT-diaphorase may potentially represent an important and novel bioprotection pathway *in vivo*.

**Lack of correlation between cisplatin sensitivity, drug uptake, DNA binding, induction and removal of adducts and glutathione metabolism in a panel of human tumour cell lines**

S.A. Shellard, P. Bedford, L.K. Hosking, A.M.J. Fichtinger-Schepman & B.T. Hill

*Cellular Chemotherapy Lab., I.C.R.F., London WC2A 3PX, UK.*

The mechanisms associated with the expression of differential cisplatin sensitivities in a panel of human tumour cell lines have been studied. We have investigated drug uptake and DNA binding, induction and removal of Pt-DNA adducts, and total glutathione levels and related enzyme activities in two testicular (SuSa & 833K), one bladder (RT112) and 4 ovarian cell lines (TR175, TR170, JAT & SK-OV-3). The $IC_{50}$ values for a one-hour *in vitro* exposure to cisplatin ranged from 0.11-4.4 μg $ml^{-1}$ with the two testicular cell lines and one of the ovarian lines (TR175) proving most sensitive and the RT112 bladder and SK-OV-3 ovarian tumour cell lines proving most resistant. These differences in cytotoxicity were not correlated with either differential drug uptake or drug-DNA binding. However, increased total glutathione levels were associated with the most resistant cell lines. Platinum-DNA adducts were measured using polyclonal antisera in a competitive ELISA either immediately following a one-hour cisplatin exposure, or 18 hours later. Differential induction and removal of Pt-DNA adducts does not appear to correlate with cisplatin sensitivity in these seven lines. For example, both SuSa cells and TR175 cells were highly sensitive to cisplatin yet the SuSa cells appeared deficient in the repair of Pt-AG and Pt-GG adducts, whilst the TR175 cells appeared proficient in the removal of all 4 adducts. In summary, therefore, no clear pattern has emerged when trying to correlate these parameters in this range of 'wild type' cell lines, suggesting not only that multifactorial mechanisms are operating, but also that different mechanisms appear to predominate in the different cell lines.

### Aphidicolin does not inhibit repair of cisplatin-induced DNA damage in a human ovarian cancer cell line

W.C.M. Dempke[1], S.A. Shellard[1],

A.-M.J. Fichtinger-Schepman[2] & B.T. Hill[1]

[1]*Imperial Cancer Research Fund, Cellular Chemotherapy, PO Box 123, London WC2A 3PX, UK; and* [2]*Medical Biological Laboratory TNO, PO Box 45, 2800 AA Rijswijk, The Netherlands.*

The recently established human ovarian cancer cell line JA-T, derived from a previously untreated patient (*Int. J. Cancer*, 1987, **39**, 219) expresses intermediate sensitivity to cisplatin. This cell line has been used to study the effects of aphidicolin (AG), a specific competitive inhibitor of DNA polymerase alpha, on the formation and removal of four platinum-DNA adducts. For the measurement of platinum-DNA adducts, logarithmically-growing cells were exposed to cisplatin ($10\,\mu g\,ml^{-1}$) alone or in combination with AG ($5\,\mu g\,m^{-1}$), which was added one hour before and during cisplatin treatment. After drug exposure for one hour cells were washed and harvested immediately, or medium (with or without AG) was added for another 18 hours. DNA adducts were then measured immunochemically. No differences between the initial levels of DNA platination and of specific DNA adducts in the presence or absence of AG were observed. Following 18 hours post-treatment incubation, the JA-T cells were shown to be proficient in the repair of all these platinum-DNA adducts. However, total platination and the levels of specific DNA adducts decreased by similar rates with or without AG treatment, indicating that AG does not alter the formation and removal of cisplatin-induced DNA adducts. In addition, growth-inhibition data for JA-T cells have provided some evidence that AG does not modify cisplatin-induced cytotoxicity. These data indicate that this non-cytotoxic concentration of AG ($5\,\mu g\,ml^{-1}$) does not modulate repair of cisplatin-induced intrastrand crosslinks in this repair proficient ovarian carcinoma cell line.

### Characterization of VP-16 resistance in a human testicular teratoma cell line

L.K. Hosking, R.D.H. Whelan & B.T. Hill

*Cellular Chemotherapy Laboratory, Imperial Cancer Research Fund, London WC2A 3PX, UK.*

Etoposide (VP-16) is one of the most effective antitumour agents in the treatment of testicular cancer. However, its clinical efficacy is limited by the development of drug resistance. To determine possible mechanisms of VP-16 resistance, we have derived a panel of resistant sublines designated VPC2, VPC3 and VPC4 from a human testicular teratoma cell line, SuSa, by continuous *in vitro* exposure to increasing VP-16 concentrations. These sublines exhibited 9-, 21- and ~40-fold levels of VP-16 resistance respectively, and also proved cross-resistant to adriamycin, vincristine and cisplatin, as determined by colony formation in soft agar. Resistance indices were defined by comparing $IC_{50}$ values (concentrations reducing survival by 50% following a 24-hour drug exposure) of parental cells and VP-16 resistant sublines. Overexpression of P-glycoprotein was identified in the two most highly resistant sublines (VPC3 and VPC4), as judged by Western blot analysis using the C219 monoclonal antibody. No differences in tritiated-VP-16 accumulation or modifications of total glutathione content, glutathione S-transferase, glutathione reductase or glutathione peroxidase activities were observed in any of the sublines compared to parental cells. However, the GST pi isozyme is constitutively expressed in all these SuSa lines. To investigate further this resistance we are currently assessing VP-16-induced DNA single-strand breakage, using the technique of alkaline elution, and possible alterations in topoisomerase II, in terms of P4 DNA unknotting activity.

### The effect of verapamil on the antitumour action of mitozantrone and doxorubicin against renal carcinoma cells *in vitro*

M.O. Symes[1], T. Lai[1], A.P. Morgan[1] & P.J.B. Smith[2]

[1]*University Department of Surgery and* [2]*Department of Urology, Bristol Royal Infirmary, Bristol BS2 8HW, UK.*

Surgical nephrectomy specimens were obtained from 12 patients with renal carcinoma, 7 at stage II (confined to the kidney) and 5 at stage III (extension beyond the kidney without metastases). Following digestion of the solid tumour with collagenase type II and DNase, the mixed cell suspension obtained was centrifuged on a Nycodenz[R] column to separate the carcinoma cells. Carcinoma cells were cultured with increasing concentrations of mitozantrone (MIT) (Lederle) or doxorubicin (DOX) (Farmitalia) ± verapamil (Verap.) (*Cordilox*, Abbot) at a concentration of 1.0 or 3.3 μM. The ability of Verap., which limits the action of the cell permeability glycoprotein P170, to potentiate the action of MIT or DOX in inhibiting the uptake of $^{75}$selenomethionine by the tumour cells, was measured.

| *Drug conc μg/ml* | *MIT + 1.0 μM Verap. Stage II* | *III* | *MIT + 3.3 μM Verap. II* | *III* | |
|---|---|---|---|---|---|
| 0.1 | 0/3[y] | 1/5 | 0/6 | 2/5 | [y]*No sig* |
| 1.0 | 2/3 | 3/5 | 3/6 | 2/5 | No of tumours |
| 5.0 | 0/3 | 2/5 | 0/6 | 3/5 | examined |
| | *DOX + Verap.* | | *DOX + Verap.* | | |
| 0.1 | 0/3 | 2/3 | 2/3 | 3/3 | |
| 0.5 | 2/3 | 2/3 | 3/3 | 3/3 | |
| 1.0 | 2/3 | 2/2 | 3/3 | 2/3 | |

For MIT, Verap. had a greater effect with tumours at stage III. DOX showed greater potentiation by Verap. and the level of significance increased at the 3.3 μM concentration. Prolonged culture of the tumour cells before testing led to a spontaneous decline in their sensitivity to DOX + Verap.

**Differences between monoclonal antibodies in immunohistochemical detection of P-glycoprotein in human and mouse multidrug resistant cell lines**

M.A. Barrand, T. Tsuruo & P.R. Twentyman

*MRC Clinical Oncology and Radiotherapeutics Unit, Hills Road, Cambridge CB2 2QH, UK.*

A variety of monoclonal antibodies are now available for detecting P-glycoprotein in both normal and tumour tissues and in multidrug resistant (MDR) cell lines. Although P-glycoprotein structure appears to be conserved across species, structural differences between proteins of this multigene family exist (Endicott & Ling (1989), *Ann. Rev. Biochem.*, **58**, 137). We report here differences in the ability of the monoclonal antibodies, MRK16, JSB-1 and C219, to detect P-glycoprotein in certain human and mouse MDR cell lines. An APAAP (alkaline phosphatase-antialkaline phosphatase) method was used both for histochemical localization and for ELISA assay. Both MRK16 at $30\,\mu g\,ml^{-1}$ and JSB-1 (Sanbio) at 1:20 dilution of ascites stained the outer cell membrane of over 70% of cells in the resistant human cell lines, H69-LX4, -LX20 and -VR2. Clear quantitative differences by ELISA were evident between these MDR human cells and their sensitive parent cell lines. Cells of another human cell line, L23R, which shows drug resistance unrelated to P-glycoprotein, were not stained. However these antibodies were unable to detect differences between the MDR mouse cell lines, RIF-1.0A, -1.0B, EMT6-AR1.0, -VR1.0 and their sensitive parent lines. There was very weak cytoplasmic staining in both resistant and sensitive mouse cell lines. By contrast C219 (Centocor) at $10\,\mu g\,ml^{-1}$ produced staining around the outer edge of the human MDR cells and both strong cytoplasmic and edge staining in the mouse MDR cells, together with clear quantitative differences by ELISA between resistant and sensitive cell lines. C219 recognises a cytoplasmic region highly conserved in all P-glycoproteins analysed, so representing a more universal probe for the protein. However C219 reacts also with proteins other than P-glycoprotein that may share structural homology with P-glycoprotein but that are not involved in drug efflux (Thiebaut *et al.* (1989), *J. Histochem. Cytochem.*, **37**, 159).

**Gene amplification in methotrexate resistant human choriocarcinoma cells**

G.R. Coren[1,2], A.D.B. Malcolm[1], F. Searle[2] & K.D. Bagshawe[2]

*[1]Department of Biochemistry, Charing Cross and Westminster Medical School, London W6 8RP; and [2]Department of Medical Oncology, Charing Cross Hospital, London W6 8RF, UK.*

An established cell line, JAR, derived from a human placental choriocarcinoma, was exposed continuously to methotrexate (MTX). Resistance to a predetermined $IC_{50}$ concentration was achieved after 45 passages. The cells were then exposed to stepwise increases in drug concentrations, producing four resistant sublines: BO (45 passages), B1 (+37 passages), B2 (+42) and B3 (+28), exposed respectively to $0.0075\,\mu M$, $0.05\,\mu M$, $0.1\,\mu M$, and $0.5\,\mu M$ MTX. Resistance was confirmed by calculating the $IC_{50}$ values for all 4 sublines using the MTT assay, showing relative resistances of 1.0 for the sensitive parental cell lines and 2.5, 3.6, 6.8 and 23.7 for the increasingly resistant sublines. $^3$H-MTX uptake was identical in all sublines, eliminating the possibility of transport defects.

Using a probe derived from the plasmid pSVDHFR, containing the mouse dihydrofolate reductase mini-gene, slot, Southern and Northern blot analyses were used to measure gene and RNA copy numbers in the resistant sublines. Relative gene copy numbers of 1.0, 1.12, 1.68, 1.82 and 2.68 were obtained for JAR, B0, B1, B2 and B3 cell lines respectively, with corresponding RNA copy numbers of 1.0, 1.10, 1.41, 1.77 and 2.18. These RNA values possibly represent normal transcription of amplified genes. The two major implications from this study are firstly that choriocarcinoma cells are resistant to methotrexate by gene amplification with no further control evident at the level of transcription. Secondly, these seemingly low levels of gene amplification are sufficient to induce resistance *in vitro*, and thus low level, 'undetectable', amplification may be responsible for clinical resistance *in vivo*.

**Gene amplification and expression in a series of verapamil hypersensitive CHO MDR cell lines**

M.W. Stow & J.R. Warr

*Department of Biology, University of York, Heslington, York YO1 5DD, UK.*

We have previously shown that two vincristine resistant CHO cell lines are hypersensitive to the calcium channel blocking agent, verapamil (Warr *et al.* (1988), *Cancer Res.*, **48**, 4477). In this study, we investigated the properties of two independently derived series of MDR CHO cell lines which have been selected in increasing concentrations of vincristine. The levels of cross resistance, and of verapamil hypersensitivity were determined, and amplification and expression of six gene classes (isolated from the cell line CH$^R$C5, which include the genes for P-glycoprotein (*mdr* gene family) and sorcin) were examined using Southern and slot-blotting techniques. In general, progressively increasing levels of multidrug resistance are associated with increasing verapamil hypersensitivity but with one exception, VRA33, the most MDR member. This cell line shows less verapamil hypersensitivity than an earlier member of the series with less multidrug resistance (VRA15). Both the MDR series of cell lines exhibit only small amounts of amplification ($<2.5$-fold) of the flanking sequences adjacent to the *mdr* gene family. There is no simple correlation between the level of P-glycoprotein gene amplification and changes in resistance observed in many cases. Transcriptional activation of the *mdr* gene is shown to be important and expression of the gene correlates with resistance. For example, there is an apparent decrease in amplification of the P-glycoprotein gene sequences between VRA15 and VRA33, although the expression of the gene increases. Transport studies using $^3$H vincristine show that the MDR cells have a decreased accumulation but similar rates of efflux of the drug out of the cells. The level of intracellular binding of vincristine differs quite considerably between the MDR cell lines.

**The role of hypoxic and nutrient stress in acquired drug resistance in V79 cells**

A.M. Jones, I.J. Stratford & G.E. Adams

*MRC Radiobiology Unit, Chilton, Didcot, Oxon. OX11 0RD, UK.*

The response of cells to treatment with chemotherapeutic drugs can be radically altered if cells are stressed before drug exposure. Hypoxia and nutrient deprivation are two such stresses. These stresses are of particular importance in cancer chemotherapy as tumour cells *in situ* are poorly vascularized and contain certain regions of hypoxia and nutrient deprivation. Such stresses are known to induce sets of cellular proteins including the oxygen regulated proteins (ORPs) and the glucose regulated proteins (GRPs). The induction of these stress proteins has also been seen to coincide with increased resistance to certain drugs such as adriamycin (ADR). We have carried out studies to examine the effect of stress on cellular survival and acquired resistance to ADR. Clonogenic survival studies with V79 cells grown in suspension have shown that previously hypoxic (16 hours exposure to 95% $N_2$/5% $CO_2$) or glucose deprived (4-day maintenance in glucose-free media containing serum) cells are considerably more resistant to subsequent exposure to ADR than the control cells. The presence of the calcium channel blocker, verapamil, during drug exposure does not reverse this acquired resistance. An alternative mechanism may involve the ORP/GRP system enabling cells to become resistant to ADR through membrane alterations. We examined this possibility by using an MTT assay to expose previously stressed cells to ADR either as attached cells or as cells in suspension. Exposure in suspension gave the same result as clonogenic survival studies. However, when previously hypoxic cells were attached before drug exposure the resistance was dramatically reduced. This was not seen with glucose deprived cells suggesting differences in the mode of action of hypoxic and glucose deprivation in causing drug resistance.

### Establishment of six ovarian carcinoma cell lines and estimation of drug sensitivity *in vitro*

I.F.D. Stephens, J.A. Plumb, A.B. MacLean[1] & S.B. Kaye

*CRC Department Medical Oncology, University of Glasgow and [1]Department of Gynaecology, Western Infirmary, Glasgow, UK.*

Cis-platinum (CDDP) is one of the most active agents in ovarian cancer. Although relapse with resistant tumour is common, little is known about the mechanisms of resistance to CDDP. To study possible mechanisms of drug resistance in ovarian cancer, six cell lines have been established from either biopsies or ascites fluid from patients with ovarian cancer. Five were derived from previously untreated patients and one from a patient who had received chemotherapy. They were established by disaggregation with collagenase followed by a combination of differential filtration and centrifugation through a discontinuous 'Ficoll-Paque' gradient. All grow as adherent monolayers with epithelial morphology and doubling times of about 24 hours. Four have produced tumours in nude mice and pathological examination of the tumours has confirmed their similarity to the original tumour. Sensitivity at early passage to CDDP and adriamycin (ADR) has been determined by a tetrazolium based chemosensitivity assay. For CDDP there is a 6-fold range in drug sensitivity ($ID_{50}$, 0.95–6.25 μM). The sensitivities to ADR are similar ($ID_{50}$, 2.5–5.6 μM) and are significantly higher than those obtained in this laboratory for many other tumour cell lines. Thus, these cell lines show a marked degree of resistance to ADR which reflects the poor clinical response to this drug. The range of sensitivities to CDDP is small and this may be related to the fact that most were established from pretreatment patients. Characterization of further post-treatment cell lines will clarify this point.

### Effect of verapamil on drug sensitivity and intracellular drug distribution in a drug resistant ovarian cancer cell line

W.H. Luo, J.A. Plumb & S.B Kaye

*CRC Department of Medical Oncology, University of Glasgow, Glasgow, UK.*

Verapamil (V, 6.6 μM) increases the sensitivity to both adriamycin (ADR) and daunorubicin (DNR) of an ADR-resistant ovarian cancer cell line 2780AD ($ID_{50}$; ADR 7 μM, DNR 10 μM) by 10-fold. When incubated with either ADR or DNR (1 μM) intracellular drug accumulation in 2780AD, estimated by scintillation counting, is markedly reduced when compared with the parent cell line, A2780. After 5 hours A2780 accumulates about 4 times as much drug as 2780AD for ADR and 16 times for DNR. Total drug per cell for DNR is twice that for ADR in A2780 and half that for ADR in 2780AD. For both drugs the amount per cell is doubled in the presence of V (6.6 μM). Intracellular drug distribution studies were carried out by autoradiography with $^3$H-DNR. Total drug per cell was estimated as the number of grains per cell. These studies revealed two distinct cell types within each cell line. Large cells with distinct cytoplasm contained about 3 times as much drug as the small cells with little cytoplasm. For A2780 the results showed a similar pattern to that obtained by scintillation counting. However, for 2780AD there was a sharp peak in grain count per cell during the first hour. The count then decreased to levels comparable to those seen by scintillation counting. V had no effect on the grain count for A2780. For 2780AD during the first hour the grain count increased by 5-fold in the presence of 3.3 μM V and by 10-fold with 6.6 μM V. For longer incubation times the grain count was doubled in the presence of V. For both cell lines the majority (about 75%) of the grains were in the nucleus and there was no evidence that V altered the cellular drug distribution. These results suggest that drug uptake during the first hour may be important in the mechanism of action of V.

### Ethacrynic acid does not alter glutathione-S-transferase (GST) activity or drug sensitivity of small-cell lung cancer cell lines with high levels of GST activity

J.A. Plumb, R. Milroy & S.B. Kaye

*CRC Department of Medical Oncology, University of Glasgow, Glasgow, UK.*

Elevated levels of GST have been implicated in resistance to a number of chemotherapeutic agents including adriamycin (ADR). We have established 8 small-cell lung cancer cell lines from biopsies taken from both untreated and treated patients. These lines show a 50-fold range in sensitivity to ADR ($ID_{50}$ 18–1050 nM). Drug sensitivity is not related to P-glycoprotein since none of the cell lines express detectable amounts of P-glycoprotein as determined by immunocytochemistry or Western blot analysis. There is, however, a 50-fold range in the total activity of GST (8–409 nM CDNB $min^{-1} mg^{-1}$) and a 4-fold range in the activity of glutathione peroxidase (GP, 10–40 nM cumene $H_2O_2$ $min^{-1} mg^{-1}$). The diuretic ethacrynic acid (EA) is a potent inhibitor of GST activity and it has been suggested that inhibition of GST could sensitize cells to cytotoxic drugs. When cell homogenates were incubated with EA (0.2 mM) there was a marked reduction in the measurable activity of GST. However, when whole cells were incubated with a non-toxic concentration of EA (1 μg $ml^{-1}$) for up to 24 hours the GST activity per cell was not altered. Furthermore, when the cell lines with the highest GST activity (LS263) were exposed to ADR, cis-platinum or chlorambucil in the presence of EA (1 μg $ml^{-1}$) for 24 hours the drug sensitivity was unaffected. Although the cell lines show a range of drug sensitivities and a range of both GST and GP activities there is no correlation between any of these. Thus, drug sensitivity cannot be related simply to GST activity and EA does not appear to be a useful modulator of drug sensitivity in these cell lines.

**Confocal microscopy of anthracycline subcellular localization in classic and atypical multidrug resistance (MDR)**

H.M. Coley, P. Workman, P.R. Twentyman & W.B. Amos

*MRC Clinical Oncology Unit and Laboratory of Molecular Biology, MRC Centre, Hills Road, Cambridge CB2 2QH, UK.*

The elimination of out-of-focus epifluorescence by optical sectioning using confocal microscopy allows the subcellular localization of anthracycline fluorescence to be determined with markedly improved resolution. We have compared the subcellular disposition of doxorubicin (DOX) and selected analogues in classic and atypical MDR lines with that in the respective parent lines (designated P). The classical MDR line was the EMT6/AR 1.0 mouse mammary tumour which was made approximately 40-fold resistant by exposure to DOX and exhibits hyperexpression of the P-glycoprotein membrane efflux pump. The atypical line was COR-L23/R which has a lower level of resistance induced by DOX and does not express P-glycoproteins. We have previously shown that resistance to DOX in both lines could be partially or completely circumvented by simultaneous exposure to verapamil (VRP) or the use of selected DOX analogues, e.g. 9-alkyl modified such as aclacinomycin A (ACL) or morpholinyl substituted such as morpholinyl doxorubicin (MRA). Cells were grown on glass coverslips, exposed to drugs for 2 hours and then rinsed before analysis on the MRC 500 confocal microscope (Biorad Lasersharp) with argon laser excitation at 488 nm and detection in the red. In EMT6/P, DOX ($10\,\mu g\,ml^{-1}$) staining was predominantly nuclear, including the nuclear membrane and distinct spots of intense sub-nuclear fluorescence; the cell membrane was unstained but there was some particulate staining within the cytoplasm. Staining was very weak in AR 1.0 but was restored almost to the parental level by VRP ($3.3\,\mu g\,ml^{-1}$). The subcellular pattern after DOX plus VRP in AR 1.0 was similar to DOX alone in EMT6/P but the intercellular heterogeneity was much greater. MRA ($1\,\mu g\,ml^{-1}$) staining of both EMT6 P and AR 1.0 was also similar to DOX in EMT6/P, with no effect of VRP. ACL ($1\,\mu g\,ml^{-1}$) gave no nuclear fluorescence in either line, but similar diffuse cytoplasmic staining was seen along with an intense vesicle-like pattern in both cases. Comparing DOX in L23/P and L23/R, the pattern of subcellular fluorescence was similar, being predominantly nuclear together with some diffuse cytoplasmic staining. However, the overall level was lower in L23/R and a characteristic perinuclear granular 'clustering' pattern which was not seen in the parent. This apparent localization of DOX within cytoplasmic organelles appears consistent with a role for a 'membrane-traffic' mechanism in atypical MDR.

**Relationship between intercellular communication and adriamycin resistance in non-small cell lung cancer**

C. Bradley[1], R.I. Freshney[1] & J.D. Pitts[2]

*[1]CRC Department of Medical Oncology, University of Glasgow; and [2]Beatson Institute, Glasgow, UK.*

There is evidence that the ability of cells to communicate with each other through gap junctions is associated with resistance to radiation and to the cytotoxic lymphokines, tumour necrosis factor and lymphotoxin. The explanation for this phenomenon is not clear, although it is possible that sharing of metabolic pools between coupled cells may allow them to withstand damage more effectively than isolated cells. To investigate whether this relationship between gap junctional communication and cellular resistance is generalized, applicable to more conventional cytotoxic drugs, the strength of coupling of a panel of 7 human non-small cell lung cancer cell lines was measured and compared to the chemosensitivity of these lines to adriamycin. Junctional communication was assessed by autoradiographic detection of transfer of $^3$H uridine nucleotides between cells. The strength of coupling varied widely between the cell lines and they could be separated into 3 groups: those which exhibit strong coupling, L-DAN and A549; those which exhibit weak coupling, SK-MES-1, Calu-3 and NC1-H125; and an intermediate group WIL and NCI-H23. Adriamycin chemosensitivity of each cell line was assessed by both clonogenic and MTT assays. The range of $ID_{50}$ values as measured by either assay was extremely narrow with no significant differences between the cell lines. Thus, despite the wide spectrum of junctional communication observed in these lines, this did not correlate with their adriamycin resistance.

**The application of the MTT assay to study drug resistance and drug combinations in acute myeloid leukaemia**

J.M. Sargent, C.G. Taylor, A.W. Elgie & K.K. Wilson

*Department of Haematology, Pembury Hospital, Pembury, Kent TN2 4QJ, UK.*

The identification of drug resistance in individual patients before treatment would be a therapeutic advance in the management of acute myeloid leukaemia. Our adaptation of the MTT assay is rapid and simple and we have validated its use in chemosensitivity testing of blast cells from AML (Sargent & Taylor (1989), *Br. J. Cancer*, **60**, 206). The assay results correlated with the clinical response in 21 of 23 cases. In 16 patients with *de novo* disease tested against up to 6 drugs, one showed resistance to all agents tested, 4 were resistant to doxorubicin, one to mitoxantrone, 2 to etoposide and 8 to cytosine arabinoside. Eight patients were tested against doxorubicin, mitoxantrone and daunorubicin. One patient showed resistance to doxorubicin and mitoxantrone but sensitivity to daunorubicin. Two patients, in relapse, had acquired resistance to doxorubicin whilst remaining sensitive to mitoxantrone. Sequential analyses in 2 patients over several months demonstrated increasing resistance to doxorubicin. The MTT assay is a suitable method to study the *in vitro* effect of potential resistance modifiers, for example pre-incubation with the calcium antagonist verapamil produced a 2 to 3-fold increase in patient's sensitivity to doxorubicin. As few cells are required to test 4 concentrations of each drug in the MTT assay, it was possible to construct a model to test for the effects of various combinations in 11 patients. No evidence of synergism was found. To conclude, the reliability, speed and simplicity of the MTT assay facilitates the study of drug resistance, resistance modifiers and drug combinations in AML.

**Penetration of targeting agents into multicellular spheroids derived from human neuroblastoma**

W.J. Angerson[1], R.J. Mairs[2], J.W. Babich[3] & T. Murray[4]

*[1]Royal Infirmary, Glasgow; [2]Belvidere Hospital, Glasgow; [3]The Royal Marsden Hospital, Sutton; and [4]Western Infirmary, Glasgow, UK.*

An autoradiographic technique was used to compare the ability of alternative radiotherapeutic targeting agents to penetrate multicellular tumour spheroids grown from the human neuroblastoma cell line NB1-G. Spheroids of approximately $300\,\mu m$ diameter were incubated for 10–120 minutes at 37°C in culture medium containing $0.14\,MBq\,ml^{-1}$ of $^{125}$I-labelled meta-iodobenzylguanidine (MIBG, specific activity $41.7\,MBq\,mg^{-1}$), $\beta$-nerve growth factor (NGF, 18.7 MBq $100\,\mu g^{-1}$), or the neuroectodermal-specific monoclonal antibody UJ13A ($21.7\,MBq\,100\,\mu g^{-1}$). After removal of unbound $^{125}$I by washing, calibrated autoradiograms showing the distribution and relative uptake of the tracer were obtained from $20\,\mu m$ frozen spheroid sections, and studied with an image analyser. MIBG was uniformly distributed throughout the spheroids, unless there was a necrotic core in which reduced uptake was observed. The mean (± SD) concentration of $^{125}$I-MIBG was 0.09 ± 0.01, 0.63 ± 0.07 and 1.12 ± 0.20 MBq g$^{-1}$ after 10, 60 and 120 minutes incubation, respectively. NGF was also uniformly distributed, but at a much lower concentration (3-4% of that of MIBG). UJ13A was bound predominantly in a thin layer around the periphery of spheroids, with little penetration to the interior (3-7% relative to MIBG). These results suggest that whole immunoglobulin molecules may have poor penetration into neuroblastoma micrometastases, and may therefore be an unsatisfactory targeting vehicle, particularly with short-range alpha or beta emitters.

### Comparison of two cytotoxicity assays in an *in vitro* screening system for anti-tumour agents

S.P. Fricker

*Johnson Matthey Technology Centre, Reading RG4 9NH, UK.*

A panel of human tumour derived cell lines has been set up as an *in vitro* screening system for new anti-tumour agents. The seven cell lines chosen for the screen are representative of different tissue origin (colon, rectum, breast, bladder, ovary) and clinical responsiveness. The cytotoxicity of test compounds was assessed against this panel. Two assay systems for cytotoxicity were compared. These were MTT assay based on the reduction of the tetrazolium salt by viable cells and sulforhodamine B (SRB) protein dye which measures total cell protein. Both are colorimetric assays capable of semi-automation using standard micro-titre equipment. Assay conditions were optimized for both methods. Both assays gave a linear response over the cell number range of $5 \times 10^3 - 10^5$ cells, but the SRB assay gave a higher absorbance value for a given cell number and a lower background absorbance. The SRB assay was also found to be more robust with better endpoint stability. The cytotoxicity of five test compounds, cisplatin, methotrexate, 5-fluorouracil, vincristine and mitomycin C, was compared using both assay systems. Dose-response curves were constructed over a concentration range of 0-200 μg ml$^{-1}$ and $IC_{50}$ values calculated. Both assay systems gave similar dose-response curves. $IC_{50}$ values obtained for the five compounds against the seven cell lines were comparable for both assays. Practical considerations indicate that the SRB assay may be better suited for a screening system.

### Camptothecin, a topoisomerase 1 inhibitor, can induce differentiation of K562 cells

P. McSheehy, Y. Lampasona, E. Erba & M. D'Incalci

*Istituto di Ricerche Famacologiche 'Mario Negri', Via Eritrea 62, 20157 Milan, Italy.*

Camptothecin (CPT) is an alkaloid isolated from the Chinese plant *Camptotheca accuminata* and was used in clinical trials in the 1970s. There has been renewed interest in the chemotherapeutic role of CPT following the demonstration that it is a specific and reversible inhibitor of topoisomerase 1 (Topo 1), which causes potent inhibition of RNA and DNA synthesis and impairment of cell division when the dose exceeds 100 nM. Recent work suggests Topo 1 may have a role in the control of the differentiation of cells (Gorsky *et al.* (1989), *Cancer Communications*, **1**, 83) and the differentiation of leukaemic cells is a potential new strategy in chemotherapy. The human erythroleukaemic cell line K562 is an established model for studies of differentiation *in vitro* and we have shown it is sensitive to NaCPT at doses above 100 nM. Incubation of exponentially growing K562 cells with 1 μM NaCPT for 60 minutes followed by recovery resulted in profound effects on the cell cycle. An initial increase in S-phase after 2-6 hours followed by a large block in SLG2M from 12-24 hours was always accompanied by an increase in the average cell size. Every 24 hours after recovery differentiation was assessed by the benzidine staining method which demonstrated a gradual increase in differentiation of the CPT-treated cells, reaching 40% at 120 hours while untreated cells were always around 2%. This change was accompanied by a total or sometimes near-total cessation of cell division indicating that terminal differentiation had taken place. We are currently determining if the oncogene-c *myc* is involved in this process.

### Protection of acute leukaemia cell lines from the cytotoxic action of mitozantrone by the drug-specific monoclonal antibody, NO-1

D.J. Flavell & S.U. Flavell

*Monoclonal Antibody Unit, University Department of Pathology, Southampton General Hospital, Southampton SO9 4XY, UK.*

The potent anti-cancer drug mitozantrone belongs to a class of agents which includes the anthracyclines which mediate their cytotoxic effects through DNA intercalation. We recently described the production of a highly specific monoclonal antibody (NO-1) against mitozantrone which we subsequently employed in an immunoassay for quantifying mitrozantrone levels in serum (Flavell & Flavell (1988), *J. Immunol. Methods*, **115**, 179). We demonstrate here that the anti-mitozantrone antibody NO-1 neutralizes the cytotoxicity of mitozantrone for two different acute leukaemia cell lines (ALL-1 & MOLT4) in a dose-dependent and highly specific manner. Complete protection of cell lines is achieved when antibody is present in ten times excess over drug. No protection is afforded against the related anthracycline drug daunorubicin, demonstrating the highly specific nature of such protection. The mechanism by which antibody protects cells is probably through the inability of drug-antibody complexes to gain entry to the cell, though this yet remains to be demonstrated. Such an antibody may have clinical uses in quickly reducing serum levels of mitozantrone, following high dose administration as part of the conditioning regime before bone marrow transplantation and for use in the construction of bispecific antibodies for targeting mitozantrone to tumour cell populations.

### The effect of chemotherapy on circulating multipotent progenitor cells

D.J. Kerr[1], A. Kilbey[2], M. Freshney[2], A. Sproul[2], G. Konwalinka[2] & I.B. Pragnell[2]

[1]*CRC Department Medical Oncology, Glasgow University; and* [2]*Beatson Institute for Cancer Research, Glasgow, UK.*

An *in vitro*, soft agar colony forming assay (CFU-A assay) for multipotent haematopoietic progenitor cells has been developed in our laboratory. In this study we report the use of the CFU-A assay to monitor these cells in the peripheral blood of normal volunteers (n = 6) and patients (n = 6) receiving adjuvant chemotherapy for breast cancer (cyclophosphamide 600 mg m$^{-2}$, 5-fluorouracil 600 mg m$^{-2}$, methotrexate 50 mg m$^{-2}$ every 3 weeks). Blood samples (10 ml) were collected into heparinised tubes on ice at weekly intervals. The mononuclear cells were separated by gradient centrifugation and plated in the CFU-A assay. The number of circulating CFU-A cells were consistent for the control group (week 0:140 ± 100 × 10$^9$ l$^{-1}$; week 1: 162 ± 98 × 10$^9$ l$^{-1}$; week 2: 202 ± 101 × 10$^9$ l$^{-1}$; week 3:158 ± 69 × 10$^9$ l$^{-1}$) but for the chemotherapy patients CFU-A cells fell from 170 ± 83 × 10$^9$ l$^{-1}$ (day of chemotherapy treatment) to undetectable levels by week 1 (0) control values ($204 \pm 98 \times 10^9 l^{-1}$) by week 2, and returned to control values ($243 \pm 105 \times 10^9 l^{-1}$) by week 3. Preliminary evidence from a parallel study indicates that subcutaneous administration of GM-CSF to patients receiving chemotherapy increases the number of circulating CFU-A cells further. The CFU-A assay may be of use in determining effective cell yield for autologous bone marrow transplants from peripheral blood.

### Effect of verapamil on daunorubicin accumulation in normal lymphocytes isolated from patients undergoing chemotherapy for epithelial ovarian cancer

A.T. McGown[1], D.J. Murphy[2], D. Crowther[2] & B.W. Fox[1]

*[1]Paterson Institute for Cancer Research, and [2]Department of Medical Oncology, Christie Hospital, Manchester, UK.*

Multidrug resistance has been implicated as a cause of the failure of contemporary chemotherapy in many drug regimens. Attempts to overcome this problem by use of agents which compete with the mdr-associated drug efflux process (e.g., verapamil) are currently being undertaken. This work describes the effect of verapamil on daunorubicin accumulation in normal lymphocytes isolated from patients with epithelial ovarian cancer. Intracellular daunorubicin accumulation was measured by flow cytometry in the presence and absence of verapamil (6 μM). Results were compared with data obtained from healthy volunteers. Statistical analysis of these data show that for healthy volunteers and for patients who had not received chemotherapy with adriamycin there was no increase in intracellular daunorubicin on simultaneous incubation with verapamil. However lymphocytes from patients who had undergone previous chemotherapy with adriamycin showed a highly significant ($P < 0.001$, Mann-Whitney) increase in anthracycline content following simultaneous incubation with verapamil. It is tempting to ascribe these results in terms of the development of a verapamil-sensitive drug efflux process in normal lymphocytes of patients undergoing cytotoxic chemotherapy. It is not known whether this has any relevance to the drug sensitivity of the tumour.

### Correlation between chemosensitivity to CB10-277 and 0⁶-alkyl-guanine-DNA alkyltransferase levels in human melanoma xenografts

J.M. Lunn[1], B.J. Foster[2], M. Jones[2], J. Siraky[2] & D.R. Newell[2]

*[1]Cancer Research Unit, University of Newcastle upon Tyne, Newcastle upon Tyne NE2 4HH; and [2]Clinical Pharmacology, Institute of Cancer Research, Sutton, Surrey SM2 5NG, UK.*

The resistance of tumours to methylating agents may be related to the ability of the tumour to repair lesions at the $0^6$-position of guanine in DNA. We have examined human melanomas to see if resistance to the triazene CB10-277 is related to their content of the enzyme $0^6$-alkylguanine-DNA alkyltransferase ($0^6$AT). Melanomas were grown as xenografts in nude mice. CB10-277 was administered as multiple intraperitoneal injections and sensitivity to treatment was assessed by measurement of tumour volume. Xenografts from two melanomas were resistant to CB10-277 given at $80 \text{ mg kg}^{-1}$ body weight, a dose close to the maximum tolerated dose, whereas those from a third melanoma displayed a dose-dependent sensitivity to the drug. Soluble extracts, prepared from untreated xenografts, were assayed for the presence of $0^6$AT activity using a $^3$H-methyl labelled substrate. The enzyme was barely detectable in xenografts which responded to CB10-277 treatment, whereas resistant tumours had levels of $0^6$AT in the range 150–500 fmol $\text{mg}^{-1}$ protein. Pharmacokinetics were assessed after single intraperitoneal injections of 80 mg CB10-277 $\text{kg}^{-1}$ body weight in non-tumour bearing and tumour-bearing mice. Initial levels of 1,000 μM CB10-277 decayed logarithmically over 500 minutes to 10 μM, maintaining peak concentrations of the monomethyl metabolite of 5–10 μM over a period of at least 250 minutes. No significant differences in the pharmacokinetics of the drug could be found between sensitive and resistant tumours. We conclude that resistance to CB10-277 is related to $0^6$AT activity in human melanomas.

### Combined hydralazine and heat effects on cell survival, growth delay and relative blood flow in KHT tumours *in vivo*

D.J. Honess, D. Hu & N.M. Bleehen

*MRC Clinical Oncology & Radiotherapeutics Unit, Hills Road, Cambridge CB2 2QH, UK.*

The purpose of this study was to investigate the mechanism of the enhancement of thermal damage by the vasoactive agent, hydralazine (HDZ). C3H mice with KHT tumours in the leg ($400 \text{ mm}^3$) were given HDZ ($5 \text{ mg kg}^{-1}$ i.v) 15 min before heating the tumour by a combined RF and waterbath technique which ensures a uniform tumour temperature. Temperatures studied were 43, 43.5 and 44°C. Tumour damage was assayed by regrowth delay or clonogenic cell survival, immediately or 24 hours after treatment. Relative tissue perfusion (RTP) was measured by $^{86}$Rb extraction: 5–10 μCi $^{86}$RbCl were given i.v. 60 sec before killing the mouse and taking tissue samples. RTP was calculated as % injected dose per gram of tissue. Tumour response to HDZ and/or 30 minutes at 43.5°C is shown below, together with the value expected if the effects of HDZ and heat (Hx) are additive. RTP was measured immediately, 24 h, 48 h and 72 h after treatment. Tumour data for 24 and 72 h are given below.

| | Growth delay (day) ±2se | Surviving fraction (±2se) Immediate | Surviving fraction (±2se) 24 h | RTP as % control (±2se) 24 h | RTP as % control (±2se) 72 h |
|---|---|---|---|---|---|
| Hx alone | 2.0 | 1.2 | 3.5 | 67 | 121 |
| | (1.82–2.2) | $(0.9-1.5).10^{-2}$ | $(2.1-5.0).10^{-3}$ | (51–83) | (94–148) |
| HDZ alone | 1.1 | 1.3 | 9.5 | 91 | 100 |
| | (1.0–1.2) | $(0.6-1.9).10^{-1}$ | $(4.4-14.6).10^{-2}$ | (71–111) | (60–140) |
| HDZ + Hx | 5.1 | 1.5 | 5.0 | 16 | 24 |
| | (4.8–5.4) | $(0.7-2.4).10^{-3}$ | $(1.8-8.2).10^{-4}$ | (2–30) | (16–32) |
| Exp'd | 3.1 | $1.5 \times 10^{-3}$ | $3.3 \times 10^{-4}$ | na | na |
| HDZ + Hx | (2.8–3.4) | | | | |

The growth delay for HDZ + Hx was significantly greater than the sum of the individual treatments ($P < 0.05$). This shows that hydralazine has enhanced the thermal toxicity and this finding is similar to that of Horsman *et al.* (1989, *Int. J. Hypertherm.*, **5**, 123). In apparent contrast, the cell survival data for both times of assay show that the cell killing for HDZ + Hx was the same as was predicted for an additive effect for the individual treatments. This suggests that the growth delay in these circumstances represents something other than the initial cell killing measurable by cell survival. the $^{86}$Rb extraction data showed that immediately after treatment RTP was reduced by Hx and HDZ individually and further reduced by HDZ + Hx. By 24 h the effect of HDZ was negligible, that of Hx was still evident, and that of HDZ + Hx was marked. At 72 h RTP following HDZ or Hx was normal but for HDZ + Hx was still severely depressed. This finding suggests that the greater than additive growth delay found for HDZ + Hx is due to the prolonged impairment of tumour perfusion, rather than greater than additive cell killing.

### Evaluation of bioreductive anti-cancer agents *in vivo*

S. Cole, H. Edwards, J.C.M. Bremner & I.J. Stratford

*MRC Radiobiology Unit, Chilton, Didcot, Oxon OX11 0RD, UK.*

In single modality therapy with bioreductively activated cytotoxic agents response of the small proportion of hypoxic tumour cells will be masked by surviving, resistant oxic cells. Therefore, in order to compare the efficacies of various potential bioreductive compounds, we have increased the proportion of susceptible, hypoxic cells in subcutaneous KHT murine sarcomas in syngeneic C3H/He mice. This was achieved after administration of the drugs by mechanical occlusion of the blood supply with a clamp for 90 minutes, or, alternatively, by irradiation of the tumours before drug administration. Responses were assessed by growth delay or *in vitro* clonogenic assay. The potencies of various 2-nitroimidazoles were ranked in the same order, both in clamped or irradiated tumour assays. Misonidazole and etanidazole had negligible bioreductive cytotoxic activity, RSU 1164 and RSU 1131 had moderate effects, while RSU 1069 and RB 6145 were the most effective. Some other types of agent, such as the benzotriazine-di-N-oxide, SR 4233, and the indoloquinone, E09, were more effective against irradiated than against clamped tumours. This may be a consequence of rapid metabolism and limited access of such drugs to clamped tumours. The effectiveness of RSU 1069 and SR 4233 was also compared in clamped RIF-1, SCCVII and 16/C murine tumours and in a human melanoma (HX118) and colonic carcinoma (MAWI, HT29) xenografts in nude mice. From these experiments we conclude that a variety of factors, including the enzymology of the tumour model, as well as the depth and duration of reduced oxygenation, contribute to the activity of any particular bio-reductive cytotoxin.

### Antitumour activity of thymidylate synthase (TS) inhibitory 2-desamino-2-methyl-N10-substituted-5, 8-dideazafolate analogues in a murine model

T.C. Stephens, J.A. Calvete, D. Janes, A.J. Barker, P.R. Marsham, J.M. Wardleworth, L.R. Hughes, A. Jackman[1] & A.H. Calvert[1]

*ICI Pharmaceuticals, Macclesfield, Cheshire; and [1]Institute of Cancer Research, Sutton, Surrey, UK.*

An *in vitro* clonogenic assay using a mutant of the murine L5178Y lymphoma and a 4-hour compound exposure period was developed as a prescreen to select TS inhibitors that were likely to have good *in vivo* antitumour activity. The mutant cells were chosen because they are deficient in thymidine kinase (TK-) and are therefore unable to protect themselves from TS inhibition by 'salvage' of thymidine (TdR). Antitumour activity is then achievable in rodents where plasma TdR levels are high. The short exposure period was chosen to select compounds that were rapidly transported into cells and retained, giving the prolonged inhibition of TS that is required for cytotoxicity. Polyglutamation has been shown to be a major mechanism of intracellular retention (Jackman *et al.* (1990), In: *Pteridines and Folic Acid Derivatives*, in press). The most active compounds reduced surviving fraction (SF) to about 0.001 at a dose of 1 μM and these were progressed to *in vivo* antitumour studies, again employing L5178Y TK- cells. Tumour cells were implanted i.m. into the hind limbs of DBA/2 mice and compounds were administered 3 days later. Leg diameters were monitored daily to assess tumour growth. The most active compounds produced 'cures' at doses of 10–100 mg kg$^{-1}$ and these were progressed to similar studies employing the 'salvage competent' parent L5178Y TK + cell line. In this case it was necessary to administer compounds daily for 5 days in order to observe cures. The most active analogues included those with methyl or ethyl N10 substitutents where the benzoyl ring was substituted at 2′ and 3′ with fluorine, and those where benzoyl was replaced by pyridine or thiazole.

### Initial plasma disposition studies of molecular combinations of 5-fluorouracil and nitrosoureas in mice

P.M. Loadman[1], M.C. Bibby[1], J.A. Double[1] & R.S. McElhinney[2]

*[1]Clinical Oncology Unit, University Bradford, BD7 1DP, UK; and [2]MRC Laboratories of Ireland, Trinity College, Dublin 2, Ireland.*

The B-compounds are a series of molecular combinations of chloro-ethylnitrosoureas (CNUs) and 5-FU synthesized by McElhinney *et al.* B3839, B3958, B3996 are highly active against certain MAC tumours at maximum tolerated dose (MTD) but this is dependent on the route of administration. B3839 is effective against the ascitic MAC 15A when given by the i.p. route and MAC 13 (solid) when given orally. B3958 is more active against both MAC 13 and MAC 15A when given by the i.p. route. Neither is active against the solid MAC 26 which is generally resistant to CNUs. All three tumours respond to B3996 by both routes. The aim of this study was to determine whether bioavailability could explain this spectrum of antitumour activity. Novel HPLC methods were used to measure plasma levels. B-compounds were administered to non-tumour bearing NMRI mice in a suspension of 10% DMSO/arachis oil. The results are tabulated below:

| Compound | Maximum tolerated dose (mg kg$^{-1}$) | $C_{max}$ (μg ml$^{-1}$) | | terminal $t_{1/2}$ (min) | | AUC μg h ml$^{-1}$ | | |
|---|---|---|---|---|---|---|---|---|
| | | oral | i.p. | oral | i.p. | oral | i.p. | F oral |
| B3839 | 50 | 8.0 | 29.8 | 94 | 16 | 15.2 | 15.3 | 0.99 |
| B3958 | 50 | 2.8 | 28.6 | 39 | 31 | 5.6 | 14.5 | 0.39 |
| B3996 | 100 | 12.1 | 35.4 | 107 | 33 | 10.0 | 27.1 | 0.37 |

Bioavailability (F) = AUC (p.o.)/AUC (i.p.)

These results show bioavailability is not the only criterion. Other factors including the need to attain threshold concentrations for a certain time are reflected in all the profiles measured. Further tissue distribution studies will be needed to confirm this.

### *In vivo* response of mouse adenocarcinoma of the colon (MAC) tumours to indoloquinone E09: correlation with bioreductive enzyme content

P. Workman[1], M.I. Walton[1], M.C. Bibby[2] & J.A. Double[2]

*[1]MRC Clinical Oncology Unit, Hills Road, Cambridge CB2 2QH; [2]Clinical Oncology Unit, University of Bradford, Yorkshire BD7 1DP, UK; and EORTC [1]Pharmacokinetics and Metabolism (PAM) and [2]Screening and Pharmacology (SPG) Groups.*

The selection of tumours for screening novel bioreductive drugs requires careful consideration. A parameter which is frequently unknown is the bioreductive enzyme content of the tumour and its relevance to the biochemistry of equivalent human malignancies. The MAC tumours are histologically comparable to human colon adenocarcinomas. Both MAC 16 and MAC 26 tumours are relatively slow growing. But while MAC 16 is highly necrotic and induces host cachexia, MAC 26 is well vascularized and well-differentiated. In addition, whereas both tumours are generally resistant to chemotherapy, MAC 16 is more sensitive than MAC 26 to the clinically effective bioreductive alkylating agent mitomycin C. Since these MAC tumours are in use as screens for developmental bioreductive drugs including the indoloquinone series, we determined the activities of two key reducing enzymes and sought a correlation between these levels and the response to the lead indoloquinone E09 (3-hydroxy-5-aziridinyl-1-methyl-2 (1*H*-indole-4,7-indione)-propenol. Solid tumours were grown s.c. in the flanks of NMRI mice. The activities ( ± 2SE, n = 4) of cytochrome P450 reductase were similar at 10.7 ± 1.5 and 13.5 ± 2.9 nM cytochrome *c* reduced $min^{-1}$ mg $protein^{-1}$ for MAC 16 and MAC 26 respectively. In contrast, the levels of DT-diaphorase were 15-fold higher in MAC 16 at 98.3 ± 9.8 than in MAC 26 at 6.2 ± 1.9 (same units). Both tumours exhibited a dose-dependent response to E09: single i.p. doses of 2 and 3 mg $kg^{-1}$ gave respective T/C values in standard protocols of 51% (1 + ) and 45% (2 + ) in MAC 16 and 89% (0) and 66% (1 + ) in MAC 26. A dose of 4.5 mg $kg^{-1}$ was toxic. The results suggest that the greater sensitivity to E09 of MAC 16 compared to MAC 26 may be due at least in part to the higher level of DT-diaphorase in MAC 16. This is consistent with our finding that E09 functions as a bioreductive substrate for both rat and human DT-diaphorase (Workman *et al.*, this meeting). The results may be especially relevant to the imminent clinical development of E09, as DT-diaphorase has been reported to be elevated in human cancer, including gastrointestinal malignancies.

**Immunocompetence: a necessary component for the anti-tumour activity of flavone acetic acid (FAA)**

M.C. Bibby & J.A. Double

*Clinical Oncology Unit, University Bradford, BD7 1DP, UK.*

FAA is a synthetic flavonoid with interesting preclinical activity against a broad spectrum of transplantable solid murine tumours. There is a clear vascular component to its mechanism and tumour site is important. Despite preclinical activity and the fact that clinical studies have indicated that plasma concentrations associated with murine activity have been achieved in man, no objective responses have been seen. FAA has immunomodulatory activity in mice but most published studies conclude that this is not a key factor in response.

To study the influence of immune status on FAA activity we have compared responses of two transplantable colon tumours (MAC 16, 26) in normal NMRI mice with those growing in thymectomised NMRI mice and nude mice. Tumours were implanted as trocar fragments in the flank. There was consistent growth and similar histological appearance in each host. Chemotherapy commenced when tumours could be reliably measured (day 0, tumour volume approx. 40 $mm^3$) Formulated FAA (a gift from Lipha, Lyon, France) was dissolved in physiological saline at an appropriate concentration for a desired dose to be administered in 0.1 ml $10g^{-1}$ body weight (i.p. day 0, 7). Chemosensitivity was assessed by serial caliper measurements of tumours and tumour volumes calculated by the formula $a^2 \times b/_2$ where a is the smaller and b the larger diameter of the tumour. At a dose of 200 mg $kg^{-1}$ × 2 both tumour types were highly responsive to FAA in their normal NMRI host (>50% cures) but neither tumours exhibited significant growth delay in thymectomised NMRI or nude hosts. Histological examination of treated tumours revealed significant areas of haemorrhagic necrosis in all three hosts. These data suggest a clear immunological component in the mechanism of FAA and also that human tumour xenograft studies to date need careful interpretation.

**Comparative studies of flavone acetic acid (FAA) pharmacokinetics in NMRI and nude mice**

R.M. Phillips, M.C. Bibby & J.A. Double

*Clinical Oncology Unit, University of Bradford, Bradford BD7 1DP, UK.*

FAA is an unusual compound whose mechanism of action remains unknown. Recent studies using a panel of murine colon tumours imply that the antitumour activity of FAA is dependent upon the host. In NMRI mice, MAC 15A, 16 and 26 tumours grown subcutaneously (s.c.) respond to FAA (>90% inhibition) whereas in nude mice, they do not respond. Immunological deficiencies may account for the lack of activity in nude mice although poor drug distribution may also play a major role. The aim of this study was to assess whether differences in FAA bioavailability can explain these observations.

Sequential excision of tumours and assessment of clonogenic cell kill *in vitro* at various time intervals following drug administration (200 mg $kg^{-1}$ i.p.) indicate that major cell kills occur between 4 and 6 hours after treatment in MAC 15A (s.c.) in NMRI mice, whereas limited cell kills are observed in tumours from nude mice. Peak plasma and plasma AUC (at 4 hours) values were higher in NRMI than in nude mice although no significant differences in tumour levels of FAA exist (375 and 335 $\mu g\ h^{-1}\ g^{-1}$ respectively). Two metabolites of FAA were detected in plasma and liver samples, none of which was present in significant amounts in tumour samples. In conclusion, it seems unlikely that differences in FAA or metabolite bioavailability can account for the differential activity of FAA in NMRI and nude mice. These studies suggest that the antitumour activity of FAA requires an immunocompetent host. Studies to determine the essential component are currently in progress.

**Flavone acetic acid: is tumour endothelium a primary target?**

J.C. Murray[1], G. Thurston[1], K.A. Smith[1], J. Denekemp[1] & D. Stern[2]

[1]*CRC Gray Laboratory, Mount Vernon Hospital, Northwood, UK; and* [2]*Department of Physiology and Cellular Biophysics, Columbia University, NY, USA.*

Flavone acetic acid (FAA), a novel antitumour agent, induces haemorrhagic necrosis and regression in many murine solid tumours, but has no activity towards leukaemias and ascitic tumours. In addition, FAA produces a rapid decrease in tumour blood flow. These findings have led to the suggestion that the tumour vasculature may be an important target for this drug with vascular occlusion and consequent nutrient deprivation contributing to the antitumour effect. To test this hypothesis we have studied the effects of FAA on the coagulation system and vascular permeability of tumour-bearing mice. FAA injected i.p. at doses of 300 mg $kg^{-1}$ activated the clotting cascade in control and tumour-bearing mice within 15 minutes. The effect was more pronounced in tumour-bearing animals and only in tumour-bearing animals did we detect the disappearance of circulating platelets and an increase in clotting times by 4–6 hours, suggesting a consumption coagulopathy. Studies with $^{125}$I-albumin showed a concomitant increase in vascular permeability in tumours and in the livers of tumour-bearing mice only. These results suggested that the endothelial cell might be the vascular target of FAA. Therefore further experiments with cultured human umbilical vein endothelial cells were carried out. These studies demonstrated that FAA treatment enhances endothelial procoagulant activity through the induction of tissue factor and that this activity is enhanced in the presence of TNF or a partially characterized cytokine isolated from cultured tumour cell supernatants. FAA treatment also increased the permeability of endothelial monolayers in a dose-dependent manner. The data indicate that the vascular effects of FAA may be mediated through a direct action on endothelial cells, and that the focal nature of FAA's action may be conferred by the particular susceptibility of endothelial cells within the tumour.

### The antitumour effects of 5-hydroxytryptamine (5-HT)

D.C. Jenkins, J. Wilkinson, J. Tite, J.N. Stables, P. Topley, L.S. Holmes, D.J. Linstead & E.B. Rapson

*Wellcome Research Laboratories, Beckenham, Kent BR3 3BS, UK.*

5-HT given intraperitoneally at the rate of $10 \text{ mg kg}^{-1}$ per day to C57BL/6 mice harbouring newly implanted subcutaneous MCA38 colon tumours inhibited the outgrowth of such tumours by more than 50% and was tumoristatic when given at similar levels to mice with established tumours. The compound was similarly active in NK-cell deficient C57 beige mutants but totally inactive in T-cell deficient CD1 nu/nu mice. 5-HT was also inactive against the human colon tumour xenografts HT-29 and DLD-1 in nu/nu mice and only weakly active against less immunogenic transplanted spontaneous mouse tumours such as Lewis Lung (LL/2) and melanoma B16 in C57BL/6 mice. These results suggest that 5-HT's antitumour activity cannot simply be associated with its known effects on the restriction of blood flow to tumours. Its antitumour activity would appear to share some common features with that seen after interleukin 2 (IL-2) or tumour necrosis factor alpha (TNF-a) treatment. Activity of 5-HT is T-cell dependent and seen only against immunogenic tumours. Experiments to determine which aspects of cellular antitumour immunity are enhanced by 5-HT treatment are in progress.

### Stage I epithelial ovarian cancer: why bother?

C. Finn, E.J. Buxton, K. Kelly, F.G. Lawton, R. Varma, G. Blackledge & D.M. Luesley

*University Department of Obstetrics & Gynaecology, Dudley Road Hospital, Birmingham B18 7QH, UK.*

A retrospective analysis of all Stage I EOC patients registered at the West Midlands Cancer Registry from 1st January 1980 to 31st December 1984 was undertaken. Completeness of ascertainment for all malignant tumours in the region is 98%. Two thousand three hundred and ninety-five patients with ovarian carcinoma were registered during the study period. All of the above records were examined and 457 records of Stage I ovarian carcinoma were identified. Details of all possible prognostic factors and demographic data were recorded. The median age was 58 years. The median duration of symptoms before presentation was 4 months. Adequate staging, according to FIGO recommendations, was carried out in 133 (29%) patients; 293 patients (64%) had Stage Ia disease, 37 (8%) Stage Ib and 124 (28%) Stage Ic disease. One hundred and forty-three (31%) received adjuvant chemotherapy and 59 (13%) received adjuvant radiotherapy. One hundred and sixteen patients (25%) developed recurrent disease. The overall 5-year survival was 74%. Initial logrank univariate analysis showed that age, stage, adjuvant chemotherapy, histological grade, histological type, distension at presentation and intact capsule at the time of surgery, were associated with 5-year survival. The adequacy of surgical staging was not significantly associated with survival. To determine the independent prognostic effect of these factors a stepwise logistic regression analysis was performed using 5-year survival as the determinant. Poor tumour differentiation, adjuvant chemotherapy, age and distension at presentation had independent negative effects upon 5-year survival. Although a retrospective review of this nature has limitations, and other factors not considered in this analysis may be important, it would appear that adequate surgical staging and adjuvant chemotherapy confer no benefit in terms of 5-year survival in Stage I disease.

### Analysis of prognostic variables at presentation surgery and chemotherapy on survival in 165 patients with ovarian epithelial carcinoma

J.J. Fennelly[1,2] M. Jones[1], R. Conroy[1], M. Pomeroy[1] & M. Foley[2]

*[1]St. Vincent's Hospital; and [2]National Maternity Hospital, Dublin, Ireland.*

Multivariate analysis (Log Rank Method) of 165 patients with epithelial ovarian carcinoma diagnosed between 1973 and 1986 was performed to define prognostic groups for future treatment strategies. Variables analysed included age, parity, stage, histology, grade, extent of surgery and type of chemotherapy. Ten per cent of patients were $\leqslant 39$ years, 26% were 40–49 years, 39% 50–59 years, 17% 60–69 years and 4% were 70 years or over.

Distribution by stage was 24% stage I, 18% stage II, 48% stage III and 10% stage IV. Hysterectomy and bilateral salpingoophorectomy plus omentectomy was carried out in 91 patients. Between 1973 and 1979, 64 patients received treosulfan. From 1980 to 1986, 101 patients received cisplatin ($\pm$ treosulfan). Treatment response rates were 57% clinical complete response (CR), 13% partial response and 30% nonresponders. However 25% of clinical CR had residual tumour at second look laparotomy; 27% of patients who were clear have relapsed. Overall survival at 5 years was 42% + .042SE, Stage I 63%, stage II 58%, Stage III 26%, and stage IV 0% ($P = .001$). There was a significant difference in prognosis, it being better after 1980. There was no significant difference between various surgical procedures. However, when corrected for other factors including extent of surgery there was a significant difference ($P = .041$) in favour of platinum-containing chemotherapy.

### Randomized trial of oral megestrol acetate (*Megace*) with carboplatin in ovarian carcinoma

B.M.J. Cantwell[1], M.J. Millward[1], A. Robinson[1], K.A. Godfrey[2], J. Carmichael[3] & A.L. Harris[3]

*[1]University Department of Clinical Oncology, Newcastle General Hospital, Newcastle upon Tyne NE4 6BE; [2]Department of Obstetrics & Gynaecology, Sunderland General Hospital, Sunderland SR4 7TP; and [3]ICRF Clinical Oncology Unit, Churchill Hospital, Oxford OX3 7LJ, UK.*

Progestogens have reported activity against ovarian carcinoma, stimulate the bone marrow, so potentially reducing chemotherapy-induced myelosuppression, and stimulate weight gain. In a randomized study, 49 patients received carboplatin every 28 days for a maximum of 6 cycles either alone (24 pts) or with continuous oral *Megace* $160 \text{ mg day}^{-1}$ (25 pts). Starting dose of carboplatin was $400 \text{ mg m}^{-2}$, with graded reductions or escalation to $440 \text{ mg m}^{-2}$ dependent on nadir (day 21) and pretreatment counts. With $>160$ cycles analysed there is no difference in nadir, or day 28, total white cells, neutrophils, platelets or haemoglobin between the two arms. One patient in each arm had to stop carboplatin because of prolonged myelosuppression. The mean received dose of carboplatin was $99 \text{ mg m}^{-2}$ per week in the *Megace* arm and $97 \text{ mg m}^{-2}$ per week in the no *Megace* arm ($P = 0.6$). Mean weight gain during chemotherapy was 6.1 kg in the *Megace* arm and 2.7 kg in the no *Megace* arm ($P = 0.02$). No patient who received *Megace* lost weight. No significant differences in response rate, relapse free survival or overall survival have become evident. Side-effects due to *Megace* have required dose reduction or cessation in 5/25 patients, including unacceptable weight gain in 3 pts. *Megace*, in combination with carboplatin, in the schedule used does not allow higher doses of chemotherapy to be given or provide additional antitumour activity, but resulting weight gain may be of benefit in selected patients.

### Carboplatin versus sequential ifosfamide carboplatin for patients with FIGO stage III epithelial ovarian carcinoma

T.J. Perren, S. Tan, I. Fryatt, P. Harper, M. Slevin & E. Wiltshaw

*Gynaecological Unit, Royal Marsden Hospital, Fulham Road, London SW3, UK (on behalf of the London Gynae Oncology Group).*

One hundred and thirty-five patients (pts) with FIGO stage III epithelial ovarian carcinoma were randomized to receive either carboplatin 400 mg $m^{-2}$ every 28 days for 6 courses (C – 68 pts) or ifosfamide and mesna 5 g $m^{-2}$ every 21 days for 3 courses followed by carboplatin 400 mg $m^{-2}$ every 28 days for 3 further courses (I/C – 67 pts). Median age was 58 yrs (28–77). Fifty-eight per cent were asymptomatic; 32% and 10% respectively had WHO I & II performance status. Eight per cent of pts had their disease completely resected, whereas 35%, 23% and 34% had <2, 2–5 and >5 cm residuum respectively. Eight per cent, 31% and 61% of pts had tumours of grade I, II or III respectively. After 3 cycles of treatment the response rate (CR + PR) in 54 and 48 evaluable C and I/C pts was 61% (10 CR, 23 PR) and 29% (2 CR, 12 PR) respectively ($P<0.005$) and at the end of treatment it was 66% (23 CR, 19 PR) and 66% (15 CR, 25 PR) in 64 and 61 evaluable pts respectively (ns). Median follow up was 18 months. Median actuarial time to relapse for C and I/C was 14 and 12 months respectively; median survival was 21 and 19 months respectively with 2-year survival of 43% and 40% respectively. Toxicity was equivalent in the two arms with the exception of grade III alopecia which occurred in 5% of C pts compared to 85% of I/C pts. Ifosfamide appears inferior to carboplatin in terms of response and toxicity. A significant proportion of pts resistant to ifosfamide can be salvaged by carboplatin.

### A phase II study of cyproterone acetate in advanced ovarian cancer

P. Thompson[1], R. Osborne[1], M. Slevin[1], P. Wrigley[1], E. Wiltshire[2], P. Blake[2], P. Harper[3], R. Coleman[3], D. Morris[3], C. Williams[4], J. Sweetenham[4], A. Young[5] & R. Leonard[5]

*[1]St Bartholomew's & Homerton Hospitals; [2]Royal Marsden Hospital; [3]Guy's Hospital; [4]Southampton General Hospital; and [5]Western General Infirmary, UK.*

The outlook for patients with advanced ovarian cancer relapsing after platinum-based chemotherapy is poor. In response to the finding that ovarian cancer cells exhibit androgen receptors (Hamilton *et al.* (1981) *J. Endocrinol.*, **90**, 421) and can synthesize androstenedione (Mahlck *et al.* (1986), *Gynecol. Oncol.*, **25**, 217), 61 patients with advanced ovarian cancer were treated with the androgen antagonist cyproterone acetate at a dose of 100 mg t.i.d. Fifty-five patients had had previous platinum-based chemotherapy and 6 patients considered too frail for first-line carboplatin were also included. Median age at entry was 63 and median number of previous chemotherapy regimens was 2. Of 55 evaluable patients, 4 achieved partial remission (PR), 7 static disease (SD) and 44 had progressive disease (PD). The durations of PR were 2.5, 3, 10+ and 10+ months. Two of the 4 patients achieving PR had not received platinum-based chemotherapy owing to frailty, one was in relapse 2.5 years after complete remission with cisplatin and the fourth patient had progressed on carboplatin 5 years after first responding to cisplatin. The median duration of SD was 3+ months. Overall median survival was 4 months with survival significantly dependent on response. Toxicity was minimal. Three patients experienced malaise, one rash and one dizziness. One patient developed a venous thrombosis which did not recur on cessation of treatment. Abdominal disease may have contributed to a further 3 patients experiencing diarrhoea, 2 nausea and 2 epigastric discomfort. Cyproterone acetate is active, and response to this agent is more likely in patients who are untreated or have previously responded to chemotherapy with long remission.

### A phase II trial of goserelin (*Zoladex*) in relapsed epithelial ovarian cancer

M.J. Lind[1], M.J. Millward[1], B.M.J. Cantwell[1], J. Carmichael[2] & A.L. Harris[2]

*[1]University Department of Clinical Oncology, Newcastle General Hospital, Newcastle upon Tyne NE4 6BE; and [2]ICRF Clinical Oncology Unit, Churchill Hospital, Oxford OX3 7LJ, UK.*

Twenty-six patients, median age 57.5 years (range 38–90 years), with relapsed epithelial ovarian cancer (EOC) were treated with the LHRH analogue, goserelin (*Zoladex*), given as a long-acting depot preparation. All patients had previously undergone debulking surgery and treated with platinum-containing chemotherapy. The sites of relapse were: pelvis (13), abdomen (18), ascites (8), lung (3), liver (5) and supraclavicular nodes (2). Histological grading of the original tumours was as follows: 3 well differentiated, 4 moderately differentiated, 13 poorly differentiated, 2 anaplastic, 1 mixed and 3 unknown. Each patient was treated with 3.6 mg of goserelin monthly until disease progression. A median of 2 cycles of treatment were given to each patient, range 1–11. There were two partial responses, each of six months duration and 3 patients had stable disease (of 3, 6 and 11 months duration). The median survival following commencement of LHRH agonist therapy was 26 weeks. No toxicity was seen. It would, therefore, appear goserelin (*Zoladex*) has limited activity in the treatment of relapsed, platinum-resistant, epithelial ovarian cancer.

### Glutathione S-transferase (GST) isoenzyme expression in normal and malignant human ovarian biopsies

J.A. Green, J. Harris, R. Britten, D. Meyer, P. Hendy-Ibbs & B. Ketterer

*CRC Department of Radiation Oncology, Clatterbridge L63 4JY, UK.*

Experiments in animal and human cancer cell lines have suggested that both glutathione and GST may be elevated in cells resistant to alkylating agents. Minimal data are available on levels in human malignancy. In this study, fresh biopsies of 6 normal ovaries and 13 malignant ovarian tumours were removed at laparotomy and frozen. The levels of GST isoenzymes were measured by affinity column HPLC, and total GST by CDNB substrate. GST Pi was detected in all samples and GST$\alpha$ in all except 3 malignant tissues, whereas GST $\mu$ was detected in only 2 malignant samples. There was no difference in mean levels of individual subunits of alpha and Pi or of total GST activity between normal and malignant tissue, although considerable variation was observed. The mean level of $\alpha$GST in the malignant tissue was $0.24 \pm 0.1$ $\mu$g mg$^{-1}$ protein and of $\pi$GST was $3.85 \pm 0.82$ $\mu$g mg$^{-1}$ protein. Corresponding values in normal ovary were $0.46 \pm 0.12$ $\mu$g mg$^{-1}$ protein for $\alpha$GST and $3.6 \pm 0.7$ $\mu$g mg$^{-1}$ protein for $\pi$GST. These studies are being extended to greater numbers of biopsies, and will be compared to tissue localization of $\alpha$, $\mu$ and Pi by immunocytochemistry in the same samples.

### Cerebral involvement in gestational and non-gestational trophoblastic malignancy

M.S. Dorreen[1], A.E. Champion[2], D.R. Millar[3], G.W. Pennington[3] & F.E. Neal[2]

[1]*Departments of Clinical Oncology and* [2]*Radiotherapy, Weston Park Hospital, and the* [3]*Jessop Hospital for Women, Sheffield, UK.*

Although cerebral involvement by trophoblastic malignancy is rare, choriocarcinoma has a particular predilection for pulmonary metastasis and affected patients (pts) are at risk for cerebral metastasis. In Sheffield, these 'high risk' pts have not received routine cranial prophylactic therapy. Between 1980 and 1989, 163 pts received treatment for persistent gestational trophoblastic disease (GTD). Of these, 2 pts aged 22 and 43, developed cerebral disease. Both had presented with multiple pulmonary metastases and high gonadotrophin (hCG) levels of $7 \times 10^4$ and $1 \times 10^5$ U $l^{-1}$, respectively. Both had also failed to achieve a sustained complete remission (CR) despite prolonged, intensive chemotherapy. One, aged 22, entered CR after a craniotomy and further intensive chemotherapy at the Charing Cross Hospital. The other pt died of widespread choriocarcinoma. Between 1984 and 1989, 250 pts with testicular germ cell tumours were referred to Weston Park Hospital and 3 pts aged 25, 32 and 33, developed cerebral disease. All had presented with pulmonary involvement by testicular choriocarcinomas (MTT) and hCG levels of $7 \times 10^4$ to $1 \times 10^5$ U $l^{-1}$. One pt, 25, presented *de novo* with a cerebral metastasis and entered CR after a craniotomy and intensive chemotherapy. The 2nd pt, 32, represented 6 months after a prompt CR with chemotherapy, with a cerebral lesion. He also entered CR after craniotomy and further intensive chemotherapy. The 3rd pt never attained CR and later developed multiple cerebral 2°'s, despite negative CSF hCG levels; he died from extensive MTT. No cerebral metastasis occurred either in pts with pulmonary involvement by non-trophoblastic germ cell tumours, or in cases of MTT without pulmonary involvement. It is recommended that all high-risk pts with MTT should receive CNS prophylaxis with intrathecal methotrexate, in addition to systemic chemotherapy. The recommendations for pts with GTD are less clear, since 14 other pts with similar high-risk features attained durable CRs without evidence of CNS disease, during this time of the study. It is, however, concluded that cure of cerebral disease is possible, provided this is surgically resectable and is followed up with multi-agent chemotherapy.

### Comparison of cisplatin (P) and carboplatin (C) combination chemotherapy for metastatic NSGCT

P.M. Wilkinson, G. Read, R.J. Johnson & R. Swindell

*Christie Hospital & Holt Radium Institute, Manchester M20 9BX, UK.*

Cisplatin-based combination chemotherapy remains the treatment of choice for both good and poor prognosis patients with NSGCT. Variants of PVB or other combinations to reduce toxicity are now being evaluated for patients with favourable prognostic features (*Lancet* (1985), **i**, 8). We have evaluated a 24-hour combination of cisplatin (100 mg $m^{-2}$ 24-hour infusion); etoposide (E), (150 mg $m^{-2}$ days 1 and 2) and vinblastine (V), (6 mg $m^{-2}$ days 1 and 2) (PEV) in 66 patients with proved metastatic disease. Patients with a residual abdominal mass after completion of CT received radical irradiation; 64/66 (97%) achieved a PR and 62 (95%) are currently NED, (median FU3 years). In an attempt to reduce toxicity of (P) this combination has been compared to a combination of CEV in a prospective randomized trial. To date, 27 patients have been entered and results are:

| *CT* | *N* | *CR* | *XRT to residual mass* | *Teratoma death* |
|---|---|---|---|---|
| CEV | 14 | 13 | 6 | 1 |
| PEV | 13 | 12 | 4 | 1 |

On this evidence CEV has equal efficacy to PEV. In conclusion, carboplatin can therefore replace cisplatin as a less toxic treatment option.

### Clinical pharmacokinetic and metabolism studies with the anti-cancer drug LL-D49194α1

M.A. Graham[1], A. Setanoians[1], T. Hamilton[1], S. Bell[1], W. Ten Bokkel Huinink[2], C. McDaniel[2], L. Adams[1], S.B. Kaye[1] & J. Cassidy[1]

[1]*CRC Department Medical Oncology, University of Glasgow, Alexander Stone Building, Garscube Estate, Glasgow G61 1BD, UK.; and* [2]*Netherland Cancer Institute, Amsterdam, The Netherlands.*

LL-D49194α1 (NSC 381856) is a new antibiotic isolated from the fermentation broth of *Streptomyces vinaceus drappus*. Significant antitumour activity has been demonstrated in a number of experimental tumours *in vivo* including the P388, B16, M5076 and the MX-1 tumour xenograft. As a prelude to phase I clinical studies, a highly sensitive assay has been developed for the determination of the drug in plasma using solid phase extraction and HPLC. the drug was extracted from plasma using 3cc C2-Bond Elut cartridges. Aliquots of plasma (0.5–4 ml) were extracted and plasma contaminants were eluted to waste with 1 ml methanol/water (1/19 v/v). The analyte was eluted in 0.6 ml methanol and aliquots of the methanolic extract (200 μl) were diluted with 200 μl $KH_2PO_4$ (5 mM) pH 7, to make the samples compatible with the HPLC mobile phase. Recovery over the concentration range 0.1 to 250 ng $ml^{-1}$ was $88 \pm 8\%$ ($r = 0.999$) and the limit of detection was 0.1 ng $ml^{-1}$. Patient samples were assayed by HPLC (injection volume 25–250 μl) using a 15 cm × 0.46 cm C6 (5 μM) analytical column. The drug was eluted isocratically in 50% methanol/acetonitrile (1:1 v/v), 50% $KH_2PO_4$ (5 mM, pH 7) at a flow rate of 1 ml $min^{-1}$ (retention time = 8 min) and detected using UV (270 and 405 nm) and fluorescence (excitation at 405 nm, emission at 480 nm) detection. Phase I studies were initiated at 0.25 mg $m^{-2}$ (equivalent to 1/10th LD10 in mice) administered as a single i.v. bolus injection and 2–5 ml blood samples were taken periodically over 24 hours. Pharmacokinetic studies showed that the drug was rapidly cleared from the plasma and the data could be fitted to a biexponential open model ($t1/2\alpha = 3.2 \pm 1$ min, $t1/2\beta = 18 \pm 2$ min at 1 mg $m^{-2}$). The dose has been escalated from 0.25 to 4 mg $m^{-2}$ at which significant ECG changes were encountered with one patient developing fatal myocardial necrosis. No haematological changes were observed and a minor response was noted in one patient with colorectal carcinoma at 0.25 mg $m^{-2}$. A metabolite (retention time 3.5 min) has been purified from plasma for identification by mass spectrometry. Preliminary pharmacokinetic data indicates non-linear pharmacokinetics with AUC values of 54, 247 and 172 ng $ml^{-1}$ × min at 1, 2 and 4 mg $m^{-2}$ respectively. The causes of the non-linear pharmacokinetics and cardiotoxicity are the subject of current investigations.

### Phase I trial of temozolamide (CCRG 81045; M&B 39831; NSC 362856)

D.B. Smith[1], E.S. Newlands[1], G. Blackledge[2], N.S.A. Stuart[2], J. Slack[3] & M.F.G. Stevens[3]

[1]*Charing Cross Hospital, London W6 8RF;* [2]*Queen Elizabeth Hospital, Birmingham B15 2TH; and* [3]*Aston University, Birmingham B4 7ET, UK.*

Temozolamide, the methyl analogue of mitozolamide, demonstrated broad spectrum pre-clinical activity in murine models and was of particular interest because it readily undergoes spontaneous ring opening to the active alkylating metabolite MTIC. This phase I study was initiated with an i.v. preparation formulated in DMSO. At 200 mg $m^{-2}$ comparison of the kinetics following oral and i.v. administration demonstrated good bioavailability in 4/5 patients and the study continued with the oral preparation. Dosage increments were 20% up to 520 mg $m^{-2}$ and 33% thereafter. Forty-nine patients were entered at 15 dose levels from 50–1200 mg $m^{-2}$. Grade 2–3 nausea and vomiting were seen at all dose levels. Consistent myelosuppression was first seen at 900 mg $m^{-2}$ and thrombocytopenia, although unpredictable, was considered the limiting toxicity at this dose.

| | *Total Wbc* | | *Platelets* | |
|---|---|---|---|---|
| *Dose/m$^{-2}$* | *median* | *range* | *median* | *range* |
| 900 | 2.6 | (1.3–21.2) | 71 | (39–487) |
| 920 | 5.9 | (0.5–16.9) | 241 | ( 7–530) |
| 1000 | 3.4 | (0.6–11.5) | 81 | (15–194) |

The wbc nadir occurred at week 3 (median) and the platelet nadir at week 2 (median). In this part of the trial hints of activity were seen in melanoma and in a squamous head and neck tumour. Preclinical studies suggested that the activity of temozolamide was schedule dependent and the trial is thus continuing with a 5-day schedule at a dose of 150 mg $m^{-2}$ daily. No myelosuppression has been seen at this dose and there has been further activity in melanoma. The current dose level is 180 mg $m^{-2} \times 5$. Pharmacology was performed at all dose levels showing that temozolamide exhibits linear kinetics. The $T_{\frac{1}{2}}$ was 1.86 h, apparent volume of distribution 28.24 l and clearance 170 +/− 52 ml $min^{-1}$. This work was carried out as part of the Cancer Research Campaign Phase I/II new drug development programme.

### Phase I and pharmacology studies of GR63178A, a water soluble analogue of mitoquidone

D.M. Eccles, J. Cummings, M.E. Stewart, M. Nicolson, A.J. MacLelland, M.A. Cornbleet, R.C.F. Leonard & J.R Smyth

*Imperial Cancer Research Fund Medical Oncology Unit, Western General Hospital, Edinburgh, UK.*

The pentacyclic pyrroloquinolone GR63178A is a water-soluble analogue of mitoquidone (NSC382057). The drug is active against a panel of rodent solid tumours and xenografts. Scheduling studies in animals favoured daily chronic dosing. Therapeutic doses in animal studies remarkably showed no significant toxicity. A phase I trial has been completed. Twenty-four patients (P) with refractory cancer (10 previously untreated) received GR63178A in doses ranging from 40–160 mg $m^{-2}$ daily for 5 consecutive days in each 21 day cycle; 37 courses were available for analysis. Dose limiting toxicity was pain, usually at the sites of known tumour. At 160 mg $m^{-2}$ daily 2/3 P experienced WHO grade 3 pain. Nausea and vomiting were seen in 3/4 P at 80 mg $m^{-2}$, 12/12 P at 120 mg $m^{-2}$, 1/1 P at 140 mg $m^{-2}$ day and 3/3 P at 160 mg $m^{-2}$ daily. Negligible haematological toxicity was seen; although WHO grade 1 anaemia was noted in a few patients, it was not related to dose. A reverse phase HPLC assay and solid phase sample preparation technique resolves up to 30 photo-degradation products and is sensitive for the parent drug down to 1 ng on column. Pharmacokinetic studies showed $C_{max}$ values at 80 mg $m^{-2}$ daily ranging from 4–13 μg $ml^{-1}$ and AUCS from 9–29 μg $ml^{-1}$. The MTD in humans gives pharmacological levels in excess of those observed at therapeutic doses in the rodent where the AUC was 7 μg $ml^{-1}$ and $C_{max}$ was 30 μg $ml^{-1}$. EORTC and CRC phase II studies are in progress at 120 mg $m^{-2}$ daily for 5 days every 21 days in a broad spectrum of tumours.

### Phase I clinical and pharmacokinetics study of phyllanthoside (NSC 328426; CRC85/13) using a single dose 30 minute infusion schedule

A. Lamont, P. Workman, I. Dennis & N.M. Bleehen

*MRC Clinical Oncology and Radiotherapeutics Unit, Hills Road, Cambridge CB2 2QH, UK.*

Phyllanthoside is a plant glycoside which acts as a DNA synthesis inhibitor, with some selectivity against the human tumour panel. A total of 16 patients (pts) were included: primary sites were lung (6), breast (4) and other solid tumours (6). All had prior radiotherapy, 8 prior chemotherapy. Phyllanthoside was given as an i.v. infusion lasting 30 minutes. The starting dose was 0.5 mg $m^{-2}$ (2 pts), rising through 1 mg $m^{-2}$ (2 pts), 2, 3, 4 and 5 mg $m^{-2}$ (3 pts). Dose limiting toxicity was partially reversible painful peripheral neuropathy (motor and sensory) appearing at 3 mg $m^{-2}$ (grade 1 in 1/3 pts) progressively increasing in severity to at least grade 3 in all patients at 5 mg $m^{-2}$, one of whom had grade 4 neuropathy and significant cerebellar impairment. This patient also had the highest peak plasma level of the drug. Nausea and vomiting occurred at doses above 1 mg $m^{-2}$ and was controlled by standard antiemetic therapy. There was no significant haematological, renal, hepatic, cardiac or pulmonary toxicity and no cumulative toxicity in 4 patients treated with up to 3 doses at 3 or 4-week intervals (without escalation) at dose levels 2, 4 and 5 mg $m^{-2}$. All 16 patients were assessable for tumour response: 15 progressed, 1 static. Plasma levels, measured by HPLC, rose rapidly to steady state during the infusion, declining rapidly with a $t_{\frac{1}{2}}$ of 2–3 min. Peak levels were proportional to dose (30–80 ng $ml^{-1}$ at 2 mg $m^{-2}$; 150–290 ng $ml^{-1}$ at 5 mg $m^{-2}$), with 3–5% of the dose appearing unchanged in urine. None of the metabolites seen after incubation with mouse or human plasma could be detected in our patients plasma. The results indicate that pharmacologically active concentrations of phyllanthoside can be achieved in man. Alternative infusion schedules are being investigated further.

### Modified BEP chemotherapy for metastatic testicular teratoma

R.E. Coleman, D. Whillis, G.C.W. Howard, R.E. Taylor & M.A. Cornbleet

*Department of Clinical Oncology, Western General Hospital, Edinburgh EH4 2XU, UK.*

During the past six years 39 men with metastatic testicular teratoma were treated in this Unit with a combination of bleomycin, etoposide and cisplatin (BEP). To minimize inpatient time, hospital visits and the risk of bleomycin lung toxicity, patients received four to six courses of bleomycin 30 mg and cisplatin 100 mg $m^{-2}$ on day 1 only plus etoposide 120 mg $m^{-2}$ (i.v.) on days 1–3. Only eight patients had poor prognostic features; six with bulky (> 5 cm) disease and four with βHCG > 1000 iu $l^{-1}$. Thirteen patients had lung involvement but in the other 26 disease was confined to lymph nodes. Twenty-nine patients (74%) are alive and disease-free. Five patients have died of disease, three progressed on relapse chemotherapy and are expected to die and two salvaged by surgery or chemotherapy; 24/31 (77%) good-prognosis patients and 5/8 (63%) with poor prognostic features are free of disease after a median follow-up of 31 months (range 11–76 months). However, only 6/13 (46%) patients with lung involvement (0/3 L1, 5/7 L2 and 1/3 L3) are alive and disease-free ($\chi^2 = 8.1$). Chemotherapy was well tolerated and there were no treatment-related deaths or episodes of septicaemia. Three patients developed significant high-tone deafness and tinnitus requiring substitution of carboplatin for cisplatin. Modified BEP chemotherapy is a well tolerated alternative to standard BEP chemotherapy for small volume nodal disease. However, patients with more advanced disease require more intensive therapy and are best treated either within clinical trials, or with standard well-tested regimens.

### A less toxic regimen of 5-fluorouracil and high-dose folinic acid which retains activity in advanced gastrointestinal adenocarcinomas

P.W.M. Johnson, P.I. Thompson, L. Squires, M.L. Slevin & P.F.M. Wrigley

*ICRF Department of Medical Oncology, St Bartholomew's and Homerton Hospitals, London, UK.*

The combination of high-dose folinic acid with 5-fluorouracil has improved response rates in several trials in advanced colorectal carcinoma. This is at the expense of increased toxicity: regimes using weekly bolus injections produce diarrhoea in an average of 50% of patients and occasional toxic deaths from this, whilst those using daily injections for one week in four report both diarrhoea and oral mucositis, the latter in an average of 60% of patients. Both types of regime have significant rates of myelosuppression, on average in 35%. A regime was recently reported using a different schedule of 5-fluorouracil and folinic acid, which appeared better tolerated but equally active (De Gramont *et al.* (1988), *Eur. J. Cancer Clin. Oncol.*, **24**, 1499). We report results using the same programme, but with allopurinol mouthwashes to reduce oral toxicity, in 47 patients with advanced adenocarcinomas (33 colorectal, 6 gastric, 3 pancreatic, 1 hepatocellular and 4 of unknown primary). Patients received 200 mg $m^{-2}$ folinic acid by infusion over 2 hours followed by an i.v. bolus of 5-fluorouracil 400 mg $m^{-2}$ then an infusion of 5-fluorouracil 400 mg $m^{-2}$ over 22 hours. This was repeated over the next 24 hours. The schedule was given every 2 weeks for a total of 6 to 12 courses depending upon the response. The overall response rate was 38% in 37 evaluable patients. Performance status improved with treatment in 34%. No toxicity greater than WHO Grade II occurred. Of 219 courses (median 5 per patient) 14% were complicated by transient diarrhoea and 5% by oral mucositis; 19% of patients experienced some diarrhoea and 21% stomatitis at some time, although this was always mild. Only 3% of courses were delayed by marrow toxicity, and no febrile neutropenic episodes were seen. We conclude that this regime demonstrates low toxicity but retains good activity in the palliative treatment of advanced gastrointestinal adenocarcinomas.

### Comparison of 1-hour vs 2-hour infusions of disodium pamidronate (APD) in breast cancer with bone metastases

M.J. Millward, D. Simmons, M. Procter & B.M.J. Cantwell

*University Department of Clinical Oncology, Newcastle General Hospital, Newcastle upon Tyne NE4 6BE, UK.*

APD is an effective therapy for malignant hypercalcaemia and has activity against bone metastases. Doses used have been 15–90 mg given over 2–24 hours. As part of an ongoing study of APD in breast cancer with bone metastases, 21 normocalcaemic patients (pts) received infusions of 30 mg APD in 250 ml saline over either one hour (11 pts) or two hours (10 pts) repeated every 3 weeks to a maximum of 12 doses, with continuous aminoglutethimide and hydrocortisone. Either radiological response or stable disease (SD) for $>3$ months in bone was seen in 5/11 pts who received one hour infusions and 4/10 pts with two-hour infusions. Biochemical analysis in 16 pts who received $\geqslant 4$ APD infusions shows no difference in total lymphocyte count, serum creatinine, alkaline phosphatase, AST, calcium, phosphate, osteocalcin and calcium excretion between the infusion rates. Patients with response, or SD, had a progressive fall in alkaline phosphatase, calcium excretion and osteocalcin over the duration of APD therapy with both infusion rates. When given over two-hours, side effects related to APD were transient nausea in one pt and transient bone pain immediately following infusions in one pt. No toxicity attributable to APD occurred when given over one hour. These results suggest that 30 mg APD when given over one hour has an equivalent effect as the slower infusion rate and this could allow convenient out-patient use of this drug.

### A phase II study of 4-hydroxyandrostenedione (4OHA) in post menopausal breast cancer

A. Bowman[1], M.E. Stewart[1], M. Dowsett[2], D. Gaskell[3], M.E. O'Brien[1], D.M. Eccles[1], U. Chetty[3], A. Rodger[1], R.C.F. Leonard[1] & J.F. Smyth[1]

*[1]Departments of Clinical Oncology and [3]Clinical Surgery, University of Edinburgh; and [2]Department of Biochemical Endocrinology, Royal Marsden Hospital, London, UK.*

4OHA is a potent aromatase inhibitor which suppresses plasma oestrogen levels without inhibition of adrenal steroid production. Fifty women (median age 63, range 33–80) with advanced, measurable breast cancer entered a phase II study of 4OHA. All were postmenopausal or postoophorectomy; ER was positive in 22 and unknown in 28; 11 patients had locoregional recurrence only and 39 had distant metastases (sites: contralateral breast/nodes 21, other soft tissue 20, bone 27, visceral 10). Forty-nine patients had prior tamoxifen (10 adjuvant, 39 therapeutic with a 39% response rate) and 18 also received megestrol. 4OHA was administered as a 500 mg i.m. injection every 14 days and a median of 7 injections (range 2–30) was given. Partial responses lasting 24 +, 75 + and 90 + weeks were observed in 3/45 evaluable patients (7%). Disease was stable for 24 + to 60 weeks in a further 15. Clinical toxicity was mild and there was no significant haematological or biochemical toxicity. Oestradiol levels in 29 patients were suppressed to a mean ($\pm$ SEM) of $7.2 \pm 0.6$ pM $l^{-1}$ ($31.2 \pm 2.6$% of baseline) 7 days after the first injection of 4OHA; although there was some recovery of oestradiol before the injections on days 14 and 28, this was not of sufficient magnitude to account for the low response rate observed.

### A randomized trial of adjuvant aminoglutethimide versus placebo in postmenopausal patients with node positive breast cancer

A.L. Jones[1], T.J. Powles[1], R.C. Coombes[1], I.E. Smith[1] & D. Easton[2]

*[1]Royal Marsden Hospital, London & Surrey; and [2]Institute of Cancer Research, Sutton, UK.*

Postmenopausal women with histologically confirmed node positive breast cancer were randomized to receive aminoglutethimide 250 mg q.d.s. with hydrocortisone 20 mg b.d. or placebo. Aminoglutethimide has a significant effect on disease-free survival with 55/153 AG patients and 70/150 placebo patients having developed metastases ($\chi^2$ 6.47, $P = 0.011$). There were 55/153 deaths in AG patients and 70/150 in placebo patients ($\chi^2 = 3.61$, $P = 0.57$). After allowing for T and N stage the effect on survival is more significant ($\chi^2 = 4.61$, $P = 0.032$). The estimated effect on relapse rate is a 34% reduction (95% CI 9–53%) and on survival a 29% (95% CI 1% increase – 50% decrease) reduction in the death rate. There is no interaction between treatment effect on T on N stage. For 48 ER negative patients AG did not affect disease-free or overall survival. For 128 ER positive patients the ratio of observed/expected deaths was 0.74 (NS). There was increased toxicity (lethargy, skin rash, ataxia) for AG compared with placebo and the trial was discontinued because of haematological toxicity, with agranulocytosis and thrombocytopenia in 3 AG patients. New aromatase inhibitors with lower toxicity could be explored as adjuvant therapy.

### Guy's/Manchester CMF trial in node positive breast cancer: an update at 8 years

S.M. O'Reilly, M.A. Richards, A. Howell & R.D. Rubens

*ICRF Clinical Oncology Unit, Guy's Hospital, London; and Christie Hospital & Holt Radium Institute, Manchester, UK.*

Between 1976 and 1985, 391 women (202 premenopausal, 189 postmenopausal) with node positive breast cancer were randomized after mastectomy and axillary clearance to receive CMF (n = 193) or no adjuvant therapy (n = 198). Details and early results have previously been reported (*Lancet* (1984), **ii**, 307). Median follow-up is now 8 years. For premenopausal patients, both relapse free survival (RFS) and survival (S) were significantly prolonged in patients receiving CMF (RFS, $P<0.001$; S, $P=0.003$). Significant improvements in RFS was seen in premenopausal patients with 1–3 nodes ($P=0.002$) and ≥4 nodes ($P<0.001$), with ER – ve ($P=0.002$) and ER + ve ($P=0.004$) tumours, and with PgR – ve ($P=0.01$) and PgR + ve ($P=0.001$) tumours. The benefit of CMF for patients ≤40 years was not influenced by the development of amenorrhoea. Premenopausal patients aged 41–54 benefited significantly from CMF ($P<0.001$) whereas postmenopausal CMF treated patients in the same age group did not. Indeed, no significant benefit from treatment was observed for any subgroup of postmenopausal patients. In summary, the previously reported significant improvement in RFS for premenopausal patients receiving CMF has been maintained with longer follow-up and an overall survival benefit has now emerged.

### Chemotherapy of advanced breast cancer using mitomycin X, mitoxantrone and methotrexate (3M) or vincristine, anthracycline and cyclophosphamide (VAC)

A.L. Jones, T.J. Powles, I.R. Judson, J.R. Hardy & S.E. Ashley

*Medical Breast Unit, Royal Marsden Hospital, Sutton, Surrey, UK.*

Patients with advanced breast cancer were randomized to receive mitomycin C 7 mg m$^{-2}$ q 6 wk, mitoxantrone 7 mg m$^{-2}$ q 3 wk and methotrexate 35 mg m$^{-2}$ q 6 wk (3M) or vincristine 1.4 mg m$^{-2}$, doxorubicin, or epidodoxorubicin, 40 mg m$^{-2}$ and cyclophosphamide 600 mg m$^{-2}$ q 3 wk (VAC); 85 patients received 3M and 77 VAC. The average number of courses per patient for 3M was 5.2 and for VAC 5.4. The overall response rate was 52% for both treatments with an assessable response of 60% for 3M and 54% for VAC. There were no differences in response by site. The median duration of response was 10.2 months for VAC and 8 months for 3M (NS). The median overall survival for both 3M and VAC was 9 months. There was a reduction in alopecia ($P<0.005$), neuropathy ($P<0.005$) and vomiting ($P<0.01$) for patients receiving VAC. Haematological toxicity was low and similar for each regimen. The response to cross-over chemotherapy to either 3M or VAC, after failure of primary chemotherapy, was 30%. 3M is an effective low toxicity regimen for the management of advanced breast cancer.

### The pharmacokinetics of oral lonidamine in breast and lung cancer patients

D.R. Newell[1], J. Mansi[2], J. Hardy[2], D. Button[2], K. Jenns[1], I.E. Smith[2], R. Picollo[3] & B. Catanese[3]

[1]*University of Newcastle upon Tyne, Newcastle, UK;* [2]*Royal Marsden Hospital, London, UK; and* [3]*Istituto di Ricerca F Angelini, Rome, Italy.*

Lonidamine is a novel agent which induces mitochondrial damage in tumour cells. The pharmacokinetics of lonidamine have been studied as part of the phase II evaluation of the drug for the treatment of breast and lung cancer. Twenty-four patients (26 courses) were studied who had been on oral treatment (450–600 mg day$^{-1}$) for 27–47 days. Plasma lonidamine levels were measured by reverse phase HPLC (15 × 0.46 cm Polygosil 60–5 C18 column eluted at 1 ml min$^{-1}$ with 75/24/1 methanol/water/acetic acid) with fluorescence detection (excitation 295 nm – emission 365 nm); assay linearity (1–20 μg ml$^{-1}$) r = 0.998, lonidamine recovery 101% intra assay CV 3%, inter assay CV 4%. Plasma samples were prepared for HPLC by treating with 9 vol. of methanol followed by centrifugation. Lonidamine was detected in the plasma of all patients after a 150 mg tablet; peak conc. 14 ± 7 μg ml$^{-1}$ (mean ± SD), time of peak 1.8 ± 1.2 h. During the study period of 24 hours the trough lonidamine concentration was 5 ± 3 μg ml$^{-1}$ and for 20 courses the lonidamine half-life was 4.2 ± 2.3 hours. There was no marked relationship between lonidamine pharmacokinetics and patient set, age, performance status, tumour type or metastasis, renal function, hepatic function or plasma protein level. Neither was there any relationship between pharmacokinetics and tolerance to lonidamine. In addition to lonidamine, fluorescent peaks were detected in plasma which suggest that lonidamine undergoes metabolism *in vivo*. One component was degraded by glucuronidase and hence may be a glucuronide conjugate. These studies indicate that lonidamine undergoes reproducible absorption in breast and lung cancer patients.

### Trends in the outcome of lung cancer management: a study of 9090 cases in the Mersey Region

S.W. Watkin, J.A. Green & G.H. Hayhurst

*CRC Department of Radiation Oncology, Clatterbridge Hospital, Merseyside L63 4JY, UK.*

A detailed Registry analysis of 9,090 cases of histologically confirmed lung tumours in Mersey Region was carried out from 1974–1986. The total number of cases per year remained constant, but the M:F ratio fell from 3.6:1 to 2.0:1. Age at diagnosis and histological type were the most important prognostic factors, with the five-year survival of adenocarcinoma, squamous carcinoma, undifferentitaed carcinoma and small cell carcinoma after treatment being 22.5%, 18.5%, 10% and 3.5% respectively. The survival of patients who underwent surgical resection in the 3 periods 1974–77, 1978–81 and 1982–86 showed a continuous improvement in median survival from 13 to 30 months ($P<0.001$). An analysis of 741 cases of small cell carcinoma given chemotherapy over the same periods showed a highly significant improvement in survival ($P<0.001$) with an increase in two-year survival from 2.5% to 7.5%; this was shown to be closely associated with the increasing use of intravenous combination chemotherapy. Overall survival curves of all treated cases showed a significant improvement in median survival from 8 to 10 months and five year survival from 12.5% to 17.5% ($P<0.001$). While selection contributes to the distribution of patients within the different categories, the data support the argument that there is a survival benefit associated with appropriate treatment for lung cancer.

### Phase II study of VP16 (V), ifosphamide (I) and cisplatin (P) [VIP] in small cell and non-small cell lung cancer

D.N. Carney & L. Grogan

*Mater Hospital, Dublin 7, Ireland.*

We have carried out a phase II study of VIP in 20 previously untreated patients (pts) with lung cancer. Treatment consisted of V: 100 mg $m^{-2}$, I: 1.0 g $m^{-2}$ and P: 20 mg $m^{-2}$ days 1–5 every 3 weeks for a total of 4 cycles. MESNA was used as a uroepithelial protector. No pts received radiation therapy. Pts were evaluated for responses after 2 and 4 cycles of treatment and all CRs were pathologically confirmed. Among 14 pts with small cell lung cancer (SCLC): (males 10; females 4; median age 62 years [52–70]) 9 pts had ED and 5 LD. The median ECOG performance status was 2 (1–3). The overall response rate was 91% (CR 9 pts; PR 4). The median survival of these pts was 12 months. Among 6 pts (all male) with locally advanced (4 pts) and metastatic (2) NSCLC, 4 pts (2 CR; 2 PR) responded to therapy with a median survival of 8 months. Toxicity of VIP was significant: leucopenia (WHO 3–4) occurred in 50% and thrombocytopenia (WHO 3–4) in 30% of pts; 8 pts had one, or more, episodes of septicaemia. Neurotoxicity (paraesthesiae) was observed in 30% but no significant nephrotoxicity or ototoxicity was observed. Although VIP is a highly effective regimen in the treatment of SCLC, its significant toxicity and need for in-patient hospital administration, coupled with treatment results similar to other regimens, suggests it has a limited role in the treatment of this disease. Its role in NSCLC needs further evaluation.

### Correction of coagulation abnormalities in small cell lung cancer using the fibrinolytic agent stanozolol

S. Bicknell[1], A. Rumley[2], J. Douglas[2], R. Milroy[3], G. Lowe[2] & S. Banham[3]

*[1]CRC Department of Medical Oncology, Glasgow University; [2]University Department of Medicine and [3]Department of Respiratory Medicine, Royal Infirmary, Glasgow, UK.*

Disordered coagulation parameters have been documented in several tumours including small cell lung cancer (SCLC). There is increasing interest in the manipulation of coagulation as a possible therapeutic manoeuvre in SCLC. We have previously demonstrated an association between increased coagulation tendency and lack of response to chemotherapy in SCLC (Milroy *et al.* (1988), *Thorax*, **43**, 978). We have now studied the haemostatic effects of the fibrinolytic agent stanozolol in 18 patients with untreated SCLC. Pretreatment fibrinogen levels were elevated with a mean of 5.5 g $l^{-1}$, range 2.32–9.82 g $l^{-1}$ (normal range 2–4 g $l^{-1}$). After 7 days treatment with stanozolol 5 mg b.d. orally, fibrinogen levels fell to a mean of 3.93 g $l^{-1}$, $P<0.001$ as measured by paired t test. The mean levels of plasminogen activator inhibitor also fell from 25.4 U $l^{-1}$ to 19.1 U $l^{-1}$ (normal range $<20$ U $l^{-1}$), $P<0.005$. Although the clinical significance of these changes are uncertain we feel stanozolol merits further study as a potential biological modulator.

### The effect of tamoxifen on serum lipids and lipoproteins in women

A.L. Jones[1], T.J. Powles[1], C.R. Tillyer[2], J. Treleaven[3] & S.E. Ashley[4]

*Departments of [1]Medicine, [2]Biochemistry, [3]Haematology and [4]Computing, Royal Marsden Hospital, Sutton and London, UK.*

Tamoxifen has been given to well women at high risk of developing breast cancer in a randomized double-blind, placebo controlled trial. Serum lipids and lipoproteins have been studied in 78 women before and at 2–12 months after, treatment. In postmenopausal women on tamoxifen there was a significant fall in total cholesterol (0.85 mM $l^{-1}$ $P<0.005$), fasting low density lipoprotein cholesterol (0.91 mM $l^{-1}$, $P<0.01$) and apolipoprotein B (15.9 mg $dl^{-1}$, $P<0.005$). The fall in cholesterol in women with elevated pretreatment levels ($>6.5$ mM $l^{-1}$) was even greater (1.91 mM $l^{-1}$, $P<0.005$). There was no significant change in fasting high density lipoprotein cholesterol, apolipoprotein A or fasting triglycerides. Premenopausal women on tamoxifen had significant falls in total cholesterol (0.44 mM $l^{-1}$, $P<0.05$) and fasting low density lipoprotein cholesterol (0.71 mM $l^{-1}$, $P<0.01$) but no significant change in other parameters. There was no adverse effect on fibrinogen/antithrombin 3 ratio. These results indicate oestrogenic activity of tamoxifen in post menopausal women which may confer significant benefit on coronary heart and cerebrovascular disease.

### New anti-emetic BRL43694 for the treatment of cytotoxic induced emesis

O.C. Cockerill, C.J. Gallagher & R.T.D. Oliver

*Department of Medical Oncology, The London Hospital, London E1, UK.*

BRL 43694 (BRL) is a new anti-emetic with specific antagonism of 5HT3 receptors in the brain and gut wall. Nausea and vomiting (n & v) are serious chemotherapy side effects against which BRL has been active. Twenty-six patients (pts) were treated for a total of 45 cycles of chemotherapy as part of a multicentre trial; 19 pts received cisplatin (P), 10 alone and 9 with other agents (adriamycin, methotrexate, vincristine, vinblastine). The dose of P was 20–80 mg $m^{-2}$ depending on renal function; 12/19 pts received BRL during subsequent cycles. Five pts received carboplatin alone, and 2 had other combinations. The BRL was given as an i.v. infusion half an hour before the start of chemotherapy, as a single dose of 40 $\mu$g $m^{-2}$. Tumour types were: ovary (11), testis (5), bladder (14), others (3). Efficacy was assessed by pts and ward staff as relief from n & v on a scale of 0–3 and side effects were noted. Overall the BRL was completely effective in 18/26 (69%) pts and 30/45 (66.6%) cycles with no nausea or vomiting. Five/26 pts vomited (2 pts 2–4 v, 3 pts $>$.4 v) and 3/26 had mild nausea only. Rescue anti-emetics were required in 4/26 (15%) of pts. Complete control of n & v was achieved in 12/19 (63%) of pts on P and 5/5 pts on carboplatin. Headache occurred in 6 pts (23%) and constipation in 5 pts (19%), but did not interfere with treatment. We conclude that BRL is safe and effective against a wide range of cytotoxic-induced emesis and should be formally tested against standard antiemetic regimens.

### Comparison of the antiemetic ondansetron with dexamethasone plus domperidone in the prophylaxis of chemotherapy induced emesis refractory to dexamethasone

R.C. Stein[1], C. Evans[1], J. Davenport[1], L. Carruthers[2] & R.C. Coombes[1]

*[1]Clinical Oncology Unit, St George's Hospital Medical School, London SW17 0RE; and [2]Glaxo Group Research Ltd, Ware, Herts., UK.*

Selective $5HT_3$ antagonists have been shown to be effective in the prophylaxis of chemotherapy induced emesis in phase II trials. We report a double-blind study of 26 patients receiving a variety of non-cisplatin chemotherapy regimens with nausea and vomiting refractory to high dose dexamethasone who were randomized to receive either ondansetron or dexamethasone and domperidone (Dex + Dom). Thirteen patients received ondansetron 8 mg (4 mg i.v. + 4 mg p.o.) with chemotherapy followed by 4 mg p.o. q.i.d. and placebo capsules 1 q.i.d. for 5 days. Thirteen patients received dexamethasone 8 mg (4 mg i.v. + 4 mg p.o.) and domperidone 20 mg p.o. with chemotherapy followed by dexamethasone p.o. 4 mg q.i.d. 2 days, 2 mg q.i.d. 2 days, 1 mg q.i.d. 1 day together with domperidone 20 mg p.o. q.i.d. for each of the 5 days. The number of vomits and retches and nausea (on a 4-point scale) were recorded daily by patients on diary cards. Treatment was considered successful if there were no vomits or retches and nausea was absent or mild. Success rates for day 1 (acute phase) and days 1–5 (overall study period) were compared using Mantel-Haenzsel $\chi^2$ test.

| | *Number (%) successful* | | *P* |
|---|---|---|---|
| | *Ondansetron* | *Dex + Dom* | |
| Day 1 | 10 (77) | 2 (15) | 0.002 |
| Days 1–5 | 8 (62) | 1 ( 8) | 0.005 |

Our results show that ondansetron is considerably more effective than the combination of dexamethasone and domperidone in the prophylaxis of chemotherapy induced emesis refractory to dexamethasone. The addition of domperidone to dexamethasone confers only marginal additional benefit to dexamethasone alone.

### Ondansetron (GR38032F): effective anti-emetic prophylaxis for patients receiving carboplatin chemotherapy

D.B. Smith[1], G.J.S. Rustin[1,2], N. Howell[2] & B.A. McQuade[3]

*[1]Charing Cross Hospital, London; [2]Mount Vernon Hospital, Middlesex; and [3]Glaxo Group Research Ltd. (for the North West Thames Ovary Group), UK.*

Carboplatin is significantly less emetogenic than the parent drug cisplatin. However, there remain patients receiving carboplatin whose symptoms are inadequately controlled using standard anti-emetics. This study investigated the activity of the 5HT3 antagonist ondansetron in patients with ovarian carcinoma who were treated with a one-hour infusion of carboplatin at a dose of 400 mg $m^{-2}$. An oral loading dose of ondansetron (8 mg) was given 15 minutes before the carboplatin and continued at 8 mg orally t.d.s. for 5 days. Fifteen patients who had failed a combination of dexamethasone (8 mg b.d. × 4) + metoclopramide (20 mg q.d.s.) and 16 naive patients were studied. Emesis was graded as C: no episodes; M: 1–2 episodes; m: 3–5 episodes; F: >5 episodes and nausea as none (N), mild (m), moderate (M), severe (S). Symptoms were recorded in the 24 hours following therapy and for days 2–5.

*Results:*

| *Refractory* | | | | | | | | *Naive* | | | | | | | |
|---|---|---|---|---|---|---|---|---|---|---|---|---|---|---|---|
| *1st 24 hours* | | | | *days 2–5* | | | | *1st 24 hours* | | | | *days 2–5* | | | |
| C | 11 | N | 7 | C | 9 | N | 3 | C | 14 | N | 10 | C | 10 | N | 6 |
| M | 2 | m | 5 | M | 3 | m | 4 | M | 1 | m | 5 | M | 3 | m | 4 |
| m | 2 | M | 2 | m | 2 | M | 5 | m | 0 | M | 0 | m | 2 | M | 5 |
| F | 0 | S | 1 | F | 1 | S | 2 | F | 1 | S | 1 | F | 1 | S | 1 |

Thus, 11/15 (73%) refractory patients and 14/16 (87%) naive patients had no emetic episodes in the 24 hours following JM8 and overall 19/31 (61%) no emesis during days 2–5. During days 2–5, 17 experienced no, or mild, nausea while 10 (one not recorded) had moderate, and 3 severe, nausea. Single agent ondansetron is effective anti-emetic prophylaxis for both refractory and naive patients receiving carboplatin and will allow a higher proportion to be treated as outpatients.

### Hypothalamic pituitary responses in induced emesis: relationship to chemically - induced emesis

D.J. Dunlop[1], K. Harvey[2], C.E. Gray[2], M. Soukop[1], & G.H. Beastall[2]

*[1]Department of Medical Oncology and [2]Institute of Biochemistry, Glasgow Royal Infirmary, Glasgow G4 0SF, UK.*

Cytotoxic induced emesis is a major dose limiting factor in the administration of cytotoxic chemotherapy. It has been reported that endogenous opioid peptides may be markers of cytotoxic related emesis but the relationship of these peptides to other neurotransmitters and hormonal systems remains unclear (Harris (1982), *Lancet*, **2**, 714; Forrest *et al.* (1987), Abstract BACR meeting, Newcastle). The aim of this study was to measure beta-endorphin, ACTH, and cortisol in voluntary subjects given syrup of ipecacuanha to assess whether previously documented peaks of beta-endorphin seen in patients experiencing cytotoxic related nausea and vomiting is merely representative of a stress response. A clear pattern of responses was seen in subjects given ipecacuanha. Most vomited within 30 minutes; beta-endorphin begins to rise with the onset of nausea and peaks during actual vomiting (mean rise 367% above baseline), accompanied by a serum ACTH peak (mean rise 444% above baseline), and a more delayed and prolonged elevation of serum cortisol (mean rise 245% above baseline). These responses could be completely ablated by 2 mg dexamethasone given the night before. However, all subjects tested so far have still vomited. These experiments would suggest that nausea and vomiting are potent triggers of the processing of propiomelanocortin in the anterior pituitary and rapid release of beta-endorphin and ACTH into the bloodstream as part of a stress response. We would also suggest that suppression of the hypothalamic/pituitary axis has little to do with dexamethasone's function as an antiemetic.

### Treatment for Hodgkin's disease relapsing after chemotherapy; a report of the first 18 pts receiving VAPEC-B, a weekly schedule comprising 5 cytotoxic drugs and prednisolone with prophylactic co-trimoxazole and ketoconazole

J.A. Radford & D. Crowther

*CRC Department of Medical Oncology, Christie Hospital, Manchester, UK.*

A total of 18 pts (12 male, 6 female; median age 29 yrs) have received VAPEC-B chemotherapy for relapsed HD. Six pts were treated in first relapse, seven pts in second relapse and five pts in third or subsequent relapse. All patients had received at least one previous adriamycin-containing regimen and 17 of 18 pts had also received radiotherapy. Initial stage was I, 1 pt; II, 5 pts; III, 3 pts; IV, 9 pts and histology was nodular sclerosing 10 pts; mixed cellularity 2 pts, lymphocyte predominant 3 pts and HD unclassified 3 pts. A median of 8 cycles of VAPEC-B were administered (range 6–11) and 8/18 pts (44%) achieved CR or equivocal CR (minimal residual abnormality on CXR or CT scan but no palpable disease and BM/biochemistry normal). A further 5 pts achieved PR, 3 pts had stable disease, 1 pt progressed and 1 pt with extensive lung disease died of sepsis after week 3. The CR/equivocal CR rate was the same for pts treated in 2nd or subsequent relapse as for those in 1st relapse (both 4 of 9, 44%) but overall response (CR & PR) was greater in this latter group (8 of 9, 88% against 5 of 9, 55%). After VAPEC-B, 12 pts proceeded to high dose cyclo and BCNU followed by ABMR. For reasons of massive disease, advanced age, psychological unsuitability or no auto/allo source of BM, 5 pts did not and of these 2 continue in CR at 12 and 15 mths (1 had nodal disease in 2nd relapse, 1 had nodal disease and heavy BM involvement in 3rd relapse). Apart from 1 septic death after week 3, toxicity has been easily manageable and subjectively the regimen is well tolerated. We conclude that weekly cytotoxic therapy is effective in relapsed HD and should be considered as a possible alternative to standard three or four weekly treatment in this disease.

VAPEC-B is Adr. 35 mg $m^{-2}$ weeks 1, 3, 5, 7, 9, 11; Cyclo. 350 mg $m^{-2}$ i.v. weeks 1, 5, 9; Etop. 100 mg $m^{-2}$ p.o. daily for 5 days weeks 3, 7, 11; Vinc. 1.4 mg $m^{-2}$ weeks 2, 4, 6, 8, 10; Bleo. 10 mg $m^{-2}$ i.v. weeks 2, 6, 10 plus prednisolone 50 mg p.o. daily weeks 1–5, 25 mg daily 6–11 then tailed to zero, and prophylactic co-trimoxazole 2 tabs 12 h and ketoconazole 200 mg 12 $h^{-1}$, both for 12 weeks.

### A randomized study of adjuvant MVPP chemotherapy after mantle radiotherapy in P.S. IA-IIB Hodgkin's disease. 10-year follow up

H. Anderson, D. Crowther, D. Deakin & W.D.J. Ryder

*Christie Hospital, Manchester, UK.*

From Oct. 1974–Aug. 1981, 115 patients with untreated supradiaphragmatic pathologically staged IA-IIB Hodgkin's disease (HD) were entered into a randomized study of adjuvant MVPP (mustine, vinblastine, prednisolone, procarbazine) or follow up alone after mantle radiotherapy. The median duration of follow up was 131 months; 56 patients were treated with radiotherapy (RT) alone and 59 received RT plus 6 cycles of adjuvant MVPP. Patient characteristics between the two groups (age, sex, stage, Karnofsky performance, bulk disease, histology and number of sites involved) were not significantly different. One hundred and thirteen patients achieved a complete remission (CR) with RT alone. The 10-year survival corrected for intercurrent death was 92%. There were 9 (8%) deaths from HD (5 received RT alone). Eight patients (7%) developed a second malignancy of whom 6 have died. There have been 4 other intercurrent deaths. The 10-year relapse-free survival (RFS) was 80% overall, 90% in the adjuvant MVPP group and 66% in the RT group ($P = 0.0004$). There were 25 relapses, 19 received RT alone and 6 received adjuvant MVPP. Of the relapsed patients one progressed and died on adjuvant MVPP, one relapsed and died of infection before therapy could be given. The other 23 received chemotherapy ± radiotherapy, 19 (83%) obtained a CR and 4 progressed and died of disease. Of the CR patients, 3 died in second relapse, 3 had intercurrent deaths and 13 (68%) continue in CR. Univariate analysis showed the following factors influenced survival – pruritus $P = 0.0014$, night sweats $P = 0.0016$, B symptoms $P = 0.0023$, bulk $P = 0.0041$, monocyte count $> 0.5 \times 10^9 l^{-1}$ $P = 0.0059$, stage IIB $P = 0.0157$, histology mixed cellularity $P = 0.0227$, lymphocyte count $< 1.7 \times 10^9 l^{-1}$ $P = 0.0385$. Univariate analysis showed that treatment – RT only $P = 0.0004$, lymphocyte count $< 1.7 \times 10^9 l^{-1}$ $P = 0.0011$ and B symptoms $P = 0.0269$ predicted relapse. Multivariate analysis was only possible for relapse as there were too few events to anlayse for HD deaths. Analysis of 24 variables showed only 3 variables predicted relapse, treatment-RT alone $P = 0.0001$, lymphocyte count $< 1.7 \times 10^9 l^{-1}$ $P = 0.0005$, and haemoglobin $< 14 g l^{-1}$ $P = 0.0191$. Adjuvant MVPP has shown a significant improvement in RFS but not overall survival.

### Residual abdominal masses following intensive weekly chemotherapy for intermediate and high grade non-Hodgkin's lymphoma (NHL)

J.W. Sweetenham, G.M. Mead & J.M.A. Whitehouse

*CRC Wessex Medical Oncology Unit, Southampton General Hospital, Tremona Road, Southampton SO9 4XY, UK.*

In patients (pts) with intermediate and high grade NHL presenting with intra-abdominal disease, a residual abdominal mass is frequently detected radiographically at the end of chemotherapy in the absence of peripheral lymphadenopathy. A previous study from the NCI has shown that following ProMACE-MOPP chemotherapy, resected residual masses rarely contain viable lymphoma (*J. Clin. Onc.* (1988), **6**, 1832). We have treated 60 pts with advanced intermediate and high grade NHL with an intensive weekly chemotherapy regimen as follows: etoposide 150 mg $m^{-2}$ i.v. day 1, doxorubicin 35 mg $m^{-2}$ i.v. day 1, cyclophosphamide 300 mg $m^{-2}$ i.v. day 1, methotrexate 100 mg $m^{-2}$ i.v. day 8 with leucovorin rescue, vincristine 1.4 mg $m^{-2}$ i.v. day 8, bleomycin 10 mg $m^{-2}$ i.v. day 8. Treatment interval 14 days for 6 cycles with continuous oral prednisolone and cotrimoxazole. Forty pts had intra-abdominal disease at presentation; 18 pts had residual abdominal masses detected by CT 4 weeks after completing chemotherapy. Median follow up for this group = 19 months (range 6 to 41). Histology (WF): DLC – 13, DM – 4, DSC – 1. Follow up CT scans were performed at 6-month intervals until complete resolution or disease progression, and no further therapy was given.

Results: The presence of bulky (> 10 cm) abdominal disease at presentation did not correlate with the presence of a residual mass. The outcome for residual masses was complete resolution in 6, taking > 1 yr in 2 cases; stable abnormality – 2 (both alive with no disease progression at 24 and 26 months); disease progression i) at site of mass only – 2, ii) at site of mass and distant site – 5, iii) at distant site only – 3. Current status for the 18 pts; alive without disease – 11 (including 2 pts treated with salvage chemotherapy), alive with disease – 3, dead with disease – 4. For this small group, no trend was observed for a higher relapse/progression rate in pts with > 5 cm compared with < 5 cm residual masses. In conclusion, the presence of residual abdominal masses on CT does not always imply viable lymphoma; the incidence of active disease in such pts after this intensive weekly chemotherapy regimen is higher than that reported for ProMACE-MOPP.

### Preliminary data from the West of Scotland Lymphoma Group randomized trial of MOPP versus MOP/EVAP in advanced or bulky Hodgkin's disease

M. Harding, N. Lucie, N. Reed, R. McNeil, E. Fitzsimmons, D. Ellis, L. Cram, S. Kaye & M. Soukop

*Departments of Medical Oncology and Haematology, West of Scotland, UK.*

In an attempt to improve the treatment of Hodgkin's disease, we have initiated a randomized trial comparing a hybrid regime to standard MOPP chemotherapy. Since 1986, 93 patients have been entered, 83 of whom are evaluable. Their median age at the time of treatment was 34 years (range 14–77); 7 had received prior radical irradiation 1–8 years previously, the remainder were not pretreated. Tumour stages were 8 I, 33 II, 22 III and 19 IV; 57 (69%) had B symptoms, 44 (53%) bulk disease and 27 (33%) extranodal lymphoma. All parameters apart from Stage IV disease were equally distributed between the treatment groups. Chemotherapy comprised mustine 6 mg $m^{-2}$, vincristine 1.4 mg $m^{-2}$ (maximum 2 mg) i.v. on days 1 and 8, with procarbazine 100 mg $m^{-2}$ and prednisolone 25 mg $m^{-2}$ orally, days 1–14 (MOPP), or identical agents days 1–7, with adriamycin 25 mg $m^{-2}$, vinblastine 6 mg $m^{-2}$, etoposide 75 mg $m^{-2}$ i.v. on day 8, oral etoposide 150 mg $m^{-2}$ on days 9 and 10, and prednisolone days 8–14 (MOP/EVAP). The median number of courses delivered at 28-day intervals was 6 in each arm (range 1–8). Eighteen patients subsequently received irradiation for CT scan PR or to sites of prior bulk disease. With a median follow up of 16 months, data are as shown:

| | *MOPP* | *MOP/EVAP* |
|---|---|---|
| Number treated | 40 | 43 |
| Stage IV disease | 14 | 5 |
| Infected episodes | 7 | 22 |
| Toxic deaths | 2 | 2 |
| CR/No. evaluable | 16/26 | 28/36 |
| PR/No. evaluable | 9/26 | 8/36 |
| Relapses | 6 | 2 |

Differences are not significant and accrual continues.

**Demonstration of DNA interstrand crosslinks in peripheral blood mononuclear cells in patients undergoing chemotherapy**

G.N. Rudd, J.A. Hartley, C.M. Chresta & R.L. Souhami

*Department of Oncology, University College & Middlesex School of Medicine, 91 Riding House Street, London W1P 8BT, UK.*

For many cytotoxic agents, such as alkylating agents, DNA interstrand cross-links (ISCs) are thought to be the lethal cellular lesion. The formation and repair of these lesions can be studied *in vitro* using the technique of alkaline elution after radioactively labelling DNA. A recent technical advance has been the use of the fluorescent DNA binding dye, Hoechst 33258, which allows measurement of ISCs without prior labelling of cells. We have adapted this technique to determine if ISCs can be demonstrated in peripheral blood mononuclear cells (PBMs) in patients treated with alkylating agents and platinum analogues. *In vitro* experiments showed that ISCs could be detected in clinically relevant dose ranges and with a sensitivity comparable to *in vitro* labelling with radioactivity. After cytotoxic chemotherapy with single agents ISCs were demonstrated in PBMs of patients with solid tumours. ISCs were detectable following treatment with cisplatin and carboplatin. An unexpected finding was the demonstration of alkali labile sites (but no ISCs) following ifosfamide administration. This technique promises to yield valuable information on the onset and repair of DNA damage in PBM (and leukaemic cells) in patients undergoing chemotherapy.

**Pharmacokinetics of high dose melphalan in patients with a range of renal function**

T.R. McCappin, D.R. Newell, C. Viner, I. Judson, M. Gore, P. Bedford[1] & T.J. McElwain

*Institute of Cancer Research, Royal Marsden Hospital, Sutton, Surrey and [1]Wellcome Foundation Ltd., Beckenham, Kent, UK.*

It is known that impaired renal function is associated with increased toxicity of high dose melphalan and an arbitary dose reduction of 50% is used routinely for patients with a GFR > 40 ml $min^{-1}$. A study was therefore initiated to investigate the effects of renal function on high-dose melphalan pharmacokinetics. To date, 23 patients have been treated with high-dose melphalan given as a single i.v. bolus injection over the range 70–200 mg $m^{-2}$ using two different formulations. Renal function was determined by measuring $^{51}Cr$-EDTA clearance and a wide range of values was observed from 39–135 ml $min^{-1}$. Following drug administration blood samples were collected at 5, 15, 30, 45, 60, 120, 180, 240, 360, 480 and 1440 minutes after injection. Melphalan was extracted from plasma to methanol by a two-step process and measured by HPLC with u.v. detection at 254 nm. Plasma melphalan pharmacokinetics were analysed according to a two compartment model. A series of 10 parameters was produced and compared to EDTA clearance to determine any relationship with renal function. A weak correlation was observed between renal function and both melphalan clearance and AUC. Further data from patients with poor renal function are required before more precise recommendations can be made regarding appropriate dose reductions. No difference was observed between the two formulations for any pharmacokinetic parameter.

| *Parameter* | *Median* | *Correlation coeff. v EDTA clearance* |
|---|---|---|
| Cl (l $min^{-1}$ $m^{-2}$) | 0.3 | 0.6 |
| AUC (μg $ml^{-1}$ $min^{-1}$ $m^{-2}$) (corr. to 200 mg $m^{-2}$) | 654.0 | − 0.5 |
| $t_{\frac{1}{2}}\beta$(min) | 53.0 | − 0.3 |

**Influence of a 24-hour infusion of hydroxyurea on the uptake of 5-iodo-2-deoxyuridine (IUDR) by bone marrow and tumour cells**

P.A. Phillip[1], K. Gatter[2], H. Turley[2], J. Carmichael[2] & A.L. Harris[1]

*[1]ICRF Clinical Oncology Unit, Churchill Hospital, Oxford OX3 7LJ; and [2]Department of Histopathology, John Radcliffe Hospital, Oxford OX3 9DU, UK.*

Hydroxyurea (HU) is a ribonucleotide reductase inhibitor which suppresses DNA synthesis in proliferating cells. Solid tumours readily acquire resistance to HU. IUdR is a thymidine analogue taken up by cells in the S-phase of the cycle via the thymidine salvage pathway. Myelosuppression is the dose limiting toxicity of IUdR. It has been shown using xenograft model that HU can reduce the uptake of IUdR by the normal cells allowing the selective introduction of IUdR into the nuclei of tumour cells that are resistant to HU (Bagshawe *et al.* (1987), *Br. J. Cancer*, **55**, 299).

We studied 10 pts with metastatic solid tumours judged to be resistant to conventional systemic therapy; melanoma (3), soft tissue sarcoma (2), lung adenoma (1), ovarian adenoma (1), adenoma unknown origin (1), breast ca (1), relapsed oat cell lung ca (1). Eight pts had received previous chemotherapy other than HU. HU was infused intravenously 1 g $h^{-1}$ for a total of 24 hours and 23 hours from the start of HU infusion, IUdR (100 mg $m^{-2}$) was given intravenously over 20–30 min. One hour from start of IUdR infusion bone marrow aspiration was performed (10 pts) using a 22 G/ 1.5 in paediatric lumbar puncture needle, followed immediately by aspiration from superficial tumour deposits (3 pts) or a malignant effusion (2 pts). IUdR labelling index (ILI) was determined using BU20 monoclonal antibody and counting the percentage of stained cells. Three pts on other chemotherapy received the same dose of IUdR followed by bone marrow aspiration before receiving any treatment to act as controls. ILI of bone marrow following HU was significantly lower compared to controls. Uptake by tumour cells was also very low (1 melanoma, 1 angiosarcoma, 1 ovarian adenoma, 1 adenoma unknown origin, and 1 breast ca). One pt (melanoma) had a significantly higher ILI pre-HU suggesting inhibition of the IUdR uptake by HU in that pt. It is possible that there is a differential sensitivity to HU for tumour and bone marrow cells operating at lower levels of exposure to HU.

**The effect of arterially administered adriamycin-loaded albumin microspheres on hepatic tumour implants in the rat**

J.A. Goldberg, D.J. Kerr, N. Willmott[1], A. McNicol & C.S. McArdle

*University Departments of Surgery & Pathology, Royal Infirmary, Glasgow; and [1]Department of Pharmacy, University of Strathclyde, Glasgow, Scotland, UK.*

The poor results of systemic chemotherapy for colorectal liver metastases has stimulated interest in regional chemotherapy. By loading anticancer agents into biodegradable particles which become trapped in small vessels following intrahepatic arterial (IHA) injection, it may be possible to increase further tumour drug exposure, and hence antitumour effect, without increasing systemic toxicity (Kerr *et al.* (1988), *Cancer*, **62**, 878). The aim of this study was to compare the antitumour effect of adriamycin-loaded albumin microspheres (ALAM) with that of the drug in solution. Sprague-Dawley rats bearing three-day-old hepatic implants of Walker tumour were divided into 4 groups: (A) sham operated; (B) operated and IHA 'empty' microspheres (2 mg); (C) operated and IHA adriamycin in solution, (30 µg) and (D) operated and IHA ALAM (2 mg containing 30 µg adriamycin). The animals were culled 4 days later, the tumours excised, weighed and submitted for histology.

| *Group (n = 6)* | | *Tumour weight [g] (mean ± SD)* |
|---|---|---|
| A | (sham) | 0.73±0.21 |
| B | (IHA empty microspheres) | 1.01±0.27 |
| C | (IHA drug in solution) | 0.74±0.12 |
| D | (IHA ALAM) | 0.45±0.08 |

Tumours were smallest in the group receiving IHA ALAM ($P = <0.05$, Mann-Whitney test). The data show that the cytotoxic effect of adriamycin is enhanced by incorporation into a slow-release mechanism.

### Intrahepatic arterial administration of mitomycin C microcapsules

D.J. Kerr, J. Goldberg, C.S. McArdle & T. Whatelely

*CRC Department of Medical Oncology and Department of Surgery, University of Glasgow & Department Pharmacy, University of Strathclyde, Scotland, UK.*

We have performed a series of clinical studies which indicate that angiotensin II can selectively increase the delivery of isotopically labelled microspheres to hepatic tumour metastases relative to normal liver by a factor of up to 10. As part of a continuing intrahepatic arterial chemotherapy programme ethylcellulose mitomycin C (MMC) microcapsules (80% drug w/w), 100–200 µm in diameter were synthesized by coacervation. *In vitro*, 80% of MMC was released in 1 hour at 37°C in stirred normal saline. Twenty mg of MMC in solution or microcapsular form was administered in random order via indwelling intrahepatic arterial portacaths to 10 patients with hepatic metastatic colorectal cancer. MMC concentrations were measured in peripheral venous blood using an HPLC method, with these results. (Mean ± SD).

| | *Peak conc (ng ml⁻¹)* | *AUC (ng ml⁻¹ h⁻¹)* | *Clearance (l h⁻¹)* | *T1/2B (h)* |
|---|---|---|---|---|
| Free MMC | 812±423 | 438±126 | 46±8 | 0.68±0.01 |
| | P<0.05 | P<0.05 | P<0.05 | P<0.05 |
| MMC micro-capsules | 80±70 | 143±43 | 140±31 | 1.56±0.04 |

There was no toxicity with either treatment. There is a clear potential for dose escalation with MMC-microcapsules and enhanced tumour targeting with intra-arterial angiotensin II.

### The effects of a somatostatin analogue (SMS 201-995) on the perfusion of liver metastases

D.M. Hemingway, D. Chang[1], S.A. Jenkins[1] & T.G. Cooke

*University Departments of Surgery, Glasgow Royal Infirmary and [1]Royal Liverpool Hospital, Liverpool, UK.*

We have previously reported that inhibition of the growth of experimental liver metastases by a somatostatin analogue, SMS 201-995, is partly due to a stimulation of hepatic reticuloendothelial activity. Since SMS 201-995 is vasoactive we have investigated its effects on hepatic haemodynamics in rats with liver tumours. Hepatic tumours were induced in rats by the intraportal inoculation of $10^6$ HSN sarcoma cells. Hepatic and tumour haemodynamics were measured using the dual microsphere method before and after the administration of SMS 201-995 (bolus dose 4 µg kg$^{-1}$ followed by continuous infusion of 4 µg kg$^{-1}$ h$^{-1}$). SMS 201-995 significantly decreased mean hepatic arterial flow 5.69 ± .87 to 2.357 ± .32 ml min$^{-1}$ ($P<0.02$ Students 't' test), and normal liver blood flow .43 ± .08 to .17 ± .02 ml min$^{-1}$ g$^{-1}$ ($P<.02$ Students 't' test) and decreased mean portal venous inflow (6.15 ± .64 to 5.43 ± .3 ml min$^{-1}$) and tumour blood flow (.23 ± .04 to .15 ± .04 ml min$^{-1}$ g$^{-1}$). These results indicate that SMS 201-995 markedly reduces hepatic and tumour blood flow, which may act synergistically with the effect of the analogue on RES activity to inhibit the growth of liver tumour.

### Intra-arterial phenylephrine increases drug delivery to liver tumour

D.M. Hemingway[1], D. Chang[2], D. Nott[2], S.A. Jenkins[2] & T.G. Cooke[1]

*[1]University Departments of Surgery, Royal Infirmary, Glasgow; and [2]Royal Liverpool Hospital, Liverpool, UK.*

The ineffectiveness of regional chemotherapy may be due in part to the relative avascular nature of most liver metastases. Manipulation of liver blood flow may alter tumour perfusion and delivery of drug to tumour. We have investigated the effects of intra-arterial phenylephrine on the distribution of a labelled marker to liver and liver tumour. Overt liver metastases were induced in rats by the intraportal inoculation of HSN sarcoma cells. $Tc^{99m}$ labelled methylene diphosphonate was injected into the hepatic artery either alone or 30 seconds after an intra-arterial bolus injection of 10 µg phenylephrine and the distribution of marker to tumour and normal liver examined. Phenylephrine caused a significant immediate retention of marker in tumour (mean % injected dose g$^{-1}$ 5.17 ± .55 before, 21.7 ± 5.69 after injection, $P<0.01$ Students 't' test). Distribution to normal liver also increased (mean % injected dose g$^{-1}$ 3.55 ± .15 before to 9.62 ± 2.45 after injection). This relative increase was maintained at 90 minutes after injection. Hepatic arterial vasoconstriction by phenylephrine causes significant retention of marker in both normal liver and hepatic tumour and may thus be of value in the regional delivery of drug to liver tumour.

### Hair loss due to cytotoxic chemotherapy: a prospective descriptive study

A. Tierney[1], J. Taylor[1], S.J. Closs[1], U. Chetty[2], A. Rodger[3] & R.C.F. Leonard[3]

*[1]Nursing Research Unit, University of Edinburgh; [2]Department of Surgery, Royal Infirmary; and [3]Department of Clinical Oncology, Western General Hospital, Edinburgh, UK.*

The main aim of a descriptive study of chemotherapy side-effects was to quantify and put in context the issue of alopecia due to various combination chemotherapy regimens. Hair loss and vomiting were anticipated as being the most difficult side-effects by three quarters of the patient sample (46, 76.7%). The visibility of alopecia placed this above vomiting in rank order of importance in 35/46 patients. All patients (pts) were offered scalp cooling (SC) at the first chemotherapy session but almost half discontinued after the first application and by the 6th only 16 were still receiving SC. Decline in use was almost entirely due to the CHOP patients experiencing hair loss and recognizing the ineffectiveness of SC. Conversely patients on CMF tended to continue with SC throughout. The problems of SC were treatment time, coldness and cap weight. At the end of chemotherapy 30/32 CHOP pts required a wig as against only 1/21 CMF pts. This last pt suffered severe hair loss after the second chemotherapy cycle. Pattern and rate and type of hair loss was variable for CHOP being mild in some patients at the start becoming severe later, whereas with other patients total hair loss occurred after the first treatment. For CMF uniformly gradual thinning of the hair occurred. Patchy hair loss occurred only in the patient who eventually lost all her hair. Body hair loss (eye lashes, eye brows, legs, axillary and pubic) was not significantly different from scalp loss so that this further measurement did not provide supportive evidence for the effectiveness of SC. We conclude pts require realistic information about the pattern and rate of hair loss, the BACUP booklet being recommended. Counselling may be of further benefit for individuals especially disturbed by hair loss. SC for CHOP regimens is ineffective but should be available for CMF regimens as a standard procedure on the basis of informed consent.

### Side-effects and chemotherapy: anticipation, information and experience

A. Tierney[1], J. Taylor[1], S.J. Closs[1], U. Chetty[2], A. Rodger[3] & R.C.F. Leonard[3]

*[1]Nursing Research Unit, University of Edinburgh; [2]Department of Surgery, Royal Infirmary; and [3]Department of Clinical Oncology, Western General Hospital, Edinburgh, UK.*

In a prospective descriptive study of patients (pts) receiving combination chemotherapy for breast cancer (adjuvant and locally advanced), 60 consecutive pts were interviewed before, during each treatment cycle (max 6) and at the completion of chemotherapy using CHOP (32 pts), CMF 21, VAP 6 and CAF 1 pt. Although pts were informed prospectively, their experience of side-effects was much more diverse than forewarned. Thirty-five (58.5%) expected hair loss to be the most difficult symptom whereas in reality only 13 (21.7%) eventually rated this as the worst side-effect even though 65% experienced it. Of all side-effects, tiredness was reported most frequently and at any time point never less than 87.5% of patients had this symptom. Following this, in order, nausea, anorexia, mouth soreness, pain, vomiting and sore eyes were reported. Overall about one third of patients found chemotherapy side-effects less than anticipated but the general experience was that the diversity of side-effects had been underestimated by the doctors and nurses giving advice before treatment. Approximately 75% of patients felt that a booklet giving full information would have been valuable. Generally doctors and nurses warning about side-effects underestimate the importance of fatigue; patients had been warned about it in only 50% of cases. Research in other treatment groups with different regimens is required to test the generality of these results.

### Limitations in the clinical utility of serum markers CA153 and HMFG2 for monitoring breast cancer

P. Bliss[1], R.C.F. Leonard[1], J. Fisken[2], S.S. Akhtar[1], M. Palmer[2], U. Chetty[3], A. Rodger[1] & J.E. Roulston[3]

*[1]Department of Clinical Oncology; [2]Clinical Surgery; and [3]Clinical Chemistry, University of Edinburgh, Scotland, UK.*

The serum from 57 patients with local and advanced breast cancer was studied using two antibodies against glycoproteins associated with human breast cancer. Ca153 is a commercial immunoradiometric assay which claims high sensitivity for monitoring disease. HMFG2 is a cell surface mucin associated with breast cancer and possibly of prognostic significance in studies of primary tumour immunopathology. Immunoassay for HMFG2 was developed locally with the assistance of Unipath plc. Serology data were correlated against clinical and radiological assessments of patients classified as having local regional disease or metastatic disease (including SCF lymph nodes as M1). Most patients with LR were preoperative, T1–T3.

| | *Ca153 (nr 0–30)* | | | | *HMFG2 (nr 0–40)* | | | |
|---|---|---|---|---|---|---|---|---|
| | *No* | *Median* | *Range* | *Elevated* | *No* | *Median* | *Range* | *Elevated* |
| LR | (22) | 13 | (6–48) | 1/22 | (18) | 10 | (0–>200) | 2/18 |
| Met | (30) | 70 | (11.5–3530) | 22/30 | (30) | 15 | (0–>200) | 6/30 |

In this series only Ca153 was adequately sensitive in the patients with metastatic disease. The sensitivity was 72%. HMFG2 alone is not an adequate marker for disease activity in either locoregional or metastatic breast cancer. However, it may be of value used in conjunction with Ca153. Ca153 clearly deserves further study in a larger series of patients.

### A phase I/II study of recombinant human granulocyte-macrophage colony-stimulating factor in patients receiving intensive chemotherapy for small cell lung cancer

H. Anderson, H. Gurney, J. Radford, W.P. Steward, A. Kamthan, J. Chang & N. Thatcher

*Christie Hospital, Manchester, UK.*

Seventeen patients with small cell lung cancer have been entered into a dose ranging Phase I/II study using rhGM-CSF (Glaxo). In the Phase I study patients received 50, 150, 300 or 500 $\mu g\, m^{-2}$ GM-CSF for 10 days as a daily subcutaneous injection. After 4 days off therapy patients received chemotherapy with adriamycin 50 $\mu g\, m^{-2}$ i.v., ifosfamide 5 $g\, m^{-2}$ with MESNA 3 $g\, m^{-2}$ i.v. on Day 1 and VP16 120 $mg\, m^{-2}$ i.v. daily for three days. In the Phase I study there was a significant rise in the neutrophils and eosinophils and to a lesser extent monocytes at all dose levels and there was some evidence of a dose-response relationship. In the Phase II study patients received the same dose of GM-CSF as in Phase I. GM-CSF commenced 24 hours after the last dose of chemotherapy and was given for 14 days. Patients were randomized to receive GM-CSF with either odd or even courses of chemotherapy. Despite partial abrogation of the neutropenia associated with chemotherapy ($P = 0.04$), GM-CSF failed to reduce the incidence of severe infection – 6 episodes on GM-CSF and 7 not on GM-CSF ($P = 0.9$). Timed blood counts over 24 hours showed that peak stimulation of leucocytes occurred 8–12 hr following GM-CSF. Toxicity, lethargy, myalgia and bone pain, occurred at all dose levels but was manageable. GM-CSF given as a once daily subcutaneous bolus is not effective in preventing infection following intensive chemotherapy for small cell lung cancer despite reduction of chemotherapy-associated neutropenia.

### Pharmacokinetics of epirubicin (EPI) in patients with abnormal liver biochemistry

C. Twelves, N. Dobbs, L. Summers, P. Harper, M. Richard & R. Rubens

*ICRF Clinical Oncology Unit, Guy's Hospital, London SE1 9RT, UK.*

Anthracyclines are metabolized in the liver and empirical dose reductions are often made in patients with impaired liver function. We investigated the effects of abnormal liver biochemistry on the pharmacokinetics of EPI in 12 patients with breast cancer (3 with normal bilirubin, and aspartate transaminase (AST) $< 2 \times$ normal (N); 9 who had liver metastases and AST $> 2 \times N \pm$ raised bilirubin). Treatment was with EPI 25 mg m$^{-2}$ i.v. weekly in all patients. Timed blood samples were taken for 48 hours after EPI. Plasma levels of EPI were measured by high performance liquid chromatography and fitted to a 3-compartment model. The area under the concentration-time curve to 48 hours (AUC) was calculated by the trapezoidal method, and terminal half-life ($T_{\frac{1}{2}}$) was estimated.

| | *Bilirubin = N* *AST<2 × N* *(n = 3)* | *Bilirubin = N* *AST>2 × N* *(n = 4)* | *Bilirubin > N* *AST>2 × 1* *(n = 5)* |
|---|---|---|---|
| AUC (ng ml$^{-1}$ h$^{-1}$) | 606 | 1011 | 1301 |
| $T_{\frac{1}{2}}$ (h) | 23 | 33 | 43 |

AST correlated strongly with AUC (r = 0.83; $P<0.001$) and $T_{\frac{1}{2}}$ (r = 0.87; $P<0.001$). We conclude epirubicin pharmacokinetics are abnormal in patients with disturbed liver biochemistry. Total exposure to EPI is increased in patients with either raised serum bilirubin or AST > 2 × N.

### Metabolism of ethanol and of fusel alcohols by rat oesophagus

V.M. Craddock & M. Abbs

*MRC Toxicology Unit, Woodmansterne Road, Carshalton, Surrey, UK.*

Consumption of alcoholic beverages is the main cause of oesophageal cancer for men in Europe and North America, but surprisingly the ability of the oesophagus to metabolize ethanol has not been studied. Many surveys in USA and France have shown that, for the same amount of ethanol, certain spirits, especially apple brandy, present a very much greater risk than does wine or beer. The high fusel alcohol content of many spirits, especially apple-based spirits, compared with wine and beer, could be the cause. The metabolism by oesophageal cytosol of ethanol, and of 3-methyl-butanol (3-Mb) and β-phenethylalcohol, two major fusel alcohols, was studied. Specific activity (SA) is expressed as nM NADH formed from $NAD^+$ per mg cytosol protein per minute, measured over the first 30 sec incubation. The rate of metabolism of ethanol increased with an increase in substrate concentration, until at 2 M the SA was about forty times greater than that of unfractionated liver enzyme measured at its optimum substrate concentration (7.5 mM). This high activity at high substrate concentrations has been reported also for the minor x-ADH of liver, and for stomach and corneal ADH. The oesophageal enzyme, however, differs from other forms in its sensitivity to inhibition by pyrazole, 4-methylpyrazole, and by 1,10-phenanthroline. The fusel alcohols were also actively metabolized at high substrate concentrations by oesophageal alcohol dehydrogenase, the SA being about eight times that of liver measured at its optimum substrate concentration. The oxidation of 3-Mb, with the formation of 3-methyl-butaldehyde, could be responsible for the dramatic increase in basal cell replication which occurs in oesophagus after intubation of 3-Mb, and in this way spirits containing fusel alcohols could promote carcinogenesis.

### The assay of the binding of aflatoxin $B_1$ to rat albumin

I.O. Olubuyide, G.E. Neal & D.J. Judah

*Toxicology Unit, MRC Laboratories, Carshalton, Surrey SM5 4EF, UK.*

The potent hepatotoxin and hepatocarcinogen, aflatoxin $B_1$ ($AFB_1$) is activated by mixed function oxidases to metabolites which undergo macromolecular binding. The binding of an $AFB_1$ metabolite, $AFB_1$-dihydrodiol, to serum albumin appears to have potential for assaying exposure to the carcinogen in human populations, using ELISA techniques. The feasibility of this has been examined using male Fischer rats exposed to $AFB_1$. The $AFB_1$ dose/albumin binding relationship has been examined using [$^3$H]-labelled $AFB_1$ with essentially similar results to those obtained by Wild *et al.* (1986, *Carcinogenesis*, **7**, 853). A procedure for the purification and concentration of the albumin adducts has been examined using pronase digestion of [$^3$H] $AFB_1$ adducted albumin followed by Sep Pak and affinity column chromatography. Radiochemical recoveries were assayed at each step in the protocol. The pronase digestion has been optimized and the distribution of label in the hydrolysate examined by HPLC. The results using *in vivo* adducted rat albumin have been compared with those obtained using albumin modified *in vitro* by microsomally activated $AFB_1$. Assays of the level of $AFB_1$ adducts based on radiolabel have been compared with results obtained from ELISA assays. A fraction of the adducted $AFB_1$, detected by radiolabel, was not recognized by either the affinity column or the ELISA. This was partly compensated by the highly immunoreactive nature of the fraction retained by the affinity column presumably reflecting the chemical structure of the $AFB_1$-adduct used as the immunogen in raising the polyclonal antibody. The overall efficiency of the albumin/ELISA technique developed will be assessed.

### New measurements of thymidine kinase using HPLC

P. Mullan, B. Armstrong, B.M. Hannigan & P.G. McKenna

*Biomedical Sciences Research Centre, University of Ulster, Coleraine, N. Ireland BT52 1SA, UK.*

Thymidine kinase (TK) (EC 2.7.1.21) is a key enzyme in the generation of substrates for DNA synthesis. The TK enzyme converts thymidine (Thd) to thymidinemonophosphate (TMP). This phosphorylation is the only pathway to introduce Thd into the DNA metabolism, and for this reason this enzyme is called a salvage enzyme. TK activity is low in non-dividing cells but increases before the onset of DNA synthesis. The growth rate of different tumours correlates in many cases with cellular TK activity. It is therefore clear that measurement of serum and cellular TK levels may be a useful adjunct to other forms of markers for cellular proliferation and cancer diagnosis. An alternate method for TK quantification is reported, using the ability of the enzyme to convert halogenated analogues of thymidine (e.g. bromodeoxyuridine (BrdU), iododeoxyuridine (IdU) etc.) to their respective monophosphates. These monophosphate products are measured using high performance liquid chromatography (HPLC). Preliminary results have shown iododeoxyuridine to be the preferred substrate of choice as IodUMP determination is performed at 290 nm, largely excluding absorbance from other cellular components. This work provides a highly reproducible method for TK assessment and compares favourably with previous methods of enzyme measurement.

### A technique for measuring DNA single strand breaks and alkali labile sites in individual cells

V.J. McKelvey[1], R.R. Tice[2] & N.P. Singh[3]

[1]*Department of Biological and Biomedical Sciences, University of Ulster, Coleraine, N. Ireland, UK;* [2]*Integrated Laboratory Systems, PO Box 13501, Research Triangle Park, N.C. 27709; and* [3]*Biology Department, Eastern Washington University, Cheney, W.A. 99004, USA.*

An electrophoretic assay capable of detecting DNA single strand breaks and/or alkali labile sites in single cells was recently introduced by Singh *et al.* (1988), *Exp. Cell Res.*, **178**, 184). The importance of this assay lies in both its requirement for only extremely small cell samples (e.g. 10 µl blood) and in its ability to evaluate intercellular differences in DNA damage or repair in any cell type. This paper presents a detailed working protocol of the Single Cell Gel (SCG). Assay technique and its value in assessing DNA damage in, for example, human peripheral blood lymphocytes following *in vitro* treatment with u.v. light. Two doses of u.v. light were used, namely $6\,J\,m^{-2}$ and $20\,J\,m^{-2}$, and the lymphocytes were harvested at 1, 2 and 6 hours following treatment. The irradiated lymphocytes were embedded in low melting point agarose on microscope slides, lysed by detergents and electrophoresed under alkaline conditions. Untreated lymphocytes were processed in parallel. Changes in DNA migration patterns were observed by ethidium bromide fluorescence and measured using a micrometer. A clear dose response was observed at each of the three harvesting times examined, and the extent of the u.v.-induced damage was observed to decrease over time, until at 6 hours following $6\,J\,m^{-2}$ u.v. treatment the lymphocytes examined virtually all displayed the control level of DNA migration. The potential of the assay for DNA damage and repair studies is indicated.

### Assessment of unscheduled DNA synthesis (UDS) in murine lymphoma cells exposed to enzymatically generated oxidants

S-A. Richardson, B.M. Hannigan & P.G. McKenna

*Biomedical Sciences Research Centre, University of Ulster, Coleraine, N. Ireland BT52 1SA, UK.*

Oxidants (oxygen radicals) are known to induce DNA damage and, as such, appear to have an important role in various biological processes e.g., ageing, mutagenesis and carcinogenesis. Assessment of the precise nature of the various forms of oxidative DNA damage may allow correlations to be drawn between oxidant activity and pathological sequelae. Thus, cellular mechanism for the repair of oxidant damage must also be determined. In this study, oxidants were generated using the enzymatic system xanthine/xanthine oxidase (X/XOD) in the presence of L5178Y(S) cells, a radiosensitive murine lymphoma cell-line. Xanthine oxidase acts aerobically upon xanthine to generate oxidants as follows: $X + O_2 \dashrightarrow O_2^{\cdot -}$ + uric acid. $O_2^{\cdot -}$ can give rise to $H_2O_2$ and the hydroxyl radical $OH^{\cdot}$. The exact mechanism by which cells are damaged while exposed to X/XOD is still uncertain. The cells' capacity for excision repair was determined by two methods: an incorporation assay (Larcom & Smith (1988), *J. Natl Can. Inst.*, **80**, 110) and autoradiography (Cleaver & Thomas (1981), In: *DNA Repair. A Laboratory Manual of Research Procedures*, 277), the latter method being regarded as a more accurate assessment of UDS. Results indicate that excision repair occurs in X/XOD treated cells, the extent varying according to the duration of oxidant treatment, period of cell recovery and duration of exposure to $^3$H-thymidine. The continuing identification of repairable oxidative DNA damage is a necessary strategy in pursuing a better understanding of the mechanisms of oxidant induced mutagenicity and carcinogenicity.

### Study of the miscoding properties of O$^6$-methylguanine using synthetic DNA primer templates

H.B. Tan & P.F. Swann

*CRC Nitrosamine-induced Cancer Research Group, Department of Biochemistry, University College London, Gower Street, London WC1E 6BT, UK.*

O$^6$-alkylguanine in the template DNA misdirects the incorporation of T during DNA synthesis and the pre-eminent role of this alkylated base in nitrosamine carcinogenesis is thought to be a consequence of the resulting mutation. The accepted belief that the miscoding occurs because O$^6$-alkylG forms a mispair with T which is more stable than the pair with C has recently been disproved by melting point studies which have shown that O$^6$-alkylG:T pairs are less stable than O$^6$-alkylG:C base pairs. This has reopened the question of why O$^6$-alkylation of G produces G.C to A.T transition mutations. To clarify this, we are carrying out detailed analysis of DNA synthesis on synthetic alkylated templates using the methods of Boosalis *et al.* ((1987), *J. Biol. Chem.*, **262**, 14689) and Kuchta *et al.* ((1988), *Biochemistry*, **27**, 6716). These are not yet complete but the preliminary results show that with the Klenow fragment, the discrimination in favour of the incorporation of T opposite O$^6$-methylG occurs during the addition of the base after the mispaired base as well as during the addition of the mispaired base itself. The incorporation of the base after a T is much more rapid than when a C is opposite the O$^6$-methylG. Since the extent of the 3′-5′ proofreading is inversely related to the rate of addition of the following base, this would contribute to the formation of O$^6$-alkylG:T pairs by allowing less removal of T than of C from the alkylated base pair by exonucleolytic proofreading. The reduced rate of addition of both the C and of the base following it may be explained by NMR studies (Kalnik *et al.* (1989), *Biochemistry*, **28**, 6170) which have shown that O$^6$-ethylG:C pairs have a 'wobble' configuration which is accompanied by significant changes to the geometry of the phosphodiester links in the 'C' strand on both the 3′ and 5′ sides of the C, while the O$^6$-alkylG:T pair retains the Watson-Crick configuration with relatively normal geometry of the phosphodiester links. These kinetic and NMR studies suggest that the fidelity of DNA synthesis depends more on the conformation of the base pairs than on their stability.

### Inhibition of DNA methylation

P.A. Hepburn & M.J. Tisdale

*Cancer Research Campaign Experimental Chemotherapy Group, Pharmaceutical Sciences Institute, Aston University, Birmingham B4 7ET, UK.*

Unmodified calf thymus DNA does not act as a substrate nor does it inhibit the transfer of methyl groups from S-adenosyl-L-methionine to *M. lysodeikticus* DNA, by partially purified eukaryotic DNA methylase. It has been observed, however, that if the calf thymus DNA is modified by various agents, it becomes an inhibitor of DNA methylation: **1.** Modification by monofunctional alkylating agents such as temozolomide or ethazolastone causes a dose-dependent inhibition of methylation. However, modification by bifunctional alkylating agents capable of cross-linking DNA such as chlorambucil produces poor inhibitors. **2.** Treatment with $^{60}$Co radiation causes an inhibition which is dose related and correlates with the appearance of single strand breaks. **3.** DNA treated with DNAase I which produces single and double strand breaks also causes an inhibition of methylase activity, whilst sonicated DNA and DNA treated with the restriction enzyme MspI does not.

Damage to DNA by these treatments may be related to an increased exposure of particular bases, as certain poly nucleotides have been shown to have an inhibitory action. In particular, a potent inhibition of DNA methylase activity has been observed with poly [G], poly [I], poly [X], poly [dG], poly [dC] but not poly [A], poly [U] or poly [C]. These results point out the importance of the O$^6$ position of guanine in the binding of DNA methylase to dCpdG, the recognition sequence for cytosine methylation. The inhibition produced by the various modifications and the poly-nucleotides appears to be due to an increased binding of the enzyme to the inhibitor polynucleotide. These results may have important implications in the mechanism of the alteration in pattern of gene expression in neoplastic cells.

**Accumulation of MCF-7 human mammary carcinoma cells in the G0/G1 phase of the cell cycle following administration of a luteinizing hormone releasing hormone agonist**

P. Mullen & W.R. Miller

*ICRF, Medical Oncology Unit, Western General Hospital, Edinburgh EH4 2XU, UK.*

The Luteinizing Hormone-Releasing Hormone agonist (LHRHa), buserelin, has been shown to inhibit the growth of MCF-7 cells in culture (Miller *et al.* (1985), *Nature*, **313**, 231). The aim of the present study was to determine if such growth inhibition was accompanied by alterations in the cell cycle distribution. Effects of buserelin were tested out on MCF-7 cells previously shown to be sensitive to LHRHa (MCF-7 1s). A second clone of MCF-7s which were insensitive (MCF-7 1i) were also studied as a negative control. Growth experiments were carried out by plating cells ($0.5 \times 10^6$) in 60 mm petri dishes containing DMEM culture medium (4 ml) in the absence and presence of buserelin ($10^{-6}$ M, $10^{-7}$ M and $10^{-8}$ M). After 1, 2, 3 and 4 days cells were harvested by trypsinisation, counted with a haemocytometer and then subjected to DNA analysis using a Coulter EPICS C flow cytometer. The MCF-7 1s cells were inhibited by busereline in a dose-related manner. Furthermore, this was accompanied by major alterations in the cell cycle distribution. Treatment with buserelin produced an accumulation of cells in the G0/G1 phase of the cell cycle and a concomitant decrease in both the S and G2/M phases. No such changes were seen in the LHRH insensitive cell line in which buserelin produced no significant change in cell numbers. These results show that the growth inhibitory effects of buserelin in the MCF-7 cell line are associated with major changes in the cell cycle parameters.

**Direct effects of GnRH analogues in prostatic cancer**

A. Qayum[1], W. Gullick[1], P. Abel[3], G. Williams[3], K. Melon[4], D. Neal[4], K. Sikora[2] & J. Waxman[2]

*[1]ICRF Unit for Molecular Oncology, [2]Clinical Oncology and [3]Urology, Hammersmith Hospital; and [4]Freeman Hospital, UK.*

We report high affinity binding (kd = 50 M) of a gonadotrophin releasing hormone (GnRH) analogue resulting in biphasic growth modulation of the human androgen-sensitive prostatic cancer cell line. LNCaP. This was in contrast to low affinity, non specific binding (kd = 10 μM), without biological effect in the human androgen insensitive line DU145. GnRH-like immunoreactivity was demonstrated in the concentrated culture media from both cell lines by a GnRH radioimmunoassay, and this peptides elution profile on HPLC was identical in nature to GnRH. Nineteen of 22 malignant tumours and 49 of 54 benign prostatic tumours exhibited high affinity binding. Fourteen of 19 malignant tumours and 17 of 49 benign tumours exhibiting high affinity binding contained GnRH-like immunoreactivity suggesting that this system may be involved in prostatic epithelial growth *in vivo*.

**The effects of progestins/anti-progestins on proliferation and progesterone receptor expression in human breast cancer cells**

H.W. van den Berg, J. Martin & M. Lynch

*Department of Therapeutics and Pharmacology, The Queen's University of Belfast, UK.*

We studied the effects of the progestins, medroxyprogesterone acetate (MPA) and ORG 2058, and the anti-progestins, RU 38.486 and ZK 98.299, on progesterone receptor (PGR) expression and proliferation of ZR-75-1 human breast cancer cells and variants differing in their steroid hormone receptor profiles. The relative affinities of progestins/anti-progestins for PGR in an oestrogen (E2) independent variant of the ZR-75-1 line (ZR-PR-LT) expressing high basal levels of the receptor were ORG 2058 = 100, MPA = 200, RU 38.486 = 44, ZK 98.299 = 30. A 5-day exposure of ZR-75-1 cells to 10−9 M E2 in the presence of 10−9 M MPA resulted in an almost complete blockade of E2 induction of PGR. 10−9 M MPA alone reduced PGR expression in ZR-PR-LT cells to 20% of basal levels whilst 10−9 M RU 38.486 reduced PGR expression by 50%. In contrast, neither ORG 2058, nor ZK 98.299, treatment (10−9 M) resulted in loss of PGR. MPA was the most potent anti-proliferative agent in all cell lines tested, but the ZR-PR-LT line was only marginally more sensitive than the parent line despite expressing a 20-fold higher basal PGR concentration. Surprisingly, the growth inhibitory response to MPA, RU 38.486 and ZK 98.299 was greatest in a tamoxifen-resistant variant (ZR-75-9a1) lacking detectable PGR. These results indicate a poor relationship between cellular PGR content and proliferative response to progestins/anti-progestins. The reason for the marked differences in the ability of these agents to down-regulate PGR is not known but may be related to differences in intrinsic glucocorticoid/anti-glucocorticoid activity.

**Interferon alpha receptor expression by human breast cancer cells**

J. Martin, B. McKibben & H.W. van den Berg

*Departments of Therapeutics, Pharmacology and Medicine, The Queen's University of Belfast, UK.*

Recombinant interferon alpha 2b (IFN) increases oestrogen receptor (ER) expression in ZR-75-1 human breast cancer cells (van den Berg *et al.* (1987), *Br. J. Cancer*, **55**, 255) but we have been unable to demonstrate induction of ER in a tamoxifen-resistant, ER negative, variant line (ZR-75-9a1). In this study we have investigated the relationship between IFN receptor expression and steroid hormone receptor content in ZR-75-1 cells, the tamoxifen-resistant variant and an oestrogen independent subline (ZR-PR-LT). The latter line lacks binding sites characteristic of the Type 1 ER but expresses elevated levels of progesterone receptor (PGR). IFN was labelled with $^{125}$I using the Iodogen reaction and purified by HPLC to a specific activity of 298 Ci mM$^{-1}$. Binding of $^{125}$-I IFN to whole cells at 4°C was determined in the presence and absence of excess cold IFN. ZR-75-1 cells contained 1498 ± 541 IFN receptors/cell which fell to 802 ± 447 receptors/cell during a 5 day exposure to 10−9 M oestradiol (E2). IFN and E2 therefore appear to have opposite effects on expression of each other's receptor in this cell line. This E2 induced reduction in IFN receptor expression is accompanied by a 6-fold increase in PGR concentration in ZR-75-1 cells. However, there was no significant reduction in IFN receptor expression in ZR-PR-LT cells associated with their elevated basal PGR content. An inverse relationship between IFN and PGR receptor expression is apparent in the ZR-75-9a1 line which lacks PGR and expresses the highest concentration of IFN receptors (3443 ± 389 sites per cell). This observation suggests that the apparent inability of IFN to induce ER in this cell line is not a consequence of failure to express IFN receptors.

**Characterization of ligand-binding and processing by gastrin releasing peptide receptors in small cell and non-small cell lung cancer cell lines**

C. Cardona, J.G. Reeve & N.M. Bleehen

*MRC Clinical Oncology and Radiotherapeutics Unit, MRC Centre, Hills Road, Cambridge CB2 2QH, UK.*

Bombesin (BN) and its mammalian homologue gastrin releasing peptide (GRP) have been shown to stimulate the growth of small cell lung cancer (SCLC) cell lines *in vitro*.

However, no obvious correlation was observed between the *in vitro* response to BN/GRP and the presence of specific BN/GRP receptors. Furthermore, the processing events that occur after BN/GRP binding have not been investigated in lung tumour cell lines. To gain further insight into the mechanisms involved in the mitogenic action of BN/GRP, the expression of GRP receptors and the kinetics of GRP binding and internalization have been studied in a panel of 10 SCLC and 3 non-SCLC (NSCLC) cell lines. For the detection of GRP receptors $10^6$ cells were incubated with 0.5 nM $^{125}$IGRP for 30 minutes at 37°C in the presence or absence of 1 $\mu$M cold GRP. Cells were pelleted, washed and specific binding determined. In contrast to previous studies, specific GRP binding sites were detected consistently on all SCLC and NSCLC cell lines examined although GRP binding varied between lines (0.06-2.01 fmol $10^{-6}$ cells). For all cell lines, binding of $^{125}$IGRP increased rapidly with time, peaked after 15–60 minutes depending on cell line, and subsequently decreased. Extraction of surface-bound ligand at low pH indicated that the iodinated peptide was internalized within minutes in both SCLC and NSCLC cells. Pre-incubation of cells with GRP markedly reduced the subsequent binding of $^{125}$IGRP to SCLC cells but not to NSCLC cells. These results indicate that GRP receptors are present on both SCLC and NSCLC cells, that in both cell types GRP is internalized and degraded, but that in contrast to NSCLC cells, surface receptors for GRP on SCLC cells are down-regulated. Hence regulation of the proliferative response to GRP stimulation may differ in these cells.

**Cell membrane receptors for granulocyte and granulocyte/monocyte colony stimulating factors on bone marrow cells from leukaemia patients: an autoradiographic study**

W.S. Gilmore, M. McCabe, G.B. Nevin & C.P. McGuckin

*Biomedical Sciences Research Centre, University of Ulster, Cromore Road, Coleraine BT52 1SA, UK.*

Granulocyte colony stimulating factor (G-CSF) and granulocyte/monocyte colony stimulating factor (GM-CSF) are haemopoietic growth factors involved in the proliferation and function of cells of the granulocytic and monocytic cell lineages. These growth factors act on their target cells via cell membrane receptors. On binding to the cell surface receptor, the growth factor is thought to activate an, as yet, undefined signal transduction mechanism resulting in cell proliferation. In the present study, binding of radiolabelled G-CSF and GM-CSF to bone marrow cells from patients with haematological malignancies was observed. Recombinant human G-CSF (Amersham International PLC) was radioiodinated and radioiodinated GM-CSF and unlabelled GM-CSF were obtained from Amersham International PLC. Bone marrow films were prepared from haematopoietic cells obtained from trephine biopsy during routine haematological investigation of patients with various haematological disorders. The labelled CSFs were then reacted with the bone marrow smears, which were then dipped in photographic emulsion and incubated for 7 days in the dark and developed. Radiolabelled G-CSF bound to cells of granulocytic origin and patients with chronic granulocytic leukaemia showed increased binding. Radiolabelled GM-CSF bound to a wide range of cells including megakaryocytes.

**Biodistribution studies in mice with synthetic polypeptide-drug conjugates**

J.A. Clegg[1], F. Hudecz[2], M.V. Pimm[1] & R.W. Baldwin[1]

[1]*Cancer Research Campaign Laboratories, University of Nottingham, UK; and* [2]*Institute of Organic Chemistry, Eotvos University, Budapest, Hungary.*

Synthetic macromolecules are of interest as carriers for anticancer agents for both non-specific and site-specific modulation of their biodistribution. In the present study, the biodistribution in mice has been examined of a range of synthetic branched polypeptides. These have a poly-L-lysine backbone and are substituted at epsilon amino groups with side chains of three alanine amino acids with or without another terminal amino acid. Polypeptides with terminal Leu, D-Leu or Pro are basic and polycationic, while those with terminal Glu are both acidic and basic and thus amphoteric under physiological conditions. Two groups of polypeptides of almost identical composition but different molecular sizes (46 kDa and 200 kDa) were examined. To assess their biodistribution, the polypeptides were labelled with $^{125}$I by reaction with iodinated Bolton-Hunter reagent. Polycationic polypeptides were cleared rapidly from the circulation, the liver being the major site of clearance. Amphoteric polypeptides showed the best blood survival, and this was seen with polypeptides of both molecular sizes. Conjugation of methotrexate or daunomycin to these amphoteric polypeptides at a molar ratio of up to 60:1 did not compromise their biodistribution. These findings suggest that these amphoteric branched polylysine-drug conjugates are candidates for further conjugation to site specific targeting agents such as monoclonal antibodies.

**Anti-methotrexate monoclonal antibodies: their production, characterization and potential uses**

A.D. Curran, E. Jacobs, M.V. Pimms, R.A. Robins & R.W. Baldwin

*Cancer Research Campaign Laboratories, University of Nottingham, Nottingham NG7 2RD, UK.*

A panel of monoclonal antibodies has been raised against the antifolate drug methotrexate, to assess their potential for use in drug assays and cancer therapy. Balb/c mice were immunized with methotrexate (MTX) covalently linked to human serum albumin (Garnett *et al.* (1985), *Anti-Cancer Drug Design*, **1**, 3), with standard techniques being used to produce the hybridomas. Hybridomas 420 ($IgG_1$) and 533 ($IgG_2b$) were selected in media containing HMT; 634.4 ($IgG_1$) and 634.42 ($IgG_1$) were selected initially in HAT media but were quickly transferred to media containing H and T only. Antibodies were purified by binding to protein A-sepharose in PBS at pH 8.0, and eluting with 3M NaSCN followed by dialysis against PBS at pH 7.2. ELISA blocking assays showed 420 and 533 to be more specific for MTX conjugated to HSA than MTX alone, whereas 634.4 and 634.42 reacted equally well with free drug. Blocking assays with MTX analogues and structural components of the MTX molecule have shown that 634.4 recognises the pteridine ring end of the drug, whereas 634.42 recognises the central region of the molecule. *In vitro* the antibody has potential for use in drug assay development: 634.42 has been tested in a quantitative competitive ELISA for the detection of methotrexate, and could detect free drug in the range of 1 to 100 $\mu$g. For *in vivo* utilization, the 634.4 hybridoma is being fused with the anti-CEA hybridoma 365 to generate an antitumour associated antigen/anti-methotrexate bispecific antibody for the site specific delivery of free or carrier-conjugated MTX.

**Cytotoxicity of monoclonal antibody-targeted methotrexate against methotrexate-resistant tumour cell lines**

K. Affleck & M.J. Embleton

*Cancer Research Campaign Laboratories, University of Nottingham, Nottingham NG7 2RD, UK.*

Several methotrexate (MTX)-resistant sublines of osteogenic sarcoma cell line 791T and colon carcinoma cell line C170 have been derived by continuous selection in the presence of MTX and 12-O-tetradecanoylphorbol 13-acetate. Studies including assays of the uptake and binding of $^{3}H$-MTX and fluoresceinated MTX have determined that resistance in these sublines was predominantly due to diminished MTX transport, and that none of them appeared to overproduce the MTX target enzyme dihydrofolate reductase. A conjugate of the anti-791T monoclonal antibody 791T/36 linked to MTX via human serum albumin was provided by Dr M.C. Garnett. This was cytotoxic selectively for cells bearing the 791T/36-defined antigen, and was found to be as cytotoxic to most of the MTX-resistant 791T sublines as it was to parental 791T cells. Another conjugate, constructed by linking the anti-colorectal carcinoma monoclonal antibody 505/4/6 directly to MTX, was also found to be more cytotoxic than free drug to some of the MTX-resistant C170 sublines, although these were not as susceptible to conjugates as were parental C170 cells. It is suggested that targeting to a cell surface antigen can partially or wholly overcome acquired drug resistance caused by deficient transport mechanisms.

**A syngeneic anti-idiotypic antibody to an antitumour antibody which induces cellular and humoral antitumour responses**

M. Doran, L.G. Durrant, R.A. Robins, E.B. Austin & R.W. Baldwin

*Cancer Research Campaign Laboratories, University of Nottingham, Nottingham NG7 2RD, UK.*

Anti-idiotypic antibodies reactive with the binding site of antibodies to tumour-associated antigens can generate antitumour responses in tumour patients. 791T/36 is a mouse monoclonal antibody which recognizes the gp72 human tumour-associated antigen (791Tgp72). Patients receiving 791T/36-RTA produce strong anti-idiotypic antibody responses and some develop antitumour responses. 730/71 is an $IgG_1$ syngeneic anti-791T/36 anti-idiotypic antibody. It specifically blocks the binding of 791T/36 (IgG2b) and ID12 (an $IgG_{2a}$ class switch variant of 791T/36) to 791Tgp72. The 730/71 defined idiotype on 791T/36 is recognized by anti-idiotypic antibodies in the serum of most of the patients receiving 791T/36-RTA. 730/71 can evoke cellular and humoral immune responses against 791T and C170 cells (human tumour cell lines expressing 791Tgp72). Mice primed with 730/71, either alone, or with adjuvants, show a significantly enhanced delayed type hypersensitivity response to both 791T and C170 cells, compared with mice primed with an irrelevant $IgG_1$. Mice, rats and rabbits immunized with 730/71 produce anti-idiotypic antibodies which block the binding of 730/71 to 791T/36. In each species, a proportion of the anti-idiotypic antibodies bind 791T cells. These results show that 730/71 has a potential role in the immunotherapy of human cancer.

**A human monoclonal anti-idiotypic antibody with potential for induction of antitumour immunity**

E.B. Austin, R.A. Robins, L.G. Durrant & R.W. Baldwin

*Cancer Research Campaign Laboratories, University of Nottingham, Nottingham NG7 2RD, UK.*

A human monoclonal anti-idiotypic antibody 105AD7 was produced by fusing a mouse-human heteromyeloma with lymphocytes from a colorectal cancer patient injected with mouse monoclonal antibody 791T/36 for tumour immunoscintigraphy (Austin *et al.* (1989), *Immunology*, **67**, 525). In quantitative flow cytometric assays, the human antibody completely blocked 791T/36 binding to tumour cells. Tests with antibody purified by affinity chromatography indicated a 1:1 stoichiometric interaction between 791T/36 and 105AD7. The complexes formed were stable in the presence of excess antigen positive tumour cells indicating a high affinity interaction between 105AD7 and the binding site of 791T/36. Anti-idiotypic antibodies reactive with the binding site region of anti-tumour antibodies have been shown to induce anti-tumour responses in animals. Therefore, the induction of anti-tumour responses in mice and rats following immunization with 105AD7 was investigated. Mice injected with 105AD7 in Freund's adjuvant developed antibodies reactive with human tumour cell line 791T. Induction of cellular responses was shown by delayed type hypersensitivity reactions in mice and rats, evaluated in coded double blind experiments. Animals were primed by a subcutaneous injection of antibody and a DTH response measured by an increase in ear thickness following tumour challenge. A significant DTH reaction was observed in animals primed with 105AD7 and challenged with 791T tumour compared with those primed with irrelevant human IgG ($P<0.01$). Challenge with tumour cells unreactive with 791T/36 did not induce DTH responses. These data indicate that 105AD7 may be useful as an agent for the stimulation of anti-tumour responses in humans.

**Mapping of monoclonal antibody-defined epitopes on the repeat peptide of polymorphic epithelial mucins**

M.R. Price[1], S. Briggs[1] & S.J. Tendler[2]

*[1]Cancer Research Campaign Laboratories; and [2]Department of Pharmaceutical Sciences, University of Nottingham, Nottingham NG7 2RD, UK.*

Many monoclonal antibodies against human breast carcinomas or milk fat globule membranes react with the protein core of highly glycosylated, polymorphic epithelial mucins (PEM) and the present investigation was designed to identify those structures in the mucin core which are recognized by these antibodies. The protein core of epithelial mucins consists of a repeated sequence of 20 amino acids (Gendler *et al.* (1988), *J. Biol. Chem.*, **263**, 12820). Initially, a series of synthetic heptapeptides were prepared, each of which overlapped by six amino acids, and several anti-PEM antibodies were tested for their reactivity with these peptides. In this way, the minimum linear sequence of amino acids required for antibody binding was identified for each antibody. All epitopes were found to reside in a discrete hydrophilic domain comprising seven amino acids (P D T R P A P) and each antibody required an epitope with as few as three, and up to five, amino acids in order to bind. Further synthetic studies were conducted in which individual amino acid residues within an epitope sequence were replaced by other amino acids and the resulting peptides were examined for antibody binding. Using these synthetic approaches, it is feasible to define at the molecular level the requirements for epitope recognition by antibodies and to acquire an understanding of the specificity of a series of monoclonal antibodies which are highly reactive against human breast carcinomas.

**Monoclonal antibodies MG7 and MC3 recognize tumour-associated antigens in cholangiocarcinomas**

N. Rothnie[1], H. Su[1], B. Fermor[1], N. Rooney[2], E. Dankwa[2], P. Newcomb, C. Wood[1] & N. Habib[1]

*[1]Department of Surgery, Royal Postgraduate Medical School, London; and [2]Department of Histopathology, Bristol Royal Infirmary, Bristol, UK.*

Radiolabelled monoclonal antibodies (MoAb) may offer a new approach to the treatment of cholangiocarcinoma after biliary decompression. Cross reactivity with normal bile duct epithelium remains a problem. MG7 and MC3 are murine MoAbs (IgG2) which have been raised after immunization with human adenocarcinoma cells. They recognize pancarcinoma antigens. The aim of this study was to assess the immunohistochemical pattern of staining of MG7 and MC3 in cholangiocarcinomas and benign and malignant liver tissue. Formalin-fixed paraffin-embedded, sections of tissue from normal liver (n = 10), cholangiocarcinomas (n = 8), colonic liver metastases (n = 5), hepatocellular carcinomas (n = 5), and benign liver disease (n = 12) were stained with MG7 using the avidin-biotin peroxidase method. Staining was assessed by the number of cells staining and by the cytological distribution. No staining was seen with the negative controls. All of the cholangiocarcinomas were positive with MG7, 75% having over 10% cells staining; 7/8 were positive with MC3, half having over 10% cells staining. Staining of tumour cells was situated in the apical membranes and the superficial mucin. No staining was seen in normal liver tissue with either antibody. One case of viral hepatitis showed slight staining of bile duct epithelium with MC3, all the other cases of benign liver disease were negative. All of the colonic metastases were strongly positive with both antibodies. MG7 and MC3 recognize tumour-associated antigens expressed on cholangiocarcinomas and secondary carcinomas in the liver but have minimal reactivity with bile duct epithelium and normal and benign liver tissue.

### Is C219 specific for P-glycoprotein?

G.C. Wishart[1], J.A. Plumb[1], J.J. Going[2], A.M McNicol[2], C.S. McArdle[3] & S.B. Kaye[1]

[1]*CRC Department of Medical Oncology, Glasgow University; and University Departments of* [2]*Pathology &* [3]*Surgery, Royal Infirmary, Glasgow, UK.*

The monoclonal antibody (MAb) C219 reacts with an epitope on the cytoplasmic face of the transmembrane protein, P-glycoprotein (P-gp). Recent work has suggested that C219 may cross-react with the heavy chain of myosin in skeletal and cardiac muscle. By immunocytochemistry with C219 on frozen sections of primary breast cancers, we have shown positive staining in stromal cells, thought to be myofibroblasts, in 26 of 29 tumours. When the same tumours were incubated with MRK16, a MAb which reacts with an external epitope of P-gp, positive staining was seen in the stromal cells of only 12 of the 29 cancers.

We have now tested both C219 and MRK16 with two other tissues known to contain myosin, skeletal muscle and granulation tissue, by immunocytochemistry. C219 showed marked reactivity with frozen sections of skeletal muscle whereas MRK16 did not. When incubated with frozen sections of granulation tissue both C219 and MRK16, as well as a MAb against myosin, reacted strongly with myofibroblasts. The higher level of staining with C219 in breast cancer stroma may represent a possible cross-reaction with myosin and this is supported by positive staining of skeletal muscle, unlike MRK16. Alternatively C219 may be recognizing a form of P-gp which MRK16 does not recognize. The positive staining with both antibodies in granulation tissue is as yet unexplained. The amount of P-gp detected by immunocytochemistry is extremely dependent on the antibody used and results should be interpreted with caution.

### Photosensitization by diaminoanthraquine

J.A. Hartley[1], N. Khan[1], R.L. Souhami[1], K. Reszka[2], P. Bilski[2], C. Chignell[2], A. Mendonca[3] & J.W. Lown[3]

[1]*Department of Oncology, University College & Middlesex School of Medicine, London, UK;* [2]*Laboratory of Mol. Biophys. NIEHS, Research Triangle Park, NC, USA; and* [3]*Department of Chemistry, University Alberta, Edmonton, Alta, Canada.*

1,4-diamino substituted anthraquinone antitumour agents such as mitoxantrone and ametantrone do not show visible light photosensitization whereas the structurally related 1,5- and 1,8-diaminosubstituted compounds (AM1 and AM2) photosensitize human leukaemic cells in culture and show significant light-activated damage in an animal model system. In an attempt to produce simpler and more efficient photosensitizing structures as potential agents for photodynamic therapy, 1-amino, 1, 5- and 1,8-diamino compounds were synthesized (containing 4,5-, 4,8- and 4,5- methoxy groups, respectively). Single oxygen production was found to be very efficient for the 1,5- and 1,8- compounds and superoxide radical generation strong for the 1,8 compound and weak for the others as measured in toluene by luminescence and EPR-spin trapping techniques, respectively. These compounds were found to be advantageous in that they were much less toxic in the dark than AM1 and AM2 with little or no killing of human leukaemic cells at 250 $\mu$M. Only the 1,5-compound however showed significant light-activated killing below 20 $\mu$M. In the case of this agent the light-induced dose modification following a short time of illumination ($<2$ min, $5\ W\ m^{-2} \lambda > 475$ nm) was 15-fold, which could be increased significantly using longer times of illumination. This dose modification was almost 4 times higher than for the 1,5-diamino substituted compound AM1. Attempts to modify the structures further to shift the absorbance maxima to a higher wavelength are currently underway.

### The effect of phthalocyanine mediated photodynamic therapy on tumour blood flow, metabolic activity and cell viability

W-S. Chan[1], N. van Bruggen[2], J.F. Marshall[1], J. Syha[2], E. Proctor[2], A.L. Busza[2], D. Gadian[2] & I.R. Hart[1]

[1]*ICRF Laboratories,* [2]*Department of Physics in Relation to Surgery, Hunterian Institute, Royal College of Surgeons, Lincoln's Inn Fields, London WC2, UK.*

Chloroaluminium sulphonated phthalocyanine (C1A1SPc) is an effective agent in the photodynamic therapy (PDT) of experimental cancer (Chan *et al.* (1988), *Cancer Res.*, **48,** 3048). The present studies were undertaken to determine the mechanisms behind this efficacy. *In vitro* colony forming efficiency (CFE) of neoplastic cells was determined, on single-cell suspensions from solid tumours, at varying times after C1A1SPc-PDT. No substantial reduction in CFE was observed until at least 5 hours after treatment and by 24 hours after PDT the CFE was reduced to 0.1% of control values. In contrast to this relatively slow response to treatment, metabolic activity of the tumour, as assessed by loss of high energy phosphate in $^{31}$P-NMR spectra, was almost completely abolished as early as one hour after PDT. Measurement of regional tumour blood flow, by the technique of $H_2$ washout, revealed a gradual decline to zero 4–6 hours after PDT. These results suggest that while early damage to neoplastic cells may develop as a consequence of C1A1SPc-PDT the observed cell death is a relatively late event probably related to disruption of tumour vasculature.

### The expression of Class I and Class II major histocompatibility complex antigens by renal carcinoma cells in culture

R. Angus[1], C.M.P. Collins[2] & M.O. Symes[1]

[1]*University Departments of Surgery and* [2]*Pathology, Bristol Royal Infirmary, Bristol BS2 8HW, UK.*

Surgical nephrectomy specimens were obtained from 6 patients with renal carcinoma. The solid tumour was enzymatically disaggregated and the carcinoma cells separated from the resulting mixed cell suspension by centrifugation on a Nycodenz$^R$ column. The carcinoma cells were cultured in RPMI1640 with 1% w/v glutamine 10% v/v newborn calf serum and antibiotics added. After varying intervals of culture drops of medium (0.3 ml) containing $10^4$-cells were placed in each well of a 3-well slide. After 24 hours culture, to allow the carcinoma cells to adhere, the medium was replaced by fresh medium alone, or medium containing 100, 500 or 1000 i.u. of α or γ interferon (Wellcome Brolech). Culture was continued for a further 1, 3 or 5 days. The cells in one well on each slide were then stained for expression of Class I MHC by the immunoperoxidase technique using a monoclonal antibody M736 and cells in a second well for expression of Class II MHC (M 704 Dako). The cells in the third well were stained without prior exposure to a MoAb. Of the 6 tumours, 4 expressed Class I MHC Ag when cultured in medium alone for one day. The other 2 tumours expressed class I Ag after culture for 3 days. Class II MAC Ag was expressed by 3 of the 6 tumours on culture in medium alone, and this expression was increased in one tumour and induced in a second by culture in the presence of γ interferon. In one tumour the appearance of Class I Ag was retarded (from 1 to 3 days) by culture in spent medium.

### Expression of MHC molecules on bladder tumour cell line

A.M.E. Nouri, A. Biro, C.V.A. Bunting & R.T.D. Oliver

*Departments of Medical Oncology and Immunology, The London Hospital, London, UK.*

A bladder cell line designate CAT has been developed from a patient with transitional cell carcinoma and has been kept in culture for at least one year. Phenotypic analysis of cell surface antigens using monoclonal antibodies (MoAb) have revealed the loss of MHC class I antigens B7, B44 and Bw6 compared with autologous lymphocytes. These cells were also found to be negative for class II antigens (DR, DP and DQ). Treatment of the cells with 2.5% (v/v) active supernatant (obtained from 48-hour cultured human peripheral blood mononuclear cells activated with PHA) resulted in the upregulation of monomorphic (B2-microglobulin-associated) class I antigen detected by W6/32 MoAb with concomitant decrease in the expression of free heavy chain (none B2 m-associated) of class I detected by HC10 Mab. It was also found that this treatment did not result in the expression of missing polymorphic antigens i.e., B7, B44 and Bw6. Active supernatant at as low as 0.1% (v/v) induced class II antigens after 36 hours of culture with order of antigenic strength of DR > DP > DQ. Preliminary results using dot blotting, locus and allele specific oligo probes indicated that there may be less B7 and B44 mRNA in CAT cells compared with autologous lymphocytes. The results of inducibility of MHC class I and II antigens to different cytokines including IL-1, IL-4, TNF and IFNs individually and in combinations will be presented.

### High metastatic ability of a bladder carcinoma cell line in nude mice

J. Stables[1], P. Topley[1], J. Chowaniec[2], C. Parry[2], L. Holmes[1], D. Linstead[1], D. Jenkins[1], E. Rapson[1] & J. Wilkinson[1]

*[1]Department of Molecular Sciences, Wellcome Research Laboratories, Beckenham, Kent BR3 3BS; and [2]Department of Histopathology, University College & Middlesex School of Medicine, London W1P 7LD, UK.*

The chemically induced rat bladder carcinoma cell line is metastatic in immune suppressed mice (Joy *et al.* (1989), *Br. J. Cancer*, **60**, 493). Studies on the growth of this cell line in nude mice following an inoculation of $1 \times 10^6$ cells subcutaneously indicated that relatively slow-growing tumours were produced which reached 0.6 g in weight after 36 days. These tumours however ulcerated in some animals as early as day 28, necessitating culling, and the experiment was terminated at day 36, at which time macrometastatic foci were detected on 40% of the lungs. Experiments have since been performed in which lung metastases from nude mice inoculated subcutaneously with fragments of the $RUCL_2$ tumour have been selected and passaged subcutaneously in nude mice. By this method it was hoped that variants of $RUCL_2$ would be produced which had either a reduced level of ulceration, or an increased level of metastatic behaviour. Two variants have been produced, $RUCL_2T$ and $RUCL_2T$, which by 45–50 days produce macrometastatic lesions in the lungs of 100% of the inoculated mice and with a level of ulceration at least 50% lower than that produced by $RUCL_2$. The metastases recovered displayed the histological features of $RUCL_2$ line, that is moderate-poorly differentiated transitional cell carcinoma. Further work is now in progress to develop the $RUCL_2$/nude mouse system into a model suitable for the evaluation of potential antimetastic compounds. If successful, this model will be invaluable as, unlike the majority of other models of metastasis using carcinoma cell lines, it is representative of the whole metastatic process.

### Evaluation of cell attachment matrix (CAM) coated plates for primary culture of human tumour biopsies for predictive testing of radiosensitivity

P. Price, C.S. Parkins, C. Bush, M. Robinson & T.J. McMillan

*Radiotherapy Research Unit, Institute of Cancer Research, Sutton, Surrey SM2 5NG, UK.*

Baker *et al.* (1986, *Cancer Research*, **46**, 1263) developed an adhesive tumour cell culture system (ATCCS) using the cell attachment matrix (CAM) (LifeTrac Inc.) to promote attachment and growth of tumour cells from biopsies so that response to irradiation could be measured using a mass culture technique. In the present study, 22 human tumour biopsies were used to test this system: bladder (11 cases), cervix (4 cases), sarcoma (3 cases), head and neck (3 cases), and prostate (1 case). Tumour biopsy weight was mean 3.3 g (range 1–10 g), with a mean cell yield after 30 min digestion of $2.5 \times 10^6$ (range $0.05–20 \times 10^6$). Successful growth was obtained in 9/20 samples (45%). In all 9 cases, growth at 1 and 7 days showed mainly fibroblasts and a few tumour cells. By day 14, in 5 cases the morphological appearance of the cells was that of fibroblasts, while in 2 cervical carcinomas and 2 sarcomas, the cells were of indeterminate type. Response to irradiation was measured using either clonogenic assay or a mass culture technique (MTT assay) in 4 cases. SF2 values obtained were 0.25, 0.28, 0.28 and 0.64. The survival curves from the cells with the low SF2 values were almost straight, thus confirming the morphological suspicion that the cells were most likely fibroblasts. In our hands, the ATCCS would not appear successful in preferentially growing tumour cells and preventing the overgrowth of fibroblasts. Comparison with other surfaces showed that CAM was no better for establishing tumour cell growth than the presence of feeder cells or an alternative attachment factor vitronectin, and was less good than conventional soft agar. The use of a mass culture technique would also seem inappropriate in assessing response to survival when good exponential growth of tumour cells was never obtained.

### Neuroblastoma: a study of radiosensitive human tumour cells

T.J. McMillan, J. Eady, A. Holmes, J. Peacock & G.G. Steel

*Radiotherapy Research Unit, Institute of Cancer Research, Sutton, Surrey SM2 5NG, UK.*

Neuroblastomas are radioresponsive tumours and this is reflected by the high sensitivity to ionizing radiation of cell lines derived from these tumours. Each of the two cell lines examined in this study, HX142 and HX138, demonstrates a sensitivity to ionizing radiation which is comparable to that of transformed Ataxia telangiectasia (A.T.) fibroblasts. These cells do not exhibit a reduced level of cellular recovery in split-dose or low dose-rate experiments unlike A.T. cells. In addition they show an inhibition of DNA synthesis following irradiation which is comparable to resistant normal cells rather than A.T. fibroblasts. Repair capacity has been assessed using the viral reactivation assay. The level of reactivation for the two neuroblastoma cell lines was found to be in the middle of the range seen for resistant tumour cell lines. This suggests that they do not have a significant repair defect. Both lines show a higher than average level of DNA double-strand breaks immediately after irradiation, however the extent of this is not sufficient to explain fully the sensitivity of these cells. Therefore, it seems that the sensitivity may be related to a high level of damage induced in the DNA by the radiation but that some as yet undetermined processes increase the conversion of this damage into lethal events.

**XH1 – a new cervical carcinoma cell line: a xenograft model of invasion and metastasis**

X. Han, P.J. Warren, R. Lyle & E. Heyderman

*Department of Histopathology, UMDS, St Thomas Hospital, London SE1 7EH, UK.*

A new cervical carcinoma cell line, XH1, has been derived from a moderately differentiated adenosquamous invasive carcinoma of the cervix in a woman of 32. It has been maintained in long term monolayer culture for one year and for 56 passages. In culture, it has the morphology of adherent polygonal epithelial cells. It is aneuploid, with mean chromosome number of 78 (range 60–84). Probing with the MS1 and MS31 hypervariable minisatellite probes has shown its DNA fingerprint to be different from that of other epithelial tumour cell lines maintained in this laboratory – Caski, A431 and Bowes melanoma. A series of subcutaneous and intraperitoneal xenografts in nude mice show a moderately differentiated adenosquamous carcinoma very similar to the original tumour. At EM level, desmosomes and tonofilaments were demonstrated. The subcutaneous xenografts showed local invasion into host muscle and blood vessels, as well as perineural invasion. In an initial group of nude mice, $1-5 \times 10^4$ XH1 cells were injected intravenously into the tail vein of 8-week-old nude mice. Two animals which died soon after injection were shown to have tumour cells in the right ventricle and in small blood vessels in the lungs. No tumours were seen in the rest up to 76 days after intravenous injection. In a more recent experiment, $5 \times 10^4$ cells were injected, and in one of the 6-week-old mice small nodules of tumour were seen in the lung parenchyma 23 days after injection. Elsewhere, small organized and endothelialized nodules or tiny calcified bodies were seen within pulmonary vessels. These are interpreted as the sites of lodgement of tumour cells which did not survive, or failed to become established. We have also raised monoclonal antibodies to whole XH1 cells in BALB/c mice using the P3X63Ag8.653 mouse myeloma cell line. Two of them, TDM35 and TDM39, have been cloned. These antibodies stain formalin-fixed paraffin-embedded tissue sections using an ABC immunoperoxidase method. They show a marked quantitative difference in staining intensity between normal and dysplastic or frankly malignant cervical epithelium. The antibodies are being evaluated on a wide variety of normal and neoplastic tissues.

**The derivation and characterization of a gastrin-producing adenocarcinoma cell line and its response to the anti-secretory agent, SMS-201-995**

S.A. Watson[1], A.R. Hall[1], L.G. Durrant[1] & D.L. Morris[2]

*[1]Cancer Research Campaign Laboratories, University of Nottingham NG7 2RD; and [2]Department of Surgery, University Hospital, Nottingham NG7 2UH, UK.*

MKN45, a human gastric adenocarcinoma cell line is gastrin-17 (G17) responsive both *in vitro* and *in vivo*. MKN45 xenografts which were trophically stimulated by G17 were selected to develop a highly G17-sensitive xenograft line. Upon successive passages, the selected line became unresponsive to G17, yet its basal growth rate had doubled. This line was denoted MKN45G. Examination by immunohistology and flow cytometry revealed that MKN45G had intracellular gastrin-like immunoreactivity (as detected with a rabbit anti-G17 polyclonal) in 70 to 80% of cells which could be eliminated by pre-absorbing the polyclonal with G17. This was not detected in the parental line. Further analysis showed that the 2 cell lines were 98% epithelial in origin (detection with an anti-cytokeratin) and both were positive for PGP 9.5, a polyclonal which detects neuroendocrine function. MKN45G was highly positive for CEA (80–90% of cells positive) unlike MKN45 ($<5\%$ cells positive). With respect to receptor expression (as measured by binding of radioligands), both cell lines were positive for gastrin receptors (MKN45:$3 \times 10^3$, MKN45G:$2 \times 10^3$/cell). However MKN45G expressed more insulin-like growth factor I receptors than MKN45 (MKN45: $3 \times 10^2$, MKN45G:$2.6 \times 10^3$/cell). Both cell lines expressed SMS-201-995 binding sites. However, *in vivo* therapy of MKN45G and MKN45 xenografts with pumped SMS-201-995 ($25 \mu g\, kg^{-1}$ daily) resulted in reduced tumour growth with the former xenografts ($P<0.001$ after 13 days therapy) and no effect on the latter xenografts. We have now identified gastric and colorectal tumour cells from primary tumours with intracellular gastrin-like immunoreactivity therefore somatostatin-like analogues may have therapeutic potential.

**Characteristics of newly developed tumour cell line**

A.M.E. Nouri[1], A. Bergbaum[3], E. Lederer[2] & R.T.D. Oliver[1]

*Departments of [1]Medical Oncology & [2]Immunology, London Hospital; and [3]Department of Cytogenics, Guy's Hospital, London, UK.*

A tumour cell line designated CAT derived from a patient with invasive (PT2aG3) transitional cell carcinoma (TCC) of bladder has been in culture for more than 18 months and been sub-cultured more than 100 times. The pattern of proliferation (doubling time about 36 hours) of these cells indicates their metabolically active nature. Epithelial origin of the cell line was established using whole range of monoclonal antibodies (Mab) and electromicroscopic examination. In parallel, 'Tumour Infiltrating Lymphocytes' (TIL) were isolated from the original tumour tissue and were expanded in conditioned medium plus interleukin-2, expressing all the phenotypic characteristics of normal activated T cells. Biochemical analysis of cell surface molecules of the TIL and CAT cells revealed the loss of HLA-B44, B7 and Bw6 from the CAT cells whilst maintaining other class I antigens including HLA-Bw4, A2 and A3. No such losses were observed on TIL. Karyotypic analysis of TIL and CAT cell revealed the presence of three rearranged chromosomes (4, 11 and 13) only in the case of CAT cells. Oncogene activation studies showed no H-*ras*, Ki-*ras* or N-*ras* activation in TIL or CAT cells. These results are indicative of the presence of activated T cells at tumour site and are consistent with the suggestion that they might have become activated in response to putative neo-antigen(s) expressed on the tumour cells. The failure of these activated T cells to inhibit tumour progression however, may be due to the loss of class I antigens from the tumour cells rendering them resistant to killing by cytolytic T cells.

### Inhibition of chemically-induced neoplastic transformation *in vitro* by saturated fatty acids

M.J. Embleton

*Cancer Research Campaign Laboratories, University of Nottingham, University Park, Nottingham NG7 2RD, UK.*

Exogenous fatty acids can modify tumour cell growth *in vitro* and *in vivo*, and it has been reported that stearic acid and its analogues can suppress chemical carcinogenesis *in vivo* (Habib *et al.* (1987), *Br. J. Cancer*, **56**, 455). In an attempt to determine whether this suppressive effect operates preferentially at particular stages of a multistep process, the effect of stearic acid (SA) analogues was tested at multiple points in an *in vitro* transformation system using Syrian hamster embryo cells. Primary cultures of 12-day embryo fibroblasts were treated for 2–4 days with 0.5 $\mu g\,ml^{-1}$ 3-methylcholanthrene (MCA), and 28 days after application of the carcinogen they were exposed continuously to 0.1 $\mu g\,ml^{-1}$ 12-O-tetradecanoylphorbol 13-acetate (TPA). Untreated cells, or cells treated with MCA or TPA alone, generally became sensecent around 6–8 weeks after plating and died, but cultures treated with both MCA and TPA developed foci of immortalized cells which progressed to yield classically transformed cells capable of growth in soft agar. Methoxy- and keto- derivatives of SA inhibited immortalization and transformation at concentrations of 50 and 100 $\mu$M, and iodo-SA was inhibitory at 100 and 200 $\mu$M. The compounds were equally active when applied before, during or after MCA, and before or during TPA treatment. They were thus not selective for phases involving cellular interaction with MCA (initiation) or TPA (promotion). Their action appears to be associated with control of growth or differentiation in a relatively non-specific manner.

### Inhibition of growth of carcinoma cells by inhibitors of 5-lipoxygenase

A. Richter[1], C. Robinson[2], D. Davies[1] & P. Alexander[1]

*Departments of [1]Medical Oncology and [2]Clinical Pharmacology, The University of Southampton, Southampton General Hospital, SO9 4XY, UK.*

Concomitant with the stimulation of DNA synthesis by polypeptide growth factors, release of arachidonic acid and its metabolites to prostaglandins and leukotrienes, has been observed. Products of 5-lipoxygenase (5LPO), such as LTB4 as well as other leukotrienes were found by Palmberg *et al.* (1987, *J. Cell Sci.*, **88**, 151), to stimulate the initiation of DNA synthesis in cultured smooth muscle cells at pM concentration. We examined the antiproliferative activities of inhibitors of 5LPO in two systems. Contact inhibited human foreskin fibroblasts (HFF1), which had undergone only limited passage, increased incorporation of thymidine (or 125 IUdR used for experimental convenience) by more than 20-fold on exposure to EGF. The 5LPO inhibitors, U-66,858 and AA861 inhibited initiation of DNA synthesis in a dose dependent manner (50% inhibition at approximately $10^{-5}$ M). An antagonist (Ly 171,883) of LTD4 and LTE4 was inactive, as was the powerful and selective cyclooxygenase inhibitor, flurbiprofen. The same pattern of activity was also seen in a proliferation assay in which cells are grown in medium with 10% serum. The inhibition of proliferation of HFF1 cells was compared with three carcinoma cell lines, HT29, A431 and HN5, which express EGF receptors and produce TGFα. Growth of the carcinoma cells was arrested by both of the 5LPO inhibitors with a $LD_{50}$ of about $3 \times 10^{-5}$ M, whereas HFF1 was significantly more resistant. Flurbiprofen and the LTD4 and LTE4 antagonist were much less active. None of the compounds studied affected EGF receptors. We suggest that certain of the leukotrienes formed in response to stimulation of EGF receptors contribute to the mitogenic action of EGF and TGFα. A role for LTB4 in tumour growth was suggested by El-Hakin *et al.* (1989, *Br. J. Cancer*, **59**, 833) who found the concentration of LTB4 in human squamous cell carcinomas to be 10 times higher than in unaffected surrounding tissues.

### Utility of primary cells transformed by collaborating oncogenes for *in vitro* antitumour drug assay

E. Rapson, D. Linstead, R. Skinner, L. Holmes, J. Stables, P. Topley, D. Jenkins & J. Wilkinson

*Department of Molecular Sciences, Wellcome Research Laboratories, Beckenham, Kent BR3 3BS, UK.*

There is considerable interest in developing new anticancer agents which would be more specific for transformed cells in their action, and thus sparing of normal tissue. Problems arise in the choice of *in vitro* assays for the search for such agents. Ideally such assays should employ cell lines transformed by well defined processes against which new more specific drugs can be directed, as a preliminary to validating these processes as targets in naturally occuring tumours. An approach to the creation of suitable cell lines is the transformation of non-malignant cells by transfection of known oncogenes. We have generated a number of transformed cell lines by transfection of the *ras* oncogene, and the E7 gene from human papilloma virus type 16 together into baby rat kidney primary cells using both constitutive and inducible expression vectors. We report on the characterization and dependence upon oncogene expression of these cells and their possible use for drug assay.

### Interactions of adriamycin with human erythrocyte inositol-lipid metabolism

M.G. Thompson & J.A. Hickman

*Cancer Research Campaign Experimental Chemotherapy Group, Pharmaceutical Sciences Institute, Aston University, Birmingham B4 7ET, UK.*

The aminoglycoside antibiotic adriamycin is a potent antitumour drug in humans and there is evidence to suggest that it may exert its effects via the plasma membrane. We have previously shown that, at relatively high concentrations, adriamycin ($10^{-4}$–$10^{-3}$ M) inhibits inositol-lipid metabolism in human erythrocytes and this may contribute to its antiproliferative effects (1987, *Cancer Res.*, **47**, 2797). However, at low concentrations, adriamycin ($10^{-9}$ M) has been shown to stimulate cell growth (1989, *Cancer Res.*, **49**, 2679), suggesting that it may stimulate inositol-lipid metabolism. We have recently found that similar concentrations of adriamycin elevate inositol 1,4,5-trisphosphate levels in human erythrocyte vesicles. However, there was no significant decrease in phosphatidylinositol 4,5-bis-phosphate levels in response to adriamycin suggesting that stimulated breakdown of this lipid may not be the reason for elevated levels of inositol 1,4,5-trisphosphate. In addition, the human erythrocyte contains a 5′-phosphomonoesterase which degrades inositol 1,4,5-trisphosphate to inositol 1,4-bisphosphate. A time-course of inositol 1,4,5-trisphosphate dephosphorylation showed that the effect of the 5′-phosphomonoesterase was decreased in the presence of adriamycin. This suggests that adriamycin may produce elevated levels of inositol 1,4,5-trisphosphate in human erythrocyte vesicles by inhibiting inositol 1,4,5-trisphosphate breakdown rather than by stimulating phosphatidylinositol 4,5-biophosphate breakdown.

### An investigation of the mechanism by which the ether lipid SRI 62-834 raises intracellular calcium concentration in HL-60 leukaemia cells

C.M Lazenby, C. Dive, M.G. Thompson & J.A. Hickman

*Cancer Research Campaign Experimental Chemotherapy Group, Pharmaceutical Sciences Institute, Aston University, Birmingham B4 7ET, UK.*

We have previously shown that the membrane active antineoplastic ether lipid SRI 62-834 produces a concentration-dependent rise in cytosolic free $Ca^{2+}$ $[Ca^{2+}]i$ in Quin-2 loaded HL-60 human promyelocytic leukaemic cells (Thompson & Hickman (1988), *Biochem. Soc. Trans.*, **16**, 278). The aim of this study was to define the nature of this rise in $[Ca^{2+}]i$. SRI 62-834 induced a $[Ca^{2+}]i$ rise in Indo-1 loaded HL-60 cells which was biphasic as opposed to that produced by triton (0.01%) which was monophasic and immediate. A purely detergent effect is therefore unlikely to be involved. Inclusion of $Mn^{2+}$ (known to quench the fluorescence of Indo-1) in the incubation buffer resulted in the quenching of the second phase of the SRI 62-834 induced $[Ca^{2+}]i$ response. This indicated that this component of the response was due to the influx of extracellular $Ca^{2+}$. We have also compared the effects of SRI 62-834 on HL-60 $[Ca^{2+}]i$ with that produced by the naturally occurring and chemically similar ether lipid platelet activating factor (PAF). PAF like SRI 62-834 produced a rise in $[Ca^{2+}]i$. Although the HL-60 response to SRI 62-834 involves $Ca^{2+}$ movement across the plasma membrane, it does not appear, however, to be mediated via a high affinity PAF receptor as PAF antagonists [500 μM] failed to inhibit the response.

### Expression of the c-erbB-2 (neu) oncogene in bladder cancer

K. Mellon[1], D.E. Neal[1], C. Wright[2], P. Johnston[2], I.P. Corbett[2] & C.H.W. Horne[2]

*[1]Urology Department, Freeman Hospital, Newcastle upon Tyne NE7 7DN and [2]University Department of Pathology, University of Newcastle upon Tyne, UK.*

The proto-oncogene c-erbB-2(neu) encodes a transmembrane glycoprotein which is similar to the epidermal growth factor receptor (EGFr). Amplification of the c-erbB-2 gene and overexpression of its protein product have been reported mainly in adenocarcinomas arising in various sites and in breast cancer are associated with a poor prognosis. We have studied expression of the c-erbB-2 oncoprotein in bladder cancer. Frozen sections from 45 primary transitional cell carcinomas of the bladder (29M, 16F; age = 70 + / − 10 yrs; pTa + T1 = 24, pT2 + pT3 + pT4 = 21; 25 = Grade 2 and 20 = Grade 3) were stained immunohistochemically using a monoclonal antibody (CB-11) against a synthetic peptide representing residues close to the C-terminus of the predicted oncoprotein sequence. Tumours were scored for both staining intensity [0, weak ( + ) or strong ( + + )] and proportion of tumour cells staining. Seventeen (38%) tumours showed evidence of positive staining; 5 tumours showed areas of strong positive staining (4 muscle invasive tumours and 1 pTaG2 which rapidly progressed). Weak positive staining was noted in a further 4 muscle invasive tumours and in 8 superficial tumours. The c-erbB-2 oncoprotein has been demonstrated in a significant proportion of bladder cancers. The relationship between its expression, EGFr status and other risk factors may provide important prognostic information.

### Analysis of phorbol ester (PE) binding to protein kinase C (PKC) using the novel fluorophore bodipy-3-PE and multi-parameter flow cytometry (FCM)

T.D. Bradshaw, C. Dive & A. Gescher

*Cancer Research Campaign Experimental Chemotherapy Laboratory, Pharmaceutical Sciences Institute, Aston University, Birmingham B4 7ET, UK.*

PKC plays a critical role in cell signalling pathways. We describe a new method to measure PKC levels in individual viable cells using a fluorescently tagged PE and FCM. A549 human lung carcinoma cells ($10^6$ $ml^{-1}$) were incubated for 10 minutes with 10 nM Bodipy-3-PE ± 5 μM unlabelled PE to determine non-specific binding. Propidium iodide (32 μM) was added immediately before analysis allowing exclusion of non-viable cells. Ten thousand cells were analysed per sample with respect to 90° light scatter (a measure of cell size), red fluorescence (propidium > 630 nM) and green fluorescence (Bodipy-3-PE, 515–560 nM) using a FACS 440 instrument with argon laser excitation at 488 nM. In competitive binding studies, cells were co-incubated with increasing concentrations of 12-O-tetradecanoylphorbol-13-acetate (TPA). Dose-dependent reduction in green fluorescence of 44%–13% was observed compared to control values with 0.1 nM–100 nM TPA respectively. In cells incubated overnight with 10 nM TPA, an 83% decrease in Bodipy-3-PE binding was seen suggestive of PKC downregulation. Moreover, in cells cultivated in the continuous presence of TPA (10 nM), negligible specific Bodipy-3-PE binding was detected. These results agree with those of radiochemical methods of PKC assay. This new assay is rapid, sensitive and will reveal heterogeneity in PKC levels within mixed cell populations. In addition, it can be combined simultaneously with the assay of other pertinent cellular parameters such as $Ca^{2+}$ flux. We are currently using this assay to explore the competitive nature of bryostatin binding to the PE receptors.

### An automated system for the determination of nucleolar organizer regions in breast carcinoma

D.J. Hehir, A.M. Seifalian & S.P. Parbhoo

*Academic Department of Surgery, Royal Free Hospital School of Medicine, London NW3 2QG.*

Nucleolar organizer regions (Ag-NOR's) are loops of DNA with ribosomal genes (Fakan & Hernandez-Verdun (1986), *Bio. Cell*, **56**, 189). They are useful markers of cellular proliferative activity, and have been used to indicate prognosis (Ploton *et al.*, (1986), *Histochem. J.*, **18**, 5). Ag-NORs are difficult to quantify, however, owing to subjective bias, prolonged counting time, and inter-observer variation. To overcome this problem, we have used a computerized system to measure Ag-NORs in archival tissues of patients with breast carcinomas. Eleven silver-stained histology slides were digitized using a video camera interfaced to a microcomputer, and for each digitized image 3 different area sizes were selected. Images were transferred to a Sun workstation, processed automatically, and also counted manually. The algorithm for quantifying Ag-NORs is based on histograms of grey values of preselected regions of interest. Quantitative information of Ag-NOR counts was determined from the histograms by fitting with a Gaussian function. A correlation coefficient of 0.904 was determined for the two methods, and the standard error of the differences in the two methods was 1.65. This computerized system is useful in determining Ag-NORs, and therefore may assist in prognosis and therapy of patients with breast cancer.

### Gene expression in oestrogen-dependent human breast cancer xenograft tumours

A.M. Thompson[1,2], C.M. Steel[2], M.E. Foster[2], D.J. Kerr[3], R.A. Hawkins[1] & W.R. Miller[1]

*[1]Department of Surgery, Royal Infirmary, Edinburgh EH3 9YW; [2]MRC Human Genetics Unit, Western General Hospital, Edinburgh CH4 2XU; and [3]Beatson Institute, Garscube Estate, Switchback Road, Glasgow G61 1BD, UK.*

Xenograft tumours have been established from the oestrogen-dependent MCF-7 breast cancer cell line in thymectomized, irradiated female CBA mice. With serial transplantation there was evidence of increased tumorigenicity of transplanted material ($P<0.04$, Fisher's exact test), and an altered pattern of gene expression compared to the original cells. However, oestrogen-dependence was retained. Following injection of the mice with oestrogen, there was an increase in DNA synthesis and mitosis was stimulated with consequent increase in tumour growth. Laser densitometry was used to quantify mRNA on Northern blots and showed that with oestrogen stimulation, c-*myc*, p53 and pS2 gene expression was increased whereas TGF-beta expression was suppressed. This xenograft system demonstrates changes in gene transcription in response to oestrogen. It thus provides an *in vivo* model for molecular and biochemical studies of hormone-sensitive human breast cancer.

### Growth factor and epidermal growth factor receptor expression in gastric carcinoma

I.M. Paterson[1], Y, Luqmani[2], C. Bennett[2] & C.M. Corbishley[3]

*Departments of [1]Surgery, [2]Oncology and [3]Histopathology, St George's Hospital, London SW17 0RE, UK.*

Polypeptide growth factors (GF) may be important in the development of some tumours but it has not been established whether they might have a role in the pathogenesis of gastric carcinoma. The expression of genes encoding Transforming Growth Factors (TGF) alpha and beta, Platelet Derived Growth Factors A and B, Insulin-like Growth Factor II and Epidermal Growth Factor Receptor (EGFR) was examined by RNA hybridization in benign and malignant gastric tissue from 22 patients. All the GFs and EGFR were produced in increased amounts by the gastric carcinoma compared to benign gastric epithelium ($P<0.05$). The transcripts were widely expressed and at least 2 were present in every sample analyzed. Co-expression of TGF alpha and EGFR was found in 15 malignant but only 6 benign specimens raising the possibility of autocrine stimulation in the neoplasms. No correlation was found between GF expression and tumour stage or patient survival. GFs may mediate various inflammatory processes but no evidence of increased production was found in 8 specimens demonstrating superficial gastritis and characterized by a marked inflammatory infiltrate. The finding of increased GF production by gastric carcinomas may have therapeutic implications.

### The influence of calcium supplementation on experimental ileo-colonic hyperplasia and carcinogen-induced neoplasia in the rat

G. Barsoum, M. Winslet, J.P. Neotolemos & M.R.B. Keighley

*Academic Department of Surgery, Queen Elizabeth Hospital, Birmingham, UK.*

Oral calcium supplementation may reduce the colonic proliferative index in patients with colorectal cancer. This study assessed the influence of dietary calcium supplementation ($25\,g\,Ca$ lactate $l^{-1}$ $24\,h^{-1}$) compared to normal chow on cellular proliferation and tumour formation in Wistar rats after 75% small bowel resection and dimethylhydrazine (DMH $40\,mg\,kg^{-1}$ for 5 weeks) administration compared with controls. Cellular proliferation was assessed by an *in vivo* stathmokinetic method. SBR ($P<0.01$ ANOVA: repeated measures), DMH ($P<0.01$) and SBR + DMH ($P<0.01$) produced a significant increase in cellular proliferation. Calcium supplementation had no influence on normal cellular proliferation but produced a significant reduction in ileal ($P<0.02$), caecal ($P<0.01$) and colonic proliferation ($P<0.01$) after SBR, DMH and SBR + DMH. Calcium supplements also produced a significant reduction in tumour ($P<0.05$) yield in the DMH group but not in the DMH + SBR group. This study supports the concept that calcium supplementation reduces colonic cellular proliferation, but may not reduce the incidence of neoplasia. As the effect is similar after both SBR and DMH administration, the mechanism is likely to be due to both binding of cancer promoting agents and inhibition of tumour initiation by a direct action on the cell membrane.

| | *Control 30* | | *DMH 30* | | *SBR 30* | | *SBR + DMH* | |
|---|---|---|---|---|---|---|---|---|
| | *°Ca* | *+ Ca* | *°Ca* | *+ Ca* | *°Ca* | *+ Ca* | *°Ca* | *+ Ca* |
| Ileum | 13 | 12 | 35 | 17 | 27 | 21 | 35 | 21 |
| Caecum | 5 | 7 | 13 | 4 | 11 | 7 | 15 | 7 |
| Colon | 6 | 3 | 17 | 5 | 19 | 8 | 16 | 7 |
| Colon tumours | 0 | 0 | 13 | 5 | 0 | 0 | 9 | 14 |

### The influence of small bowel resection on hydrazine-induced carcinogenesis in an animal model of colorectal cancer

G. Barsoum, M. Winslet, A. Kappas & M.R.B. Keighley

*Academic Department of Surgery, Queen Elizabeth Hospital, Birmingham, UK.*

Subcutaneous dimethylhydrazine (DMH) administration in the rat is an established method of producing experimental colonic carcinoma. Colonic hyperplasia after small bowel resection may promote such carcinogenesis. This study assessed the influence of 75% mid small bowel resection and DMH administration ($40\,mg\,kg^{-1}$ for 5 weeks) for 10 and 30 weeks on ileal, caecal and colonic cellular proliferation and macroscopic tumour formation in the Wistar rat, compared with controls. The crypt cell production rate (CCPR) (cells/crypt/h) was assessed by an *in vivo* stathmokinetic method. The CCPR at the 3 different sites was significantly different at 10 and 30 weeks ($P<0.01$ ANOVA: repeated measures). The influence of DMH on CCPR was significant in all 3 areas only at 30 weeks ($P<0.01$). SBR produced a significant increase in the ileum and colon at 10 weeks ($P<0.01$) and all 3 areas at 30 weeks ($P<0.01$). Simultaneous SBR failed to increase cellular proliferation or macroscopic tumour yield in the DMH model. DMH probably produces neoplasia by a direct and systemic action. SBR produces hyperplasia by luminal and hormonal mechanism. As SBR + DMH failed to increase tumour yeild it suggests that hyperplasia does not increase susceptibility to malignant change by solely increasing the population of at risk malignant cells or rapidly perpetuating any mutational change.

| *CCPR Result* | *Control 10 (n = 8)* | *DMH 10 (n = 8)* | *SBR1 (n = 8)* | *Control 30 (n = 6)* | *DMH 30 (n = 6)* | *SBR 30 (n = 6)* | *DMH + SBR 30 (n = 6)* |
|---|---|---|---|---|---|---|---|
| Ileum | 35 | 45 | 66 | 13 | 35 | 27 | 35 |
| Caecum | 19 | 21 | 21 | 5 | 13 | 11 | 15 |
| Colon | 9 | 9 | 15 | 6 | 17 | 19 | 16 |
| Colon tumours | | 0 | | | 12 | | 9 |

### The relationship between ileo-colonic cellular proliferation and tumour formation in an animal model of colorectal cancer

G. Barsoum, M. Winslet, D. Youngs & M.R.B. Keighley

*Academic Department of Surgery, The Queen Elizabeth, Birmingham, UK.*

Dimethylhydrazine (DMH) administration in the rat results in an increase in colorectal cellular proliferation and macroscopic tumour formation. The influence of such a stimulus on simultaneous small bowel and caecal cell turnover is poorly documented. This study assessed the effect of DMH administration (40 mg $kg^{-1}$ body weight per week) for 5 weeks on simultaneous ileal, caecal and rectal cellular proliferation and macroscopic tumour formation at 15 (DMH15 = 22) and 30 weeks (DMH30 = 22) in the Wistar rat, compared with controls (C). CCPR (cells/crypt/h ± SEM) was assessed by an *in vivo* stathmokinetic method with vincristine-induced metaphase arrest.

| | *ILEUM* | | *CAECUM* | | *(Left) COLON* | |
|---|---|---|---|---|---|---|
| *Weeks* | *CCPR* | *Tumour* | *CCPR* | *Tumour* | *CCPR* | *Tumour* |
| C15 | $20.9 \pm 1.5^{a}$ | 0 | $11.2 \pm 6.4^{b}$ | 0 | $5.2 \pm 0.4^{c}$ | 0 |
| DMH15 | $25.9 \pm 2.9$ | 0 | $12.4 \pm 0.7$ | 0 | $6.7 \pm 0.7$ | 0 |
| C30 | $9.1 \pm 0.6^{a,d}$ | 0 | $4.7 \pm 0.5^{b,e}$ | 0 | $3.9 \pm 0.6^{c,f}$ | 0 |
| DMH30 | $16.6 \pm 1.8^{a,d}$ | 0 | $9.6 \pm 1.6^{b,e}$ | 0 | $9.3 \pm 1.1^{c,f}$ | $13^{g}$ |

a,b,c,d,e,f: $P = 0.05$ ANOVA with linear regression in groups.
g: $P < 0.05$ $\chi^2$, compared to ileum and caecum.

This study confirms that CCPR decreased with age. DMH administration produced an increase in CCPR at 30 weeks only, in both ileal and colonic mucosa. Despite the fact that the caecum contains large bowel mucosa, with carcinogenesis it acts like small bowel. As in the human condition, tumour formation predominantly occurs in the left colon. This suggests that tumour formation in the rat model is dependent on luminal factors as well as DMH-induced cellular hyper-proliferation.

### EGF and TGF-β act as markers of tumour progression in experimental oral carcinogenesis

S.M. Game, A. Stone, C. Scully & S.S. Prime

*University Department of Oral Medicine, Surgery and Pathology, Bristol Dental Hospital, Bristol BS1 2IY, UK.*

This study examined the expression of EGF and TGF-β cell surface receptors, and the growth response to these factors, in oral keratinocyte cell lines that reflected tumour progression in the rat-4NQO model of oral carcinogenesis. Tumour progression was expressed in terms of cellular immortality, anchorage independence and tumorigenicity. The expression of cell surface receptors was measured in radio-ligand binding assays and the response to growth factors by tritiated thymidine incorporation studies. Keratinocytes from 4NQO-treated tissues had predominantly fewer EGF and TGF-β receptors than normal controls. Whilst the expression of TGF-β receptors was unrelated to anchorage independence and tumorigenicity, the expression of high affinity EGF receptors paralleled the emergence of both the anchorage independent and tumorigenic phenotype. Normal keratinocytes were stimulated and inhibited, in a dose-dependent manner, by EGF and TGF-β respectively. By contrast, studies with immortal/anchorage dependent/non-tumorigenic and immortal/anchorage independent/tumorigenic cells demonstrated a progressive independence of growth factor control. The results suggest that the responses to EGF and TGF-β, and the expression of cell surface receptors, may be useful markers of the premalignant epithelial phenotype in the rat-4NQO model of oral carcinogenesis.

### An *in vitro* model of tumour progression in human colorectal carcinogenesis

A.C. Williams, S. Harper & C. Paraskeva

*Department of Pathology, The Medical School, University of Bristol, University Walk, Bristol BS8 1TD, UK.*

To study tumour progression in human colorectal carcinogenesis, the premalignant human colonic PC/AA adenoma cell line (Paraskeva *et al.* (1984), *Int. J. Cancer*) was transformed in multiple steps towards the malignant phenotype. A rare clonogenic variant of PC/AA was isolated (AA/C1) which, when treated for 14 days with 1 mM of the differentiating agent sodium butyrate, gave rise to a cell line with a greater than four-fold increase in colony forming efficiency (CFE). This anchorage dependent cell line was designated AA/C1/SB. On exposure to the carcinogen N-Methyl-N-Nitro-Nitrosoguanidine (MNG) these cells became anchorage independent. The resulting cell line was designated AA/C1/SB10. AA/C1 cells treated with the carcinogen alone did not become anchorage independent. On continuous *in vitro* passage, the CFE in agarose of AA/C1/SB10 has so far increased from 0.16% to 17.3% and the cells have become tumorigenic, producing moderate to well differentiated adenocarcinomas in nude mice. AA/C1, AA/C1/SB and AA/C1/SB10 cell lines have common chromosomal abnormalities which include a deleted chromosome 1p, and possible abnormalities in chromosomes 17 and/or 18. This *in vitro* progression provides the first reported experimental evidence for the adenoma to carcinoma sequence in human colorectal epithelial cells. Experiments are in progress to investigate allele loss using polymorphic probes to chromosomes implicated in colonic carcinogenesis. The molecular characterization of cells with different malignant potentials may enable the isolation of tumour suppressor gene(s).

### Expression of PS2 in human stomach

Y.A. Luqmani[1], C. Bennett[1], I. Paterson[1], C. Corbishley[1], M.C. Rio[2], P. Chambon[2] & G. Ryall[1]

*[1]Medical Oncology, St George's Hospital Medical School, London SW17 0RE, UK; and [2]LGME/CNRS and U.184/INSERM, Faculte de Medicine, 67085 Strasbourg, France.*

The oestrogen-induced breast cancer related pS2 has been found to be synthesized and secreted from human stomach (Rio *et al.* (1988), *Science*, **241**, 705). We examined its expression in 75 samples of normal, hyperplastic and malignant resections, and noted marked differences. Varying transcript levels were seen in the non-neoplastic groups, with very high levels in 79% of tissues with gastritis. Only 43% of tumours were + ve, and high levels seen in only 18%; there was no correlation to age grading, stage or site, or to expression of several growth factors. pS2 protein was localized immunocytochemically, to epithelia in the neck region of antral and body glands, in luminal secretions and in sub-nuclear regions of foveolar cells. Both dysplastic epithelium and tumour cells were heterogeneously stained. Immunocytochemically, oestrogen receptor was undetectable in 30 tissues examined or in 5 cell lines, two of which expressed pS2. A BamH1 polymorphism was observed in KATO III and MKN-28 cells. Thus pS2 expression, which may be associated with normal gastric secretory activity, is reduced in malignancy but is high during non-neoplastic hyperplasia, in complete contrast with its mode of expression in the breast. The pS2 protein sequence bears similarities to the pancreatic spasmolytic polypeptide and both contain a PRO-Tryp-Cys-Phe sequence found only in the Kringle domain of fibrinolytic proteases, leading to suggestions of common ancestral origins. So far no biological activity has been assigned to either the Kringles or to pS2.

**Detection by $^{32}$P-postlabelling of DNA adducts in the upper gastrointestinal tract of patients with familial adenomatous polyposis**

A.D. Spigelman[1], R.K.S. Phillips[1], D. Scates[2] & S. Venitt[2]

*[1]St Mark's Hospital & Professorial Surgical Unit, St Bartholomew's Hospital, London, UK; and [2]Institute of Cancer Research, Royal Marsden Hospital, Sutton, Surrey, UK.*

Duodenal adenomas occur almost inevitably in patients with familial adenomatous polyposis (FAP) whereas gastric adenomas are rare. FAP patients are also at high risk of duodenal cancer. Adenomas and most duodenal cancers cluster around the ampulla of Vater, suggesting that bile plays a role in tumour development (Spigelman *et al.* (1989), *Lancet*, **ii,** 783). To test the hypothesis that duodenal tissue in FAP is exposed to DNA-reactive substances we used $^{32}$P-postlabelling to determine adduct levels in DNA from duodenal biopsies from 25 controls and 39 FAP patients. The table shows relative adduct labelling (RAL) of duodenal DNA from controls and FAP patients, grouped by smoking habit.

| | *Controls* | | *FAP patients* | |
|---|---|---|---|---|
| Smokers | No[A] | Yes[B] | No[C] | Yes[D] |
| Number | 15 | 10 | 24 | 15 |
| | RAL values (adducts/$10^9$ nucleotides) | | | |
| Mean | 10.1 | 9.9 | 30.8 | 41.5 |
| Median | 6.0 | 10.0 | 13.0 | 26.0 |
| Range | 0–35 | 0–29 | 0–162 | 3–109 |

Mann-Whitney 1-tailed *P*-values: B≈A, 0.412; D>C, 0.033; C>A, 0.027; D>B, 0.0014.

Duodenal DNA from FAP patients had significantly higher adduct levels than DNA from controls. FAP patients who smoked had significantly higher DNA-adduct levels than those who did not smoke. These results indicate that duodenal DNA from patients at high risk of duodenal neoplasia carries a higher burden of DNA adducts than DNA from normal subjects, and that smoking appears to increase this load. The identity of duodenal DNA-adducts is at present unknown, as is the nature and origin of the substances involved in their formation. However, we suggest that bile is the most likely source, although pancreatic juice must also be considered, bearing in mind its route of secretion. DNA damage caused by high adduct levels in DNA may contribute to the genesis of duodenal neoplasia in FAP by causing loss, inactivation or mutation of the tumour-suppressor gene thought to be associated with this precancerous condition.

**Production of extracellular matrix degrading enzymes by human and rat bladder cancer cell lines *in vitro***

C.A. Parry & J. Chowaniec

*Department of Histopathology, U.C.M.S.M., London W1P 7LD, UK.*

Synthesis of extracellular matrix-degrading enzymes, particularly the metalloproteinases, has been shown to play a major role in tumour invasion (Liotta *et al.* (1988), *Breast Ca. Res. Treat.*, **11,** 113). We are currently investigating the secretion of metalloproteinases by a range of human and rat bladder cancer cell lines, EJ, T24, RT112, RT4 and RU-CL2, the *in vivo* growth potential, i.e., tumorigenicity, invasive and metastatic behaviour of which have been characterized previously in nude and/or immune-suppressed mice (Masters *et al.* (1986), *Cancer Res.*, **46,** 3630; Joy *et al.* (1989), *Br. J. Cancer*, **60,** 493) as well as their growth *in vitro* (Marshall *et al.* (1977), *J. Natl. Cancer Inst.*, **58,** 1743; Parry *et al.* (1990), *J. Pathol.*, in press). Serum-free media conditioned by cultures of cells in the exponential growth phase were concentrated using Amicon concentrators. Samples were then assayed for the presence of proteins using SDS polyacrylamide gel electrophoresis under non-reducing conditions. Irrespective of their species of origin or invasive/non-invasive behaviour *in vivo*, all cell lines secreted proteins with approximate molecular weights (MWs) of 74 kDa and 56 kDa. These values are close to those published for the MWs of type IV collagenase, vertebrate collagenase and stromelysin, as well as for plasminogen activating factors (Alexander & Werb (1989), *Curr. Opin. Cell Biol.*, **1,** 974) and the putative autocrine motility factor (AMF) proposed as a marker for invasive bladder cancer (Guirguis *et al.* (1988), *J. Natl Cancer Inst.*, **80,** 1203). Specific characterization of these two protein bands is now in progress, using electrophoresis of appropriate polyacrylamide substrate gels.

**The influence of the extracellular matrix on the growth, differentiation and chemosensitivity of HT29 human colon adenocarcinoma cells**

J.A. Gummer, K.M.S. Townsend, S.P. Langdon & J.A. Hickman

*Cancer Research Campaign Experimental Chemotherapy Group, Pharmaceutical Sciences Institute, Aston University, Birmingham B4 7ET, UK.*

Epithelial cells grow with a unique morphological polarization which is essential for their functional properties. HT29 cells grown on plastic substrata are characteristically anaplastic: the conditions of growth dictate against the development of functional polarity. When HT29 cells were implanted to the flank of immunodeficient mice they formed relatively well differentiated tumours exhibiting typical epithelial characteristics. We have subcultured HT29 cells for at least 10 passages on collagen rafts – either on plastic or floating in media. These cells exhibited tight junctions and microvilli, with a degree of polarization that was comparable with the xenografted tumour. The expression of the brush-border associated enzyme alkaline phosphatase was greater in these cells (Day 12 values were 125, 182 and 82 $\mu$M product mg$^{-1}$ protein for non-released collagen gels, released collagen gels and plastic respectively). There was an increase in the lag phase of growth when cells grown on collagen were compared to cells grown on plastic, but the exponential growth rate was unchanged. Although cells grown on collagen gels would appear to be morphologically and possibly functionally more differentiated than those growing on plastic substrata, we have shown that their chemosensitivity and clonogenicity to a range of standard cytotoxic agents was essentially unaltered. However, this system may represent an interesting model for *in vitro* studies of carcinoma cell biology and pharmacology.

**Neural cell adhesion molecular expression in human embryonic and brain tumours**

K. Patel, S. Bourne, H.B. Coakham & J.T. Kemshead

*ICRF Paediatric & Neuro-Oncology Group, Frenchay Hospital, Frenchay, Bristol BS16 1LE, UK.*

Neural cell adhesion molecules (NCAMs) are a family of glycoproteins believed to play an important role during development of the nervous system. A number of isoforms are generated through alternative splicing of mRNA transcribed from a single gene and through post-translational modification of the primary protein product. The expression of these isoforms is temporally and developmentally regulated. In addition, recent evidence suggests that tissue-specific NCAMs arise through insertion/deletion of short nucleotide sequences coded for by mini-exons. Recently, we have shown that monoclonal antibodies UJ13A and ERIC-1 recognize different epitopes on NCAM. This has led to a detailed investigation of NCAM expression in human embryonic and brain tumours since information in this important area of research is lacking. Northern blot analysis shows that neuroblastoma cell lines express predominantly 6.7 and 5.5 kb mRNA transcripts, whereas fetal brain expressed 7.4, 6.7, 5.5, 4.3 and 2.9 kb transcripts. The significance of this observation is unclear at present. Western blot analysis of the protein isoforms corroborates these findings. Changes in NCAM were also noted during human brain development and different areas of brain express different NCAM isoforms. Primary brain tumours also showed variability in NCAM expression. We are currently investigating the presence/absence of mini-exons, which give rise to tissue-specific NCAMs, in a variety of embryonic tumours, using the polymerase chain reaction.

### Vitronectin receptor expression on melanocytes and malignant melanoma cells

J.F. Marshall, M.H. Horton & I.R. Hart

*Imperial Cancer Research Fund, Lincoln's Inn Fields, London WC2A 3PX, UK.*

The level of expression of the vitronectin receptor (VnR), a member of the integrin family, has been investigated using a panel of 9 human melanoma cell lines and 4 normal melanocyte strains. The monoclonal antibodies, 13C2 and 23C6 were used in these studies (Davies *et al.* (1989), *J. Cell. Biol.*, **109**, 1817). Both antibodies immunoprecipitated a heterodimeric molecule of approximately 160 kDa ($\alpha$ chain) and 95 kDa ($\beta$ chain) size whose varying levels of expression correlated well with the ability of melanoma cells to adhere to vitronectin and fibrinogen substrates. Flow cytometry and immunoprecipitation analysis showed that considerably less VnR was expressed by melanocytes than by most of their transformed counterparts. Initial results suggest a correlation between subcutaneous growth as xenografts in athymic nude mice and the expression of higher levels of VnR suggesting that interactions between this integrin and substrate molecules may play a role in tumorigenesis. Staining of paraffin-embedded sections of human melanoma with 13C2 antibody showed that clinical material expressed high VnR levels. These results suggest that up-regulation of VnR levels may be associated with transformation of cells of the melanocyte lineage.

### Altered levels of expression of the gp90$^{Hermes}$ (lymphocyte homing receptor) cell surface molecule in transformed melanocytes

M. Birch, S. Mitchell & I.R. Hart

*Imperial Cancer Research Fund, Lincoln's Inn Fields, London WC2A 3PX, UK.*

The CD44 antigen is a 90 kDa cell surface glycoprotein which appears to be identical to the lymphocyte homing receptor recognized by the Hermes 1 and 3 antibodies. Increased expression of the CD44 antigen has been reported to occur in carcinoma, but not normal epithelial, cells (Stamenkovic *et al.* (1989), *Cell*, **56**, 1057). We have monitored the expression of the gp90$^{Hermes}$ antigen on a panel of 9 human melanoma cell lines and 6 strains of normal human melanocytes using CD44, Hermes 1 and Hermes 3 monoclonal antibodies. Immunoprecipitation of surface iodinated cells with all three antibodies revealed a 90 kDa band, indicating that cells of the melanocyte lineage express this receptor at their cell surface. Normal melanocytes appeared to express much lower levels of gp90$^{Hermes}$ than melanoma cells and this was confirmed by FACS and ELISA assays on viable and fixed cells. Northern blot analysis of mRNA, which revealed 4.8, 2.5 and 1.5 kb transcripts, suggested that this upregulation of surface glycoprotein associated with transformation was a consequence of transcription. It is possible that increased expression of CD44/gp90$^{Hermes}$ may facilitate melanoma cell adherence and play a role in metastasis.

### Changes in protein expression during melanoma differentiation determined by computer analysis of 2-D gels

D. Easty[1], I.R. Hart[1], K. Patel[2], C. Seymour[2], M. Yacoub[2], A Domscheit[3], S. Gunther[3], W. Postel[3], A. Gorg[3] & M.J. Dunn[2]

*[1]ICRF, Lincoln's Inn Fields, London, UK; [2]Department of Cardiothoracic Surgery, National Heart and Lung Institute, London, UK; and [3]Lehrstruhl fur Allgemeine Lebensmittel Technologie, Technische Universitat, Munich, Germany.*

Cytodifferentiation in many melanocytic cells is regulated through the adenylate cyclase-cAMP pathway. To study molecular changes associated with this process we have compared the proteins produced by two closely related cell lines which respond very differently to manipulation of this pathway. The human melanoma DX3 shows little change in *in vitro* characteristics following treatment with cAMP elevating agents; in contrast the more malignant LT5.1 variant, derived from the DX3 parental line, shows pronounced dendrification, decreased proliferation and a reduction in metastatic capacity after similar treatments. The two cell lines were treated with phosphodiesterase inhibitors (1 mM IBMX plus 1 mM theophylline) for 5 days and then processed for 2-D gel characterization using an immobilized pH gradient for IEF. Proteins were detected by silver densitometer and PDQ-scan software. 2-D gel patterns were matched and a melanoma protein data base of 637 spots constructed on an Orion 1/05 computer. Sixty-one proteins were downregulated in both cell lines after treatment, whereas 30 new spots were identified after treatment of DX3 cells and 52 new spots after treatment of LT5.1 cells. The 22 novel proteins identified may be associated with the observed changes in differentiation patterns and metastasis. Our results illustrate the resolving power of this technique and suggest potential applications to the study of cellular differentiation.

### Isolation of a novel gene expressed differentially in human melanoma lines of varying malignancy

D. Easty[1], C.J. Farr[2], J. Rao[1] & I.R. Hart[1]

*[1]Biology of Metastasis Laboratory and [2]Human Molecular Genetics Laboratory, ICRF, Lincoln's Inn Fields, London WC2A 3PX, UK.*

The rapid amplification of cDNA ends (RACE) procedure, using as a primer an oligonucleotide sequence conserved in genes encoding zinc-finger binding proteins, has been applied to polyA$^+$ mRNA derived from LT5.1, a human melanoma cell line. Amplified cDNAs were ligated into a Bluescript vector and 800 colonies were screened for differential hybridization using as probes either cDNA prepared from untreated control cells or from cells which had been induced to differentiate by treatment with phosphodiesterase inhibitors (1 mM IBMX plus 1 mM theophylline). Northern blot analysis was used to screen ten differentially hybridizing clones and one of these BS35, was found to be upregulated in the treated cell line (LT5.1) but not in the parental line (DX-3) which was unaffected by treatment with phosphodiesterase inhibitors. Southern blot revealed binding of BS35 to multiple bands, possibly suggesting it is one member of a multigene family. Partial DNA sequence data have not established significant homology with known genes. These results suggest that BS35 represents a novel gene, differentially expressed in human melanoma cell lines whose expression is regulated variably by cAMP in lines of distinct biological behaviour.

**The development of ribozymes to control oncogene expression**

P. Stephenson & I. Gibson

*School of Biology, University of East Anglia, Norwich, UK.*

The long term aim of antisense technology is the specific and controlled regulation of gene expression. Ultimately the hope would be that the control of oncogene expression could halt or even reverse tumour progression. In this study we have concentrated on the design and production of specific 'hammerhead' ribozymes directed against the N-*ras* oncogene. A full length CDNA clone of the MMTV/N-*ras* fusion gene which had been used to transform mouse 3T3 cells was transferred from $\lambda$ gt10 into a transcription vector so that mRNA-like transcripts could be produced in quantity for *in vitro* experiments. A subclone was also constructed which contained approximately 300 bp spanning the translation initiation site. To construct the ribozyme two complementary oligodeoxynucleotides were synthesized. These encoded the hammerhead consensus sequence (Haseloff & Gerlach (1988), *Nature*, **334**, 585; Forster & Symons (1987), *Cell*, **50**, 9), together with 10 bases on each side which would confer homology to the N-*ras* gene. These oligos were annealed and cloned into a vector such that transcription from the T7 promotor produced virtually pure ribozyme. *In vitro* this ribozyme cleaved the N-*ras* mRNA-like molecules in a very specific fashion to produce the predicted fragments at an optimun pH of 8.0 and with a requirement for magnesium ions. Pre-annealing the ribozyme and substrate molecules, or the inclusion of urea in the reaction mixture, caused a favourable reduction in the reaction temperature. The reaction is not inhibited by the inclusion of excess non-substrate RNA or high protein concentrations.

**Most cervical cancers progress to metastasis without the involvement of *ras* point mutations**

G. Willis[1], B. Jennings[1], R. Ball[2] & I. Gibson[1]

*[1]University of East Anglia, Norwich NR4 7TJ; and [2]Norfolk and Norwich Hospital, Norwich, UK.*

*Ras* point mutations are associated with many types of cancer. However, the stage of progression at which they occur has not been extensively studied. Comprehensive surveys of the genetic differences between primary and secondary tumours may lead to an understanding of genes involved in the development of the metastatic phenotype. We chose cervical cancer for our studies because tumour samples from Wertheim's hysterectomy patients are available from histopathology wax block archives and Riou *et al.* (1988, *Oncogene*, **3**, 329) claimed that mutation of c-Ha-*ras* correlated with late stage disease. Ten micro sections were cut from the primary and metastatic tumours of 16 patients. Areas of tumour were identified on haematoxylin and eosin stained slides and tissue scraped from adjacent, unstained sections. DNA from this tissue was amplified by PCR using primers flanking codons 12 and 13 of Ha K and N *ras* genes and codon 61 of K-*ras*. The amplified DNA was slot blotted and analysed by hybridization to probes specific for all possible amino acid changes at these codons. The Ha-*ras* PCR products were also analysed by digestion with restriction enzyme MSP I. *Ras* point mutations were found in only one of the sixteen patients. This patient had a point mutation in codon 13 of K-*ras* which was present in two metastases, but not in an area of invasive primary tumour or a third metastasis. These data strongly argue that *ras* point mutations are not a common phenomenon in patients with advanced cervical cancer and even when present appear to confer no strong selective advantage.

**Expression of the *myc*, *fos* and *ras* oncogenes during DMSO-induced detransformation of HT29 human colon cells**

S. Smart & I. Gibson

*School of Biology, University of East Anglia, Norwich, UK.*

The human colorectal cancer cell line, HT29, exhibits striking morphological changes upon exposure to 1.5% DMSO: The cells alter to a more flattened, elongated shape. The growth rate decreases several-fold and further investigation demonstrates a loss of clonogenicity of the cells and alterations in the arrangement of the actin stress fibres. Such a system is suitable for studies of detransformation. However, no changes indicative of differentiation were observed, such as brush-border formation or increase expression of the brush-border related enzymes, alkaline phosphatase and sucrose-isomaltase. A dramatic decrease in the level of carcinoembryonic antigen (CEA) occurred concurrently with a detransformation of the cells. Southern blotting experiments indicated low level (3–5 fold) amplification of the c-*myc* oncogene in the HT29 cells and in several clonal lines derived from the parent population RNA dotblots demonstrated reductions in C-*myc* expression within one hour of addition of DMSO and using a sensitive ELISA assay system the c-*myc* protein was found to be downregulated within two hours. Low levels of the *fos* oncoprotein (p55) were observed in all HT29 cells both before and after exposure to DMSO. Expression of the H-, K- and N-*ras* oncogenes at the levels of RNA and protein remained the same after exposure of the cells to DMSO. It was concluded that c-*myc* may be important in the detransformation of HT29 cells and preliminary experiments suggest that it may be possible to inhibit the expression of this oncogene using antisense oligodeoxynucleotides in order to study this role further.

**Measurement of c-*myc* protein in PHA stimulated lymphocytes by SDS PAGE electrophoresis and immunoblotting: perchloric acid pretreatment abrogates non-specific protein degradation**

D.G. Spiller, A.F. Chapman, D.M. Tidd & H.M. Warenius

*CRC Department of Radiation Oncology, University of Liverpool, Clatterbridge Hospital, Wirral, L63 4JY, UK.*

We have previously reported that *myc* protein was only detectable in lymphocytes 72 hours after stimulation with PHA (1989, *Br. J. Cancer*, **60**, 480). We have since added recombinant *myc* protein immediately before lysis with conventional lysis buffer (10% SDS, 10% glycerol, 0.1 mM leupeptin, 0.1 mM phenylmethylsulphonyl fluoride in 0.04 M Tris, pH 6.8), and discovered that the protein was degraded. Examination of SDS PAGE gels stained for protein with Coomassie Blue suggested that there was non-specific protein degradation and the degradation was more severe in fresh and also in unstimulated cultures. Boiling lysates immediately after cell lysis increased the proportion of high molecular weight proteins, but added *myc* protein was still degraded. Pretreatment of cells with 4.2% w/v perchloric acid followed by washing and dissolving the precipitated protein in normal lysis buffer completely abrogated *myc* protein degradation and again increased the proportion of high molecular weight proteins. Using this method for preparing lymphocytes extracts *myc* protein was detectable in unstimulated lymphocytes and there was a rise in *myc* protein concentration shortly after PHA addition. We conclude that lymphocytes contain factors which rapidly degrade protein that are not suppressed by the protease inhibitors in conventional lysis buffers and perchloric acid pretreatment can be used to eliminate non-specific protein degradation before SDS PAGE electrophoresis and immunoblotting.

**Secondary transfection of metastatic capability and confirmation by PCR of concomitant transfer of donor (human) DNA**

M. Cairney, A. Taylor & D. Tarin

*Nuffield Department of Pathology, University of Oxford, John Radcliffe Hospital, Headington, Oxford OX3 9DU, UK.*

Previous work at this laboratory provided evidence indicating transfer of metastatic capability by transfection of mouse tumour cells with total genomic DNA from human metastatic melanoma (A375M) cells. The transfected cells heavily colonized the lungs in 84% of inoculated animals. Human DNA could be detected in the primary transfectants (designated AH8 test cells) by Southern blotting and by *in situ* hybridization using human specific probes but the demonstration of such exogenous DNA was difficult and capricious indicating that the amount of human DNA incorporated was at the limit of resolution by standard techniques. In our subsequent work the sensitivity of the polymerase chain reaction (PCR) has been used therefore to verify the transfer of donor (human) DNA by selective amplification of human specific sequences. The results reproducibly show the presence of donor DNA in the primary transfectant line. In the current work we report secondary transfection of heavy metastatic capability with DNA from experimental lung metastases formed by the primary transfectants. The DNA designated AH8 LMC was transfected with the calcium phosphate precipitation technique into the same mouse fibrosarcoma line (TR4 Nu) used as the recipient in the primary transfection. The secondary transfectants were inoculated into nude mice via the tail vein and heavily colonized the lungs in 85% of animals. Using the PCR and primers specific for human Alu repeat sequences we have also reproducibly detected concomitant transfer of human DNA into the secondary transfectants. The results provide further evidence that metastatic capability can be conferred by transfer of genomic DNA and provide a reliable data base for efforts to clone the exogenous sequences and test whether they have a specific causal role in the new behaviour induced.

**Oestrogen-receptor (ERD5) in colorectal carcinoma**

J.F.R. Robertson, D.L. Morris, I.O. Ellis & N.C. Armitage

*Departments of Surgery and Pathology, University Hospital, Nottingham, UK.*

Oestrogen receptor status by the ERD5 method is an independent prognostic factor in gastric cancer. We have studied ERD5 immunocytochemical expression in 68 primary colorectal cancers. Tumours were regarded as positive for ERD5 if any of the tumour cells showed immunostaining. ERD5 status was correlated with other tumour characteristics, recurrence and survival. Fifty-six per cent of the tumours stained positive for ERD5. ERD5 status did not correlate with the sex of the patient ($P = 1.000$). ERD5 status did not correlate with stage of disease ($P = 0.16$) histological grade ($P = 0.45$), tumour cell proliferation (Ki67 antigen) expression ($P = 0.29$), DNA ploidy status ($P = 0.37$) or the site in the colon of the primary tumour ($P = 0.31$). There was also no correlation with the sex of the patient (1df; $P = 1.00$). ERD5 status showed no significant correlation with time to recurrence for the whole group ($P = 0.62$), for men only ($P = 0.6$) or for women only ($P = 0.7$). ERD5 status did not correlate with overall survival ($P = 0.51$). In conclusion, oestrogen receptor was expressed in 56% of colorectal cancers in this study compared to 55% of gastric cancers. However, unlike gastric cancer, oestrogen expression in colorectal primary tumours was of no prognostic significance.

**A rapid and efficient method for separating infiltrating cells and tumour cells from colorectal tumours**

L.G. Durrant & R.W. Baldwin

*Cancer Research Campaign Laboratories, University of Nottingham, Nottingham NG7 2RD, UK.*

As solid tumours are composed of a heterogeneous mixture of cells, the role of any particular cell type is difficult to analyze. A rapid method of sorting freshly disaggregated highly viable single cell suspensions of colorectal tumours has therefore been developed. Infiltrating cells were initially sorted from tumour cell suspensions using magnetic beads coated with a cocktail of monoclonal antibodies recognizing leucocyte common antigen (lymphocytes), CDw14 (macrophages and granulocytes) and thy1 antigen (stromal cells). Colorectal tumours are composed of a 1:1 mixture of infiltrating cells:tumour cells. Between $4-10 \times 10^6$ cells, 70- 87% pure were rapidly sorted. The tumour cells were then sorted from the remaining cell suspension using a cocktail of monoclonal antibodies which recognize 100% of colorectal tumours. Between $2-7 \times 10^6$ tumour cells, 70-87% pure were rapidly sorted. Both populations of cells are 95% viable and grow *in vivo*.

**Pattern of ornithine decarboxylase (ODC) activity in colorectal mucosa of normal subjects and of patients with adenomatous polyps or cancer of both sexes**

S. Bellentani, G. Saccoccio, C. Armocida, A. Grisendi, A.M. Ferrari, F. Manenti & M. Ponz de Leon

*Catt. Gastroent., Ist. Patol. Med. & Div. Med. Int., Modena, Italy.*

ODC activity is usually taken as a good index of cell proliferation. The aims of our study were to define the normal pattern of ODC activity in different tracts of the large bowel in both sexes and to compare ODC activity in the apparently normal mucosa of patients with polyp, or cancer. ODC activity in the colorectal biopsies taken from 33 individuals was measured by release of $^{14}CO_2$ after incubation of the colonic homogenate with $^{14}$C-L-ornithine for 30 minutes. Results showed that: **1.** no difference was present in ODC colonic mucosa activity of controls neither between males and females, nor between right and left colon; **2.** ODC activity in apparently normal mucosa was significantly higher ($P<0.05$) in patients with polyps or tumours ($1.3 \pm 0.6$ nM $CO_2$ $mg^{-1}$ protein $h^{-1}$) than in controls ($0.6 \pm 0.4$) in both sexes, with a trend to be higher in females; **3.** in patients with adenoma, ODC activity was much lower in biopsy taken directly from the polyp ($0.3 \pm 0.1$) than those taken either in the close vicinity ($1.4 \pm 0.7$, $P<0.01$), or at distance from the mass. We conclude that ODC activity may provide a good biological marker to detect subjects at high risk for large bowel cancer.

**Immunological and biophysical studies on the peptide repeat of polymorphic epithelial mucins**

M.J. Scanlon[1,2], M.R. Price[1] & S.J.B. Tendler[2]

[1]*Cancer Research Campaign Laboratories; and* [2]*Department of Pharmaceutical Sciences, University of Nottingham, Nottingham NG7 2RD, UK.*

The protein core of human polymorphic epithelial mucins consists of a repeated sequence of 20 amino acids (Gendler *et al.* (1988), *J. Biol. Chem.*, **263,** 12820) containing an immunodominant region composed of seven amino acids (P D T R P A P). Secondary structure predictions and hydropathicity calculations indicate that this seven amino acid sequence should include a hydrophilic turn region of the protein which would be accessible to recognition and binding by antibodies. A series of synthetic peptides were prepared based upon the repeat sequence and those containing the immunodominant domain were shown to bind to several monoclonal antibodies which are reactive with epithelial mucins (i.e., anti-breast carcinoma antibodies, anti-human milk fat globule antibodies). To probe the structural aspects of the immunodominant domain, high field NMR studies were undertaken on an antigenic eleven amino acid fragment (P D T R P A P G S T A). A type I β-turn was identified between $Asp^2$ and $Arg^4$ and this turn is further extended by the $Arg^4$ to $Pro^5$ bond being in the *trans* conformation. The NMR studies confirm the secondary structure predictions and may indicate that these elements of secondary structure are recognized in breast carcinoma associated mucins by monoclonal antibodies which react with a variety of human carcinomas including breast tumours.

**Familial adenomatous polyposis: clinical and epidemiological features from 22 families**

M. Ponz de Leon, R. Sassatelli, C. Sacchetti & G. Zanghieri

*Istituto di Patologia Medica, Policlinico, Modena 41100, Italy.*

Familial Adenomatous Polyposis (FAP) is a genetic disease with an autosomal dominant transmission. FAP is characterized by the presence of more than 100 polyps in the large bowel and by several extracolonic manifestations. Colorectal cancer develops in almost all untreated patients. We describe the main clinical and genetic features of 22 families with FAP from Northern Italy; 269 unaffected and 84 affected family members were studied. Gene frequency at birth was estimated in the order of 1:7,000–1:20,000. In affected branches the segregation ratio was 0.38, close to the 0.50 theoretically expected for dominant transmission. The penetration of the gene was 63% and the male:female ratio 44:40. All affected individuals with symptoms already had a colorectal cancer at diagnosis, whereas no malignancy was usually found in patients in whom diagnosis was made before the appearance of symptoms. Extracolonic changes were detected in all families, the most frequent being cutaneous cysts and retinal lesions. In conclusion, the gene frequency of FAP in Italy seems similar to that reported in other countries. Early diagnosis, before the appearance of symptoms, is essential for adequate surgical treatment. The frequency of extracolonic manifestations suggests that clinical studies should not be limited to the large bowel but should also include the skin, the upper digestive tract, several bones and the retinal epithelium.

**Cell kinetics and DNA ploidy in colorectal cancer: relation to grading, staging and site of tumours**

M. Ponz de Leon, A. Costa, A. Scalmati, L. Roncucci, G. Colella, S. Veneroni & R. Silvestrini

*Istituto di Patologica Medica, Univ. Modena; and Istituto Nazionale Tumori, Milan, Italy.*

The prognostic value of cell kinetics has been well established for many tumours whereas its importance in colorectal cancer is still unknown. Therefore, we evaluated S-phase fraction and DNA ploidy in 72 consecutive colorectal cancers and related the observed findings to the most predictive clinical parameters. Specimens of neoplastic tissue were obtained at surgery and processed by a standard autoradiographic technique; in 39 cases tumour cell suspensions were stained with propidium iodide and analysed for DNA ploidy in a FACS-STAR flow cytometer. Labelling Index (LI, % of labelled cells, i.e., the proliferative fraction) was $20.0 \pm 1.3$ SEM. LI tended to increase in the more distal portions of the large bowel (from 17.1 to 25.5%) though not significantly. Grading was unrelated to LI, whereas staging was inversely related to the S-phase fraction (Dukes' A + B $21.5 \pm 1.5$, C $22.7 \pm 3.1$, D $15.2 \pm 1.2$, $P < 0.01$, A + B or C versus D). Ploidy was unrelated to tumour site, grading or LI. Aneuploidy, however, was more frequent in metastatic (Dukes' C–D) than in localized tumours (15 out of 17 versus 13 out of 22, $P < 0.05$). In conclusion, our findings suggest that in advanced colorectal cancer cell proliferation tends to decrease, while aneuploidy becomes more frequent. Moreover, cell proliferation seems more active in neoplasms of the distal large bowel, which seem to be biologically and epidemiologically different from tumours of the right colon.

**Genetics of large bowel cancer: evidence for an autosomal recessive type of transmission**

M. Ponz de Leon, C. Scapoli, R. Sassatelli, G. Zanghieri, C. Sacchetti & I. Barrai

*Istituto di Patological Medica, Univ. Modena and Istituto Zoologia, Univ. Ferrara, Italy.*

With the exception of a few well defined conditions (Adenomatosis Coli and Lynch syndrome) the contribution of heritable factors to the pathogenesis of the commonly observed colorectal cancer is still poorly defined. We tested several hypotheses of genetic transmission of this malignancy by critically analysing the clinical data of a population-based Cancer Registry. For each patient registered in 1984–86 for colorectal cancer in the local Health District a careful genealogical tree was traced. Information was obtained on 390 incident cases (the probands) and on a total of 2,851 first-degree relatives. Classical segregation analyses were carried out on the whole series according to the maximum likelihood method. After exclusion of a few families with clinical evidence of Cancer Family Syndrome, segregation analysis showed that the hypothesis of one recessive allele at one locus with reduced penetration (both in intercrosses and in backcrosses) did not deviate from the maximum likelihood ($\chi^2$ 1.1–0.7 and 0.8 respectively for probability of ascertainment of 1.0–0.8 and 0.6). All other tested hypotheses deviated significantly from the maximum likelihood. In conclusion, our data suggest that the susceptibility to colorectal cancer in the majority of the affected population may be due to a recessive gene with reduced penetration.

**Human colorectal cancer, but not breast cancer, shows a high frequency of chromosomal telomere shortening**

A.M. Thompson[1,2], M.G. Dunlop[1,2], M. Dempster[2], C.A. Purdie[3], R.C. Allshire[2] & N.D. Hastie[2]

[1]*University Department of Surgery, Royal Infirmary, Edinburgh;* [2]*MRC Human Genetics Unit, Western General Hospital, Edinburgh; and* [3]*University Department of Pathology, Teviot Place, Edinburgh, UK.*

Understanding the fundamental molecular mechanisms of carcinogenesis may provide accurate prognostic indicators and might lead to novel approaches to treatment. We report the first demonstration of a high frequency of shortening of chromosomal telomeres in colorectal cancer. The terminal 10–15 kilobases of normal human telomeric DNA comprise repeat sequences which maintain the structural integrity of each chromosome. We have examined telomere length in blood/tumour DNA pairs from 52 colorectal and 50 breast carcinomas. Twelve adenomas and 12 normal colonic mucosa samples were also analysed. DNA was digested with restriction endonuclease, gel fractionated, Southern blotted on to nylon membranes, radiolabelled telomere DNA probe hybridized to the DNA on the membranes and densitometric analysis of autoradiographs performed. Eighty-eight per cent (45/52) of colon cancers and 12% (6/50) of breast cancers demonstrated severe telomeric shortening compared to blood DNA controls. Adenomas and normal mucosa did not show such changes. In colon cancer, telomeric shortening showed no correlation with tumour site, size or stage, ploidy level by flow cytometry, or allele losses from chromosomes 17p, 18q and 5q. Similarly, no correlation between telomere size and clinicopathological parameters was apparent in breast tumours. These data suggest structural chromosomal changes are important in colorectal carcinogenesis but not in breast carcinoma.

**Site-directed integration of selectable gene in the adenomatous polyposis coli (APC) locus on chromosome 5**

H. Sasai & W.F. Bodmer

*Director's Laboratory, Imperial Cancer Research Fund, 44 Lincoln's Inn Fields, London WC2A 3PX, UK.*

As an alternative strategy for mapping and cloning the APC gene, we are using techniques of gene targetting by homologous recombination to integrate dominant selectable genes near this locus. Targetting vectors have been made using the close marker sequences D5S98 and D5S71 containing neo-resistant gene and HSV-TK gene. Following transfection into a human chromosome 5 containing CHO hybrid, selection was then achieved using G418 and ganciclovir. Successful targetting was checked using appropriate primers for PCR. The integrated region can then be transfered using X ray irradiation hybrids into further recipient cells for subsequent isolation and characterization.

**Abnormalities in the P53 gene in colorectal cancer**

N. Rodrigues, A. Rowan, M. Smith, I.B. Kerr, D. Lane & W. Bodmer

*Imperial Cancer Research Fund, PO Box 123, Lincoln's Inn Fields, London WC2A 3PX, UK.*

The implication of the P53 gene as a possible suppressor oncogene has come about since the reports of point mutations in the coding region of the gene in many cancers, including colon, breast and lung cancers, which show chromosome 17p allele loss. We have used an antibody PAb240 that recognizes an evolutionary conserved epitope on p53, to study colorectal carcinomas. On immunocytochemical staining, 13 out of 28 colorectal carcinomas showed increased reactivity for presumably a mutant form of p53, and of these, 3 were only focally reactive. None of the 10 adenomas from both polyposis and sporadic cases showed any p53 staining. Elisa assays on cell lines from colorectal carcinomas show greater reactivity with PAb240 than with PAb421, an anti-p53 antibody in 19 out of the 24 cell lines studied, while the reverse was observed in lymphoblastoid cell lines with putative normal p53 expression. We further analysed some of the colorectal and lymphoblastoid cell lines in order to correlate the antibody staining with the presence of a mutation. We found that there was homozygous expression of mutant p53 mRNA by using an asymmetric polymerase chain reaction based mRNA sequencing strategy.

**Detection of allele loss in ovarian cancer**

B.J. Milner[1], N.E. Haites[1], K.F. Kelly[1], M. Hall[2], H. Kitchener[2] & A.W. Johnston[3]

*[1]Department of Genetics and Microbiology, University of Aberdeen; [2]Aberdeen Maternity Hospital; and [3]Ward 3/4, Aberdeen Royal Infirmary, Aberdeen AB9 2ZD, UK.*

DNA has been analysed from eleven benign ovarian cysts, a familial malignant ovarian carcinoma, and one metastatic nodule from a previously removed ovarian carcinoma. Using Jeffrey's minisatellite and chromosome 17p VNTR (variable number of tandem repeat) probes, tumour and normal DNA have been compared. DNA from the familial tumour demonstrated a loss of heterozygosity at the 17p locus detected by VNTR probe pYNZ22. Before this finding, a 6 kb fragment loss had been seen in the tumour fingerprint of the same individual, using Jeffreys' 33.15 probe. Steps are currently being taken to identify the chromosomal location of this sequence. The metastatic nodule also demonstrated 17p loss of heterozygosity using the pYNZ22 probe. None of the benign tumours were seen to lose 17p alleles at this locus. This has led us to suggest that loss of function of an anti-oncogene from 17p, such as P53, could be involved in the transition from the benign to the more malignant stages of ovarian cancer.

**Cloning of mRNA sequences from human pancreatic adenocarcinoma and identification of differentially expressed cDNA**

R. Flomen, D. Grant & A. Grant

*Department of Surgery, St. George's Hospital Medical School, London SW17 0RE, UK.*

A λgt11 expression library prepared from a human pancreatic ductular adenocarcinoma cell line (GER) has been screened with mRNA derived from normal and tumour cells in order to identify sequences which are unique to either the transformed, or normal pancreatic cell. Five clones have been selected (GER 1–5) and amplified by the polymerase chain reaction (PCR) using Taq polymerase and 24 mer primers complementary to the β-galactosidase encoding regions which flank the E coR1 cloning site. Two of the inserts (λGER2, 1.8 kb and λGER4, 1.6 kb) were used to prepare cDNA probes for Northern blot analysis of RNA isolated from a variety of tumours and normal tissue. λGER2 identified a major band of 1.7 kb in mRNA derived from both the GER cell line and other pancreatic tumour biopsies, but absent from normal pancreas. Although this transcript was associated with the transformed state it was not pancreas specific since it was also present in a number of other tumours. Identical Northern blots probed a 1.1 kb restriction fragment from λGER4 gave a different pattern of hybrization. This probe hybridized to high molecular weight mRNA species in both normal and neoplastic pancreatic tissues that were not present in mRNA derived from other tumours. These inserts are now being subcloned into M13 for sequencing by the dideoxy method or primer directed PCR sequencing.

**Isolation of a cDNA sequence homologous to HSP 86 and its expression in human breast**

A. Jameel, Y.A. Luqmani, R.A. Skilton, T. Campbell & R.C. Coombes

*St George's Hospital Medical School, London SW17 0RE, UK.*

To identify potential markers for breast cancer, a human breast cDNA library constructed using mRNA from lymph node metastases was screened with a rabbit polyclonal antisera raised to deglycosylated breast metastases membranes. From a primary screen of $3.3 \times 10^5$ pfu, 6 immunopositive clones have been isolated. One of these clones, AJ1, was analysed in more detail; a partial sequence and a computer aided homology search of the predicted amino acid sequence in the Swiss protein data bank showed complete identity with amino acids 226-311 of the human HSP86. Using $^{32}P$ labelled AJ1, we studied its expression in various human tissues by Northern and dot blot hybridization. A single 3.3 kb band was observed in all cases. It was highly expressed in stomach and colon and to a much lesser degree in thyroid, adrenal, ovary, lung and muscle and undetectable in specimens of bladder, skin, placenta and spleen. In the breast, we observed varying degrees of hybridization in all of 54 primary cancers examined; in contrast, 10 non-malignant samples taken adjacent from tumours expressed this gene to a much lesser extent. Similarly, high levels were found in several breast cancer cell lines but low amounts in the BHL100 line. Interestingly, however, mRNA from 4 separate organoids made from normal reduction mammoplasties had levels similar to the cancers and much higher than in unprocessed normal tissues. Preliminary results using the MCF-7 cell line indicated that HSP86 message could be induced several fold by incubation at 42°C, reaching a peak and declining within 24 hours. We also found that oestradiol could increase transcription in a time-dependent manner, similar to that observed for mouse uterus (Shyamala *et al.* (1989), *Mol. Cell Biol.*, **9**, 3567). The function of heat shock proteins is somewhat obscure, but there is increasing evidence to suggest an important role in steroid receptor regulation, which will be studied in MCF-7 cells using this clone.

### Cloning and characterization of the human topoisomerase II promoter

D. Hochhauser, C. Stanway, A.L. Harris & I. Hickson

*Molecular Oncology Laboratory, Imperial Cancer Research Fund, Institute of Molecular Medicine, John Radcliffe Hospital, Headington, Oxford OX3 9DU, UK.*

Topoisomerase II is a key target for certain anticancer drugs, such as adriamycin and more specifically etoposide (VP16). A possible reason for tumour sensitivity to these drugs could be over expression of topoisomerase II. Therefore, we decided to study the regulation of topoisomerase II expression. A cosmid library derived from placental tissue was screened, with a radiolabelled 24-mer oligonucleotide homologous to the 5′ end of the published cDNA sequence. A genomic clone was isolated encompassing the putative promoter and 5′ region of the gene. Sequence analysis revealed a series of potential promoter elements including AP1 and Sp1 sites. Mapping of the 5′ end of the topoisomerse II mRNA also suggests that this region may function as a promoter. We have also examined the transcriptional response of the promoter to cellular transformation by SV40 and also to serum starvation; both of which are known to alter topoisomerase II expression.

### Stress and cancer surveys: attitudes of participants in a case-control study

P. Trowbridge, C. Taylor & C. Chilvers

*Section of Epidemiology, Institute of Cancer Research, Sutton, Surrey SM2 5NG, UK.*

It has been suggested that both cases and healthy controls may be inadvertently subjected to stress when they are included in a 'cancer survey'. Case-control studies requiring personal interviews are common and both doctors and ethics committees frequently express concern about the effect of participation on their own patients and healthy controls. In studies of cancer aetiology, controls from the general population rather than hospitalized controls are often required. A postal questionnaire was sent to women previously interviewed in a case-control study of the aetiology of cervical cancer. The questionnaire covered attitudes to taking part in the study, stress engendered by participation, whether any particular questions were distressing, factors relevant to the decision to participate, and the role of their doctor with respect to participation. Responses were received from 110 cases and 116 controls, a participation rate of 87%. Nearly all respondents were glad that they had participated while only 2/226 regretted taking part. Half the responders (115/226) perceived some actual benefit from taking part. The interview carried out in the case-control study was both long and detailed and included topics such as numbers of sexual partners and history of sexually transmitted diseases. As expected, the questions causing most concern to interviewees were those on number of sexual partners, but only 13% of participants were bothered by these questions and only 4% felt inclined to terminate the interview early. The lack of evidence of stress caused by this potentially difficult interview, suggests that, in the hands of experienced interviewers, stress is unlikely to be caused by participation, and many participants actually feel that they have benefited from taking part. Doctors and ethics committees should find these results reassuring.

### Inheritance of gastric cancer: data from a population-based registry

M. Ponz de Leon, G. Zanghieri, C. Sacchetti & R. Sassatelli

*Istituto di Patologia Medica, Policlinico, 41100 Modena, Italy.*

Gastric cancer still represents epidemiological and pathogenetic enigma. This tumour has usually been related to environmental factors, whereas less attention has been paid to the possible relevance of genetic factors. In 1984 a Tumour Registry was instituted in our Health Care District and this gave us the possibility of investigating the importance of hereditary factors. The purpose of the present study was, therefore, to describe the frequency of gastric cancer among first-degree relatives of 154 patients with tumours of the stomach registered in 1986 and 1987, and among relatives of 154 controls matched for age and sex. Crude gastric cancer incidence was 28.2/100,000/year. Among 907 first-degree relatives of registered patients, 30 cases of cancer of the stomach were observed versus 15 cases among 991 control relatives (Mantel-Haenszel $\chi^2$ with Liang's correction = 5.93, $P<0.01$, odds ratio 3.14). This excess of cases was particularly evident among siblings (17 vs. 7, M-H $\chi^2=4.23$, $P<0.02$) whereas it was not significant among parents (13 vs. 8). A family history of gastric cancer was appreciably more frequent in diffuse than in intestinal carcinoma. No excess of other malignancies was observed among relatives of incident cases. Our results therefore suggest that gastric cancer is significantly more frequent among close relatives of patients affected by cancer of the stomach. A genetic susceptibility to gastric cancer might be supposed as for other common malignancies.

### A cytogenetic/histological study in non-Hodgkin's lymphoma with particular reference to t (14,18)

J.A. Radford[1], S. Murray[1], C. Harrison[3], P. Bishop[2], M. Harris[2] & D. Crowther[1]

[1]*CRC Department of Medical Oncology;* [2]*Department of Histopathology, Christie Hospital; and* [3]*CRC Department of Cytogenetics, Paterson Institute for Cancer Research, Manchester, UK.*

Concurrent histological and cytogenetic examination has been performed on 64 lymph node biopsy specimens collected between January 1981 and January 1989. The trypsin-Giemsa banding technique was employed after short term cell culture and cytogenetic abnormalities were recorded when found in at least 20 cells. Review of paraffin embedded H and E sections supplemented as necessary by special stains and immunohistochemistry was also carried out and classification made according to the Keil system. Twenty-five of 64 (39%) cases had cb/cc follicular (F) or follicular and diffuse (FD) histology and 10 (16%) were cb/cc diffuse. Twenty-nine of 64

(45%) were other forms of NHL (immunoblastic 9, centroblastic 8, ML unclassified 5, centrocytic 4, lymphocytic 3). Twenty-two of 64 (34%) cases had t(14,18) and of these, 15 had a concurrent cb/cc (F) histology and 2 were cb/cc (FD). Of the remaining 5 cases, 3 had a cb/cc (F) or (FD) pattern on a previous biopsy. Eight of 25 (31%) pts had (F) or (FD) histology but no t(14,18) and a further 3 pts without the translocation had a (F) or (FD) pattern on previous biopsy. Overall, 20 of 22 (91%) cases with t(14,18) had a (F) or (FD) histological pattern at some stage in their history. Details of time to first recurrence, number of relapses and overall survival were available for 17 of 20 (F) or (FD) pts with t(14,18) and 10 of 11 (F) or (FD) pts without the cytogenetic marker. With a median follow up of 56 mths, 6 of 17 pts with t(14,18) have died (3 from follicular NHL, 2 from diffuse NHL and 1 from AML) against 5 of 10 pts where t (14,18) was absent (2 from follicular NHL and 3 from diffuse NHL). This is a significant difference between the 2 groups for deaths from NHL ($P = 0.02$) but relapse free survival is the same for both groups ($P = 0.23$). From this small study we conclude that in (F) or (FD) non-Hodgkin's lymphoma, the presence of t(14,18) is associated with a significantly better prognosis than where t(14,18) is absent. Larger studies are required to confirm these findings.

**The role of flow cytometric nucleoid analysis in predicting a response to radiotherapy in patients with bladder cancer**

T.H. Lynch, P. Anderson, A.T.M. Vaughan, D.G. Gordon, R.P. Beaney & D.M.A. Wallace

*Department of Urology, Queen Elizabeth Medical Centre, Birmingham, UK.*

Flow Cytometric Nucleoid Analysis (FCNA) is a new technique used by cell biologists to detect radiation damage to cells *in vitro*. When cells are placed in a highly concentrated salt solution, the nuclear envelope and histone proteins are removed leaving the nucleoids. Nucleoids extracted from cells which have been exposed to radiation are larger than those extracted from non-irradiated cells. The amount of expansion correlates with the known radiosensitivity of certain cell lines. The purpose of this study is to determine if the technique of FCNA can be applied to muscle invasive transitional cell carcinoma of the bladder, to differentiate those tumours that are radiosensitive from those that are radioresistant.

We analysed tumour specimens from 100 patients with invasive bladder cancer before radiotherapy. Tumour samples were received from either transurethral resection samples or urine. All specimens were analysed fresh without any form of fixative. Single cell suspensions were prepared and irradiated with a cobalt 60 gamma source and analysed on the flow cytometer. The degree of nuclear expansion is compared to the subsequent local tumour response at 3 and 6 months following radiotherapy. We have initial results on follow up in 30 patients at 3 months after completion of radical radiotherapy which show a correlation of radiosensitivity *in vitro* as determined by nucleoid expansion with the response of the patient to treatment. All tumours showing a nucleoid expansion of greater than 10% responded to radiotherapy. This technique can be applied using voided urine or TUR samples of tumour and gives a result within hours. Therefore, it may be of practical benefit in selecting patients for radical radiotherapy as opposed to other treatment modalities.

**α-Fucosidase (ALF) as a marker of genetic deletion in ovarian carcinomas**

J.E. Roulston[1], J.M. Richardson[1], J. Fisken[1] & R.C.F. Leonard[2]

[1]*Department of Clinical Chemistry, Royal Infirmary; and* [2]*Department of Clinical Oncology, Western General Hospital, Edinburgh, UK.*

Wells *et al.* (*Cancer Genetics, Cytogenetics*, **25**, 247) have reported suppression of serum α-1-fucosidase in hereditary EOC. The gene located on 6qter may thus be a target for genetic deletion and in more recent studies using Southern blotting techniques on tumour and blood DNA, other workers and ourselves have found deletions at 6q, 17p and 11p. We have therefore examined the activity of α-1-fucosidase in the serum of 199 normal subjects (blood donors) compared against 147 patients with EOC. ALF activity was measured as the change in absorbance control of paranitrophenyl-α-1-fucoside as a substrate. The range of activity in both test and control populations measured as absorbance units was between 0 and 2.6. No assumption was made about the normality of distribution of data in either set and so the results were compared non-parametrically. By this analysis there was no difference between the two groups. We conclude that there is no measurable reduction in activity of this gene product in the serum of patients with common epithelial ovarian carcinoma. Therefore, it is not a good screening target for population studies. It does not, however, exclude the possibility that a small subset of patients have lower levels of ALF in association with hereditary predisposition.

**Clinical trials – a game of chance?**

M. Stewart, R. Rye & A. Young

*ICRF Medical Oncology Unit, Western General Hospital, Edinburgh, Scotland EH4 2XU, UK.*

The management and monitoring of data in any clinical trial can be fraught with difficulties. From its inception through to the published paper there are many pitfalls to avoid. This poster aims to illustrate both the problems in running a trial and possible solutions through the analogy of the board game 'snakes and ladders'. Progress through the squares on the board is determined by landing on a square with a ladder leading upwards or a snake leading downwards. Ladders would thus represent events which would take the player closer to the end point of the trial such as the protocol being passed by the local ethics committee, or adequate patient numbers being accrued to the trial. Snakes by contrast represent problematic events. These would include blood tests not being done according to protocol, or X-rays missing for an external audit. While on one hand more resources than ever before are collectively facilitating progress towards improving cancer treatments, on the other it is almost certain that a clinical trial will never go completely according to plan.